# Beyond Retigabine: Design, Synthesis, and Pharmacological Characterization of a Potent and Chemically Stable Neuronal Kv7 Channel Activator with Anticonvulsant Activity

**DOI:** 10.1021/acs.jmedchem.2c00911

**Published:** 2022-08-16

**Authors:** Simona Musella, Lidia Carotenuto, Nunzio Iraci, Giulia Baroli, Tania Ciaglia, Piera Nappi, Manuela Giovanna Basilicata, Emanuela Salviati, Vincenzo Barrese, Vincenzo Vestuto, Giuseppe Pignataro, Giacomo Pepe, Eduardo Sommella, Veronica Di Sarno, Michele Manfra, Pietro Campiglia, Isabel Gomez-Monterrey, Alessia Bertamino, Maurizio Taglialatela, Carmine Ostacolo, Francesco Miceli

**Affiliations:** ‡ Department of Pharmacy, University of Salerno, Via G. Paolo II 132, Fisciano 84084, Salerno, Italy; § Department of Neuroscience, Reproductive Sciences and Dentistry, University Federico II of Naples, Via Pansini, 5, Naples 80131, Italy; † Department of Chemical, Biological, Pharmaceutical and Environmental Sciences (CHIBIOFARAM), University of Messina, Viale Ferdinando Stagno d’Alcontres 31, Messina 98166, Italy; ∥ Department of Science, University of Basilicata, Via dell’Ateneo Lucano 10, Potenza 85100, Italy; ¥ Department of Pharmacy, University Federico II of Naples, Via D. Montesano 49, Naples 80131, Italy

## Abstract

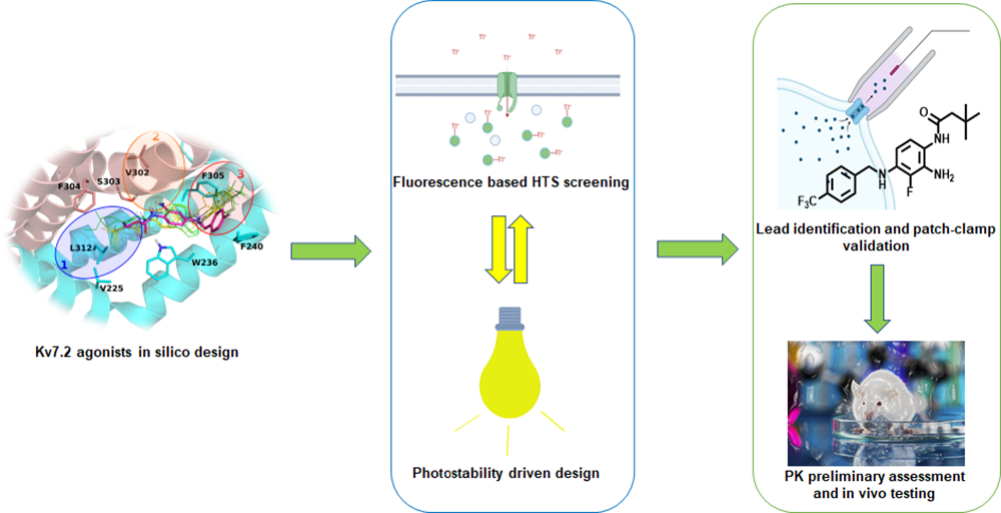

Neuronal Kv7 channels represent important pharmacological targets for hyperexcitability disorders including epilepsy. Retigabine is the prototype Kv7 activator clinically approved for seizure treatment; however, severe side effects associated with long-term use have led to its market discontinuation. Building upon the recently described cryoEM structure of Kv7.2 complexed with retigabine and on previous structure–activity relationship studies, a small library of retigabine analogues has been designed, synthesized, and characterized for their Kv7 opening ability using both fluorescence- and electrophysiology-based assays. Among all tested compounds, **60** emerged as a potent and photochemically stable neuronal Kv7 channel activator. Compared to retigabine, compound **60** displayed a higher brain/plasma distribution ratio, a longer elimination half-life, and more potent and effective anticonvulsant effects in an acute seizure model in mice. Collectively, these data highlight compound **60** as a promising lead compound for the development of novel Kv7 activators for the treatment of hyperexcitability diseases.

## Introduction

Epilepsy, commonly defined as a state of recurrent, spontaneous seizures occurrence, is the second most common neurological disease with more than 50 million patients diagnosed in the world.^1^ Although several details of the neurobiology of seizures are still undefined, one general principle to explain seizure occurrence is an imbalance between excitation and inhibition occurring at different levels of the nervous system: ions and membranes, cells, circuits/synapses, and large-scale neuronal networks.^2^ Currently, more than 30 antiseizure medications (ASMs) are approved for epilepsy treatment; however, approximately 30% of patients do not respond adequately to these agents, highlighting the urgent need to develop novel and safer ASMs.

Voltage-gated neuronal potassium channels (Kv channels) are critical determinants of neuronal excitability, regulating the input/output balance of individual neurons. Kv channels are among the most heterogeneous group of voltage-gated ion channels, with more than 40 genes encoding for voltage-gated potassium channel pore-forming α-subunits, which are classified into 12 subfamilies (Kv1–Kv12), and are structurally similar to a single domain of the α-subunit of voltage-gated sodium and calcium channels.^3^ Each Kv subunit is formed by six transmembrane segments (S_1_–S_6_); the voltage-sensing domain (VSD) is encompassed by S_1_–S_4_, whereas S_5_, S_6_, and the S_5_–S_6_ intervening linker form the pore region.

Among neuronal Kv channels, the Kv7 family comprises 5 different members (Kv7.1–Kv7.5), characterized by distinct tissue distribution and physiological roles. Kv7.1 is predominantly expressed in the heart, while Kv7.2–5 are most abundantly expressed in the nervous system; expression of Kv7.2 and Kv7.3 is mostly restricted to the nervous system, whereas that of Kv7.4 and Kv7.5 is more widespread, also including smooth and skeletal muscles.^4^ In both central and peripheral neurons, Kv7.2, Kv7.3, and Kv7.5 subunits underlie the M-current, a repolarizing current that limits repetitive firing and causes spike frequency adaptation.^4^ Neuronal Kv7 channels have received considerable attention in the last decades as critical targets for human epilepsy pathophysiology and therapy. In fact, on one side, mutations in KCNQ2, KCNQ3, and KCNQ5 genes encoding for Kv7.2, Kv7.3, and Kv7.5 subunits, respectively, are associated with a spectrum of seizure disorders ranging from self-limited familial neonatal epilepsy to severe epileptic and developmental encephalopathies.^5^ On the other, pharmacological activation of Kv7 channels appears as a rational approach to treat epilepsy as well as other neuropsychiatric disorders in which neuronal hyperexcitability plays a critical role, such as neuropathic pain, ischemic stroke, and amyotrophic lateral sclerosis. Several naturally occurring^6,7^ or synthetic^8^ compounds specifically acting as neuronal Kv7 channels activators have been developed. Among synthetic compounds, retigabine is the prototype Kv7 activator that has been approved for clinical use since 2011 as an add-on treatment for drug-resistant partial onset seizures with or without secondary generalization in adults. However, severe side effects associated with its long-term use, including urinary retention, blue skin discoloration, and retinal pigmentation have been recently documented,^9^ thus leading to a progressive decline in retigabine’s clinical use, followed by its market discontinuation in 2017.

Although the mechanism for the retigabine-induced tissue discoloration is still only partially understood, a long-term repeat dosing study performed to determine the distribution of retigabine and its metabolites revealed that, in the rat eye, retigabine is oxidized by light to quinone diimine, which may subsequently form dimers and, by further oxidation, phenazinium ions; the melanin binding ability of retigabine effectively concentrates the drug in melanin-rich tissues to enable mixed condensation reactions to occur.^10^ In order to reduce the side effects and, at the same time, increasing potency at specific Kv7 channel subtypes, several retigabine analogues have been synthesized. Among these, SF0034 incorporates a fluorine substituent in the 3-position of the tri-aminophenyl ring of retigabine; the introduction of an electron-withdrawing fluorine substituent on the aniline ring of retigabine is expected to improve metabolic stability. Moreover, SF0034 was also demonstrated to be five times more potent than retigabine in activating Kv7.2/Kv7.3 channels in vitro and to display anticonvulsant potency higher than that of retigabine in two mouse models of seizures.^11^ Furthermore, the introduction of a CF_3_ group at the 4-position of the benzylamine moiety of SF0034 generated the compound named RL-81, which was >15 times more potent than retigabine as a Kv7 activator in vitro.^12^ Starting from RL-81, even more potent Kv7.2/Kv7.3 channel activators have been described.^13^ Another strategy, aimed to prevent retigabine photodegradation, is the replacement of the secondary amino group of retigabine with a sulfur atom to obtain sulfide analogues.^14^ This chemical modification avoids the detrimental oxidation of the aromatic ring and shifts oxidation toward the formation of less toxic metabolites devoid of the risk of quinone formation.

In addition to chemical lability, another drawback of retigabine is its relatively low brain distribution (brain/plasma ratio of 0.16), which may require relatively high dosing, thereby reducing its safety margin for antiepileptic activity and increasing the risk for potential off-target effects. Incorporation of a propargyl group at the N4-position of retigabine generated a novel Kv7 activator, P-retigabine (P-RET), with a 20-fold improved brain distribution over retigabine; compared to retigabine, P-RET showed similar Kv7 channels subtype selectivity and potency in vitro, although it suppressed epileptic activity in vivo with 2.5 times higher potency.^15^ More recently, deleting the ortho liable -NH_2_ group and installing two adjacent methyl groups to the carbamate motif of P-RET led to the discovery of HN37 (pynegabine), a novel Kv7 activator characterized by chemical stability, improved potency, and anticonvulsant efficacy in the maximal electroshock test and in the 6 Hz model of pharmacoresistant limbic seizures.^16^

In our previous work, we have designed and synthesized a small library of conformationally restricted retigabine derivatives and characterized some of these compounds for their potency, selectivity, chemical stability, and in vitro pharmacokinetics. As a result, two compounds (**23a** and **24a**) emerged as potent Kv7.2/Kv7.3 channel activators with improved chemical stability over the parental molecule.^17^ To continue such optimization effort, we herein describe the design, synthesis, and in vitro and in vivo pharmacological evaluation of a novel series of retigabine derivatives with potential application for the treatment of epilepsy and other hyperexcitability diseases.

## Results and Discussion

### Chemistry

**Scheme 1:**
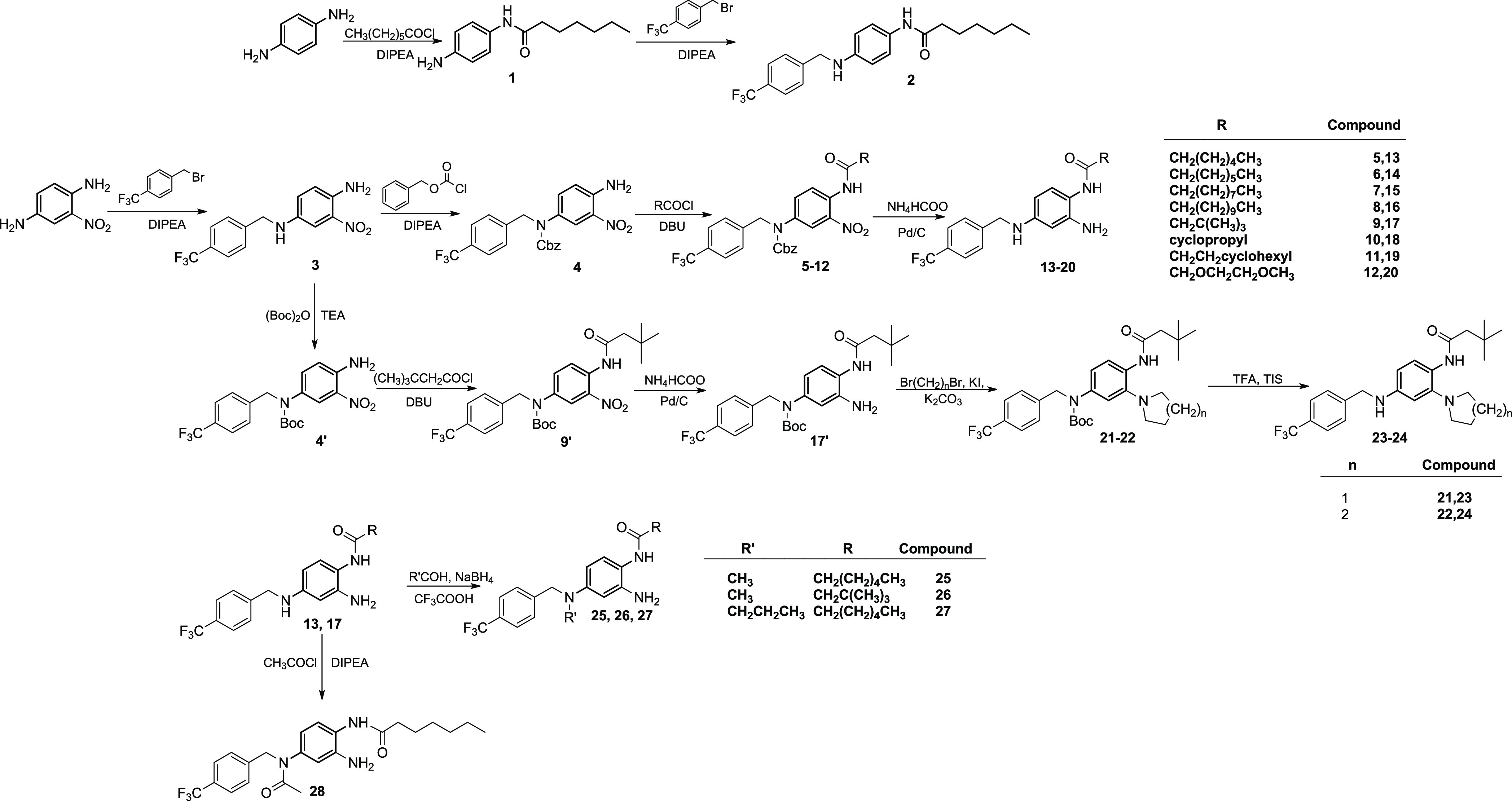
Synthesis of 1,4-Phenylenediamine (**2**), Benzene-1,2,4-triamines (**13**−**20**), 2-(pyrrolidin-1-yl)benzene-1,4-diamine (**23**), 2-(Piperidin-1-yl)benzene-1,4-diamine (**24**), *N*^4^-Alkylbenzene-1,2,4-triamine (**25**,**26**,**27**), and *N*-(3,4-Diaminophenyl)acetamide (**28**) Derivatives

The synthesis of the phenylenediamine derivative **2** was accomplished by acylation of 1,4-phenylenediamine followed by N-4 alkylation with 4-trifluoromethylbenzyl bromide (Scheme 1). The same N-4 alkylation procedure was followed to obtain intermediate **3** starting from 2-nitrobenzene-1,4-diamine. The reaction of **3** with benzylchloroformate under alkaline conditions led to the Cbz-protected intermediate **4**, which was then N-acylated using different acyl chlorides to give intermediates **5–12**. Hydrogenation of intermediates **5–12** using ammonium formate and Pd/C under reflux gave the final products **13–20** (Scheme 1). Following a different synthetic route, intermediate **3** was reacted with di-*tert*-butyl dicarbonate in alkaline media to give the N-Boc protected intermediate **4′**. The reaction of **4′** in alkaline media with 3,3-dimethylbutyryl chloride led to the N-1 acyl intermediate **9′**. Upon reduction of the nitro group of **9′** by ammonium formate and Pd/C, the corresponding amine (**17′**) was reacted with 1,4-dibromobutane or 1,5-dibromopentane under basic conditions affording intermediates **21** and **22**, respectively. Removal of the Boc protecting group from **21** and **22** with TFA/TIS led to the final products **23** and **24** (Scheme 1). Derivatives **13** and **17** were further modified at the N-4 by reductive amination reaction, leading to the N-4 alkyl derivatives **25**–**27**. Similarly, derivative **13** was reacted with acetyl chloride, leading to the N-4-acetyl derivative **28** (Scheme 1).

**Scheme 2:**
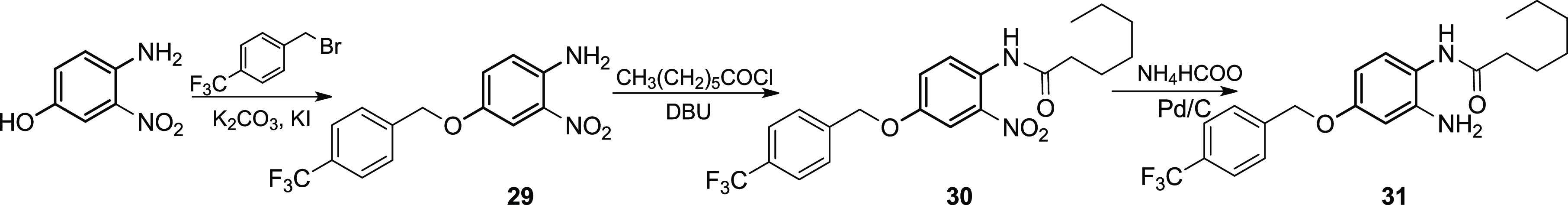
Synthesis of 3,4-Diaminophenol Derivative **31**

The 3,4-diaminophenol derivative was synthesized according to Scheme 2. 4-Amino-3-nitrophenol was converted to the corresponding ether (**29**) by reaction with 4-trifluoromethylbenzyl bromide in alkaline media. N-Acylation of **29** by heptanoyl chloride under basic conditions afforded intermediate **30.** Reduction of the nitro group of **30** by Pd/C-catalyzed hydrogenation gave final product **31** (Scheme 2).

**Scheme 3:**
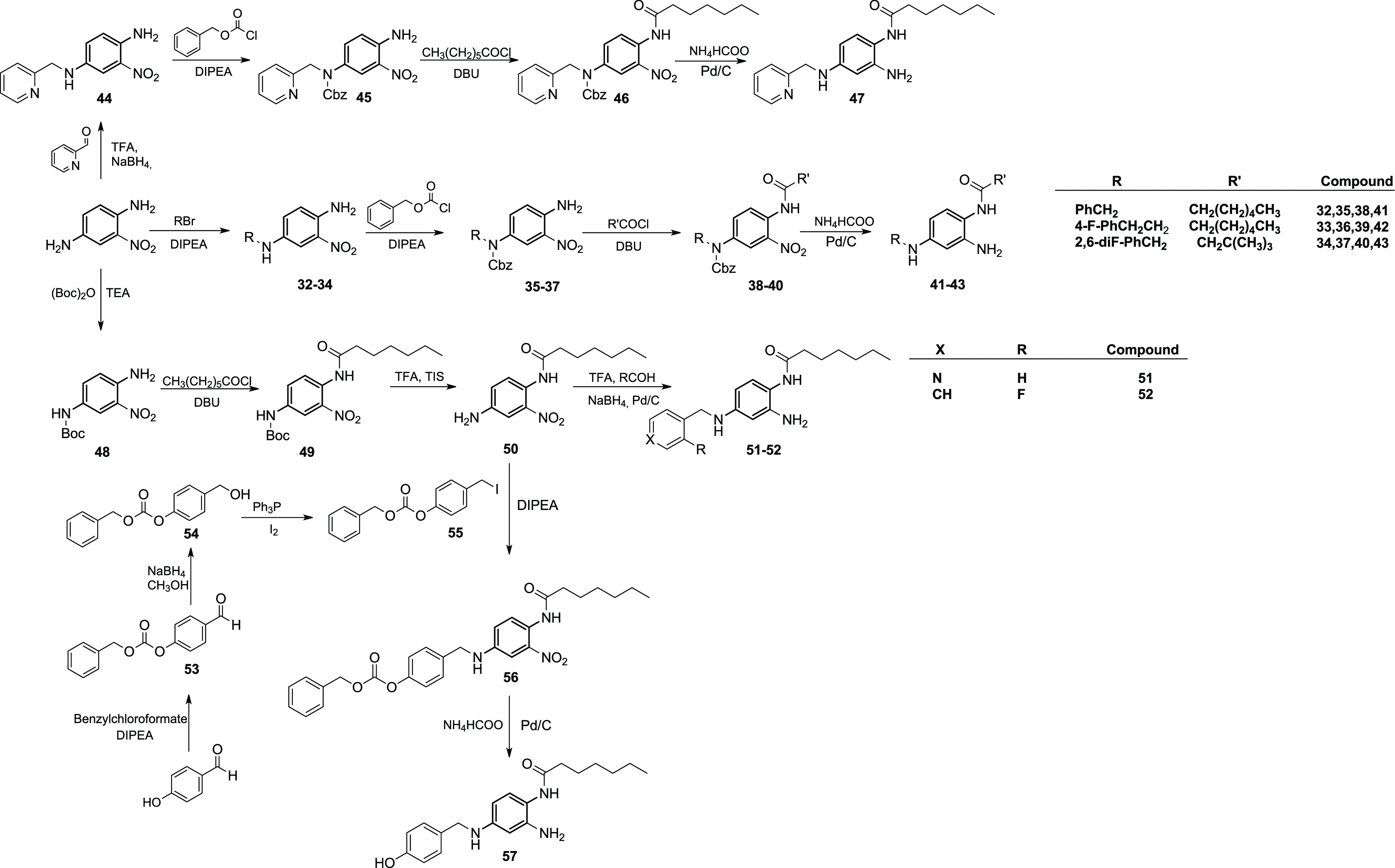
Synthesis of Benzene-1,2,4-triamine Derivatives **41–43, 47**, **51**, **52**, and **57**

To further explore the influence of the substituents in position 4 of the benzene-1,2,4-triamine scaffold, derivatives **41–43**, **47**, **51**, **52**, and **57** were synthesized (Scheme 3).

To obtain derivatives **41–43**, the 2-nitrobenzene-1,4-diamine scaffold was reacted with variously substituted benzyl halides under basic conditions to give intermediates **32–34**. N-Cbz protection of these intermediates was attained by reaction with benzylchloroformate under alkaline conditions to give derivatives **35–37**, that were acylated with heptanoyl and 3,3-dimethylbutyryl chloride affording compounds **38–39** and **40**, respectively. Catalytic hydrogenation of **38–40** over Pd/C provided the reduction of the nitro group and the removal of the Cbz protecting group leading to final derivatives **41–43**. The 4-pyridin-2-yl-methyl analogue (**47**) of derivatives **41–42** and its 4-pyridin-4-yl isomer (**51**) were synthesized using two different reaction pathways due to the peculiar reactivity of picolinaldehyde and isonicotinaldehyde used as the starting material (Scheme 3). For the synthesis of **47**, picolinaldehyde was coupled to 2-nitrobenzene-1,4-diamine by an acid-catalyzed reductive amination reaction using NaBH_4_ as the reducing agent. Intermediate **44,** thus obtained, was reacted with benzylchloroformate in alkaline conditions to give the N-protected derivative **45**, which was subjected to N-acylation using heptanoyl chloride and DBU, leading to compound **46**. Reduction of the 2-NO_2_ group and simultaneous removal of the N4-Cbz protection by Pd-catalyzed hydrogenation afforded the final compound **47** (Scheme 3). Compound **51**, indeed, was synthesized by N-4 Boc protection of 2-nitrobenzene-1,4-diamine using di-*tert*-butyl dicarbonate in alkaline media followed by acylation of the N-1 by heptanoyl chloride to give intermediate **49**. Removal of the Boc protecting group from **49** by TFA afforded N-(4-amino-2-nitrophenyl)heptanamide (**50**), which was coupled with isonicotinaldehyde under TFA-catalyzed reductive amination conditions, also leading to the reduction of the 2-NO_2_ group to the corresponding amine, providing the final product **51**. The same procedure was used for the synthesis of N-(2-amino-4-((2-fluorobenzyl)amino)phenyl)heptanamide (**52**) replacing isonicotinaldehyde with 2-fluorobenzaldehyde (Scheme 3). Intermediate **50** was also reacted in alkaline media with (4-(iodomethyl)phenyl) carbonate (**55**) that has been prepared following a previously described procedure (Scheme 3). The resulting intermediate **56** was subjected to Pd/C catalytic hydrogenation to afford the final derivative **57**.

**Scheme 4:**
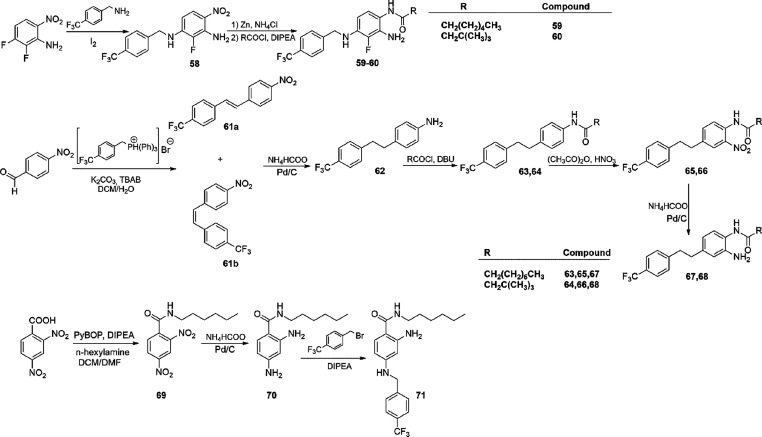
Synthesis of *N*-((2-Amino-3-fluoro-4-(benzyl)amino)phenyl)amide (**59** and **60**), *N*-(2-Amino-4-(4-(trifluoromethyl)phenethyl)phenyl)amide (**67** and **68**) Derivatives, and 2-Amino-*N*-hexyl-4-((4-(trifluoromethyl)benzyl)amino)benzamide (**71**)

*N*-(2-Amino-3-fluoro-4-(benzyl)amino)phenyl)amide (**59** and **60**) were synthesized according to Scheme 4. The Buchwald–Hartwig-type reaction of 4-trifluorobenzylamine with 2,3-difluoro-6-nitroaniline led to the intermediate **58** that was reduced using Zn and NH_4_Cl to the corresponding amine and coupled with the proper acyl chloride in a one-pot reaction affording final products **59–60** (Scheme 4). The synthesis of *N*-(2-amino-4-(4-(trifluoromethyl)phenethyl)phenyl)amide derivatives was attained starting from the Wittig-type reaction of 4-nitrobenzaldehyde with 4-trifluoromethylbenzyltriphenylphosphonium bromide, giving a mixture of *cis* and *trans* isomers of 1-nitro-4-(4-(trifluoromethyl)styryl)benzene (**61a** and **61b**). The mixture of isomers was then reduced to the corresponding 4-(4-(trifluoromethyl)phenethyl)aniline (**62**) by means of Pd/C catalytic hydrogenation. Acylation at the N-1 followed by electrophilic aromatic substitution by HNO_3_ in acetic anhydride gave the *N*-(2-nitro-4-(4-(trifluoromethyl)phenethyl)phenyl)amide intermediates **65** and **66**. Catalytic hydrogenation of these intermediates gave final products **67** and **68**. Finally, 2-amino-*N*-hexyl-4-((4-(trifluoromethyl)benzyl)amino)benzamide (**71**, Scheme 4) was synthesized by coupling of 2,4-dinitrobenzoic acid with *n*-hexylamine, using PyBOP as the coupling agent. The amide compound obtained (**69**) was reduced to the corresponding diamino derivative (**70**) by a catalytic hydrogenation procedure and then coupled with 4-trifluoromethylbenzyl bromide as previously described, to attain the final compound **71**.

### In Silico-Guided Synthesis of Novel Kv7.2 Activators

**Figure 1:**
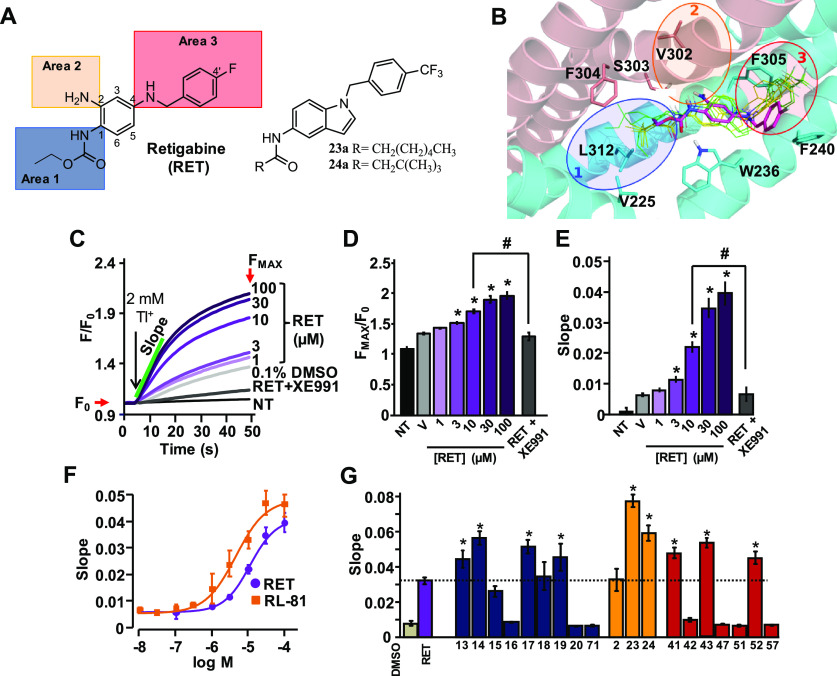
Functional characterization of a first series of retigabine derivatives (compounds **13–20, 71, 2, 23, 24, 41–43, 47, 51, 52, 57**). (A) Retigabine compounds **23a** and **24a** structures with the three different areas investigated by structure-based approach. Highlighted: area 1 in blue, area 2 in orange, and area 3 in red. (B) Retigabine binding pocket in Kv7.2 channels. The two Kv7.2 subunits shown are colored in cyan and salmon. Bound conformations of **23a** (yellow) and **24a** (green) are shown in thin solid sticks. For each ligand, three bound conformations (sampled at 0, 60, and 120 ns from 120 ns-long MD simulations of the ligand/Kv7.2 complex) are shown. Experimentally solved bound conformation of retigabine (PDB ID: 7CR2) is shown in magenta thick transparent sticks. (C) FluxOR fluorescence signals generated in Kv7.2/Kv7.3-transfected cells by the following: vehicle DMSO 0.1% (gray curve), retigabine (RET, purple curves), and retigabine + XE991 co-administrated (black curve). Non-transfected cell (NT) signal is also shown. (D,E) Average value of maximal fluorescence (*F*_MAX_/*F*_0_; D) and initial slope (E) of the FluxOR fluorescence signal calculated between 5 and 15 s. (F) Dose–response curves of RET (purple) and RL-81 (yellow). Solid lines represent fits of the experimental data to the four parameter logistic equation used to estimate EC_50_ values. (G) Average FluxOR fluorescence signals obtained in Kv7.2/Kv7.3-transfected cells upon exposure to the synthesized compounds exploring the chemical space at area 1 (blue bars), area 2 (orange bars), and area 3 (red bars) at a concentration of 10 μM in comparison with retigabine (purple bar). * and ^#^ indicate values significantly different (*p* < 0.05) from respective controls.

Early site-directed mutagenesis results, based on primary sequence differences between retigabine-sensitive (Kv7.2–5) and -insensitive (Kv7.1) channels, suggested that a tryptophan (W) residue in the pore domain (W236 in Kv7.2, W265 in Kv7.3) is critical for retigabine binding.^18,19^ Further elegant analysis using non-natural isosteric H-bond-deficient W analogues revealed that this W residue acts as a hydrogen bond donor (HBD) with the carbamate group of retigabine acting as a hydrogen bond acceptor (HBA).^20^ More recently, the introduction of larger substituents such as an heptyl group or a 2,2-dimethylbuthyl group at retigabine’s carbamate region of a series of conformationally restricted analogues (compounds **23a** and **24a**, respectively; Figure 1A) caused a marked increase in potency, allowing to hypothesize the existence of a large and plastic binding pocket accommodating larger substituents. As suggested by homology modeling studies, this pocket was lined by residues L221, V225, L232, F304, L307, I311, and L312.^17^ The observation that the Kv7 opening actions of **23a** and **24a**, similar to retigabine, were almost completely abolished upon substitution of the W residue at 236 with a non-H-bond-forming l residue confirmed that these analogues maintained the general binding orientation of the parental molecule. To identify additional specific protein/ligand contacts, which might be responsible for the improved activity of **23a** and **24a** over retigabine, molecular docking and molecular dynamics (MD) experiments were performed using the recently described cryoEM structure of Kv7.2 in complex with retigabine.^21^ The results from these experiments revealed that the larger substituents of both **23a** and **24a** specifically interact with residues V225, F304, and L312 lining a region defined as pocket 1; these interactions are less pronounced in retigabine, given the smaller size of its amide carbonyl substituent (Figure 1B; Figure S94A–C). In addition, MD simulations gave insight into the interaction of retigabine, **23a**, and **24a** with two additional regions in Kv7.2, one contributed by S303 (pocket 2, Figure 1B; Figure S94A–C, Table S2) forming a H-bond with the NH_2_ at position 2 of retigabine (area 2 in Figure 1A) and flanking a small hydrophobic pocket lined by T276, L299, V302, S303, F305, and A306 and another formed by F240, L243, L268, L272, L275, and F305 (pocket 3 in Figure 1B, Figure S94, Table S2), where F305 and F240 may interact with the fluorophenyl ring of retigabine (area 3 in Figure 1A).

These three pockets identified in Kv7.2 (Figure 1B) also occur in other retigabine-sensitive Kv7 subunits, as revealed by the recent cryoEM structure of Kv7.4 in complex with retigabine.^22^ Moreover, the high primary sequence similarity among retigabine-sensitive subunits at the level of the residues contributing to these pockets also suggests that the indicated interactions may also occur in Kv7.3 and Kv7.5 subunits (Figure S93).

To verify the in silico hypothesis and to identify optimized agonists, chemical modifications in each of the three areas of retigabine (Figure 1A) were pursued, and a first library of retigabine derivatives was synthesized.

### Functional Evaluation of New Retigabine Analogues as Kv7.2/Kv7.3 Openers

To evaluate the Kv-7 opening activity of the newly synthesized retigabine derivatives, a fluorescence-based medium-throughput assay was implemented. To this aim, a commercially available assay based on the thallium-sensitive fluorescent dye FluxOR was performed in mammalian CHO cells stably expressing Kv7.2 alone or Kv7.2 + Kv7.3 subunits.^23−25^ When CHO cells expressing Kv7.2 + Kv7.3 were preincubated with the fluorescent dye and then exposed to thallium (Tl^+^) ions, retigabine (1–100 μM) dose-dependently increased the maximal fluorescence (Figures 1C,D) and the initial slope of the fluorescence signal (Figure 1E); both effects were abolished by 10 μM of the Kv7 blocker XE991.^26^ Retigabine-induced changes in maximal fluorescence intensity were much smaller than those in the slope of the fluorescence signal; using the latter parameter the EC_50_ for retigabine was 11.2 ± 1.6 μM, whereas it was about 3-times lower for RL-81 (4.0 ± 1.0 μM, Figure 1F). While assay robustness in Kv7.2/Kv7.3-expressing cells was high (*Z*′ factor > 0.5),^27^ the *Z*′ factor calculated in CHO cells expressing Kv7.2 subunits alone was <0.5; thus, all subsequent pharmacological screens were performed in Kv7.2/Kv7.3-expressing cells. Having determined the robustness of the fluorescent assay and its ability to detect potency differences between known Kv7 activators, the changes in the initial slope of the fluorescence signal produced by 10 μM of each newly synthesized retigabine analogue were compared to that of 10 μM retigabine (or other compounds used as reference for each subseries). The overall results from these experiments, which will be discussed referring to the previously mentioned retigabine areas (1, 2, and 3), are shown in Figure 1G.

**Figure 2:**
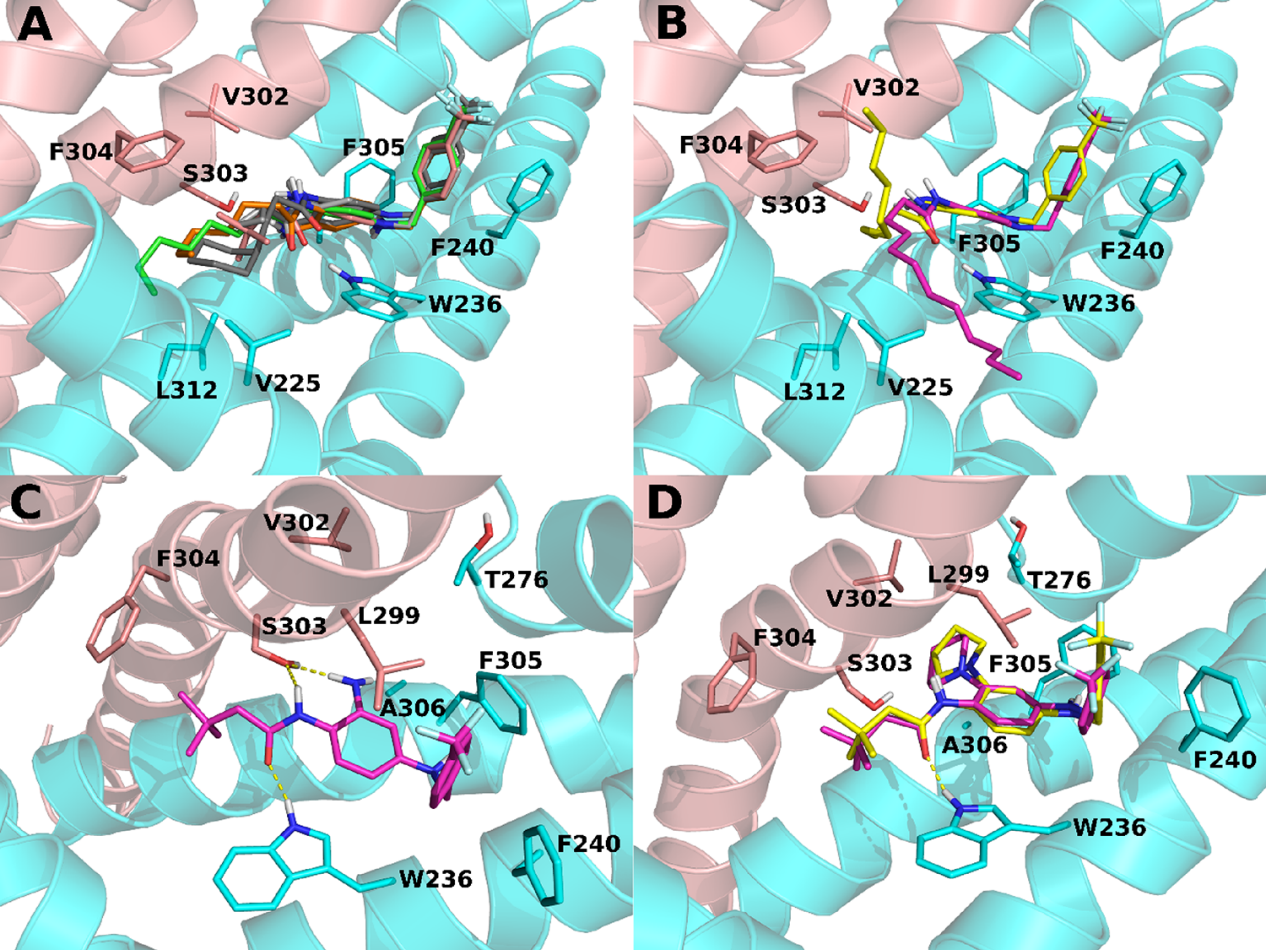
Molecular dynamics (MD) simulations of retigabine analogues. (A) Predicted bound conformations of **13** (orange), **14** (green), **17** (pink), **18** (light gray), and **19** (dark gray) at 60 ns of MD simulations are depicted in sticks. (B) Bound conformations of **15** (yellow) and **16** (magenta) at 60 ns of MD simulations are depicted in sticks. (C) Predicted bound conformation of **17** (magenta sticks) at 60 ns of MD simulations. (D) Predicted bound conformations of **23** (yellow sticks) and **24** (magenta sticks). In all panels, the two different Kv7.2 monomers are depicted in sticks and cartoons and colored in cyan and salmon. H-bonds are represented by yellow dashed lines.

Exploration of the lipophilic pocket 1 (Figure 1B) was performed with compounds indicated as **13–20** and **71** carrying substitutions at R1 in area 1 (Figure 1G, blue bars; Table 1). For this subseries, compound **13** was considered as the reference compound, since a 4-(trifluoromethyl)benzyl group at R3 responsible for a marked improvement in agonist activity (i.e., RL-81)^17,12^ was present in all these derivatives. The results obtained confirm the presence of a lipophilic pocket in which linear (compounds **13**, **14**), branched (compound **17**), or cyclic (compounds **18**, **19**) substituents are well accommodated when up to 7 carbon atoms are present at R1 (Table 1, Figure 2A). Instead, at least for linear chains, beyond this optimal length, a progressive decrease in Kv7 opening ability was observed (compounds **15** and **16**); consistent with this are the results of MD simulations showing the escape from the binding pocket of the longer side chains of compounds **15** and **16** (Figure 2B). In addition, given the hydrophobic nature of pocket 1, hydrophilic substituents at R1 are poorly tolerated, as for the ethylene glycol chain of compound **20** showing a complete loss of activity. As previously introduced, H-bonding between the carbamate group of retigabine and the indole nitrogen atom of the W236 residue is critical for Kv7 opening activity.^20^ The observation that the inversion of the amide group as in derivative **71** leads to a complete loss of activity confirmed the constraints imposed by the specific orientation of the hydrogen bond donor (HBD)–hydrogen bond acceptor (HBA) pattern at W236 for the Kv7 opening.

**Table 1:**
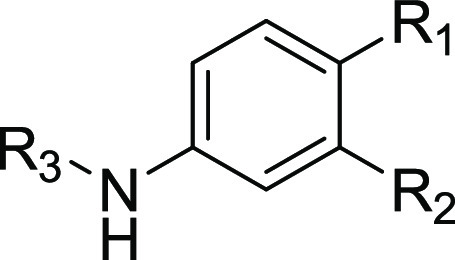

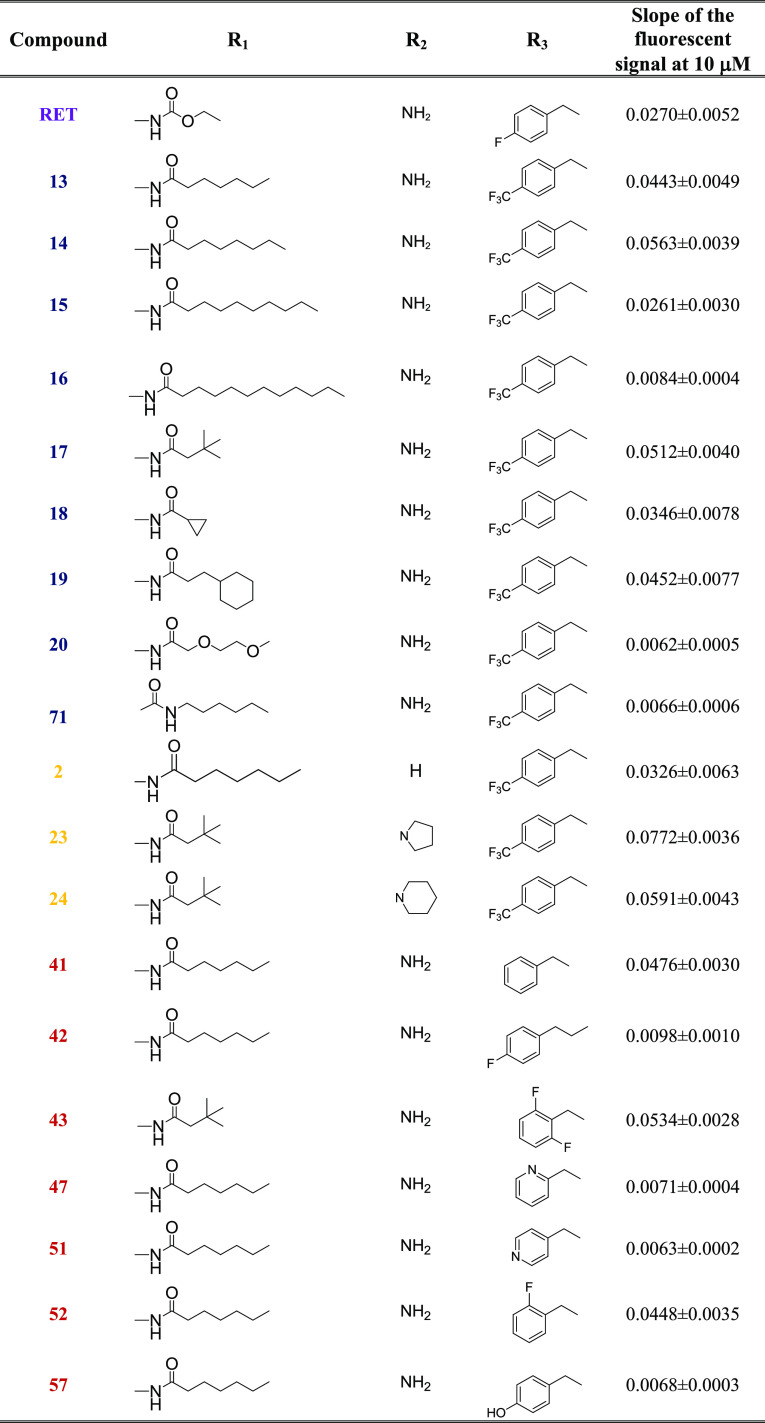
Chemical Structures and Kv-7 Opening Activity of Synthesized Compounds

CryoEM studies in Kv7.2 channels have recently revealed that the amine group at position 2 in retigabine within area 2 establishes an H-bond with the S303 side chain.^21^ However, Kv7.2 channels in which S303 was substituted with an alanine residue lacking the H-bonding side-chain hydroxyl group were still sensitive to activation by retigabine although to a reduced extent.^21^ Noteworthily, HN37 (pynegabine)^16^ in which the NH_2_ group at position 2 of retigabine was replaced with a methyl group also unable to act as a HBD was even more potent than retigabine or its analogue P-RET,^15^ suggesting that this H-bond interaction is not essential for Kv7 opening ability. Consistent with this hypothesis, compound **2** of our series, in which the NH_2_ is replaced by a hydrogen atom, was still active although with slightly lower efficacy when compared to the structurally similar compound **13** (Figure 1G). Moreover, replacement of the NH_2_ with larger substituents unable to act as HBDs such as pyrrolidin-1-yl and piperidin-1-yl groups (compounds **23** and **24**, respectively) resulted in a Kv7 opening ability comparable to that of the reference compound **17**, with **23** being even more active (Figure 1G). Altogether these results suggest that lipophilic interactions can occur within pocket 2; molecular modeling studies suggested that residues T276, L299, V302, S303, F305, and A306 might act as possible contributors to such interactions (Figure 2C,D). Noteworthily, hydrophobic interactions involving the methyl group at position 2 may explain the increased potency shown by HN37 when compared to P-RET.^16^

Within area 3, the Kv7.2/retigabine cryoEM structure suggests that two phenylalanines (F305 and F240) are close enough to π–π stack with the retigabine benzyl ring,^21^ a result confirmed by the present MD simulations. By contrast, opposite to recent suggestions,^28^ no direct contact between the retigabine fluorine atom and the carbonyl oxygen of the protein backbone at the A265 residue^28^ was found in our simulation. Differences in the modeling templates (the open state of KCNA2/Kv1.2 chimera^28^ and the activated Kv7.2 cryoEM structure in our simulations) provide plausible explanations for these diverging results.

In an attempt to probe the interactions of the terminal phenyl ring of retigabine with pocket 3, a series of analogue carrying modifications at R3 in area 3 were synthesized (Table 1) and tested (Figure 1G; red bars). Moving the fluorine atom in position 2 of the phenyl ring (compound **52**) or its removal (compound **41**) resulted in no change in activity when compared to reference compound **13**, thus ruling out any specific halogen bond involving this fluorine atom; in addition, the 2,6-difluoro analogue of compound **17** (compound **43**) designed to help the ligand phenyl ring to assume an optimal orientation for edge-to-face and/or face-to-face interactions with phenylalanines 240 and 304 still displayed strong activity. Altogether, these results confirm the critical functional role of the previously mentioned π–π stacking interactions for Kv7 opening. Moreover, replacement of the fluorobenzyl group with hydrophilic hydroxybenzyl (compound **57**) or pyridine (compounds **47** and **51**) groups led to a complete loss of activity, despite their ability to form π–π stacking interactions; these results suggest that a critical degree of hydrophobicity at this region is required for Kv7 opening. Finally, increasing the length of the linker between the fluorobenzyl ring and the amino group at N4 of retigabine with an extra CH_2_ led to a complete loss of activity (compound **42**), likely because this substitution impedes the interaction with pocket 3 residues L272 and F305 (Figure S95, Table S3).

### Synthesis of a Second Series of Retigabine Derivatives with Improved Photostability

**Figure 3:**
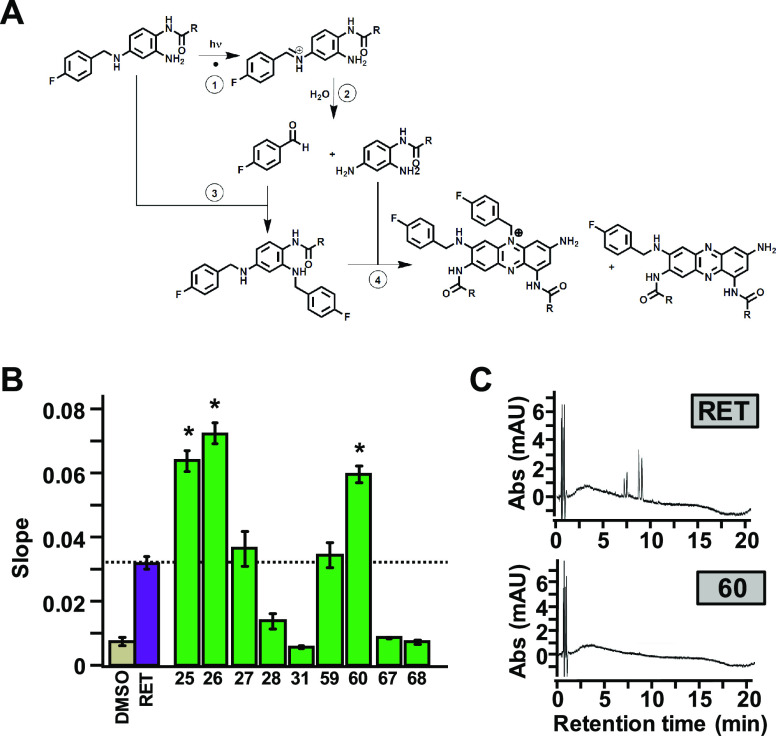
Functional characterization of a second series of retigabine derivatives with improved photostability (compound **25–28**, **31**, **59**, **60**, **67**, **68**). (A) Proposed mechanism for retigabine photo-oxidation. (B) Average FluxOR fluorescence signals obtained in Kv7.2/Kv7.3-transfected cells upon exposure to the indicated photostable compounds, each used at a concentration of 10 μM in comparison with retigabine (RET 10 μM; purple bar). * indicates values significantly different (*p* < 0.05) from respective control. (C) HPLC traces of retigabine (RET) and compound **60** at 550 nm after 3 h exposure to UV–visible light.

The previously described structure-based exploration of the retigabine binding site led to the identification of several retigabine analogues active as Kv7.2/Kv7.3 agonists. Nevertheless, their photostability was unknown as was their lability for photo-induced dimers formation.^29,10^ We previously hypothesized^17^ that the first step of retigabine photo-oxidation is the cleavage of the C–N bond^30,31^ in the linker between the two phenyl groups, leading to the formation of 4-fluorobenzaldehyde and ethyl (2,4-diaminophenyl)carbamate (reactions 1 and 2; Figure 3A). Notably, ethyl (2,4-diaminophenyl)carbamate has been consistently detected as one of the four process-related impurities in several batches of retigabine;^32^ moreover, 4-fluorobenzaldehyde is formed upon UV–visible light irradiation of retigabine solution.^17^ Reaction of the aldehyde intermediate with an intact retigabine molecule leads to the formation of ethyl (2,4-bis((4-fluorobenzyl)amino)phenyl)carbamate (reaction 3; Figure 3A);^17^ further reaction of the ethyl (2,4-bis((4-fluorobenzyl)amino)phenyl)carbamate with ethyl (2,4-diaminophenyl)carbamate, most likely in the imino tautomeric form, drives the formation of phenazine and phenazinium dimers (reaction 4; Figure 3A), such as those detected in melanin-rich eye tissues upon long-term treatment with retigabine.^10^

In order to assess the photostability and dimer-forming ability of the most effective Kv7 agonists described in the previous section, compounds **13**, **14**, **17**, **19**, **23**, **24**, **41**, **43**, and **52**, each dissolved in a saline solution at 10 μM, were exposed for 3 h to UV–visible light followed by HPLC analysis of the reaction products. Two different HPLC wavelengths were utilized: (a) 220 nm to evaluate the decreased concentration of the starting molecule; (b) 550 nm to investigate the formation of phenazine/phenazinium dimers, as previously described.^10^ Unfortunately, dimer formation was detected for all tested compounds, except for **23** and **24**. However, these two compounds showed an enhanced degradation when compared to retigabine (Table S1). The inability of compounds **23** and **24** to form dimers is likely due to the lack of the free amino group in position 2 required for reaction 4 to occur (Figure 3A). Thus, to minimize dimer formation and, at the same time, retain the optimal pharmacological activity revealed by previously described structure–activity relationship studies, three additional groups of retigabine analogues were designed, synthesized (Table 2), and tested (Figure 3B). The first group consisted of N4 (−X- in Table 2) substituted analogues, in which the tertiary amine is unavailable for the formation of phenazine dimers (compounds **25–28**). Small lipophilic substituents at N4, such as the methyl groups of **25** and **26**, improved agonist activity, whereas longer lipophilic substituents, such as a propyl group of **27** did not improve activity; finally, rigid substituents, such as the acetyl group of **28**, markedly reduced activity. These observations suggest that pocket 3 displays a limited degree of plasticity, accommodating only small lipophilic substituents. In accordance with this hypothesis, P-RET carrying a propargyl group (whose size is similar to that of the propyl group present in compound **27**) at position N4 does not show an improved activity over retigabine as a Kv7.2/Kv7.3 channel activator.^15^ The second group of molecules designed to prevent C–N bond photo-oxidative cleavage, which included isosteric replacements of the NH in position 4 with oxygen (**31**) or methylene groups (**67–68**), failed to activate Kv7.2/ Kv7.3 channels. The third group included derivatives replacing hydrogen atoms with electron-withdrawing fluorine atoms at position R2 of the benzene-1,2,4-triamine core scaffold (compounds **59, 60**), a strategy likely reducing the reactivity of N2 and N4. This latter approach has been profitably used before to develop potent and metabolically stable Kv7.2 activators such as RL-81.^12^ Within this series, when compared to retigabine, Kv7 opening activity was similar for **59** and enhanced for **60**.

**Table 2:**
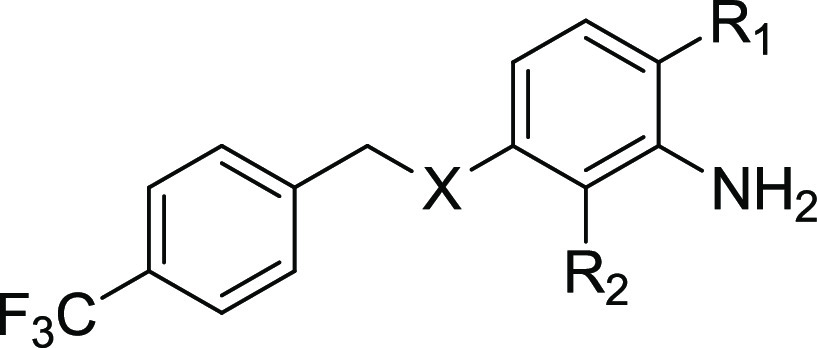

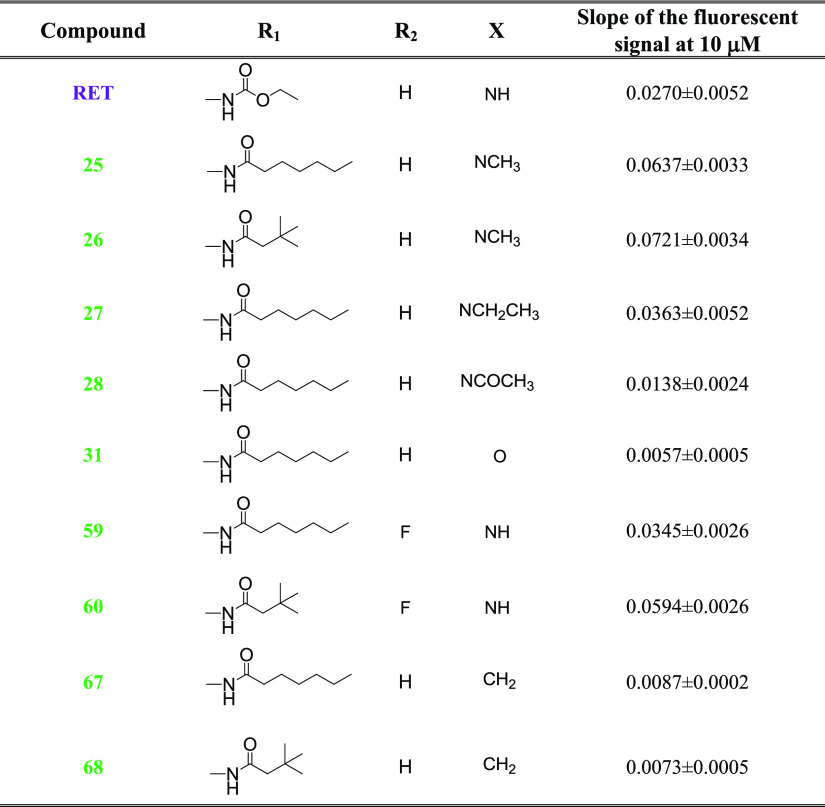
Chemical Structures and Kv-7 Opening Activity of Photostable Retigabine Derivatives.

Overall, within this novel series of molecules, three compounds (**25**, **26**, **60**) displayed efficacy as Kv7.2/Kv7.3 channel activators higher than that of retigabine. Intriguingly, while compounds **25** and **26** did not form dimers but underwent extensive photodegradation (Table S1), compound **60** was both more photostable than retigabine and failed to dimerize, as indicated by the absence of the peaks at 550 nm in the HPLC spectrum (Figure 3C). These results are consistent with the proposed mechanism for retigabine photodegradation and dimer formation shown in Figure 3A. In fact, the tertiary amine in position 4 of **25** and **26**, although preventing phenazine dimer occurrence, remained prone to C-N photo-oxidative cleavage. The reduced electron availability at N2 and N4 due to the presence of fluorine atom in position 3 of compound **60** strongly reduces also the first photo-oxidative step, thus conferring remarkable photostability. The compound also showed chemical stability when solubilized in the same aqueous buffer for 3 h avoiding UV light exposure (Figure S96).

To further explore the metabolic stability of compounds **25**, **26**, and **60** in a biologically relevant model, they were tested in an in vitro metabolism assay (S9 fraction of human liver microsomes) using retigabine as a comparator.^33,34^ In this assay, a very small extent (4.7 ± 0.5%) of retigabine undergoes phase I metabolism, whereas a larger fraction (17.4 ± 1.2%) was metabolized in phase II reactions, as previously reported.^35^ Such a metabolic profile largely overlaps that of compound **60**, whereas larger fractions of both compounds **25** and **26** underwent in vitro metabolism via both pathways; indeed, compounds **60**, **25** and **26** showed a phase I turnover metabolism of 8.2 ± 2.7%, 20.6 ± 1.4% and 32.1 ± 1.2, respectively, and a phase II turnover metabolism of 15.6 ± 0.3, 33.2 ± 1.0% and 32.7 ± 0.3, respectively.

### Electrophysiological Assessment of Compound **60** as the Kv7 Opener: Comparison with Retigabine and RL-81

**Figure 4:**
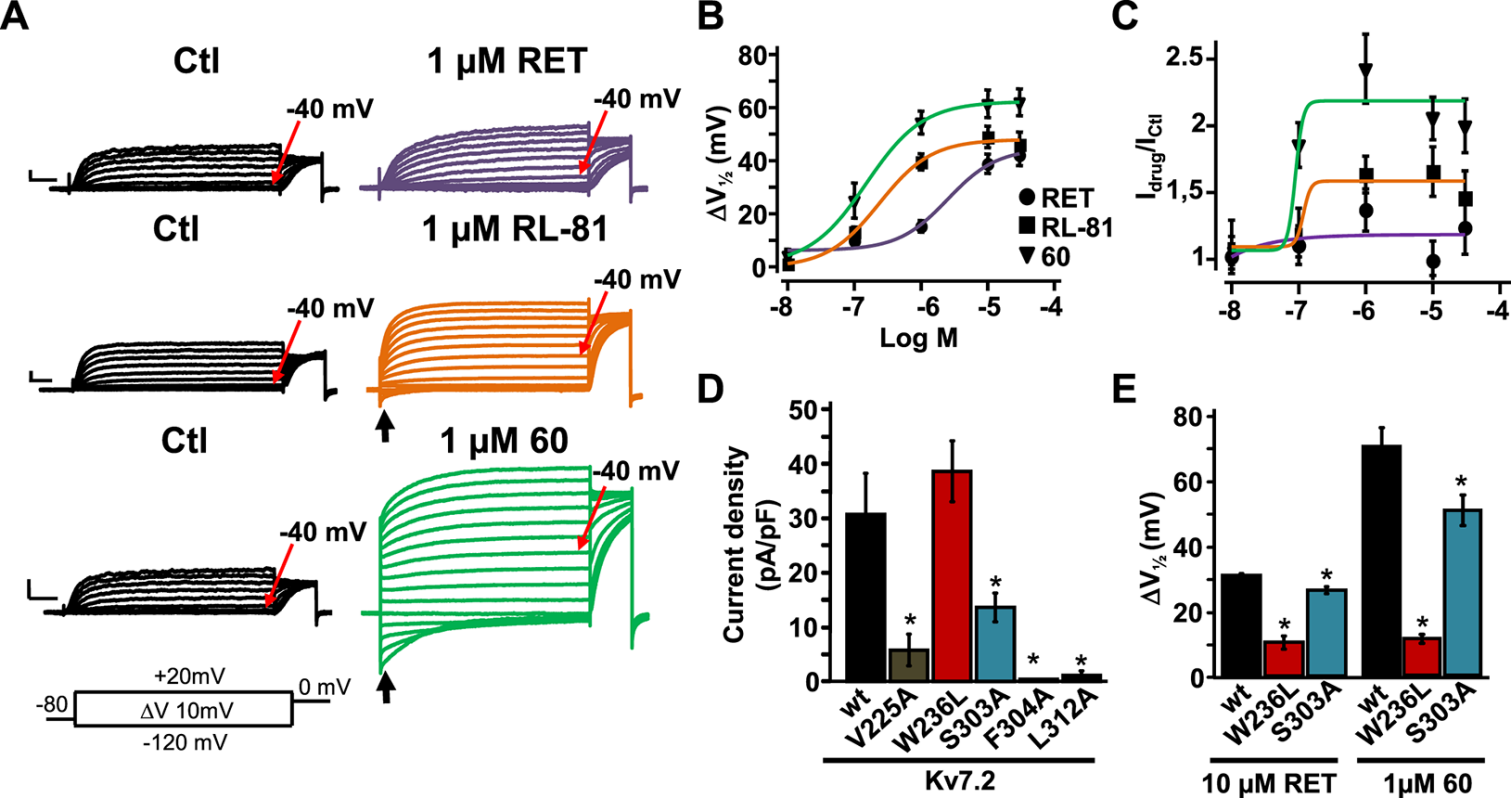
Effect of retigabine (RET), RL-81, and compound **60** on Kv7.2/Kv7.3 currents. (A) Representative macroscopic current traces recorded from CHO cell expressing Kv7.2/Kv7.3 channels in response to the indicated voltage protocol before (left) and after (right) application of 1 μM RET, RL-81 and compound **60**, as indicated. Current scale, 200 pA; time scale, 200 ms. (B,C) Dose–response curves reporting the effects of the 3 indicated compounds on the *V*_1/2_ shift (Δ*V*_1/2_) in mV (B) and on maximal current density (*I*_drug_/*I*_control_; C) calculated at +20 mV for Kv7.2/Kv7.3 channels. (D,E) Current density of the indicated homomeric Kv7.2 channels (D) and the effect of 10 μM RET or 1 μM **60** on the indicated homomeric Kv7.2 mutant channels. * indicates values significantly different (*p* < 0.05) from respective controls.

Given the marked photostability and in vitro metabolic profile of compound **60** and considering its higher efficacy as a Kv7.2/Kv7.3 activator when compared to retigabine in the FluxOR assay, a comparative assessment among retigabine, RL-81,^12^ and compound **60** was carried out using the whole-cell patch-clamp electrophysiological technique, the gold-standard assay for a detailed evaluation of ion channel modulators. Kv7.2/ Kv7.3 channels expressed in CHO cells generated voltage-dependent K^+^-selective currents characterized by a slow time course of activation and deactivation, a threshold for current activation around −40 mV (Figure 4A), and a half activation potential (*V*_1/2_) of −30.2 ± 0.7 mV. Perfusion with 1 μM retigabine induced a leftward shift in *V*_1/2_ (Δ*V*_1/2_) of about 10 mV; the same concentration of RL-81 or compound 60 caused a leftward shift of about 40 and 50 mV, respectively (Figure 4A). The negative shift in the activation voltage triggered by RL-81 and, more so, compound **60** in Kv7.2/Kv7.3 channels caused a significant fraction of channels to be open at the holding voltage of −80 mV; most of those open channels were closed upon membrane hyperpolarization to −120 mV, leading to the appearance of deactivating inward currents (arrows in Figure 4A).

To better assess the quantitative differences occurring in Kv7-opening ability between retigabine, RL-81, and compound **60**, dose–response experiments (0.01–30 μM) were performed to calculate EC_50s_ using both functional parameters of Δ*V*_1/2_ (Figure 4B) and maximal current increase (Figure 4C). Δ*V*_1/2_ EC_50_ were 2.5 ± 1.8, 0.24 ± 0.06, and 0.15 ± 0.03 μM for retigabine, RL-81, and compound **60** (*p* < 0.05 RL-81 and **60** vs retigabine, *n* = 5), respectively. Instead, it was not possible to define an EC_50_ for retigabine when the maximal current was taken into consideration, given the small size of the drug-induced effect, as previously reported;^21^ in fact, the *I*_retigabine_/*I*_control_ was 0.99 ± 0.15. The same experiments carried out with RL-81 (in which the *I*_drug_/*I*_control_ was 1.6 ± 0.2) and compound **60** (whose *I*_drug_/*I*_control_ was 2.1 ± 0.3**)** revealed EC_50s_ of 0.27 ± 0.04 and 0.06 ± 0.01 μM (p < 0.05, n = 6–11), respectively. Altogether, these data, while confirming the 10-fold higher potency of RL-81 over retigabine as the Kv7.2/Kv7.3 activator,^12^ also revealed that compound **60** was 16 times more potent than retigabine, thus resulting about twice more potent than RL-81. Such rank-order of potency is similar to that revealed by the Tl^+^-based fluorescent assay, although the absolute EC_50_ values calculated with electrophysiological methods appear generally lower than those assessed with the fluorescence assay; indeed, the EC_50_ values were 11.2 ± 1.6 μM for retigabine, 4.0 ± 1.0 μM for RL-81 (*p* < 0.05 vs retigabine, *n* = 5), and 3.2 ± 1.7 μM for compound **60** (*p* < 0.05 vs retigabine, *n* = 5).

When compared to most previously described retigabine analogues such as SF0034, RL-81, P-RET, and NS15370, which only modestly enhanced the maximal currents,^11,12,15,36^ the marked increase in current size at depolarized potentials observed with compound **60** is suggestive of a slightly different mechanism of channel activation by this drug. Thus, experiments were carried out to identify the molecular basis for such a unique mechanism. Noticeably, similar to retigabine, the Kv7 opening ability of compound **60** was almost fully abolished in W236L channels and slightly but significantly reduced in S303A channels (Figure 4D,E), suggesting a marked similarity in the overall binding of compound **60** and retigabine. Unfortunately, we could not test whether, as predicted by our molecular modelling studies, the slight increase in potency and the markedly higher efficacy as the Kv7 activator shown by compound **60** over retigabine or RL-81 was also due to its ability to establish additional and specific hydrophobic interactions with residues in pocket 1; in fact, Kv7.2 V225A, F304A, and L312A mutant channels carried currents whose size was too low to be amenable for pharmacological analysis (Figure 4D). Noteworthily, compound **60** and RL-81 only differ in the size and hydrophobicity of their substituents at position R1, strongly suggesting that the small but significant potency and efficacy difference as Kv7.2/Kv7.3 activators existing between these molecules can be only attributed to structural difference at this position. Moreover, the fact that the increased hydrophobicity in area 3 due to the incorporation of a propargyl group at position N4 in P-RET failed to increase potency and efficacy over retigabine^15^ also highlights the critical functional role of area 1 substitutions.

In order to evaluate the effects of compound **60** on other Kv7 channels, its ability to activate Kv7.4 channels expressed in CHO cells was also investigated. Application of compound **60** at a concentration corresponding to the EC_50_ for Kv7.2/Kv7.3 channels (0.1 μM), caused a Δ*V*_1/2_ of −29.2 ± 4.4 mV and *I*_drug_/*I*_control_ of 2.5 ± 0.3 (*n* = 4) on Kv7.4 currents; both these values were not significantly different (*p* > 0.05) from those observed in Kv7.2/Kv7.3 channels (Figure 4B,C, respectively). These results suggest that, similar to retigabine, compound **60** does not discriminate between Kv7.2/Kv7.3 and Kv7.4 channels, a result consistent with the high degree of conservation of the amino acids involved in retigabine binding between Kv7.2^21^ and Kv7.4 channels^22^ (Figure S93), as well as with the structural similarity of pocket 1 likely accommodating the R1 substituents responsible for the higher potency of compound **60** as a Kv7 activator. As a matter of fact, other retigabine analogues substituted within this region exert similar effects in both Kv7.2/Kv7.3 and Kv7.4 channels.^36^

### Anticonvulsant Effects of Compound **60** in a Mouse Model of Acute Seizures

Overall, the data reported suggest that compound **60** is a chemically stable, highly potent Kv7.2/Kv7.3 channel activator; given that activation of the Kv7.2/Kv7.3 channels exerts antiseizure effects in vivo,^37^ the possible anticonvulsant activity of compound **60** was evaluated in an acute seizure model and compared to that of retigabine. To this aim, a widely used mouse model of generalized myoclonic seizures such as the acute exposure to the GABA_A_ receptor antagonist pentylenetetrazol (PTZ) was chosen; in this model, Kv7.2/Kv7.3 activation induced by retigabine,^32^ HN37,^16^ ICA27243,^38^ or LuyAA41178^39^ exerts antiseizure effects.

**Figure 5:**
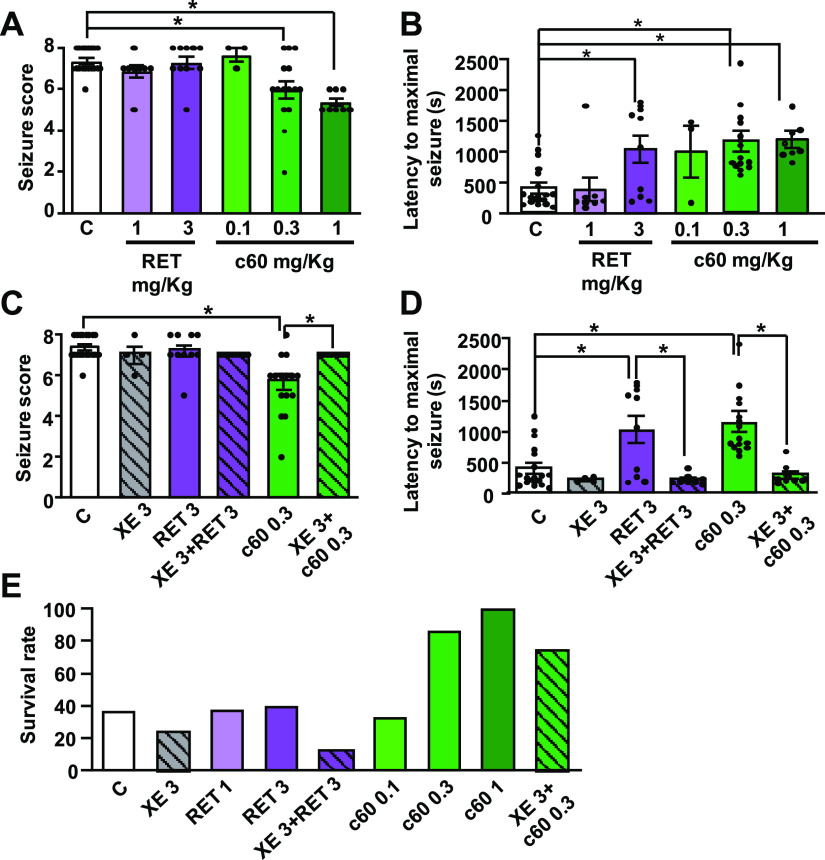
Anticonvulsant efficacy of retigabine and compound **60** (**c60**) in pentylenetetrazol (PTZ)-induced acute seizures in mice. (A,B) Average values for the seizure score (A) and latency to onset of a maximal seizure (B) as a function of retigabine (RET) or compound **60** doses. Individual scores calculated in each animal are indicated by dots. (C,D) Effect of retigabine (RET) and compound **60**, with (hatched bars) or without (empty bars) pretreatment with XE991 (XE 3 mg/kg) on seizure score (C) and latency to onset of a maximal seizure (D). (E) Effect of RET (1, 3 mg/kg), **c60** (0.3–1 mg/kg) or XE991 (3 mg/kg) on mice survival rate after PTZ exposure. The number of animals used in each group was: 19 for controls, 4 for XE (3 mg/kg), 8 for RET (1 mg/kg), 10 for RET (3 mg/kg), 8 for XE + RET (3 + 1 mg/kg); 3 for **c60** (0.1 mg/kg), 15 for **c60** (0.3 mg/kg), 8 for **c60** (1 mg/kg), and 8 for XE + **c60** (3 + 0.1 mg/kg). The asterisk (*) indicates values significantly different (*p* < 0.05) from respective controls.

A subcutaneous (s.c.) injection of 100 mg/kg PTZ in mice is able to trigger convulsive behavior whose intensity can be assessed and quantified according to the revised Lüttjohann’s scale (see Materials and Methods),^40^ using a 9-point severity score ranging from 0 (whisker trembling) to 8 (wild jumping). For each animal, the maximal severity score (Figure 5A) and the time latency required to reach such values (Figure 5B) were recorded. To assess the antiseizure effects of retigabine and compound **60**, each mouse was pretreated with retigabine (1 or 3 mg/kg i.p.) or compound **60** (0.1, 0.3, or 1 mg/kg i.p.) 30 min before PTZ injection. Vehicle-treated mice gave a seizure score of 7.4 ± 0.1 (Figure 5A) and a latency to maximal seizures of 407.6 ± 89.3 s (Figure 5B). Retigabine failed to affect seizure severity (seizure score of 6.9 ± 0.3 and 7.3 ± 0.3 at 1 and 3 mg/kg, respectively, Figure 5A), whereas it significantly increased the latency to maximal seizure(s) when used at 3 mg/kg (1041.4 ± 223.2 s, Figure 5B), in full agreement with literature data.^41^ By contrast, compound **60** was able to reduce both the severity and the latency of PTZ-induced seizures when used at doses 10 times lower than those of retigabine (seizure score of 6.0 ± 0.4 and latency time of 1159.7 ± 169.4 s at 0.3 mg/kg dose, Figure 5A,B, respectively). The antiseizure effects of retigabine and compound **60** appeared to be largely mediated by their Kv7 channel-opening actions, as revealed by the ability of the selective channel blocker XE991 (3 mg/kg i.p.), to fully prevent their antiseizure effects; a dose of 3 mg/kg i.p. of XE991 was chosen since this was previously shown to be effective in reverting the antiseizure^42^ or neuroprotective^43^ actions of retigabine in vivo. It should be highlighted that, in addition to its Kv7 opening actions, retigabine may act as a GABA_A_ agonist, although this effect only occurs at concentrations higher than those required to activate Kv7 channels (namely, ≥10 μM).^44,45^ In the present work, we did not evaluate a possible direct effect of compound **60** on GABA_A_ receptors, but our in vivo results showing the ability of XE991 to fully revert the antiepileptic effect of **60** both on the seizure score and latency to maximal seizures strongly suggest that Kv7 channels play a major role in the anticonvulsant effect of **60**. As shown in Figure 5C,D, XE991 alone did not affect the seizure severity score or latency. In these experiments, the doses used for retigabine or compound **60** were 3 and 0.3 mg/kg, respectively, representing the minimum effective doses calculated from previous experiments. In several acute seizure animal models, including the PTZ model herein investigated, high mortality rates are observed.^46^ In our experiments, only about 37% (7/19) of vehicle-treated mice survived at the end of the 60 min observation period (Figure 5E). In agreement with its strong antiseizure effect, compound **60** dose-dependently reduced mortality, with 87% (13/15) animals treated with 0.3 mg/kg and all animals (8/8) treated with the highest dose of 1 mg/kg surviving; instead, no protective effect on mortality was observed with the highest dose of retigabine (3 mg/kg), with only 40% (4/10) of mice surviving. Notably, compound **60**-induced pro-survival effects were largely (though not fully) abolished by XE991 pretreatment (Figure 5E), with 75% (6/8) of mice surviving after PTZ administration. As reported,^47^ doses higher than 3 mg/kg XE991 led to the occurrence of significant tremors, which may affect the behavioral observation of the epileptic phenotype; this might have resulted in the lower survival observed in XE-treated animals when compared to controls or RET-treated animals; thus, no attempt was made to use doses higher than 3 mg/kg to revert the pro-survival effect of compound **60**. Further experiments using different chronic models of epilepsy, such as the kainic acid-induced status epilepticus (KASE) model, will be necessary to better understand the mechanism of action of compound **60**.

### Pharmacokinetic Assessment

Given the favorable antiseizure effects shown by compound **60**, an initial in vivo assessment of its pharmacokinetic properties was performed. To this aim, the brain/plasma distribution in mice of compound **60** and retigabine was compared. The results obtained revealed that after 60 min i.p. administration of each drug at 1 mg/kg, the brain and plasma concentrations were 469.2 ± 142.8 and 202.2 ± 90.9 ng/mg for retigabine (*n* = 3) and 726.6 ± 178.8 and 18.2 ± 3.7 ng/mg for compound **60** (*n* = 3), respectively. Thus, compound **60** showed a remarkable brain accumulation, with a brain/plasma ratio 18-times higher than that of retigabine (40.4 vs 2.3, respectively). This result is likely explained by the higher lipophilicity (log *P* 4.74) of compound **60** over that of retigabine (log *P* 3.08). A similar increase in brain/plasma ratio (14-fold) was also observed for P-retigabine (log *P* 3.48) when compared to retigabine. Both its higher brain accumulation and increased potency as the Kv7.2/Kv7.3 activator likely contribute to the stronger antiseizure actions of compound **60** over that of retigabine. Finally, blood sampling at predetermined intervals (0, 0.5, 2, 4, 8, and 24 h) after i.p. administration of retigabine (3 mg/kg) or compound **60** (0.3 mg/kg) was performed to provide initial clues on time-dependent pharmacokinetics of compound **60** when compared to retigabine; these experiments required multiple blood sampling in a short time, and therefore rats instead of mice were used (*n* = 3 for each interval). The results obtained revealed that the plasma half-life of compound **60** (16.9 h) was about 5-times higher than that of retigabine (2.4 h, Figure S97). These results suggest that the longer plasma half-life of compound **60** might overcome another important limitation of retigabine, namely, its three times a day dosing requirement.^35^ Chronic administration studies will be needed to confirm such a hypothesis.

## Conclusions

Kv7 channels represent a relevant pharmacological target to develop novel ASMs; the prototype Kv7 activator retigabine has been marketed for a few years, but it has been discontinued for toxicity issues mostly unrelated to its mechanism of action. Thus, despite Kv7 channel activation being validated as an anticonvulsant mechanism, no drug is currently available to target this ion channel family. To overcome some of the limitations of retigabine, a small library of retigabine analogues has been designed, synthesized, and evaluated in the present study. Guided by molecular modeling and molecular dynamic studies and building upon new structure–activity relationships, a novel compound was identified (compound **60**); when compared to retigabine, compound **60** showed higher potency and efficacy as a Kv7 channel activator in vitro, no photo-induced dimer formation, higher brain/plasma ratio, and longer plasma half-life in vivo. All these pharmacokinetic and pharmacodynamic properties likely contributed to its increased antiseizure activity in vivo with respect to the parent compound. Overall, our results suggest that compound **60** might represent a promising lead compound for further development of novel Kv7 activators for clinical use in epilepsy and other hyperexcitability diseases.

## Experimental Section

### General

All reagents and solvents used were purchased from Sigma-Aldrich (Milan, Italy), unless otherwise noted. Reactions were carried out with magnetic stirring in round-bottom flasks except for microwave-assisted reactions that were conducted using glass vials and microwave closed vessel apparatus (Monowave 300, Anton Paar, Graz, Austria). Oven-dried glassware under a nitrogen stream and freshly distilled solvents were used to perform moisture-sensitive reactions. TLC analysis of reaction mixtures was performed over pre-coated glass silica gel plates (F254, 0.25 mm, VWR International, Radnor, USA), while crude products were purified by an automated flash chromatography system (Isolera Spektra one, Biotage, Uppsala, Sweden) coupled with an APCI mass detector (Dalton 2000, Biotage), using pre-loaded silica gel cartridges (SNAP KP-Sil, Biotage). NMR spectra were recorded on a Bruker Avance 400 MHz apparatus at room temperature. Chemical shifts are reported in δ values (ppm) relative to internal Me_4_Si, while *J* values are reported in Hertz (Hz). The following abbreviations are used to describe the ^1^H-NMR peaks: s (singlet), d (doublet), dd (double double), t (triplet), q (quadruplet), and m (multiplet). HR-MS analysis was conducted using the LTQ-Orbitrap-XL-ETD mass spectrometer (Thermo Scientific, Bremen, Germany), through an electrospray source. Purity of final compounds was assessed by UHPLC analyses, performed on a Nexera UHPLC system (Shimadzu, Kyoto, Japan) consisting of a CBM-20A controller, two LC-30 AD dual-plunger parallel-flow pumps, a DGU-20 A_R5_ degasser, an SPD-M20A photodiode array detector (equipped with a 2.5 μL detector flow cell volume), a CTO-20A column oven, and a SIL-30 AC autosampler. No unexpected or unusually high safety hazards were encountered. Purity assessment UHPLC runs were carried out on a EVO C18 150 × 2.1 mm × 2.6 μm (100 Å) column (Phenomenex, Bologna, Italy). The optimal mobile phase consisted of 0.1% HCOOH/H_2_O *v/v* (A) and 0.1% HCOOH/ACN *v/v* (B). Analysis was performed in gradient elution as follows: 0–20.00 min, 5–95% B; 20–25.00 min, 95–95% B; 25–31.00 min, isocratic to 5% B. Flow rate was 0.5 mL min^–1^. Column oven temperature was set to 40 °C. Injection volume was 2 μL of sample. The following PDA parameters were applied: sampling rate, 12.5 Hz; detector time constant, 0.160 s; cell temperature, 40 °C. Data acquisition was set in the range 190–800 nm, and chromatograms were monitored at 254 nm. Final compounds always showed a purity >95%. The corresponding chromatograms are reported in the Supplementary Information.

### General Procedures

#### General Procedure A: N-Acylation

Starting material (1.0 equiv) was dissolved in THF, DIPEA (1.2 equiv) and the proper acyl chloride (1.2 equiv) were added, and the solution was stirred at room temperature for 1 h. The mixture was then dried under vacuum and reconstituted in DCM. The organic phase was sequentially washed with a solution of K_2_CO_3_ and brine, extracted, dried over anhydrous Na_2_SO_4_, filtered, and concentrated in vacuo. The crude product was purified using a linear gradient of *n*-hexane/ethyl acetate.

#### General Procedure B: N-Acylation

Starting material (1.0 equiv), DBU (2 equiv), and the proper acyl halide (2 equiv) were dissolved in THF. The reaction was conducted under μW, at 140 °C, for 1 h with continuous stirring. The resulting mixture was then concentrated in vacuo, reconstituted in DCM, and washed with a solution of K_2_CO_3_ and brine. The organic phase was extracted, dried over anhydrous Na_2_SO_4_, filtered, and concentrated in vacuo. Crude products thus-obtained were purified using a linear gradient of *n*-hexane/ethyl acetate.

#### General Procedure C: N-Alkylation

Starting products (1.0 equiv) were dissolved in DMF. Then 1.2 equivalents of the proper substituted benzyl bromide and 1.2 equivalents of DIPEA were added. The reaction mixture was refluxed under stirring for 3 h. The resulting solution was then washed with a solution of K_2_CO_3_ and brine. The organic phase was extracted, dried over anhydrous Na_2_SO_4_, filtered and concentrated in vacuo. Crude product was purified using a linear gradient of *n*-hexane/ethyl acetate.

#### General Procedure D: Synthesis of N-Protected Derivatives (Carbamates)

Upon dissolution in THF, amines (1.0 equiv) were added with benzyl chloroformate (1.2 equiv) and DIPEA (1.2 equiv) or with Boc anhydride (1.2 equiv) and TEA (1.2 equiv), to obtain the N-Cbz and N-Boc-protected derivatives, respectively. The reaction mixtures were stirred at room temperature till reaction completion (30–60 min) to give the corresponding N-protected intermediates. Afterward, the reaction mixture was evaporated to dryness, reconstituted in DCM, washed with K_2_CO_3_, dried over anhydrous Na_2_SO_4_, and filtered. After concentrating in vacuo, crude products were purified by flash chromatography affording the corresponding N-protected derivatives.

#### General Procedure E: Catalytic Hydrogenation

To a solution of starting material (1.0 equiv) in THF/MeOH (1:1 v:v), ammonium formate (10 equiv) and Pd/C (6% mol) were added. The reaction mixture was refluxed at 100 °C for 1 h. After completion, the reaction solution was filtered through Celite 503 (Merck Millipore, Burlington, USA) and evaporated in vacuo. The resulting intermediates were used in the following step without further purification. Indeed, final products obtained with this procedure were purified by flash chromatography.

#### General Procedure F: Boc Removal

N-Boc-protected compounds (1.0 equiv) were dissolved in a mixture of DCM/TFA (3:1 v:v), and catalytic amounts of triethylsilane were added as a radical scavenger. The resulting mixtures were stirred at room temperature till reaction completion, as evidenced by TLC. Then, the solution was diluted with DCM, quenched with a solution of K_2_CO_3_, washed with water, and separated. Upon drying over anhydrous Na_2_SO_4_, filtration and concentration in vacuo afforded the crude products, which were purified by flash chromatography.

#### General Procedure G: Reductive Amination

Starting compounds (1.0 equiv) were dissolved in MeOH, and the proper aldehydes (1.2 equiv) and TFA (1.0 equiv) were added. The reaction was refluxed under stirring for 3 h and cooled to 0 °C, and 3 equivalents of NaBH_4_ was added. After stirring for 20 min, the solution was concentrated in vacuo, reconstituted in DCM, and washed with a solution of K_2_CO_3_ and brine. After drying over Na_2_SO_4_, the organic phase was filtered and dried under vacuum. Products thus-obtained were purified using linear gradients of *n*-hexane/ethyl acetate as the mobile phase.

#### General Procedure H: One-Pot NO_2_ Reduction and Selective Acylation

Starting compounds (1.0 equiv) were dissolved in methanol, and zinc powder (5 equiv) and NH_4_Cl (5 equiv) were added. The mixture was stirred at room temperature until disappearance of the starting material assessed by TLC. Then, different acyl halides (1.2 equiv) and DIPEA (1.2 equiv) were added, and the reaction was stirred at room temperature for a further 45 min. The resulting mixture was evaporated to dryness, reconstituted in DCM, and extracted with an aqueous solution of K_2_CO_3_ and brine. After drying over Na_2_SO_4_, the organic phase was filtered and concentrated in vacuo. Intermediates thus-obtained were purified using linear gradients of *n*-hexane/ethyl acetate as the mobile phase.

#### *N*-(4-Aminophenyl)heptanamide (**1**)

Synthesized from 1,4-phenylenediamine following general procedure A (yield 88%).

^1^H NMR (CDCl_3_, 400 MHz) δ: 0.91 (t, 3H, C*H*_3_, *J* = 6.8 Hz); 1.28–1.40 (m, 6H, 3 C*H*_2_); 1.70–1.74 (m, 2H, C*H*_2_); 2.32 (t, 2H, C*H*_2_, *J* = 7.7 Hz); 3.61 (bs, 2H, N*H*); 6.65 (d, 2H, aryl, *J* = 8.6 Hz); 7.14 (bs, 1H, CON*H*); 7.28 (d, 2H, aryl, *J* = 8.6 Hz); HR-MS *m/z*: calcd for C_13_H_21_N_2_O, [(M + H)^+^]: 221.1654; found 221.1658.

#### *N*-(4-((4-(Trifluoromethyl)benzyl)amino)phenyl)heptanamide (**2**)

Synthesized from **1** following general procedure C (yield 72%).

^1^H NMR (CD_3_OD, 400 MHz) δ: 0.92 (t, 3H, C*H*_3_, *J* = 6.9 Hz); 1.32–1.40 (m, 6H, 3 C*H*_2_); 1.64–1.71 (m, 2H, C*H*_2_); 2.31 (t, 2H, C*H*_2_, *J* = 7.6 Hz); 4.40 (s, 2H, C*H*_2_); 6.58 (d, 2H, aryl, *J* = 8.9 Hz); 7.23 (d, 2H, aryl, *J* = 8.8 Hz); 7.53–7.61 (m, 4H, aryl); ^13^C NMR (CD_3_OD, 100 MHz) δ: 13.0; 22.2; 25.7; 28.6; 31.3; 36.4;112.6; 122.1; 124.9; 127.3; 128.2; 128.5; 128.8; 145.1; 145.5; 173.0. ^19^F NMR (CDCl_3_, 376.3 MHz) δ: −62.44 (3F, C*F*_3_); HR-MS *m/z*: calcd for C_21_H_25_F_3_N_2_O, [(M + H)^+^]: 379.1997; found 379.2001.

#### 3-Nitro-*N*^1^-(4-(trifluoromethyl)benzyl)benzene-1,4-diamine (**3**)

Synthesized from 2-nitrobenzene-1,4-diamine following general procedure C (yield 69%).

^1^H NMR (CDCl_3_, 400 MHz) δ: 4.40 (s, 2H, C*H*_2_); 5.67 (bs, 2H, N*H*_2_); 6.73 (d, 1H, aryl, *J* = 8.7 Hz); 6.88 (dd, 1H, aryl, J_1_ = 6.5 Hz, J_2_ = 2.5 Hz); 7.28 (s, 1H, aryl); 7.51 (d, 2H, aryl, *J* = 7.5 Hz); 7.63 (d, 2H, aryl, *J* = 7.5 Hz); HR-MS *m/z*: calcd for C_14_H_13_F_3_N_3_O_2_, [(M + H)^+^]: 312.0960; found 312.0957.

#### Benzyl(4-amino-3-nitrophenyl)(4-(trifluoromethyl)benzyl)carbamate (**4**)

Synthesized from **3** following general procedure D for the synthesis of Cbz-protected derivatives. (yield 84%).

^1^H NMR (CDCl_3_, 400 MHz) δ: 4.90 (s, 2H, C*H*_2_); 5.20 (s, 2H, C*H*_2_); 6.16 (bs, 2H, N*H*_2_); 6.72 (d, 1H, aryl, *J* = 8.6 Hz); 7.26 (dd, 1H, aryl, J_1_ = 6.5 Hz, J_2_ = 2.3 Hz); 7.33–7.36 (m, 7H, aryl); 7.56 (d, 2H, aryl, *J* = 7.6 Hz); 7.94 (s, 1H, aryl); HR-MS *m/z*: calcd for C_22_H_19_F_3_N_3_O_4_, [(M + H)^+^]: 446.1328; found 446.1332.

#### *tert*-Butyl(4-amino-3-nitrophenyl)(4-(trifluoromethyl)benzyl)carbamate (**4′**)

Synthesized from **3** following general procedure D for the synthesis of Boc-protected derivatives (yield 79%).

^1^H NMR (CDCl_3_, 400 MHz) δ: 1.45 (s, 9H, 3 C*H*_3_); 4.86 (s, 2H, C*H*_2_); 6.72 (d, 1H, aryl, *J* = 8.9 Hz); 7.13 (bs, 1H, aryl); 7.37 (d, 2H, aryl, *J* = 7.9 Hz); 7.59 (d, 2H, aryl, *J* = 7.9 Hz); 7.93 (s, 1H, aryl); HR-MS *m/z*: calcd for C_19_H_21_F_3_N_3_O_4_, [(M + H)^+^]: 412.1484; found 412.1488.

#### Benzyl(4-heptanamido-3-nitrophenyl)(4-(trifluoromethyl)benzyl)carbamate (**5**)

Synthesized from **4** and heptanoyl chloride following general procedure B (yield 65%).

^1^H NMR (CDCl_3_, 400 MHz) δ: 0.91 (t, 3H, C*H*_3_, *J* = 6.7 Hz); 1.29–1.34 (m, 6H, 3 C*H*_2_); 1.75–1.78 (m, 2H, C*H*_2_); 2.49 (t, 2H, C*H*_2_, *J* = 7.4 Hz); 4.97 (s, 2H, C*H*_2_); 5.22 (s, 2H, C*H*_2_); 7.27–7.42 (m, 8H, aryl); 7.57 (d, 2H, aryl, *J* = 8.2 Hz); 8.06 (s, 1H, aryl); 8.78 (d, 1H, aryl, *J* = 8.8 Hz); 10.33 (s, 1H, CON*H*); HR-MS *m/z*: calcd for C_29_H_31_F_3_N_3_O_5_, [(M + H)^+^]: 588.2216; found 588.2212.

#### Benzyl(3-nitro-4-octanamidophenyl)(4-(trifluoromethyl)benzyl)carbamate (**6**)

Synthesized from **4** and octanoyl chloride following general procedure B (yield 68%).

^1^H NMR (CDCl_3_, 400 MHz) δ: 0.91 (t, 3H, C*H*_3_, *J* = 6.6 Hz); 1.29–1.34 (m, 8H, 4 C*H*_2_); 1.74–1.78 (m, 2H, C*H*_2_); 2.49 (t, 2H, C*H*_2_, *J* = 7.4 Hz); 4.97 (s, 2H, C*H*_2_); 5.22 (s, 2H, C*H*_2_); 7.25–7.36 (m, 8H, aryl); 7.57 (d, 2H, aryl, *J* = 8.1 Hz); 8.08 (s, 1H, aryl); 8.78 (d, 1H, aryl, *J* = 8.7 Hz); 10.33 (s, 1H, CON*H*); HR-MS *m/z*: calcd for C_30_H_33_F_3_N_3_O_5_, [(M + H)^+^]: 572.2372; found 572.2374.

#### Benzyl(4-decanamido-3-nitrophenyl)(4-(trifluoromethyl)benzyl)carbamate (**7**)

Synthesized from **4** and decanoyl chloride following general procedure B (yield 62%).

^1^H NMR (CDCl_3_, 400 MHz): δ: 0.92 (t, 3H, C*H*_3_, *J* = 6.8 Hz); 1.29–1.38 (m, 12H, 6 C*H*_2_); 1.73–1.78 (m, 2H, C*H*_2_); 2.49 (t, 2H, C*H*_2_, *J* = 7.5 Hz); 4.96 (s, 2H, C*H*_2_); 5.21 (s, 2H, C*H*_2_); 7.27–7.34 (m, 8H, aryl); 7.57 (d, 2H, aryl, *J* = 8.3 Hz); 8.06 (s, 1H, aryl); 8.78 (d, 1H, aryl, *J* = 8.9 Hz); 10.33 (s, 1H, CON*H*); HR-MS *m/z*: calcd for C_32_H_37_F_3_N_3_O_5_, [(M + H)^+^]: 600.2685; found 600.2688.

#### Benzyl(4-dodecanamido-3-nitrophenyl)(4-(trifluoromethyl)benzyl)carbamate (**8**)

Synthesized from **4** and dodecanoyl chloride following general procedure B (yield 55%).

^1^H NMR (CDCl_3_, 400 MHz): δ: 0.91 (t, 3H, C*H*_3_, *J* = 6.7 Hz); 1.26–1.37 (m, 16H, 8 C*H*_2_); 1.72–1.78 (m, 2H, C*H*_2_); 2.49 (t, 2H, C*H*_2_, *J* = 7.6 Hz); 4.96 (s, 2H, C*H*_2_); 5.21 (s, 2H, C*H*_2_); 7.26–7.35 (m, 8H, aryl); 7.57 (d, 2H, aryl, *J* = 8.4 Hz); 8.06 (s, 1H, aryl); 8.78 (d, 1H, aryl, *J* = 8.6 Hz); 10.32 (s, 1H, CON*H*); HR-MS *m/z*: calcd for C_34_H_41_F_3_N_3_O_5_, [(M + H)^+^]: 628.2998; found 628.3001.

#### Benzyl(4-(3,3-dimethylbutanamido)-3-nitrophenyl)(4-(trifluoromethyl)benzyl)carbamate (**9**)

Synthesized from **4** and 3,3-dimethylbutyryl chloride following general procedure B (yield 81%).

^1^H NMR (CDCl_3_, 400 MHz) δ: 1.13 (s, 9H, 3 C*H*_3_); 2.35 (s, 2H, C*H*_2_); 4.96 (s, 2H, C*H*_2_); 5.21 (s, 2H, C*H*_2_); 7.32–7.45 (m, 8H, aryl); 7.57 (d, 2H, aryl, *J* = 8.3 Hz); 8.06 (s, 1H, aryl); 8.80 (d, 1H, aryl, *J* = 8.7 Hz); 10.26 (s, 1H, CON*H*); HR-MS *m/z*: calcd for C_28_H_29_F_3_N_3_O_5_, [(M + H)^+^]: 544.2059; found 544.2057.

#### *tert*-Butyl (4-(3,3-dimethylbutanamido)-3-nitrophenyl)(4-(trifluoromethyl)benzyl)carbamate (**9′**)

Synthesized from **4′** and 3,3-dimethylbutyryl chloride following general procedure B (yield 77%).

^1^H NMR (CDCl_3_, 400 MHz) δ: 1.12 (s, 9H, 3 C*H*_3_); 1.46 (s, 9H, 3 C*H*_3_); 2.27 (s, 2H, C*H*_2_); 4.93 (s, 2H, C*H*_2_); 7.36 (d, 2H, aryl, *J* = 8.2 Hz); 7.44 (d, 1H, aryl, *J* = 7.6 Hz); 7.59 (d, 2H, aryl, *J* = 8.2 Hz); 8.08 (s, 1H, aryl); 8.75 (d, 1H, aryl, *J* = 8.7 Hz); 10.23 (s, 1H, CON*H*); HR-MS *m/z*: calcd for C_25_H_31_F_3_N_3_O_5_, [(M + H)^+^]: 510.2216; found 510.2219.

#### Benzyl(4-(cyclopropanecarboxamido)-3-nitrophenyl)(4-(trifluoromethyl)benzyl)carbamate (**10**)

Synthesized from **4** and cyclopropanecarbonyl chloride following general procedure B (yield 77%).

^1^H NMR (CDCl_3_, 400 MHz) δ: 0.87–0.91 (m, 2H, C*H*_2_); 0.94–1.00 (m, 2H, C*H*_2_); 1.65–1.68 (t, 1H, C*H*); 4.96 (s, 2H, C*H*_2_); 5.21 (s, 2H, C*H*_2_); 7.25–7.37 (m, 8H, aryl); 7.57 (d, 2H, aryl, *J* = 8.4 Hz); 8.06 (s, 1H, aryl); 8.75 (d, 1H, aryl, *J* = 8.9 Hz); 10.55 (s, 1H, CON*H*); HR-MS *m/z*: calcd for C_26_H_23_F_3_N_3_O_5_, [(M + H)^+^]: 514.1590; found 514.1592.

#### Benzyl(4-(3-cyclohexylpropanamido)-3-nitrophenyl)(4-(trifluoromethyl)benzyl)carbamate (**11**)

Synthesized from **4** and 3-cyclohexylpropanoyl chloride following general procedure B (yield 81%).

^1^H NMR (CDCl_3_, 400 MHz) δ: 0.92–1.00 (m, 2H, C*H*_2_); 1.18–1.31 (m, 4H, 2 C*H*_2_); 1.60–1.78 (m, 7H, 3 C*H*_2_ and C*H*); 2.52 (t, 2H, C*H*_2_, *J* = 8.1 Hz); 4.96 (s, 2H, C*H*_2_); 5.21 (s, 2H, C*H*_2_); 7.25–7.36 (m, 8H, aryl); 7.57 (d, 2H, aryl, *J* = 8.2 Hz); 8.06 (s, 1H, aryl); 8.77 (d, 1H, aryl, *J* = 8.7 Hz); 10.32 (s, 1H, CON*H*); HR-MS *m/z*: calcd for C_31_H_33_F_3_N_3_O_5_, [(M + H)^+^]: 584.2372; found 584.2369.

#### Benzyl(4-(2-(2-methoxyethoxy)acetamido)-3-nitrophenyl)(4-(trifluoromethyl)benzyl)carbamate (**12**)

Synthesized from **4** and (2-methoxyethoxy)acetyl chloride following general procedure B (yield 67%).

^1^H NMR (CDCl_3_, 400 MHz) δ: 3.42 (s, 3H, C*H*_3_); 3.68 (t, 2H, C*H*_2_, *J* = 4.7 Hz); 4.40 (t, 2H, C*H*_2_, *J* = 4.7 Hz); 4.50 (s, 2H, C*H*_2_); 5.21 (s, 2H, C*H*_2_); 6.91–7.16 (m, 8H, aryl); 7.35 (d, 2H, aryl, *J* = 8.2 Hz); 7.70 (s, 1H, aryl); 8.37 (d, 1H, aryl, *J* = 8.4 Hz); 9.16 (s, 1H, CON*H*); HR-MS *m/z*: calcd for C_27_H_27_F_3_N_3_O_7_, [(M + H)^+^]: 562.1801; found 562.1800.

#### *N*-(2-Amino-4-((4-(trifluoromethyl)benzyl)amino)phenyl)heptanamide (**13**)

Synthesized from **5** as an off-white solid in 86% yield using general procedure E.

^1^H NMR (CDCl_3_, 400 MHz): δ: 0.81 (t, 3H, C*H*_3_, *J* = 6.5 Hz); 1.19–1.25 (m, 6H, 3 C*H*_2_); 1.55–1.62 (m, 2H, C*H*_2_); 2.25 (t, 2H, C*H*_2_, *J* = 7.6 Hz); 4.27 (s, 2H, C*H*_2_); 5.95–6.00 (m, 2H, aryl); 6.65 (d, 1H, aryl, *J* = 8.4 Hz); 7.42 (d, 2H, aryl, *J* = 8.1 Hz); 7.48 (d, 2H, aryl, *J* = 8.1 Hz); ^13^C NMR (CD_3_OD, 100 MHz) δ: 13.0; 22.2; 25.7; 28.7; 31.3; 35.8;100.9; 104.2; 114.1; 124.8; 126.9; 127.3; 128.4; 128.8; 142.9; 145.2; 148.0; 174.1. ^19^F NMR (CDCl_3_, 376.3 MHz) δ: −62.43 (3F, C*F*_3_); HR-MS *m/z*: calcd for C_21_H_27_F_3_N_3_O, [(M + H)^+^]: 394.2106; found 394.2098.

#### *N*-(2-Amino-4-((4-(trifluoromethyl)benzyl)amino)phenyl)octanamide (**14**)

Synthesized from **6** as an off-white solid in 82% yield using general procedure E.

^1^H NMR (CD_3_OD, 400 MHz): δ: 0.93 (t, 3H, C*H*_3_, *J* = 6.9 Hz); 1.34–1.40 (m, 8H, 4 C*H*_2_); 1.67–1.74 (m, 2H, C*H*_2_); 2.37 (t, 2H, C*H*_2_, *J* = 7.6 Hz); 4.40 (s, 2H, C*H*_2_); 6.08–6.12 (m, 2H, aryl); 6.77 (d, 1H, aryl, *J* = 8.4 Hz); 7.55 (d, 2H, aryl, *J* = 8.3 Hz); 7.60 (d, 2H, aryl, *J* = 8.3 Hz); ^13^C NMR (CD_3_OD, 100 MHz) δ: 13.0; 22.3; 25.8; 28.8; 29.0; 31.5; 35.8;100.7; 114.1; 124.8; 126.7; 126.8; 127.3; 128.4; 128.7; 129.2; 142.8; 145.2; 148.0; 174.1. ^19^F NMR (CD_3_OD, 376.3 MHz) δ: -63.86 (3F, C*F*_3_); HR-MS *m/z*: calcd for C_22_H_29_F_3_N_3_O, [(M + H)^+^]: 408.2263; found 408.2271.

#### *N*-(2-Amino-4-((4-(trifluoromethyl)benzyl)amino)phenyl)decanamide (**15**)

Synthesized from **7** as an off-white solid in 83% yield using general procedure E.

^1^H NMR (CD_3_OD, 400 MHz) δ: 0.92 (t, 3H, C*H*_3_, *J* = 7.0 Hz); 1.23–1.42 (m, 12H, 6 C*H*_2_); 1.67–1.74 (m, 2H, C*H*_2_); 2.37 (t, 2H, C*H*_2_, *J* = 7.6 Hz); 4.40 (s, 2H, C*H*_2_); 6.07–6.12 (m, 2H, aryl); 6.78 (d, 1H, aryl, *J* = 8.4 Hz); 7.55 (d, 2H, aryl, *J* = 8.3 Hz); 7.60 (d, 2H, aryl, *J* = 8.3 Hz); ^13^C NMR (CD_3_OD, 100 MHz) δ: 13.0; 22.3; 25.8; 29.0; 29.2; 31.6; 35.8; 100.7; 104.2; 114.1; 124.8; 126. 9; 127.3; 142.8; 145.2; 148.0; 174.1. ^19^F NMR (CDCl_3_, 376.3 MHz) δ: −62.43 (3F, C*F*_3_); HR-MS *m/z*: calcd for C_24_H_33_F_3_N_3_O, [(M + H)^+^]: 436.2576; found 436.2582.

#### *N*-(2-Amino-4-((4-(trifluoromethyl)benzyl)amino)phenyl)dodecanamide (**16**)

Synthesized from **8** as a white wax in 79% yield using general procedure E.

^1^H NMR (CD_3_OD, 400 MHz) δ: 0.92 (t, 3H, C*H*_3_, *J* = 7.0 Hz); 1.23–1.38 (m, 16H, 8 C*H*_2_); 1.67–1.74 (m, 2H, C*H*_2_); 2.37 (t, 2H, C*H*_2_, *J* = 7.6 Hz); 4.39 (s, 2H, C*H*_2_); 6.07–6.12 (m, 2H, aryl); 6.78 (d, 1H, aryl, *J* = 8.4 Hz); 7.55 (d, 2H, aryl, *J* = 8.3 Hz); 7.60 (d, 2H, aryl, *J* = 8.3 Hz); ^13^C NMR (CD_3_OD, 100 MHz) δ: 13.0; 22.3; 25.8; 29.0; 29.3; 31.7; 35.8; 100.7; 104.2; 114.1; 124.8; 126.9; 127.3; 142.9; 145.2; 148.0; 174.1. ^19^F NMR (CDCl_3_, 376.3 MHz) δ: −62.43 (s, 3F, C*F*_3_); HR-MS *m/z*: calcd for C_26_H_37_F_3_N_3_O, [(M + H)^+^]: 464.2889; found 464.2885.

#### *N*-(2-Amino-4-((4-(trifluoromethyl)benzyl)amino)phenyl)-3,3-dimethylbutanamide (**17**)

Synthesized from **9** as a white powder in 86% yield using general procedure E.

^1^H NMR (CD_3_OD, 400 MHz) δ: 1.11 (s, 9H, 3 C*H*_3_); 2.25 (s, 2H, C*H*_2_); 4.40 (s, 2H, C*H*_2_); 6.07–6.12 (m, 2H, aryl); 6.77 (d, 1H, aryl, *J* = 8.4 Hz); 7.55 (d, 2H, aryl, *J* = 8.3 Hz); 7.60 (d, 2H, aryl, *J* = 8.3 Hz); ^13^C NMR (CD_3_OD, 100 MHz) δ: 28.9; 30.5; 49.2; 100.7; 104.2; 114.2; 124.79; 124.83; 126.9; 127.3; 142.9; 145.2; 148.0; 172.4. ^19^F NMR (CDCl_3_, 376.3 MHz) δ: −62.43 (3F, C*F*_3_); HR-MS *m/z*: calcd for C_20_H_25_F_3_N_3_O, [(M + H)^+^]: 380.1950; found 380.1958.

#### *tert*-Butyl(3-amino-4-(3,3-dimethylbutanamido)phenyl)(4-(trifluoromethyl)benzyl)carbamate (**17′**)

Synthesized from **9′** as a white powder in 84% yield using general procedure E.

^1^H NMR (CDCl_3_, 400 MHz) δ 1.11 (s, 9H, 3 C*H*_3_); 1.44 (s, 9H, 3 C*H*_3_); 2.25 (s, 2H, C*H*_2_); 4.80 (s, 2H, C*H*_2_); 6.51 (d, 1H, aryl, *J* = 7.6 Hz); 6.58 (s, 1H, aryl); 7.00 (s, 1H, aryl, *J* = 7.5 Hz); 7.36 (d, 2H, aryl, *J* = 8.2 Hz); 7.58 (d, 2H, aryl, *J* = 8.2 Hz); HR-MS *m/z*: calcd for C_25_H_33_F_3_N_3_O_3_, [(M + H)^+^]: 480.2474; found 480.2475.

#### *N*-(2-Amino-4-((4-(trifluoromethyl)benzyl)amino)phenyl)cyclopropanecarboxamide (**18**)

Synthesized from **10** as a gray powder in 81% yield using general procedure E.

^1^H NMR (CD_3_OD, 400 MHz) δ: 0.84 (d, 2H, C*H*_2_, *J* = 6.9 Hz); 0.93 (s, 2H, C*H*_2_); 1.77 (t, 1H, C*H*, *J* = 4.0 Hz); 4.39 (s, 2H, C*H*_2_); 6.08–6.12 (m, 2H, aryl); 6.81 (d, 1H, aryl, *J* = 8.0 Hz); 7.55 (d, 2H, aryl, *J* = 7.6 Hz); 7.60 (d, 2H, aryl, *J* = 7.6 Hz); ^13^C NMR (CD_3_OD, 100 MHz) δ: 6.3; 13.5; 100.7; 104.2; 114.4; 124.8; 126.8; 127.3; 142.8; 145.3; 148.0; 174.2. ^19^F NMR (CDCl_3_, 376.3 MHz) δ: −62.44 (3F, C*F*_3_); HR-MS *m/z*: calcd for C_18_H_19_F_3_N_3_O, [(M + H)^+^]: 350.1480; found 350.1469.

#### *N*-(2-Amino-4-((4-(trifluoromethyl)benzyl)amino)phenyl)-3-cyclohexylpropanamide (**19**)

Synthesized from **11** as an off-white solid in 78% yield using general procedure E.

^1^H NMR (CD_3_OD, 400 MHz) δ: 0.93–1.02 (m, 2H, C*H*_2_); 1.16–1.34 (m, 4H, 2 C*H*_2_); 1.57–1.82 (m, 7H, 3 C*H*_2_ and C*H*); 2.38 (t, 2H, C*H*_2_, *J* = 8.0 Hz); 4.39 (s, 2H, C*H*_2_); 6.07–6.12 (m, 2H, aryl); 6.77 (d, 1H, aryl, *J* = 8.4 Hz); 7.55 (d, 2H, aryl, *J* = 8.2 Hz); 7.60 (d, 2H, aryl, *J* = 8.2 Hz); ^13^C NMR (CD_3_OD, 100 MHz) δ: 26.0; 26.3; 32.8; 33.4; 37.4; 100.7; 104.2; 114.1; 124.8; 126.9; 127.3; 142.9; 145.2; 148.0; 174.4. ^19^F NMR (CDCl_3_, 376.3 MHz) δ: −62.42 (3F, C*F*_3_); HR-MS *m/z*: calcd for C_23_H_29_F_3_N_3_O, [(M + H)^+^]: 420.2263; found 420.2271;

#### *N*-(2-Amino-4-((4-(trifluoromethyl)benzyl)amino)phenyl)-2-(2-methoxyethoxy)acetamide (**20**)

Synthesized from **12** as a white powder in 71% yield using general procedure E.

^1^H NMR (CD_3_OD, 400 MHz) δ: 3.41 (s, 3H, C*H*_3_); 3.64 (d, 2H, C*H*_2_, *J* = 2.7 Hz); 3.78 (d, 2H, C*H*_2_, *J* = 2.7 Hz); 4.14 (s, 2H, C*H*_2_); 4.40 (s, 2H, C*H*_2_); 6.09–6.12 (m, 2H, aryl); 6.88 (d, 1H, aryl, *J* = 8.3 Hz); 7.55 (d, 2H, aryl, *J* = 7.8 Hz); 7.61 (d, 2H, aryl, *J* = 7.8 Hz); ^13^C NMR (CD_3_OD, 100 MHz) δ: 46.7; 57.8; 70.1; 70.6; 71.4; 100.4; 104.0; 112.8; 124.8; 126.9; 127.3; 142.9; 145.2; 148.2; 170.3. ^19^F NMR (CDCl_3_, 376.3 MHz) δ: −62.42 (3F, C*F*_3_); HR-MS *m/z*: calcd for C_19_H_23_F_3_N_3_O, [(M + H)^+^]: 398.1692; found 398.1701.

#### *tert*-Butyl(4-(3,3-dimethylbutanamido)-3-(pyrrolidin-1-yl)phenyl)(4-(trifluoromethyl)benzyl)carbamate (**21**)

Intermediate **21** was synthesized slightly modifying the procedure previously described by Xu and coworkers^48^ (1.0 equiv). Intermediate **17′** was dissolved in acetonitrile, and K_2_CO_3_ (2 equiv), KI (2 equiv), and 1,4-dibromobutane (2.0 equiv) were added. The mixture was refluxed under stirring overnight. The organic phase was concentrated in vacuo and diluted with DCM. The DCM was then washed with K_2_CO_3_, dried over anhydrous Na_2_SO_4_, filtered, and concentrated in vacuo. The crude product was purified using a linear gradient of *n*-hexane/ethyl acetate.

^1^H NMR (CDCl_3_, 400 MHz) δ: 1.13 (s, 9H, 3 C*H*_3_); 1.43 (s, 9H, 3 C*H*_3_); 1.59–1.62 (m, 2H, C*H*_2_); 1.70–1.72 (m, 4H, 2 C*H*_2_); 2.26 (s, 2H, C*H*_2_); 2.92 (s, 4H, 2 C*H*_2_); 4.84 (s, 2H, C*H*_2_); 6.84 (d, 1H, aryl, *J* = 8.7 Hz); 7.35 (d, 2H, aryl, *J* = 8.2 Hz); 7.59 (d, 2H, aryl, *J* = 8.2 Hz); 8.09 (s, 1H, aryl); 8.20 (d, 1H, aryl, *J* = 8.4 Hz); HR-MS *m/z*: calcd for C_29_H_39_F_3_N_3_O_3_, [(M + H)^+^]: 534.2944; found 534.2947.

#### *tert*-Butyl(4-(3,3-dimethylbutanamido)-3-(piperidin-1-yl)phenyl)(4-(trifluoromethyl)benzyl)carbamate (**22**)

Intermediate **22** was synthesized reacting **17′** with 1,5-dibromopentane under the same experimental conditions described for **21**.

^1^H NMR (CDCl_3_, 400 MHz) δ: 1.13 (s, 9H, 3 C*H*_3_); 1.43 (s, 9H, 3 C*H*_3_); 1.92 (s, 4H, 2 C*H*_2_); 2.26 (s, 2H, C*H*_2_); 2.68 (s, 4H, 2 C*H*_2_); 4.84 (s, 2H, C*H*_2_); 6.89 (d, 1H, aryl, *J* = 8.7 Hz); 7.36 (d, 2H, aryl, *J* = 8.2 Hz); 7.57 (d, 2H, aryl, *J* = 8.2 Hz); 8.35 (d, 1H, aryl, *J* = 8.7 Hz); 8.44 (s, 1H, aryl); HR-MS *m/z*: calcd for C_29_H_39_F_3_N_3_O_3_, [(M + H)^+^]: 534.2944; found 534.2947.

#### 3,3-Dimethyl-n-(2-(pyrrolidin-1-yl)-4-((4-(trifluoromethyl)benzyl)amino)phenyl)butanamide (**23**)

Synthesized from **21** as a white solid in 92% yield following general procedure E**.**

^1^H NMR (CD_3_OD, 400 MHz) δ: 1.10 (s, 9H, 3 C*H*_3_); 1.87–1.90 (m, 4H, 2 C*H*_2_); 2.22 (s, 2H, C*H*_2_); 3.13 (t, 4H, 2 C*H*_2_, *J* = 6.5 Hz); 4.41 (s, 2H, C*H*_2_); 6.14 (dd, 1H, aryl, J_1_ = 6.0 Hz, J_2_ = 2.5 Hz); 6.18 (d, 1H, aryl, *J* = 2.4 Hz); 6.96 (d, 1H, aryl, *J* = 8.4 Hz); 7.56 (d, 2H, aryl, *J* = 8.3 Hz); 7.60 (d, 1H, aryl, *J* = 8.4 Hz); ^13^C NMR (CD_3_OD, 100 MHz) δ: 24.6; 28.9; 30.7; 49.3; 50.2; 100.7; 104.1; 116.4; 124.79; 124.83; 127.4; 128.4; 145.4; 146.0; 146.1; 147.5; 172.7. ^19^F NMR (CD_3_OD, 376.3 MHz) δ: −63.84 (3F, C*F*_3_); HR-MS *m/z*: calcd for C_24_H_31_F_3_N_3_O, [(M + H)^+^]: 434.2419; found 434.2415.

#### 3,3-Dimethyl-*N*-(2-(piperidin-1-yl)-4-((4-(trifluoromethyl)benzyl)amino)phenyl)butanamide (**24**)

Synthesized from **22** as a white solid in 89% yield following general procedure E.

^1^H NMR (CDCl_3_, 400 MHz) δ: 1.12 (s, 9H, 3 C*H*_3_); 1.59 (d, 2H, C*H*_2,_*J* = 4.0 Hz); 1.70–1.73 (m, 4H, 2 C*H*_2_); 2.23 (s, 2H, C*H*_2_); 2.74 (t, 4H, 2 C*H*_2_, *J* = 4.7 Hz); 4.10 (bs, 1H. NH); 4.39 (s, 2H, C*H*_2_); 6.39 (d, 1H, aryl, *J* = 8.7 Hz); 6.44 (s, 1H, aryl); 7.49 (d, 2H, aryl, *J* = 7.9 Hz); 7.60 (d, 2H, aryl, *J* = 7.9 Hz); 8.16 (s, 1H, CON*H*), 8.20 (d, 1H, aryl, *J* = 8.7 Hz); ^13^C NMR (CDCl_3_, 100 MHz) δ: 24.1; 27.0; 29.9; 48.2; 52.2; 53.7; 105.8; 108.8; 120.6; 122.8; 125.0; 125.50; 125.55; 127.6; 129.3; 129.6; 143.8; 144.1; 169.2. ^19^F NMR (CDCl_3_, 376.3 MHz) δ: −62.42 (3F, C*F*_3_); HR-MS *m/z*: calcd for C_25_H_33_F_3_N_3_O, [(M + H)^+^]: 448.2576; found 448.2577.

#### *N*-(2-Amino-4-(methyl(4-(trifluoromethyl)benzyl)amino)phenyl)heptanamide (**25**)

Synthesized following general procedure G from **13** and formaldehyde as a white powder in 67% yield.

^1^H NMR (CD_3_OD, 400 MHz) δ: 0.82 (t, 3H, C*H*_3_, *J* = 6.5 Hz); 1.19–1.31 (m, 6H, 3 C*H*_2_); 1.56–1.63 (m, 2H, C*H*_2_); 2.26 (t, 2H, C*H*_2_, *J* = 7.4 Hz); 2.89 (s, 3H, C*H*_3_); 4.47 (s, 2H, C*H*_2_); 6.08 (dd, 1H, aryl, J_1_ = 7.1 Hz, J_2_ = 1.5 Hz); 6.16 (s, 1H, aryl); 6.75 (d, 1H, aryl, *J* = 8.6 Hz); 7.29 (d, 2H, aryl, *J* = 7.8 Hz); 7.47 (d, 2H, aryl, *J* = 7.8 Hz); ^13^C NMR (CD_3_OD, 100 MHz) δ: 13.0; 22.2; 25.7; 28.7; 31.3; 35.8; 37.85; 55.7; 100.8; 103.5; 114.2; 124.9; 126.9; 127.1; 142.9; 144.1; 149.2; 174.1. ^19^F NMR (CDCl_3_, 376.3 MHz) δ: −62.41 (3F, C*F*_3_); HR-MS *m/z*: calcd for C_22_H_29_F_3_N_3_O, [(M + H)^+^]: 408.2263; found 408.2257.

#### *N*-(2-Amino-4-(methyl(4-(trifluoromethyl)benzyl)amino)phenyl)-3,3-dimethylbutanamide (**26**)

Synthesized following general procedure G from **17** and formaldehyde as a white powder in 63% yield.

^1^H NMR (CD_3_OD, 400 MHz) δ: 1.12 (s, 9H, 3 C*H*_3_); 2.26 (s, 2H, C*H*_2_); 3.01 (s, 3H, C*H*_3_); 4.60 (s, 2H, N*H*_2_); 6.20 (dd, 1H, aryl, *J*_1_ = 6.1 Hz, J_2_ = 2.6 Hz); 6.28 (d, 1H, aryl, *J* = 2.7 Hz); 6.87 (d, 1H, aryl, *J* = 8.6 Hz); 7.41 (d, 2H, aryl, *J* = 7.8 Hz); 7.59 (d, 2H, aryl, *J* = 7.8 Hz); ^13^C NMR (CD_3_OD, 100 MHz) δ: 29.0; 30.5; 37.9; 49.2; 55.7; 100.9; 103.5; 114.3; 124.9; 125.8; 127.1; 128.5; 128.8; 142.9; 144.1; 149.1; 172.4. ^19^F NMR (CD_3_OD, 376.3 MHz) δ: −63.76 (3F, C*F*_3_); HR-MS *m/z*: calcd for C_21_H_27_F_3_N_3_O, [(M + H)^+^]: 394.2106; found 394.2111.

#### *N*-(2-Amino-4-(propyl(4-(trifluoromethyl)benzyl)amino)phenyl)heptanamide (**27**)

Synthesized following general procedure G from **13** and propionaldehyde as a white powder in 71% yield.

^1^H NMR (CD_3_OD, 400 MHz) δ: 0.82 (t, 3H, C*H*_3_, *J* = 6.5 Hz); 1.19–1.31 (m, 6H, 3 C*H*_2_); 1.56–1.63 (m, 2H, C*H*_2_); 2.26 (t, 2H, C*H*_2_, *J* = 7.4 Hz); 2.89 (s, 3H, C*H*_3_); 4.47 (s, 2H, C*H*_2_); 6.08 (dd, 1H, aryl, J_1_ = 7.1 Hz, J_2_ = 1.5 Hz); 6.16 (s, 1H, aryl); 6.75 (d, 1H, aryl, *J* = 8.6 Hz); 7.29 (d, 2H, aryl, *J* = 7.8 Hz); 7.47 (d, 2H, aryl, *J* = 7.8 Hz); ^13^C NMR (CD_3_OD, 100 MHz) δ: 13.0; 22.2; 25.7; 28.7; 31.3; 35.8; 37.85; 55.7; 100.8; 103.5; 114.2; 124.9; 126.9; 127.1; 142.9; 144.1; 149.2; 174.1. ^19^F NMR (CDCl_3_, 376.3 MHz) δ: −62.41 (3F, C*F*_3_); HR-MS *m/z*: calcd for C_22_H_29_F_3_N_3_O, [(M + H)^+^]: 408.2263; found 408.2257.

#### *N*-(2-Amino-4-(*N*-(4-(trifluoromethyl)benzyl)acetamido)phenyl)heptanamide (**28**)

Synthesized from **13** and acetyl chloride following general procedure A as a white powder in 77% yield.

^1^H NMR (CD_3_OD, 400 MHz) δ: 0.92–0.98 (m, 6H, 2 C*H*_3_); 1.31–1.45 (m, 6H, 3 C*H*_2_); 1.67–1.74 (m, 4H, 2 C*H*_2_); 2.37 (t, 2H, C*H*_2_, *J* = 7.6 Hz); 3.38 (t, 2H, C*H*_2_, *J* = 7.7 Hz); 4.62 (s, 2H, C*H*_2_); 6.12 (dd, 1H, aryl, J_1_ = 8.7 Hz, J_2_ = 2.8 Hz); 6.21 (d, 1H, aryl, *J* = 2.7 Hz);6.82 (d, 1H, aryl, *J* = 8.7 Hz); 7.41 (d, 2H, aryl, *J* = 8.1 Hz); 7.58 (d, 2H, aryl, *J* = 8.1 Hz); ^13^C NMR (CD_3_OD, 100 MHz) δ: 10.2; 13.0; 20.2; 22.2; 25.7; 28.7; 31.3; 35.8; 53.2; 53.9; 100.7; 103.5; 113.8; 124.9; 126.9; 142.9; 144.5; 148.0; 174.1. ^19^F NMR (CD_3_OD, 376.3 MHz) δ: −63.82 (3F, C*F*_3_); HR-MS *m/z*: calcd for C_24_H_33_F_3_N_3_O, [(M + H)^+^]: 436.2576; found 436.2583.

#### 2-Nitro-4-((4-(trifluoromethyl)benzyl)oxy)aniline (**29**)

To a solution of 4-amino-3-nitrophenol in DMF were added 4-(trifluoromethyl)benzyl bromide (1.2 equiv), K_2_CO_3_ (1.2 equiv), and a catalytic amount of KI (5% mol). The mixture was refluxed under magnetic stirring for 3 h. Upon cooling to room temperature, DCM was added and the mixture was extracted with aqueous solution of Na_2_CO_3_ and brine. The organic phase was dried over Na_2_SO_4_, filtered, and concentrated in vacuo. Intermediate **29** was obtained in 67% yield after flash-chromatographic purification using linear gradients of *n*-hexane/ethyl acetate as the mobile phase.

^1^H NMR (CDCl_3_, 400 MHz) δ: 5.11 (s, 2H, C*H*_2_); 5.94 (bs, 2H, N*H*_2_); 6.81 (d, 1H, aryl, *J* = 9.1 Hz); 6.16 (dd, 1H, aryl, *J*_1_ = 6.1 Hz, J_2_ = 3.0 Hz); 7.57 (d, 2H, aryl, *J* = 8.0 Hz); 7.66–7.69 (m, 3H, ary); HR-MS *m/z*: calcd for C_14_H_12_F_2_N_2_O_3_, [(M + H)^+^]: 313.0800; found 313.0805.

#### *N*-(2-Nitro-4-((4-(trifluoromethyl)benzyl)oxy)phenyl)heptanamide (**30**)

Prepared from **29** in 65% yield following general procedure B.

^1^H NMR (CDCl_3_, 400 MHz) δ: 0.81 (t, 3H, C*H*_3_, *J* = 6.8 Hz); 1.19–1.26 (m, 6H, 3 C*H*_2_); 1.61–1.71 (m, 2H, C*H*_2_); 2.39 (t, 2H, C*H*_2_, *J* = 7.7 Hz); 5.10 (s, 2H, C*H*_2_); 7.22 (dd, 1H, aryl, J_1_ = 6.1 Hz, J_2_ = 3.0 Hz); 7.48 (d, 2H, aryl, *J* = 7.9 Hz); 7.60 (d, 2H, aryl, *J* = 7.9 Hz); 7.68 (d, 1H, aryl, *J* = 6.4 Hz); 8.65 (d, 1H, aryl, *J* = 8.7 Hz); 10.05 (s, 1H, CON*H*); HR-MS *m/z*: calcd for C_21_H_24_F_3_N_2_O_4_, [(M + H)^+^]: 425.1688; found 425.1691.

#### *N*-(2-Amino-4-((4-(trifluoromethyl)benzyl)oxy)phenyl)heptanamide (**31**)

Synthesized according to general procedure E starting from intermediate **30**. The final compound **31** was isolated in 89% yield as an off-white solid.

^1^H NMR (CD_3_OD, 400 MHz) δ: 0.82 (t, 3H, C*H*_3_, *J* = 6.6 Hz); 1.26–1.33 (m, 6H, 3 C*H*_2_); 1.59–1.66 (m, 2H, C*H*_2_); 2.37 (t, 2H, C*H*_2_, *J* = 7.6 Hz); 5.15 (s, 2H, C*H*_2_); 6.96 (s, 1H, aryl); 7.04 (d, 1H, aryl, *J* = 9.8 Hz); 7.13 (d, 1H, aryl, *J* = 8.8 Hz); 7.55 (d, 2H, aryl, *J* = 8.1 Hz); 7.60 (d, 2H, aryl, *J* = 8.1 Hz); ^13^C NMR (CD_3_OD, 100 MHz) δ: 13.0; 22.2; 25.1; 28.7; 31.3; 35.8; 69.3; 110.2; 115.3; 124.6; 125.1; 126.5; 126.7; 127.5; 129.7; 130.0; 141.1; 157.4; 174.6. ^19^F NMR (CD_3_OD, 376.3 MHz) δ: −62.66 (3F, C*F*_3_); HR-MS *m/z*: calcd for C_21_H_26_F_3_N_2_O_2_, [(M + H)^+^]: 395.1946; found 395.1948.

#### *N*^1^-Benzyl-3-nitrobenzene-1,4-diamine (**32**)

Intermediate **33** was prepared in 59% yield from 2-nitrobenzene-1,4-diamine and (bromomethyl)benzene following general procedure C.

^1^H NMR (CDCl_3_, 400 MHz) δ: 4.32 (s, 2H, C*H*_2_); 5.74 (bs, 2H, N*H*_2_); 6.72 (d, 1H, aryl, *J* = 8.9 Hz); 6.88 (dd, 1H, aryl, J_1_ = 6.6 Hz, J_2_ = 2.8 Hz,); 7.22 (d, 2H, aryl, *J* = 7.9 Hz); 7.31–7.39 (m, 6H, aryl,); HR-MS *m/z*: calcd for C_13_H_14_N_3_O_2_, [(M + H)^+^]: 244.1086; found 244.1089.

#### 4-(4-Fluorophenethyl)-2-nitroaniline (**33**)

Intermediate **34** was prepared in 62% yield from 2-nitrobenzene-1,4-diamine and 1-(bromomethyl)-4-fluorobenzene following general procedure C.

^1^H NMR (CDCl_3_, 400 MHz) δ: 2.92 (t, 2H, *J* = 6.8 Hz); 3.37 (t, 2H, *J* = 6.8 Hz); 5.73 (bs, 2H, N*H*_2_); 6.72 (d, 1H, aryl, *J* = 8.9 Hz); 6.81 (dd, 1H, aryl, J_1_ = 6.2 Hz, J_2_ = 2.7 Hz); 7.03 (t, 2H, aryl, *J* = 8.6 Hz); 7.18–7.22 (m, 2H, aryl); 7.31 (d, 1H, aryl, *J* = 2.4 Hz). HR-MS *m/z*: calcd for C_14_H_14_FN_3_O_2_, [(M + H)^+^]: 276.2857; found 276.2860.

#### *N*^1^-(2,6-Difluorobenzyl)-3-nitrobenzene-1,4-diamine (**34**)

Intermediate **35** was synthesized in 67% yield starting from 2-nitrobenzene-1,4-diamine and 2-(bromomethyl)-1,3-difluorobenzene following general procedure C.

^1^H NMR (CDCl_3_, 400 MHz) δ: 4.32 (s, 2H, C*H*_2_); 5.74 (bs, 2H, N*H*_2_); 6.72 (d, 1H, aryl, *J* = 8.9 Hz); 6.89–6.93 (m, 3H, aryl); 7.21–7.28 (m, 1H, aryl); 7.46 (d, 1H, aryl, *J* = 2.8 Hz); HR-MS *m/z*: calcd for C_13_H_14_N_3_O_2_, [(M + H)^+^]: 244.1086; found 244.1089.

#### Benzyl(4-amino-3-nitrophenyl)(benzyl)carbamate (**35**)

Intermediate **35** was attained by Cbz protection of **32** in 71% yield following the general procedure D.

^1^H NMR (CDCl_3_, 400 MHz) δ: 4.85 (s, 2H, C*H*_2_); 5.20 (s, 2H, C*H*_2_); 6.06 (bs, 2H, N*H*_2_); 6.69 (d, 1H, aryl, *J* = 8.7 Hz); 7.20 (d, 2H, aryl, *J* = 8.1 Hz); 7.23–7.28 (m, 5H, aryl,); 7.35–7.39 (m, 3H, aryl); 7.92 (s, 1H, aryl); HR-MS *m/z*: calcd for C_21_H_20_N_3_O_4_, [(M + H)^+^]: 378.1454; found 378.1451.

#### Benzyl(4-amino-3-nitrophenyl)(4-fluorophenethyl)carbamate (**36**)

Intermediate **36** was attained by Cbz protection of **33** in 62% yield following general procedure D.

^1^H NMR (CDCl_3_, 400 MHz) δ: 2.87 (t, 2H, C*H*_2_, *J* = 7.0 Hz); 3.87 (t, 2H, C*H*_2_, *J* = 7.0 Hz); 5.15 (s, 2H, C*H*_2_); 6.13 (bs, 2H, N*H*_2_); 6.75 (d, 1H, aryl, *J* = 8.7 Hz); 6.95 (t, 2H, aryl, *J* = 8.4 Hz); 7.02–7.15 (m, 5H, aryl); 7.20–7.40 (m, 3H, aryl); 7.88 (s, 1H, aryl). HR-MS *m/z*: calcd for C_22_H_21_FN_3_O_4_, [(M + H)^+^]: 410.1511; found 410.1515.

#### Benzyl(4-amino-3-nitrophenyl)(2,6-difluorobenzyl)carbamate (**37**)

Intermediate **37** was attained by Cbz protection of **34** in 69% yield following general procedure D.

^1^H NMR (CD_3_OD, 400 MHz) δ: 5.09 (s, 2H, C*H*_2_); 5.22 (s, 2H, C*H*_2_); 6.88 (t, 2H, aryl, *J* = 8.1 Hz); 7.26–7.48 (m, 8H, aryl); 7.95 (s, 1H, aryl); 8.22 (d, 1H, aryl, *J* = 9.0 Hz); HR-MS *m/z*: calcd for C_21_H_18_F_2_N_3_O_4_, [(M + H)^+^]: 414.1216; found 414.1218.

#### Benzyl Benzyl(4-heptanamido-3-nitrophenyl)carbamate (**38**)

Intermediate **38** was attained from **36** and heptanoyl chloride in 75% yield following general procedure B.

^1^H NMR (CDCl_3_, 400 MHz) δ: 0.91 (t, 3H, C*H*_3_, *J* = 6.7 Hz); 1.29–1.41 (m, 6H, 3 C*H*_2_); 1.72–1.79 (m, 2H, C*H*_2_); 2.48 (t, 2H, C*H*_2_, *J* = 7.6 Hz); 4.92 (s, 2H, C*H*_2_); 5.22 (s, 2H, C*H*_2_); 7.20 (dd, 2H, aryl, J_1_ = 6.0 Hz, J_2_=; 1.3 Hz); 7.28–7.42 (m, 9H, aryl); 8.04 (s, 1H, aryl); 8.75 (d, 1H, aryl, *J* = 9.1 Hz); 10.31 (s, 1H, CON*H*); HR-MS *m/z*: calcd for C_28_H_32_N_3_O_5_, [(M + H)^+^]: 490.2342; found 490.2337.

#### Benzyl 4-Fluorophenethyl(4-heptanamido-3-nitrophenyl)carbamate (**39**)

Intermediate **39** was attained from **36** and heptanoyl chloride in 72% yield following general procedure B.

^1^H NMR (CDCl_3_, 400 MHz) δ: 0.91 (t, 3H, C*H*_3_, *J* = 6.8 Hz); 1.29–1.42 (m, 6H, 3 C*H*_2_); 1.76–1.80 (m, 2H, C*H*_2_); 2.51 (t, 2H, C*H*_2_, *J* = 7.7 Hz); 2.88 (t, 2H, C*H*_2_, *J* = 7.2 Hz); 3.93 (t, 2H, C*H*_2_, *J* = 7.2 Hz); 5.16 (s, 2H, C*H*_2_); 6.95 (t, 2H, aryl, *J* = 8.6 Hz); 7.05–7.08 (m, 2H, aryl); 7.05–7.38 (m, 6H, aryl); 7.93 (s, 1H, aryl); 8.79 (d, 1H, aryl, *J* = 8.9 Hz); 10.35 (s, 1H CON*H*); HR-MS *m/z*: calcd for C_29_H_33_FN_3_O_5_, [(M + H)^+^]: 522.2404; found 522.2410.

#### Benzyl 2,6-Difluorobenzyl(4-(3,3-dimethylbutanamido)-3-nitrophenyl)carbamate (**40**)

Intermediate **40** was attained from **37** and 3,3-dimethylbutyryl chloride in 68% yield following general procedure B.

^1^H NMR (CDCl_3_, 400 MHz) δ: 1.13 (s, 9H, 3 C*H*_3_); 2.34 (s, 2H, C*H*_2_); 5.07 (s, 2H, C*H*_2_); 5.20 (s, 2H, C*H*_2_); 6.82 (t, 2H, aryl, *J* = 7.9 Hz); 7.19–7.43 (m, 7H, aryl); 8.04 (s, 1H, aryl); 8.78 (d, 1H, aryl, *J* = 9.1 Hz); 10.30 (s, 1H, CON*H*); HR-MS *m/z*: calcd for C_27_H_28_F_2_N_3_O_5_, [(M + H)^+^]: 512.1992; found 512.1996.

#### *N*-(2-Amino-4-(benzylamino)phenyl)heptanamide (**41**)

Final product **41** was synthesized in 87% yield as a white powder by reduction of intermediate **38** under the conditions described in general procedure E.

^1^H NMR (CD_3_OD, 400 MHz) δ: 0.82 (t, 3H, C*H*_3_, *J* = 6.4 Hz); 1.19–1.25 (m, 6H, 3 C*H*_2_); 1.55–1.62 (m, 2H, C*H*_2_); 2.25 (t, 2H, C*H*_2_, *J* = 7.6 Hz); 4.17 (s, 2H, C*H*_2_); 5.99 (d, 1H, aryl, *J* = 8.4 Hz); 6.04 (s, 1H, aryl); 6.65 (d, 1H, aryl, *J* = 8.4 Hz); 7.07–7.11 (m, 1H, aryl); 7.17 (t, 1H, aryl, *J* = 7.2 Hz); 7.24 (d, 2H, aryl, *J* = 7.4 Hz); ^13^C NMR (CD_3_OD, 100 MHz) δ: 13.0; 22.2; 25.7; 28.7; 31.3; 35.8; 100.9; 104.3; 114.0; 126.3; 126.8; 126.9; 128.0; 140.2; 142.7; 148.4; 174.1. HR-MS *m/z*: calcd for C_20_H_28_N_3_O, [(M + H)^+^]: 326.2232; found 326.2230.

#### *N*-(2-Amino-4-((4-fluorophenethyl)amino)phenyl)heptanamide (**42**)

Final product **42** was synthesized in 87% yield as an off-white powder by reduction of intermediate **39** under the conditions described in general procedure E.

^1^H NMR (CD_3_OD, 400 MHz) δ: 0.94 (t, 3H, C*H*_3_, *J* = 6.8 Hz); 1.35–1.43 (m, 6H, 3 C*H*_2_); 1.68–1.76 (m, 2H, C*H*_2_); 2.38 (t, 2H, C*H*_2_, *J* = 7.6 Hz); 2.86 (t, 2H, C*H*_2_, *J* = 7.2 Hz); 3.29 (t, 2H, C*H*_2_, *J* = 7.6 Hz); 6.11 (dd, 1H, aryl, *J*_1_ = 6.0 Hz, J_2_ = 2.4 Hz); 6.19 (d, 1H, aryl, *J* = 2.4 Hz); 6.82 (d, 1H, aryl, *J* = 8.4 Hz); 7.02 (t, 2H, aryl, *J* = 8.8 Hz); 7.23–7.27 (m, 2H, aryl); ^13^C NMR (CD_3_OD, 100 MHz) δ: 13.0; 22.2; 25.7; 28.7; 31.3; 34.2; 35.8; 45.3; 100.8; 104.3; 114.2; 114.7; 126.9; 130.1; 135.8; 142.9; 148.2; 160.3; 162.7; 174.1. ^19^F NMR (CDCl_3_, 376.3 MHz) δ: −62.41 (1F, C*F*); HR-MS *m/z*: calcd for C_21_H_29_FN_3_O, [(M + H)^+^]: 358.2295; found 358.2296;

#### *N*-(2-Amino-4-((2,6-difluorobenzyl)amino)phenyl)-3,3-dimethylbutanamide (**43**)

Final product **47** was synthesized in 83% yield as a pink powder by reduction of intermediate **40** under the conditions described in general procedure E.

^1^H NMR (CD_3_OD, 400 MHz) δ: 1.11 (s, 9H, 3 C*H*_3_); 2.24 (s, 2H, C*H*_2_); 4.35 (s, 2H, C*H*_2_); 6.18 (d, 1H, aryl, J_1_ = 6.0 Hz, *J*_2_ = 2.5 Hz); 6.26 (d, 1H, aryl, *J* = 2.5 Hz); 6.79 (d, 1H, aryl, *J* = 8.4 Hz); 6.95 (t, 2H, aryl, *J* = 8.1 Hz); 7.26–7.33 (m, 1H, aryl); ^13^C NMR (CD_3_OD, 100 MHz) δ: 29.0; 35.4; 49.2; 101.2; 104.1; 110.8; 111.0; 114.7; 114.9; 115.1; 115.3; 126.9; 129.1; 129.2; 129.3; 142.9; 147.7; 160.5; 160.6, 162.9; 163.0; 172.4; ^19^F NMR (CDCl_3_, 376.3 MHz) δ: −117.3 (2F, C*F*); HR-MS *m/z*: calcd for C_19_H_24_F_2_N_3_O, [(M + H)^+^]: 348.1882; found 348.1889.

#### 3-Nitro-*N*^1^-(pyridin-2-ylmethyl)benzene-1,4-diamine (**44**)

Synthesized in 54% yield from 2-nitrobenzene-1,4-diamine and pyridine 2-carboxaldehyde using the reductive amination conditions described for general procedure G.

^1^H NMR (CDCl_3_, 400 MHz) δ: 4.86 (s, 2H, C*H*_2_); 5.75 (bs, 2H, N*H*_2_); 6.74 (d, 1H, aryl, *J* = 8.8 Hz); 6.98 (dd, 1H, aryl, J_1_ = 6.2 Hz, J_2_ = 2.6 Hz); 7.25–7.39 (m, 3H, aryl); 7.72–7.75 (m, 1H, aryl); 8.61 (d, 1H, aryl, *J* = 3.9 Hz); HR-MS *m/z*: calcd for C_12_H_13_N_4_O_2_, [(M + H)^+^]: 245.1039; found 245.1042.

#### Benzyl(4-amino-3-nitrophenyl)(pyridin-2-ylmethyl)carbamate (**45**)

Synthesized in 78% yield from **44** using the conditions described for Cbz protection in general procedure D.

^1^H NMR (CDCl_3_, 400 MHz) δ: 4.97 (s, 2H, C*H*_2_); 5.19 (s, 2H, C*H*_2_); 6.10 (bs, 2H, N*H*_2_); 6.73 (d, 1H, aryl, *J* = 8.8 Hz); 7.18–7.34 (m, 8H, aryl); 7.64–7.68 (m, 1H, aryl); 8.04 (s, 1H, aryl); 8.55 (d, 1H, aryl, *J* = 3.8 Hz). HR-MS *m/z*: calcd for C_20_H_19_N_4_O_4_, [(M + H)^+^]: 379.1406; found 379.1403.

#### Benzyl(4-heptanamido-3-nitrophenyl)(pyridin-2-ylmethyl)carbamate (**46**)

Synthesized in 81% yield from **45** and heptanoyl chloride as described in general procedure B.

^1^H NMR (CDCl_3_, 400 MHz) δ: 0.94 (t, 3H, C*H*_3_, *J* = 6.8 Hz); 1.35–1.43 (m, 6H, 3 C*H*_2_); 1.68–1.76 (m, 2H, C*H*_2_); 2.48 (t, 2H, C*H*_2_, *J* = 7.6 Hz); 5.01 (s, 2H, C*H*_2_); 5.20 (s, 2H, C*H*_2_); 7.19–7.32 (m, 7H, aryl); 7.63–7.67 (m, 1H, aryl); 8.26 (s, 1H, aryl); 8.56 (d, 1H, aryl, *J* = 3.7 Hz); 8.76 (d, 1H, aryl, *J* = 9.1 Hz); 10.31 (s, 1H, CON*H*); HR-MS *m/z*: calcd for C_27_H_31_N_4_O_5_, [(M + H)^+^]: 491.2294; found 491.2297.

#### *N*-(2-Amino-4-((pyridin-2-ylmethyl)amino)phenyl)heptanamide (**47**)

Final product **47** was synthesized as an off-white solid starting from **46** and using the reaction conditions described in general procedure E.

^1^H NMR (CD_3_OD, 400 MHz) δ: 0.94 (t, 3H, C*H*_3_, *J* = 6.8 Hz); 1.31–1.43 (m, 6H, 3 C*H*_2_); 1.67–1.74 (m, 2H, C*H*_2_); 2.37 (t, 2H, C*H*_2_, *J* = 7.7 Hz); 4.41 (s, 2H, C*H*); 6.07 (d, 1H, aryl, J_1_ = 6.1 Hz, J_2_ = 2.3 Hz); 6.12 (d, 1H, aryl, *J* = 2.4 Hz); 6.78 (d, 1H, aryl, *J* = 8.4); 7.28 (t, 1H, aryl, *J* = 6.7 Hz); 7.47 (d. 1H, aryl, *J* = 7.9 Hz); 7.76 (t, 1H, aryl, *J* = 6.1 Hz); 8.48 (d, 1H, aryl, *J* = 4.5 Hz); ^13^C NMR (CD_3_OD, 100 MHz) δ: 13.0; 22.2; 25.7; 28.7; 31.3; 35.8; 48.5; 100.6; 104.0; 114.2; 121.6; 122.1; 127.0; 137.3; 143.0; 147.9; 148.2; 160.0; 174.1. HR-MS *m/z*: calcd for C_19_H_27_N_4_O, [(M + H)^+^]: 327.2185; found 327.2191.

#### *tert*-Butyl(4-amino-3-nitrophenyl)carbamate (**48**)

Synthesized in 67% yield from 2-nitrobenzene-1,4-diamine using the conditions described for Boc protection in general procedure D.

^1^H NMR (CDCl_3_, 400 MHz) δ: 1.53 (s, 9H, 3 C*H*_3_); 5.95 (bs, 2H, N*H*_2_); 6.46 (s, 1H, N*H*); 6.77 (d, 1H, aryl, *J* = 9.0 Hz); 7.52 (s, 1H, aryl); 8.06 (s, 1H, aryl); HR-MS *m/z*: calcd for C_11_H_16_N_3_O_4_, [(M + H)^+^]: 254.1141; found 254.1146.

#### *tert*-Butyl(4-heptanamido-3-nitrophenyl)carbamate (**49**)

Intermediate **49** was synthesized in 72% yield by acylation of **48** following general procedure B.

^1^H NMR (CDCl_3_, 400 MHz) δ 0.91 (t, 3H, C*H*_3_, *J* = 6.6 Hz); 1.28–1.40 (m, 6H, 3 C*H*_2_); 1.52 (s, 9H, 3 C*H*_3_); 1.75–1.78 (m, 2H, C*H*_2_); 2.48 (t, 2H, C*H*_2_, *J* = 7.8 Hz); 6.78 (bs, 1H, N*H*); 7.54 (d, 1H, aryl *J* = 7.4 Hz); 8.40 (s, 1H, aryl); 8.71 (d, 1H, aryl, *J* = 9.1 Hz); 10.17 (s, 1H CON*H*); HR-MS *m/z*: calcd for C_18_H_28_N_3_O_5_, [(M + H)^+^]: 366.2029; found 366.2035.

#### *N*-(4-Amino-2-nitrophenyl)heptanamide (**50**)

Intermediate **50** was synthesized in 88% yield from **49** following the method for Boc removal described in general procedure F.

^1^H NMR (CDCl_3_, 400 MHz) δ: 0.91 (t, 3H, C*H*_3_, *J* = 6.6 Hz); 1.27–1.40 (m, 6H, 3 C*H*_2_); 1.73–1.77 (m, 2H, C*H*_2_); 2.45 (t, 2H, C*H*_2_, *J* = 7.8 Hz); 4.51 (bs, 2H, N*H*_2_); 6.98 (d, 1H, aryl *J* = 7.2 Hz); 7.47 (s, 1H, aryl); 8.48 (d, 1H, aryl, *J* = 9.3 Hz); 9.94 (s, 1H CON*H*); HR-MS *m/z*: calcd for C_13_H_20_N_3_O_3_, [(M + H)^+^]: 266.1505; found 266.1509.

#### *N*-(2-Amino-4-((pyridin-4-ylmethyl)amino)phenyl)heptanamide (**51**)

Compound **51** was synthesized slightly modifying the last stages of general procedure F. In particular, intermediate **50** (1.0 equiv) was dissolved in MeOH and pyridine 4-carboxaldehyde (1.2 equiv) and TFA (1.0 equiv) were added. The reaction was refluxed under stirring for 3 h. Upon cooling the reaction mixture to 0 °C, NaBH_4_ was added together to Pd/C 10% (6% mol). In this way, simultaneous reduction of the imine intermediate and of the 2-nitro group was attained. The resulting solution was filtered through Celite 503, concentrated, and reconstituted in DCM. Extraction with aqueous K_2_CO_3_ and brine, separation of the organic phase, drying over anhydrous Na_2_SO_4_, filtration, and concentration in vacuo led to the crude product, which was purified by flash chromatography. Final product **51** was isolated as a gray oil in 57% yield.

^1^H NMR (CD_3_OD, 400 MHz) δ: 0.94 (t, 3H, C*H*_3_, *J* = 6.7 Hz); 1.34–1.43 (m, 6H, 3 C*H*_2_); 1.67–1.74 (m, 2H, C*H*_2_); 2.37 (t, 2H, C*H*_2_, *J* = 7.6 Hz); 4.38 (s, 2H, C*H*); 6.04–6.09 (m, 2H, aryl); 6.78 (t, 1H, aryl, *J* = 3.8 Hz); 6.78 (d, 1H, aryl, *J* = 8.4); 7.44 (d. 2H, aryl, *J* = 5.5 Hz); 8.43 (d, 2H, aryl, *J* = 5.5 Hz); ^13^C NMR (CD_3_OD, 100 MHz) δ: 13.0; 22.2; 25.7; 28.7; 31.3; 35.8; 46.0; 100.5; 103.9; 114.2; 122.5; 127.0; 143.0; 147.8; 148.4; 151.8; 174.1. HR-MS *m/z*: calcd for C_19_H_27_N_4_O, [(M + H)^+^]: 327.2185; found 327.2179.

#### *N*-(2-Amino-4-((2-fluorobenzyl)amino)phenyl)heptanamide (**52**)

Compound **52** was synthesized as a white powder in 66% yield starting from **50** and 2-fluoro carboxaldehyde using the same procedure described above for **51**.

^1^H NMR (CD_3_OD, 400 MHz) δ: 0.94 (t, 3H, C*H*_3_, *J* = 6.6 Hz); 1.31–1.43 (m, 6H, 3 C*H*_2_); 1.67–1.74 (m, 2H, C*H*_2_); 2.37 (t, 2H, C*H*_2_, *J* = 7.5 Hz); 4.35 (s, 2H, C*H*_2_); 6.11 (d, 1H, aryl, *J* = 8.5 Hz); 6.16 (s, 1H, aryl); 6.78 (d, 1H, aryl, *J* = 8.5 Hz); 7.06–7.11 (m, 2H, aryl); 7.22–7.27 (m, 1H, aryl); 7.40 (t, 1H, aryl, *J* = 7.4 Hz); ^13^C NMR (CD_3_OD, 100 MHz) δ: 13.0; 22.2; 25.7; 28.7; 31.3; 35.8; 40.6; 100.7; 104.1; 114.2; 114.4; 114.6; 123.7; 126.9; 128.1; 129.01; 129.06; 142.9; 148.1; 159.7; 162.1; 174.1. ^19^F NMR (CDCl_3_, 376.3 MHz) δ: −119.13 (1F, C*F*); HR-MS *m/z*: calcd for C_20_H_27_FN_3_O, [(M + H)^+^]: 344.2138; found 344.2141.

#### Benzyl (4-formylphenyl) carbonate (53)

Intermediate **53** was attained in 72% yield by Cbz protection of the phenol moiety in 4-hydroxybenzaldehyde as described in general procedure D.

^1^H NMR (CDCl_3_, 400 MHz): δ: 5.32 (s, 2H, C*H*_2_); 7.39–7.46 (m, 7H, aryl); 7.95 (d, 2H, aryl, 7.9 Hz); 10.02 (s, CO*H*). HR-MS *m/z*: calcd for C_15_H_13_O_4_, [(M + H)^+^]: 257.0814; found 257.0821.

#### Benzyl(4-(hydroxymethyl)phenyl) carbonate (**54**)

Intermediate **54** was synthesized from **53** following a previously described procedure.^17^ Briefly, **55** (1.0 equiv) was dissolved in methanol and the solution was cooled in an ice bath before portionwise addition of 3 equivalents of NaBH_4_. After warming to room temperature, the reaction was stirred for a further 15 min. The reaction mixture was quenched with aqueous 2 N HCl. The aqueous phase was extracted with ethyl acetate, and the resulting organic phase was washed two times with brine, dried over anhydrous Na_2_SO_4_, filtered, and evaporated *in vacuo*. After flash chromatographic purification, **56** was obtained in 81% yield.

^1^H NMR (CDCl_3_, 400 MHz) δ: 4.63 (d, 2H, C*H*_2_, *J* = 3.5 Hz); 5.29 (s, 2H, C*H*_2_); 7.17 (d, 2H, aryl, *J* = 8.4 Hz); 7.35 (d, 2H, aryl, *J* = 8.4 Hz); 7.41–7.48 (m, 5H, aryl,); HR-MS *m/z*: calcd for C_15_H_15_O_4_, [(M + H)^+^]: 259.0970; found 259.0973.

#### Benzyl(4-(iodomethyl)phenyl) carbonate (**55**)

Synthesized from **54** according to the procedure previously described.^49^ Iodine (1.5 equiv) and triphenylphosphine (1.5 equiv) were solubilized in dry DCM and stirred under a nitrogen stream for 1.5 h. Then, 1.5 equivalents of imidazole was added and the solution was stirred for a further 1 h. Intermediate **54** (1.0 equiv) was added to the resulting slurry that was stirred at room temperature overnight. The resulting suspension was quenched with a saturated aqueous solution of Na_2_S_2_O_3_, and the organic layer was separated and further washed with brine. The resulting organic solution was dried over Na_2_SO_4_, filtered, and concentrated in vacuo. Compound **55** was obtained in 62% yield after purification of the crude product by flash chromatography using a linear gradient of *n*-hexane/ethyl acetate as the mobile phase.

^1^H NMR (CDCl_3_, 400 MHz) δ: 4.47 (s, 2H, C*H*_2_); 5.29 (s, 2H, C*H*_2_); 7.14 (d, 2H, aryl, *J* = 8.6 Hz); 7.40–7.46 (m, 7H, aryl); HR-MS *m/z*: calcd for C_15_H_13_IO_3_, [(M + H)^+^]: 367.9909; found 367.9912.

#### Benzyl(4-(((4-heptanamido-3-nitrophenyl)amino)methyl)phenyl) carbonate (**56**)

Intermediate **56** was synthesized in 59% yield starting from compounds **50** and **55** and following the method described in general procedure C.

^1^H NMR (CDCl_3_, 400 MHz) δ: 0.90 (t, 3H, C*H*_3_, *J* = 6.6 Hz); 1.32–1.39 (m, 6H, 3 C*H*_2_); 1.73–1.77 (m, 2H, C*H*_2_); 2.45 (t, 2H, C*H*_2_, *J* = 7.6 Hz); 4.61 (s, 2H, C*H*_2_); 4.66 (s, 2H, C*H*_2_); 5.88 (bs, 1H, N*H*); 6.80–6.83 (m, 2H, aryl); 7.03–7.10 (m, 3H, aryl,); 7.21–7.24 (m, 2H, aryl); 7.27–7.40 (m, 3H, aryl); 7.49 (d, 1H, aryl, *J* = 2.5 Hz); 8.40 (d, 1H, aryl, *J* = 9.2 Hz); 9.86 (s, 1H, CON*H*); HR-MS *m/z*: calcd for C_28_H_32_N_3_O_6_, [(M + H)^+^]: 506.2291; found 506.2298.

#### *N*-(2-Amino-4-((4-hydroxybenzyl)amino)phenyl)heptanamide (**57**)

Final product **57** was isolated as a dark gray powder in 82% yield starting from intermediate **56** and following general procedure E for catalytic hydrogenation.

^1^H NMR (CD_3_OD, 400 MHz) δ: 0.94 (t, 3H, C*H*_3_, *J* = 6.5 Hz); 1.31–1.44 (m, 6H, 3 C*H*_2_); 1.67–1.74 (m, 2H, C*H*_2_); 2.37 (t, 2H, C*H*_2_, *J* = 7.6 Hz); 4.17 (s, 2H, C*H*_2_); 6.12 (d, 1H, aryl, *J* = 8.4 Hz); 6.18 (s, 1H, aryl); 6.72–6.79 (m, 3H, aryl); 7.18 (d, 2H, aryl, *J* = 8.2 Hz); ^13^C NMR (CD_3_OD, 100 MHz) δ: 13.0; 22.2; 25.7; 28.7; 31.3; 35.8; 101.2; 104.5; 114.1; 114.7; 126.8; 128.3; 130.7; 142.7; 148.5; 156.0; 174.1. HR-MS *m/z*: calcd for C_20_H_28_NO_2_, [(M + H)^+^]: 342.2182; found 342.2171.

#### 2-Fluoro-4-nitro-*N*^1^-(4-(trifluoromethyl)benzyl)benzene-1,3-diamine (**58**)

2,3-Difluoro-6-nitroaniline (1.0 equiv), 4-trifluoromethyl benzylamine (1.2 equiv), DIPEA (1.2 equiv), and a catalytic amount of I_2_ were dissolved in DMF. The solution was warmed for 4 h at 170 °C.^11^ The reaction was washed successively with a saturated solution of Na_2_S_2_O_3_, K_2_CO_3_, and brine. The organic phase was extracted, dried over anhydrous Na_2_SO_4_, filtered, and concentrated in vacuo. Crude product was purified using a linear gradient of *n*-hexane/ethyl acetate giving intermediate **65** in 81% yield.

^1^H NMR (CD_3_OD, 400 MHz) δ: 4.49 (s, 2H, C*H*_2_); 5.95 (dd, 1H, aryl, *J*_1_ = 1.5 Hz, *J*_2_ = 8.1 Hz); 7.42 (d, 2H, aryl, *J* = 8.0 Hz); 7.53 (d, 2H, aryl, *J* = 8.0 Hz); 7.64 (dd, 1H, aryl, *J*_1_ = 8.0 Hz, *J*_2_ = 1.7 Hz); HR-MS *m/z*: calcd for C_14_H_12_ F_4_ N_3_O_2_, [(M + H)^+^]: 330.0866; found 330.0862.

#### *N*-(2-Amino-3-fluoro-4-((4-(trifluoromethyl)benzyl)amino)phenyl)heptanamide (**59**)

Synthesized in 59% yield as an off-white powder by the reaction of intermediate **58** with heptanoyl chloride under the conditions described in general procedure H.

^1^H NMR (DMSO-*d*_6_, 400 MHz) δ: 0.87 (t, 3H, C*H*_3_, *J* = 6.7 Hz); 1.27–1.31 (m, 6H, 3 C*H*_2_); 1.53–1.57 (m, 2H, C*H*_2_); 2.24 (t, 2H, C*H*_2_, *J* = 7.4 Hz); 4.39 (d, 2H, C*H*_2_, *J* = 6.0 Hz); 4.57 (bs, 2H, N*H*_2_); 5.78, (t, 1H, N*H*, *J* = 8.8 Hz); 6.02 (t, 1H, aryl, *J* = 5.8 Hz); 6.58 (d, 1H, aryl, *J* = 8.4 Hz); 7.55 (d, 2H, aryl, *J* = 8.0 Hz); 7.66 (d, 2H, aryl, *J* = 8.0 Hz); 8.98 (s, 1H, CONH); ^13^C NMR (DMSO-*d*_6_, 100 MHz) δ: 14.3; 22.5; 25.7; 28.8; 31.5; 36.0; 46.2; 100.1; 115.6; 121.5; 125.5; 127.6; 128.0; 131.4; 131.5; 134.5; 139.6; 141.8; 146.2; 171.9. ^19^F NMR (CDCl_3_, 376.3 MHz) δ: −60.72 (3F, C*F*_3_); −154.89 (1F, C*F*); HR-MS *m/z*: calcd for C_21_H_26_F_4_N_3_O, [(M + H)^+^]: 412.2012; found 412.2018.

#### *N*-(2-Amino-3-fluoro-4-((4-(trifluoromethyl)benzyl)amino)phenyl)-3,3-dimethylbutanamide (**60**)

Synthesized in 66% yield as a white powder by the reaction of intermediate **58** with 3,3-dimethylbutyryl chloride under the conditions described in general procedure H.

^1^H NMR (CD_3_OD, 400 MHz) δ: 1.10 (s, 9H, 3 C*H*_3_); 2.25 (s, 2H, C*H*_2_); 4.47 (s, 2H, C*H*_2_); 5.99 (t, 1H, aryl, *J* = 8.8 Hz); 6.60 (dd, 1H, aryl, *J*_1_ = 6.7 Hz, *J*_2_ = 1.9 Hz); 7.54 (d, 2H, aryl, *J* = 8.2 Hz); 7.60 (d, 2H, aryl, *J* = 8.2 Hz); ^13^C NMR (CD_3_OD, 100 MHz) δ: 28.9; 30.5; 46.3; 49.1; 101.5; 115.2; 121.2; 124.90; 124.93; 127.2; 130.9; 131.0; 135.0; 135.1; 140.2; 142.5; 145.0; 172.5. ^19^F NMR (CD_3_OD, 376.3 MHz) δ: −62.48 (3F, C*F*_3_); −158.0 (1F, C*F*); HR-MS *m/z*: calcd for C_20_H_24_F_4_N_3_O, [(M + H)^+^]: 398.1856; found 398.1867.

#### (*E*,*Z*)-1-Nitro-4-(4-(trifluoromethyl)styryl)benzene (**61a** and **61b**)

4-Nitrobenzaldehyde (1.0 equiv) was dissolved in dichloromethane (DCM, 26.7 mL per gram of aldehyde). and 4-trifluoromethylbenzyltriphenylphosphonium bromide, prepared from 4-(trifluoromethyl)benzyl bromide and triphenylphosphine as described earlier,^4^ and a catalytic amount of tetrabutylammonium bromide (TBAB, 0.2% mol) were added. To this solution, half volume of aqueous K_2_CO_3_ (1.0 equiv) was added and the resulting mixture was allowed to stir at room temperature for 12 h. The reaction was then diluted with DCM and water, and the organic phase was extracted, dried over anhydrous Na_2_SO_4_, filtered, and concentrated in vacuo. The crude product was purified by flash chromatography using hexane/ethyl acetate (95:5 v:v) obtaining the mixture of geometric isomers of 1-nitro-4-(4-(trifluoromethyl)styryl)benzene as a yellow solid.

^1^H NMR (CD_3_Cl_3_, 400 MHz) δ: 6.76 (d, 1H, CH, *J* = 12.3 Hz); 6.84 (d, 1H, CH, *J* = 12.3 Hz); 7.28 (d, 1H, CH, *J* = 9.0 Hz); 7.33 (d, 2H, aryl, *J* = 8.2 Hz); 7.37 (d, 2H, aryl, *J* = 8.6 Hz); 7.53 (d, 2H, aryl, *J* = 8.2 Hz); 7.68 (d, 1H, CH, *J* = 9.0 Hz); 8.11 (d, 2H, aryl, *J* = 8.8 Hz); HR-MS *m/z*: calcd for C_15_H_11_F_3_NO_2_ [M + H^+^]: calcd: 294.0742, found: 294.0749.

#### 4-(4-(Trifluoromethyl)phenethyl)aniline (**62**)

Compound **79** was synthesized as an off-white solid starting from the mixture of **78a** and **78b** and following general procedure E for catalytic hydrogenation.

^1^H NMR (CD_3_Cl_3_, 400 MHz) δ: 2.87 (t, 2H, CH_2_, *J* = 5.4 Hz); 2.96 (t, 2H, CH_2_, *J* = 5.3 Hz); 3.61 (bs, 2H, NH_2_,); 6.66–6.68 (m, 2H, aryl); 6.97–7.00 (m, 2H, aryl); 7.27–7.31 (m, 2H, aryl); 7.55–7.57 (m, 2H, aryl); HR-MS *m/z*: calcd for C_15_H_15_F_3_N [M + H^+^]: calcd: 266.1151, found: 266.1153.

#### *N*-(4-(4-(Trifluoromethyl)phenethyl)phenyl)heptanamide (**63**)

Compound **80** was synthesized as a gray solid in 71% yield, starting from **79** and heptanoyl chloride, using general procedure B for N-acylation.

^1^H NMR (CDCl_3_, 400 MHz) δ: 0.91 (t, 3H, C*H*_3_, *J* = 7.0 Hz); 1.30–1.39 (m, 6H, 3 C*H*_2_); 1.70–1.78 (m, 2H, C*H*_2_); 2.37 (t, 2H, C*H*_2_, *J* = 7.9 Hz); 2.91 (t, 2H, C*H*_2_, *J* = 6.3 Hz); 2.97 (t, 2H, C*H*_2_, *J* = 6.3 Hz); 7.10 (d, 2H, aryl, *J* = 8.3 Hz); 7.27 (d, 2H, aryl, *J* = 8.0 Hz); 7.46 (d, 2H, aryl, *J* = 8.0 Hz); 7.54 (d, 2H, aryl, *J* = 8.1 Hz); HR-MS *m/z*: calcd for C_22_H_27_F_3_NO, [(M + H)^+^]: 378.2045; found 378.2051.

#### 3,3-Dimethyl-*N*-(4-(4-(trifluoromethyl)phenethyl)phenyl)butanamide (**64**)

Compound **81** was synthesized as a gray solid in 78% yield, starting from **79** and 3,3-dimethylbutyryl chloride, using general procedure B for N-acylation.

^1^H NMR (CDCl_3_, 400 MHz) δ: 1.12 (s, 9H, 3 C*H*_3_); 2.24 (s, 2H, C*H*_2_); 2.91 (t, 2H, C*H*_2_, *J* = 6.5 Hz); 2.97 (t, 2H, C*H*_2_, *J* = 6.5 Hz); 7.11 (d, 2H, aryl, *J* = 8.4 Hz); 7.27 (d, 2H, aryl, *J* = 7.72 Hz); 7.45 (d, 2H, aryl, *J* = 8.4 Hz); 7.54 (d, 2H, aryl, *J* = 8.0 Hz); HR-MS *m/z*: calcd for C_21_H_25_F_3_NO, [(M + H)^+^]: 364.1888; found 364.1885.

#### *N*-(2-Nitro-4-(4-(trifluoromethyl)phenethyl)phenyl)heptanamide (**65**)

Intermediate **80** (1.0 equiv) was dissolved in acetic anhydride (40 mL per g of product) and cooled to 0 °C. Then, HNO_3_ (0.41 mL per mmol of starting product) was added dropwise during 15 min. The reaction was stirred for a further 30 min at the same temperature and then water was added, leading to the precipitation of a yellow solid. The slurry was filtered, and the residue was washed with water. After collection, the yellow solid was dried under vacuum and used in the following synthetic step without further purification (83% yield).

^1^H NMR (CD_3_OD, 400 MHz) δ: 0.94 (t, 3H, C*H*_3_, *J* = 7.0 Hz); 1.35–1.44 (m, 6H, 3 C*H*_2_); 1.68–1.76 (m, 2H, C*H*_2_); 2.45 (t, 2H, C*H*_2_, *J* = 7.6 Hz); 3.06 (s, 4H, 2 C*H*_2_); 7.39 (d, 2H, aryl, *J* = 8.0 Hz); 7.50 (dd, 1H, aryl, J_1_ = 6.4 Hz, J_2_ = 2.0 Hz); 7.57 (d, 2H, aryl, *J* = 8.1 Hz); 7.90 (d, 1H, aryl, *J* = 2.0 Hz); 7.93 (d, 1H, aryl, *J* = 8.4 Hz); HR-MS *m/z*: calcd for C_22_H_26_F_3_N_2_O_3_, [(M + H)^+^]: 423.1896; found 423.1901.

#### 3,3-Dimethyl-*N*-(2-nitro-4-(4-(trifluoromethyl)phenethyl)phenyl)butanamide (**66**)

Intermediate **83** was synthesized according to the procedure described above for **82.** The product was collected as a yellow solid in 79% yield.

^1^H NMR (CDCl_3_, 400 MHz) δ: 1.14 (s, 9H, 3 C*H*_3_); 2.33 (s, 2H, C*H*_2_); 3.01 (s, 4H, 2 C*H*_2_); 7.29 (d, 2H, aryl, *J* = 7.9 Hz); 7.45 (dd, 1H, aryl, *J*_1_ = 6.6 Hz, *J*_2_ = 2.1 Hz); 7.57 (d, 2H, aryl, *J* = 7.9 Hz); 8.01 (d, 1H, aryl, *J* = 2.1 Hz); 8.74 (d, 1H, aryl, *J* = 8.6 Hz); 10.24 (s, 1H, N*H*); HR-MS *m/z*: calcd for C_21_H_24_F_3_N_2_O_3_, [(M + H)^+^]: 409.1739; found 409.1743.

#### *N*-(2-Amino-4-(4-(trifluoromethyl)phenethyl)phenyl)heptanamide (**67**)

Final product **84** was synthesized in 92% yield as a white solid, starting from compound **82** and using general procedure E for catalytic hydrogenation.

^1^H NMR (CD_3_OD, 400 MHz) δ: 0.94 (t, 3H, C*H*_3_, *J* = 6.7 Hz); 1.37–1.45 (m, 6H, 3 C*H*_2_); 1.69–1.77 (m, 2H, C*H*_2_); 2.42 (t, 2H, C*H*_2_, *J* = 7.6 Hz); 2.86 (t, 2H, C*H*_2_, *J* = 6.9 Hz); 2.99 (t, 2H, C*H*_2_, *J* = 6.9 Hz); 6.58 (dd, 1H, aryl, J_1_ = 6.4 Hz, J_2_ = 2.1 Hz); 6.72 (d, 1H, aryl, *J* = 1.7 Hz); 6.99 (d, 1H, aryl, *J* = 8.0 Hz); 7.36 (d, 2H, aryl, *J* = 7.8 Hz); 7.54 (d, 2H, aryl, *J* = 7.5 Hz); ^13^C NMR (CD_3_OD, 100 MHz) δ: 13.0; 22.2; 25.7; 28.7; 31.3; 35.9; 36.7; 37.1; 117.2; 118.5; 121.9; 124.7; 125.7; 127.6; 127.9; 128.8; 140.3; 141.7; 146.3; 173.8: ^19^F NMR (CD_3_OD, 376.3 MHz) δ: −63.81 (3F, C*F*_3_); HR-MS *m/z*: calcd for C_23_H_23_F_3_N_2_O, [(M + H)^+^]: 393.2154; found 393.2157.

#### *N*-(2-Amino-4-(4-(trifluoromethyl)phenethyl)phenyl)-3,3-dimethylbutanamide (**68**)

Final product **84** was synthesized in 87% yield as a white solid, starting from compound **83** and using general procedure E for catalytic hydrogenation.

^1^H NMR (CD_3_OD, 400 MHz) δ: 1.13 (s, 9H, 3 C*H*_3_); 2.29 (s, 2H, C*H*_2_); 2.85 (t, 2H, C*H*_2_, *J* = 7.1 Hz); 3.00 (t, 2H, C*H*_2_, *J* = 7.1 Hz); 6.57 (dd, 1H, aryl, J_1_ = 6.1 Hz, J_2_ = 1.9 Hz); 6.71 (d, 1H, aryl, *J* = 1.8 Hz); 6.97 (d, 2H, aryl, *J* = 8.0 Hz); 7.37 (d, 1H, aryl, *J* = 8.0 Hz); 7.54 (d, 1H, aryl, *J* = 8.1 Hz); ^13^C NMR (CD_3_OD, 100 MHz) δ: 28.9; 30.6; 36.7; 37.1; 49.2; 117.2; 118.4; 122.0, 124.7; 125.7; 127.6; 127.9; 128.8; 140.3; 141.8; 146.3; 172.1. ^19^F NMR (CD_3_OD, 376.3 MHz) δ: −63.82 (3F, C*F*_3_); HR-MS *m/z*: calcd for C_21_H_26_F_3_N_2_O, [(M + H)^+^]: 379.1997; found 379.1992.

#### *N*-Hexyl-2,4-dinitrobenzamide (**69**)

2,4-Dinitrobenzoic acid (1.0 equiv) was dissolved in a mixture of DCM/DMF (1:1 v:v), and then 1.2 equivalents of benzotriazol-1-yloxytripyrrolidinophosphonium hexafluorophosphate (PyBOP) and 2.4 equivalents of DIPEA were added. The mixture was stirred under reflux for 30 min. Then, 1.2 equivalents of hexylamine was added and the mixture was allowed to stir for a further 3 h. Upon completion of the reaction, as observed by TLC analysis, the mixture was cooled to room temperature, DCM was added, and the solution was extracted sequentially (3 times per step) with a HCl (2 N) aqueous solution, a saturated solution of NaHCO_3_, and brine. The organic phase was extracted, dried over anhydrous Na_2_SO_4_, filtered, and concentrated in vacuo. The crude product was purified by flash chromatography using mixtures of *n*-hexane/ethyl acetate as solvents. Compound **69** was obtained as a yellowish solid in 77% yield. ^1^H NMR (CDCl_3_, 400 MHz) δ: 0.92 (t, 3H, C*H*_3_, *J* = 6.2 Hz); 1.25–1.34 (m, 6H, 3 C*H*_2_); 1.60–1.65 (m, 2H, C*H*_2_); 3.43 (t, 2H, C*H*_2_, *J* = 9.5 Hz); 6.32 (s, 1H, N*H*); 6.80 (s, 1H, aryl); 7.71 (dd, 1H, aryl, J_1_ = 5.1 Hz, J_2_ = 2.3 Hz); 8.49–8.52 (m, 1H, aryl); 8.85 (d, 1H, aryl, *J* = 10.5 Hz); HR-MS *m/z*: calcd for C_13_H_18_N_3_O_5_, [(M + H)^+^]: 296.1241; found 296.1248.

#### 2,4-Diamino-*N*-hexylbenzamide (**70**)

Compound **70** was prepared starting from intermediate **69** and following general procedure E for catalytic hydrogenation. The compound was isolated upon flash chromatography as a dark-gray solid in 89% yield. ^1^H NMR (CDCl_3_, 400 MHz) δ: 0.90 (t, 3H, C*H*_3_, *J* = 6.5 Hz); 1.32–1.39 (m, 6H, 3 C*H*_2_); 1.54–1.61 (m, 2H, C*H*_2_); 3.36 (dd, 2H, C*H*_2_, *J*_1_ = 6.9 Hz, *J*_2_ = 6.1 Hz); 4.64 (bs, 4H, 2 N*H*_2_); 5.91–5.99 (m, 3H, aryl and N*H*); 7.13 (d, 1H, aryl, *J* = 8.4 Hz); HR-MS *m/z*: calcd for C_13_H_22_N_3_O, [(M + H)^+^]: 236.1757; found 236.1761.

#### 2-Amino-*N*-hexyl-4-((4-(trifluoromethyl)benzyl)amino)benzamide (**71**)

Final product **71** was obtained by the reaction of **70** with 4-(trifluoromethyl)benzyl bromide, following the general procedure C (yield 69%). ^1^H NMR (CDCl_3_, 400 MHz) δ: 0.92 (t, 3H, C*H*_3_, *J* = 6.7 Hz); 1.34–1.41 (m, 6H, 3 C*H*_2_); 1.57–1.63 (m, 2H, C*H*_2_); 3.39 (dd, 2H, C*H*_2_, *J*_1_ = 6.8 Hz, *J*_2_ = 6.1 Hz); 3.82 (m, 2H, N*H*_2_); 4.44 (s, 2H, C*H*_2_); 5.76 (bs, 1H, N*H*); 5.93–5.96 (m, 2H, aryl); 7.20 (d, 1H, aryl, *J* = 8.4 Hz); 7.49 (d, 2H, aryl, *J* = 7.9 Hz); 7.59 (d, 2H, aryl, *J* = 7.9 Hz); 8.60 (bs, 1H, N*H*); ^13^C NMR (CDCl_3_, 100 MHz) δ: 14.3; 22.6; 22.7; 29.8; 31.6; 39.6; 46.7; 96.6; 102.9; 106.4; 125.49; 125.52; 127.2; 129.0; 143.7; 150.6; 151.3; 169.7 ^19^F NMR (CDCl_3_, 376.3 MHz) δ: −62.38 (3F, C*F*_3_); HR-MS *m/z*: calcd for C_21_H_27_F_3_N_3_O, [(M + H)^+^]: 394.2101; found 394.2098.

### Photostability and Pharmacokinetic Experiments

#### Instrumentation

UHPLC analyses were performed using a Shimadzu Nexera (Shimadzu, Milan, Italy) UHPLC consisting of two LC 30 AD pumps, a SIL 30 AC autosampler, a CTO 20 AC column oven, and a CBM 20A controller. For UHPLC–MS/MS analysis, the above described system was coupled online to a triple quadrupole LCMS 8050 (Shimadzu, Kyoto, Japan) equipped with an electrospray ionization (ESI) source.

#### Sample Treatment

Photostability experiments were performed by dissolving compounds in DMSO and then diluting in buffered aqueous solutions at pH 7.4 to a final concentration of 10 μM. 1 cm quartz cells, filled with the abovementioned solutions, were irradiated by a UV lamp (UV Consulting TQ 150 equipped with Duran 50 sleeve and 150 W power supply unit, Peschl, Germany) at a fixed distance of 20 cm from the UV source. Control samples were maintained at 37 °C, wrapped with aluminum foil to avoid light exposure, and used to assess chemical stability of the compounds. At predetermined intervals, aliquots were withdrawn and analyzed by UHPLC in order to assess the concentration decrease of the starting materials and the presence of the dimers usually formed by retigabine.

To measure the brain/plasma ratio, male C57Bl/6 mice (Charles River Laboratories, Italy) were i.p. injected with the vehicle, compound **60** (1 mg/hg), or retigabine (1 mg/kg) (see above in the Drugs section). The animals were sacrificed after 60 min, and brain and blood were collected.

For the assessment of plasmatic concentration, 20 μL of plasma was treated with 100 μL of methanol in a test tube. The tube was vortexed for 30 s. Then, the sample was centrifuged for 10 min at 14680 rpm, at 4 °C. 90 μL of supernatant was dried under nitrogen, reconstituted in 150 μL of MeOH, and then injected into the UHPLC–MS/MS.

Frozen brain tissues were lyophilized overnight, then weighed, carefully homogenized, and extracted with methanol in a 10 mg:0.400 μL tissue:solvent ratio. The samples were vortexed for 30 s, treated in an ultrasonic bath for 5 min, and then centrifuged for 10 min at 14680 rpm, at 4 °C. The supernatants were dried under nitrogen, reconstituted in 1 mL of methanol, filtered by 0.45 μM RC-membrane filters, and then injected into the UHPLC–MS/MS.

To assess the drug AUC and half-life, rats (Sprague–Dawley) were used to allow multiple blood sampling at 0, 0.5, 2, 4, 8, and 24 h. The drug half-lives were calculated using GraphPad Prism 8.0.2 (GraphPad Software, LaJolla, CA).

#### UHPLC Conditions

Photostability and chemical stability experiments were carried out on a Kinetex^TM^ C18 150 × 2.1 mm × 2.6 μm (100 Å) column (Phenomenex, Bologna, Italy). The optimal mobile phase consisted of 0.1% TFA/H_2_O v/v (A) and 0.1% TFA/ACN v/v (B). Analysis was performed in gradient elution as follows: 0–13.0 min, 5–65% B; 13–14.0 min, 65–95% B; 14–15.0 min, isocratic to 95% B; 15–15.01 min, 95–5% B; then 3 min for column re-equilibration. The flow rate was 0.5 mL min^–1^. The column oven temperature was set to 45 °C. The injection volume was 7 μL of sample. The following PDA parameters were applied: sampling rate, 12.5 Hz; detector time constant, 0.160 s; cell temperature, 40 °C. Data acquisition was set in the range 190–800 nm, and chromatograms were monitored at 224 nm to assess the decrease in concentration of the starting material, while a wavelength of 550 nm was used to eventually detect dimerization.

UHPLC–MS/MS pharmacokinetic analyses were performed on a Luna Omega Polar 50 × 2.1 mm, 1.6 μm (100 Å, Phenomenex) employing as mobile phases the following: (A) 0.1% HCOOH in H_2_O (v/v) and (B) 0.1% HCOOH in ACN (v/v), with the following gradient: 0 min, 40% B, 0.01–2.00 min, 40–100% B, isocratic for 1.50 min. Returning to 40% B in 0.10 min. The flow rate was set at 0.5 mL/min. The column oven was set at 45 °C, and 5 μL of extract was injected. All additives and mobile phases were LCMS-grade and purchased from Merck (Milan, Italy).

The ESI was operated in positive mode. MS/MS analyses were conducted in scheduled multiple reaction monitoring (MRM), employing as transitions for retigabine: 303.9 > 109.10 (quantifier ion), 303.9 > 230.10 (qualifier ion); for compound **60**: 397.9 > 300.15 (quantifier ion), compound **60**: 397.9 > 140.10 (qualifier ion), dwell time 50 ms. Interface temperature, desolvation line temperature, and heat block temperature were set, respectively, to 250, 250, and 350 °C. Nebulizing, drying (N_2_), and heating gas (air) were set, respectively, to 3, 10, and 10 L/min.

Retigabine and compound **60** were selected as external standards for the quantitation. Stock solutions (1 mg/mL) were prepared in DMSO, and the calibration curve were obtained in methanol in the concentration range of 1–100 ng/mL (R^2^ = 0.999). Retigabine LOD and LOQ were 0.017 ng/mL and 0.058 ng/mL, respectively. Compound **60** LOD was 0.019 ng/mL while LOQ was 0.063 ng/mL.

### In Silico Studies

The EM structure of human KCNQ2 in complex with retigabine (PDB ID: 7CR2)^21^ was downloaded from the Protein Data Bank.^50^ The Kv7.2_216–330_ tetramer structure was processed, as previously reported,^51^ through the Schrodinger Protein Preparation Wizard^52^ and used for the following studies. Ligands were sketched through the Schrödinger Maestro GUI^53^ and processed through the LigPrep utility,^54^ to get low-energy conformations and account for tautomeric and ionization states at a pH range of 6–8. For each ligand, only the state with the lowest state penalty was retained for the docking studies. Molecular docking simulations were carried out using the standard protocol of Schrodinger Induced Fit Docking.^55^ Docking space for the initial Glide^56^ docking was defined as a 900 Å^3^ cubic box centered on the experimental bound pose of retigabine, and the ligand diameter midpoint was required to stay inside a smaller, nested 400 Å^**3**^ box. Residues within 5 Å from the experimental bound retigabine structure were mutated to alanine; ligand and receptor nonpolar atom vdW radii were scaled by 0.70 and 0.50 factors, respectively. A maximum number of 20 poses per ligand was advanced to the Prime refinement stage, where all residues within 5 Å from ligand poses were optimized. For the refinement stage, an implicit membrane, based on OPM^57^ entry 7CR2, was set. Final docking was carried out using Glide XP with default settings. For each ligand, only the best scoring complex was retrieved and used to set MD simulations up.

MD simulated environments were set up using the Desmond^58,59^ system builder utility; complexes were inserted into a POPC bilayer, based on the 7CR2 entry from the OPM database. Solvation was treated explicitly using the TIP3P water model.^60^ The systems were neutralized by Na^+^ and Cl^–^, which were added to a final concentration of 0.15 M. Prior to MD production stage, Membrane/ligand/protein systems were equilibrated by means of the standard equilibration stepwise protocol for membrane proteins distributed with Desmond. After system equilibration, 120 ns-long MD simulations were carried out at 300 K in the NpγT ensemble, using a Nosé–Hoover chain thermostat and a Martyna–Tobias–Klein barostat. Backbone heavy atoms were constrained during the production stage (1 kcal/mol). Time steps were set to 2, 2, and 6 fs for bonded, near, and far interactions, respectively. MD trajectories were analyzed using VMD^61^ and the Simulation Interaction Diagram embedded in the Schrodinger Suite. OPLS2005^62^ was used as the force field in all of the simulations.

### Generation of Stable Cell Lines and Fluorescence-Based Assay

#### Transfection

The Kv7.2/Kv7.3-stable cell line was generated using a plasmid-based PiggyBac transposon system that enables genomic integration of multiple independent transposons containing distinct cDNA sequences. cDNAs for KCNQ2 and KCNQ3 genes (encoding for Kv7.2 and Kv7.3, respectively) were cloned into two different PiggyBac expression vectors (pB-CMV-MCS-EF1α-RedPuro, System Biosciences), and these two plasmids, together with a plasmid encoding for the transposase (System Biosciences), were transfected into CHO cells using Lipofectamine 2000 (Invitrogen) as indicated in the protocol provided by the seller company.

#### Clone Selection and Characterization

An initial screening of the clones was based on two selection markers: puromycin resistance and red fluorescence. Puromycin (Sigma) at concentration of 4 μg/mL was added to the medium 24 h after transfection. After 6 days in the plate, clones were serially diluted into a 96-well. After a week, cells showing the strongest red fluorescence were selected, trypsinized (trypsin 0.25%, Gibco), and plated into Petri dishes, obtaining seven different clones. Cells were grown in DMEM supplemented with 10% FBS, 50 U/mL Pen-Strep, 2 mM l-glutamine (all purchased from Gibco), and 4 μg/mL puromycin (Sigma) in a humidified atmosphere at 37 °C with 5% CO_2_.

A subsequent screening was made using the electrophysiological patch clamp technique to identify a clone exhibiting biophysical properties similar to those showed by CHO cells transiently transfected with the cDNAs encoding for Kv7.2 and Kv7.3.

Transiently transfected cells generated a voltage-dependent K^+^ selective current with a current density of 119.7 ± 13.2 pA/pF and a half-activation potential (*V*_1/2_) of −35.1 ± 1.6. Among seven clones, clone number 5, showing a *V*_1/2_ of −32.2 ± 1.7 (*p* < 0.05) and current density at 0 mV of 71.1 ± 17.4 pA/pF (*p* < 0.05), was selected (Figure S98A,B).

To ensure that both Kv7.2 and Kv7.3 subunits were expressed into the selected clone and formed heteromeric channels, pharmacological experiments with the Kv7 channel blocker tetraethylammonium (TEA) were performed.^64^ These experiments are based on differential TEA sensitivity of homomeric Kv7.2 and Kv7.3 channels and heteromeric Kv7.2/Kv7.3 channels: Kv7.2 is highly sensitive to TEA (IC_50_ = 0.3 mM), Kv7.3 is TEA-insensitive (IC_50_ = 30 mM), and the heteromeric Kv7.2/Kv7.3 channel shows an intermediate sensitivity (IC_50_ = 3 mM).^18^ The effect of TEA blockade on Kv7.2/Kv7.3 currents on the selected clone was compared to that of the blockade exerted on the control group, represented by CHO cells transiently transfected with Kv7.2/Kv7.3 channels. The effect of the TEA blockade was investigated using a ramp protocol in which Kv7.2/Kv7.3 currents were activated by 3 s voltage ramps from −80 to +20 mV. Perfusion with 3 mM TEA in the control group induced a current inhibition of about 55% while in the selected clone, the inhibition was about 35%, suggesting that both Kv7.2 and Kv7.3 subunits are likely expressed, possibly with a slightly higher participation of Kv7.3 subunits (Figure S98C).

#### Fluorescence-Based Assay

The selected clone stably expressing Kv7.2/Kv7.3 channels was grown in DMEM supplemented with 10% FBS, 50 U/mL Pen-Strep, 2 mM l-glutamine (all purchased from Gibco), and 4 μg/mL puromycin (Sigma) in a humidified atmosphere at 37 °C with 5% CO_2_. To perform the FluxOR II Green Potassium Ion Channel Assay (Invitrogen), cells were seeded in a 96-well Biocoat Poly-D-Lysine Cellware White/Clear Plate (Corning) at a density of 1.6 × 10^4^ cells/well in 80 μL of medium. 24 h after seeding, the FluxOR assay was performed, following the “Wash method” as described in the manufacturer’s protocol. A kinetic dispense microplate reader (FLUOstar Optima) was used to read the plate, setting the excitation filter at 485 nm and the emission filter at 520 nm; “Stimulus buffer” prepared as indicated in the protocol was automatically added to wells after 5 s of recording; the plate was read every 1 s, for 50 s. The results were analyzed using Optima Data Analysis and Microsoft Excel.

### Electrophysiological Experiments

#### Cell Culture and Transient Transfection

Channel subunits were expressed in Chinese hamster ovary (CHO) cells by transient transfection, using plasmids containing cDNAs encoding human Kv7.2 and/or Kv7.3, all cloned in the pcDNA3.1 vector. According to the experimental protocol, these plasmids were expressed individually or in combination, together with a plasmid-expressing enhanced green fluorescent protein (Clontech, Palo Alto, CA) used as a transfection marker. Total cDNA in the transfection mixture was kept at 4 μg. CHO cells were grown in 100 mm plastic Petri dishes in Dulbecco’s modified Eagle’s medium containing 10% fetal bovine serum, nonessential amino acids (0.1 mM), penicillin (50 U/mL), and streptomycin (50 mg/mL) in a humidified atmosphere at 37 °C with 5% CO_2_. 24 h before transfection, the cells were plated on glass coverslips coated with poly-l-lysine and were transfected on the next day with the appropriate cDNA using Lipofectamine 2000 (Life Technologies), according to the manufacturer’s protocol. Electrophysiologic experiments were performed 24 h after transfection.

#### Whole-Cell Electrophysiology

Currents from CHO cells were recorded at room temperature (20–22 °C) using the whole-cell configuration of the patch-clamp technique, with glass micropipettes of 3–5 MΩ resistance. During the recording, constant perfusion of extracellular solution was maintained. The extracellular solution contained (in mM): 138 NaCl, 2 CaCl_2_, 5.4 KCl, 1 MgCl_2_, 10 glucose, and 10 HEPES, at pH 7.4, with NaOH. The pipette (intracellular) solution contained (in mM): 140 KCl, 2 MgCl_2_, 10 EGTA, 10 HEPES, 5 Mg^2+^-ATP, at pH 7.3–7.4, with KOH. Current was recorded using an Axopatch-200A amplifier, filtered at 5 kHz, and digitized using a DigiData 1440A (Molecular Devices). The pCLAMP software (version 10.2) was used for data acquisition and analysis (Molecular Devices). To evaluate the activity of each compound on Kv7.2 + Kv7.3 currents, the cells were clamped at −80 mV and conductance–voltage curves (*G*/*V* curves; activation curves) were generated by normalizing to the maximal value of the instantaneous isopotential currents at 0 mV and expressing the normalized values as a function of the preceding voltages. Data were fit to a Boltzmann distribution of the following form: *y* = *max*/[1 + exp (*V*_1/2_ – *V*)/*k*], where *V* is the test potential, *V*_1/2_ is the half-activation potential, and *k* is the slope factor.

The Δ*V*_1/2_ values, calculated as the difference between the *V*_1/2_ for controls and after drug exposure, were plotted versus log(concentration) of the compound, fitted to a four parameter logistic equation, and EC_50_ values were calculated with SigmaPlot (version 12.3). Indicated EC_50_ values are the mean of at least three independent experiments ± standard error of the mean (SEM).

### *In Vivo* Experiments

#### Animals

Male C57Bl/6 mice (Charles River Laboratories, Italy) arrived in the animal facility at 21 days of age, and they were housed in groups of three per cage under controlled conditions (temperature 21 ± 1 °C, 60 ± 10% relative humidity and 12/12 h light cycle with lights on at 07:00 a.m.). Food and water were available ad libitum. Animals were experimentally naive and were used only once. Sample size (*n*) is indicated in the figure legends. The experiments were approved by the Italian Ministry of Health (n. 246/2019-PR) and performed in agreement with the ARRIVE (Animals in Research: Reporting In Vivo Experiments) guidelines,^64^ with the guidelines released by the Italian Ministry of Health (D.L. 26/14) and the European Community Directive 2010/63/EU.

#### Drugs

Retigabine (Valeant), compound **60**, and XE-991 (Tocris) were dissolved in saline containing 2% Tween-20 and 2% PEG-400. Retigabine was administered in doses of 1 and 3 mg/kg at concentrations of 0.1 and 0.3 mg/mL; compound **60** was administered in doses of 0.1, 0.3, and 1 mg/kg at concentrations of 0.01, 0.03, and 0.1 mg/mL, and XE-991 was administered in a dose of 3 mg/kg at concentrations of 0.3 mg/mL. Thus, each dose was dissolved to allow injection of 0.01 mL/g, i.p. The control group was represented by mice injected only with vehicle solution (saline containing 2% Tween-20 and 2% PEG-400). Retigabine, compound **60**, and vehicle solution were administered 30 min prior to induction of seizures based on pharmacokinetics data published in a previous paper of retigabine efficacy against pentylenetetrazol (PTZ) induced seizures;^41^ XE-991 was administered 15 min before the retigabine or compound **60** or vehicle injection.

#### Seizure Testing

PTZ (P6500-25G, Sigma, USA) (100 mg/kg, s.c.) was dissolved in saline and administered in doses of 10 mg/mL; thus, it was dissolved to allow injection of 0.01 mL/g, s.c. Animals were removed from their home cage, weighed, numbered, and treated with vehicle, retigabine, or compound **60** 30 min prior to PTZ administration. PTZ was injected, and animals were placed in clear Plexiglass boxes for observation of seizure activity. The severity of convulsions (from minimal “clonic” to maximal “generalized tonic–clonic”) as well as the latency to onset of maximal seizure was recorded. Animals were observed for 30 min following PTZ injection. The experiment was repeated upon pretreatment with XE-991 15 min before vehicle, retigabine, or compound **60** injection.

#### Seizure Scoring

Seizures were scored using a 9-point scoring system modified from Lüttjohann’s scale (Lüttjohann et al., 2009). 0 = whisker trembling, 1 = sudden behavioral arrest, 2 = facial jerking, 3 = neck jerks, 4 = clonic seizures (sitting), 5 = tonic clonic seizures (lying on belly), 6 = clonic seizures (lying on side), 7 = tonic clonic seizures (lying on side), 8 = wild jumping. The behavioral assessments described above were performed in a blind manner and the observers had to reach a unanimous agreement regarding the scoring of the behavior.

### Statistical Analysis

#### Whole-Cell Electrophysiology

Statistically significant differences in electrophysiological data were evaluated with the Student *t* test or with ANOVA followed by the Student–Newman–Keuls test when multiple groups were compared, with the threshold set at *p* < 0.05. Data were analyzed using the SigmaPlot 12.3 for Windows (Systat Software Inc, San Jose, CA). Values are expressed as the mean ± SD of at least three cells recorded in at least two independent transfections as the mean ± standard error of the mean (SEM) of at least three cells recorded in at least three independent transfections.

#### Fluorescence-Based Assay

Assay robustness was determined according to the *Z*′ factor.^27^
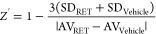


SD is the standard deviation of triplicate in a single experiment, AV is the average of triplicate in a single experiment, RET means retigabine at a concentration of 10 μM; Vehicle means the assay buffer prepared as indicated in the FluxOR protocol +0.1% DMSO.

Only experiments resulting in a *Z*′ > 0.5 were considered. Data shown (Figures 1D,E,F,G and 3B) were obtained from at least three independent experiments. The slope of the fluorescence curves was calculated from a point at second 5 to a point at second 15 (as showed in Figure 1C) in Microsoft Excel: Values are expressed as the mean ± SEM.

Data were analyzed using the GraphPad Prism 8.0.2 (GraphPad Software, LaJolla, CA). Statistically significant differences in the initial slope of the curves were evaluated through ordinary one-way ANOVA, and multiple comparisons were corrected with the Tukey test. The threshold of *p* < 0.01 is indicated in figures as asterisks. The slope values of the curves were plotted versus log(concentration) of the compound and fitted to a four-parameter logistic equation, and EC_50_ values were calculated with SigmaPlot (version 12.3). Indicated EC_50_ values are the mean of at least three independent experiments ± standard error of the mean (SEM).

#### In Vivo Experiments

The number of animals needed for the experiment was determined using the power analysis software GPower version 3.1.92. The required minimum sample size was 8 mice per group. Statistical analyses were performed using GraphPad Prism (GraphPad Software, LaJolla, CA) using a one-way analysis of variance with the Tukey post-hoc test. The *P* value of <0.05 was accepted as indicative of a statistically significant difference.

## Conflict of Interest

N.I., A.B., P.C., M.T., C.O., and F.M. have a patent pending for some of the compounds described in this paper (WO2020157126A1). The remaining authors have no conflicts of interest to declare.

## Supporting Information Available

Figures S1–S84: NMR spectra and HPLC traces of synthesized compounds; Table S1: photostability experiment results; Figures S85–S92: HPLC traces of retigabine and representative compounds at 550 nm; Figures S93: Sequence alignment of the S5, S6, and the S5–S6 intervening linker forming the pore domain of the different Kv subunits; Figures S94–S96: ligand interaction diagram of 120 ns-long MD simulations of Kv7.2 in complex with retigabine, **23a**, **24a**, and **42**; Table S2: average distances between ligands retigabine, **23a**, and **24a** and Kv7.2 residues V225, F304, and L312; Table S3: average distances between ligands retigabine and **42** and Kv7.2 residues F240, L272 and F305 ; Figure S96: HPLC chromatograms at different time points showing stability of compound **60** solubilized in saline buffer; Figure S97: retigabine and compound **60** plasma concentration curves; Figure S98: pharmacological characterization of CHO stably expressing kv7.2 + Kv7.3 channels (PDF)
Molecular formula strings (CSV)
apo7CR2_prepared (PDB)
complex **13**-7CR2 (PDB)
complex **14**-7CR2 (PDB)
complex **15**-7CR2 (PDB)
complex **16**-7CR2 (PDB)
complex **17**-7CR2 (PDB)
complex **18**-7CR2 (PDB)
complex **19**-7CR2 (PDB)
complex **23**-7CR2 (PDB)
complex **24**-7CR2 (PDB)

## Supplementary Material

jm2c00911_si_002.csv

jm2c00911_si_003.pdb

jm2c00911_si_004.pdb

jm2c00911_si_005.pdb

jm2c00911_si_006.pdb

jm2c00911_si_007.pdb

jm2c00911_si_008.pdb

jm2c00911_si_009.pdb

jm2c00911_si_010.pdb

jm2c00911_si_011.pdb

jm2c00911_si_012.pdb

## ABBREVIATIONS List

ACN: acetonitrile
ASM: antiseizure medication
CHO: Chinese hamster ovary
CryoEM: cryogenic electron microscopy
DIPEA: *N*,*N*-diisopropylethylamine
EGTA: glycol ether diamine tetraacetic acid
EM: electron microscopy
ESI: electrospray ion source
FBS: fetal bovine serum
HEPES: 4-(2-hydroxyethyl)-1-piperazineethanesulfonic acid
MeOH: methanol
PDA: photodiode array
Pen-Strep: penicillin–streptomycin
Pd/C: palladium over activated carbon
POPC: phosphatidylcholine
P-RET: *N*-propargyl retigabine
PTZ: pentylenetetrazol
PyBop: benzotriazol-1-yloxytripyrrolidinophosphonium hexafluorophosphate
TEA: triethylamine
TIS: triisopropylsilane
