# Supplementary material for: Beyond Retigabine: Design, Synthesis, and Pharmacological Characterization of a Potent and Chemically Stable Neuronal Kv7 Channel Activator with Anticonvulsant Activity
Source: J Med Chem. 2022 Aug 16;65(16):11340–64. doi: 10.1021/acs.jmedchem.2c00911 (PMC9421656; doi:10.1021/acs.jmedchem.2c00911)
Supplement: Supplementary file 1 — jm2c00911_si_001.pdf [file jm2c00911_si_001.pdf]

Supplementary information file

Beyond retigabine: design, synthesis, and pharmacological characterization of a potent and chemically-stable neuronal Kv7 channel activator with anticonvulsant activity

Simona Musella,[‡,#] Lidia Carotenuto,[¶,#], Nunzio Iraci,[Ŧ,#] Giulia Baroli,[¶] Tania Ciaglia,[‡] Piera Nappi,[¶] Manuela Giovanna Basilicata[‡,] , Emanuela Salviati[‡], Vincenzo Barrese,[¶] Vincenzo Vestuto,[‡] Giuseppe Pignataro,[¶] Giacomo Pepe,[‡] Eduardo Sommella,[‡] Veronica Di Sarno,[‡] Michele Manfra,[‖] Pietro Campiglia,[‡] Isabel Gomez Monterrey,[¥] Alessia Bertamino,[‡] Maurizio Taglialatela,[¶,] Carmine Ostacolo, [¥,*] and Francesco Miceli[¶].*

[‡] Department of Pharmacy, University of Salerno, Via G. Paolo II 132, 84084, Fisciano, Salerno, Italy

[¶] Department of Neuroscience, Reproductive Sciences and Dentistry, University Federico II of Naples, Via Pansini, 5, 80131, Naples, Italy

[Ŧ] Department of Chemical, Biological, Pharmaceutical and Environmental Sciences (CHIBIOFARAM), University of Messina, Viale Ferdinando Stagno d'Alcontres 31, 98166, Messina, Italy

[‖] Department of Science, University of Basilicata, Via dell'Ateneo Lucano 10, 85100 Potenza, Italy

[¥] Department of Pharmacy, University Federico II of Naples, Via D. Montesano 49, 80131, Naples, Italy

[*]Corresponding authors (Maurizio Taglialatela: mtaglial@unina.it; Carmine Ostacolo: ostacolo@unina.it)

[#]Equal contribution

Table of content:

1. **NMR spectra and HPLC traces of synthesized compounds...S3-S86**
2. **Table S1.** Photoinduced degradation of retigabine (RET) and its analogues under UV Lighting**…………………………………………………………………….....S87**

   Figures S85-S92: HPLC traces of selected compounds at 550 nm**………………….S88-S95**
3. **Figure S93: Sequence alignment of Kv7 channels ……………………………………………………………………………...S96**

Figure S94: Ligand interaction of Kv7.2 in complex with retigabine, **23a** and **24a**…………………………………………………………………………………..........S97

Table S2. Distances between retigabine, **23a** and **24a** and selected Kv7.2 residues……………………………………………………………………………….…….S98

Figure S95. Ligand interaction diagram of Kv7.2 in complex with **42**…………………………………………………………………………………...S98

Table S3. Average distances between retigabine, 42 and selected Kv7.2 residues………..S98

4. **Figure S96.** HPLC chormatograms of compound 60 solubilized in saline buffer at different time points……………………………………………………………………….......S99
5. **Figure S97.** Plasma concentration-time curves after a single-dose of Retigabine (3mg/Kg) or compound **60** (0,3 mg/Kg) injected i.p in rats………………………………………......S100
6. **Figure S99.** Pharmacological characterization of CHO stably expressing Kv7.2+Kv7.3 channels…………………………………………………………………………...S101

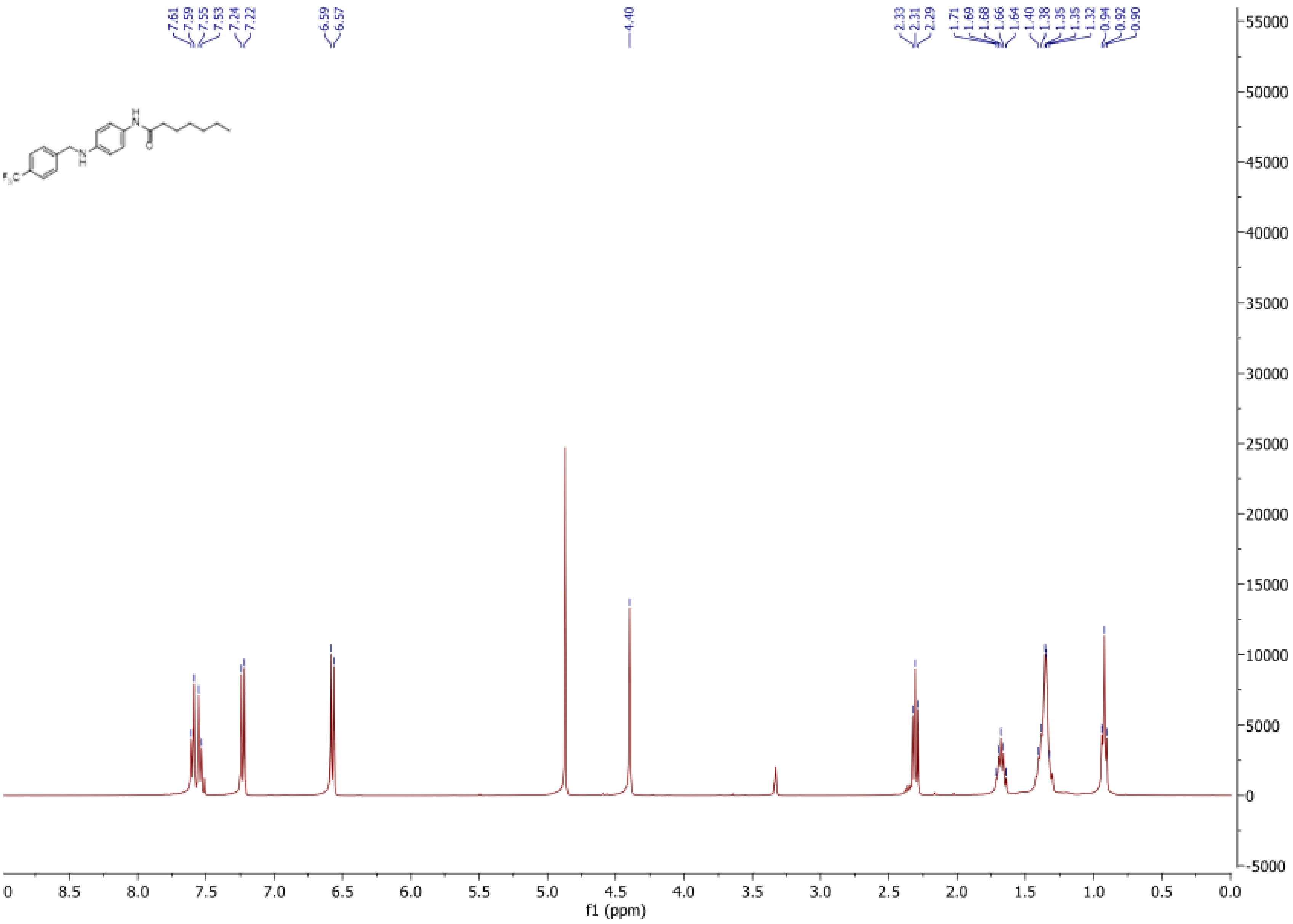

Figure S1: ^{1}H NMR spectra of derivative **2**

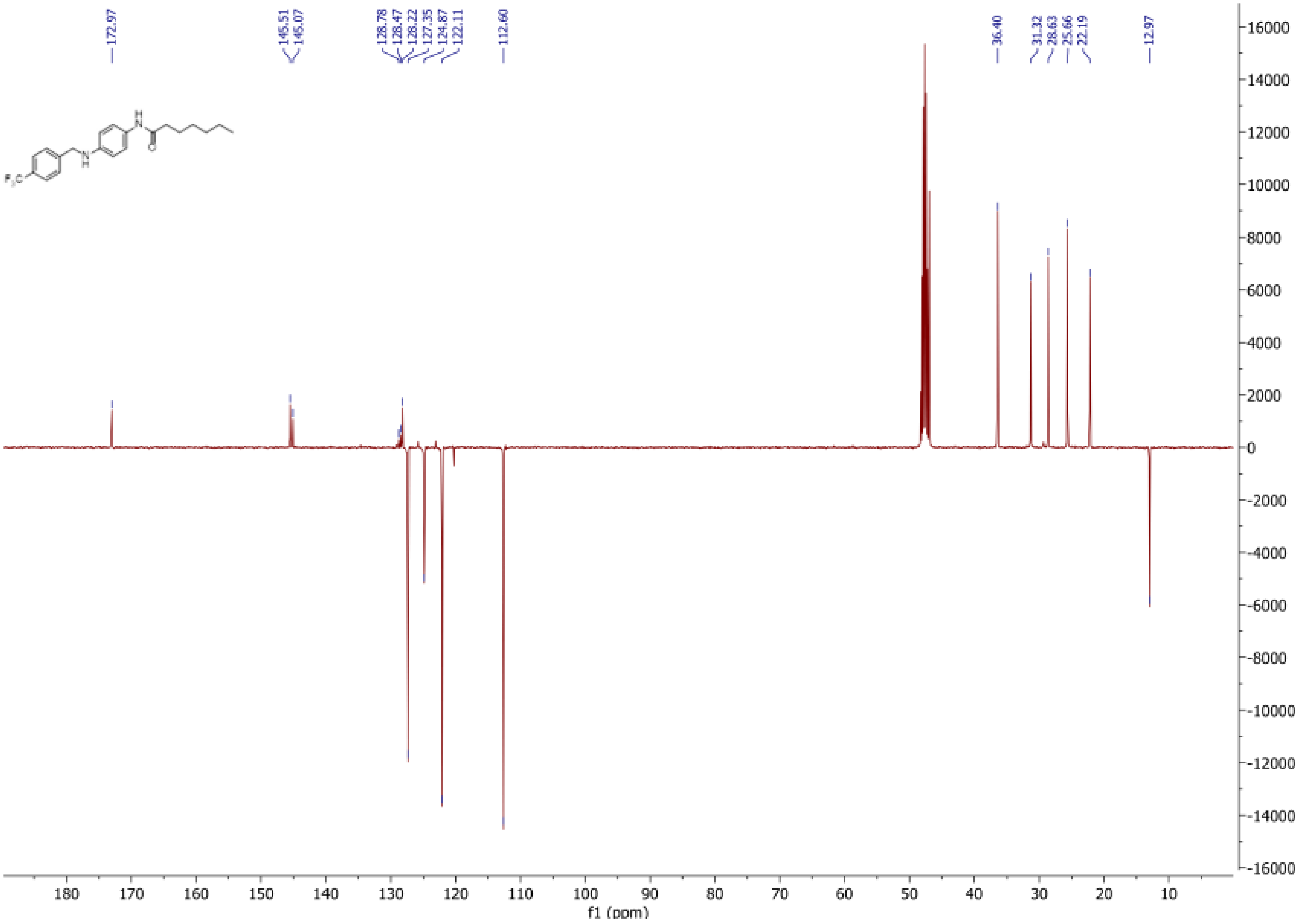

Figure S2: DEPT NMR spectra of derivative **2**

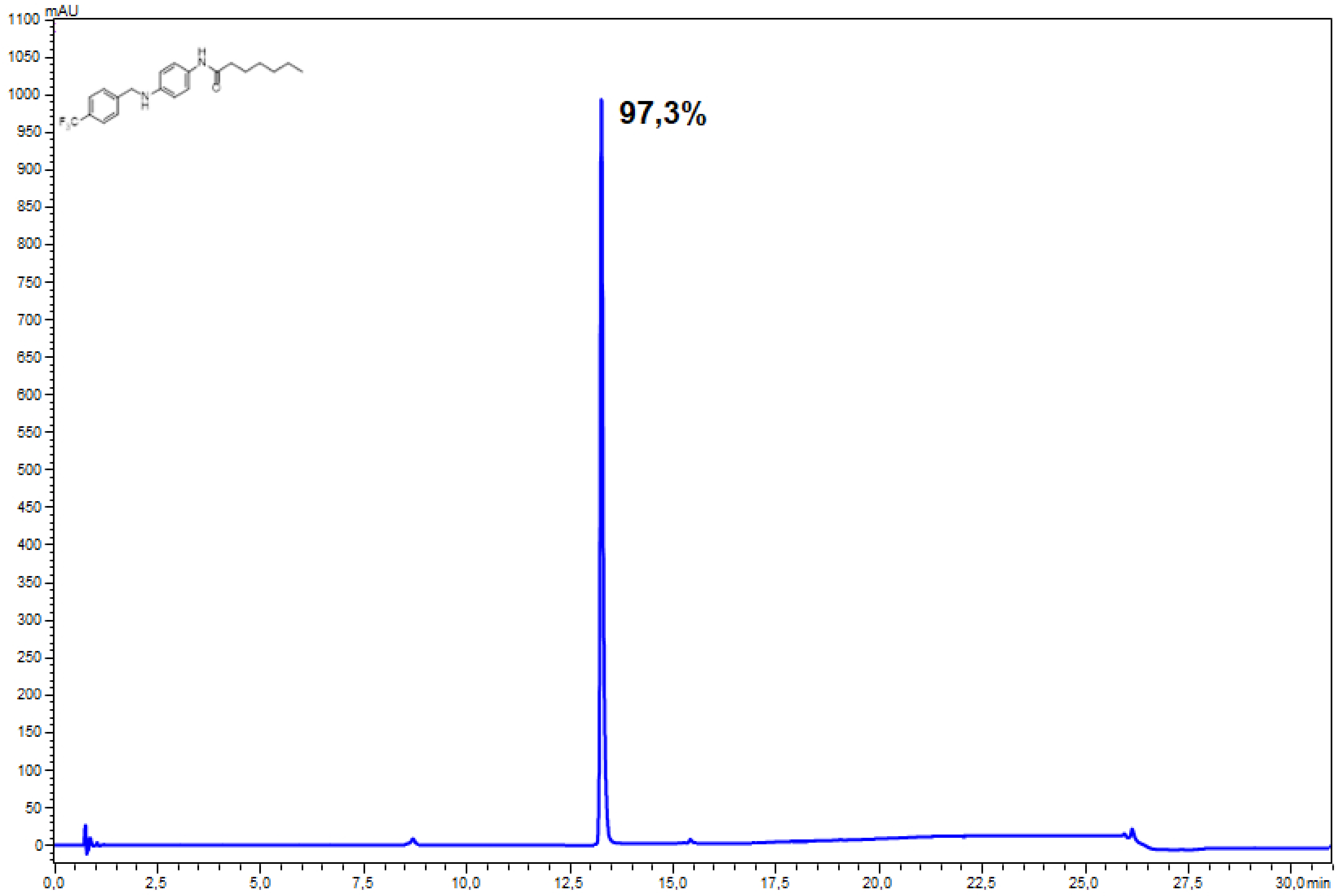

Figure S3: HPLC trace of derivative **2**

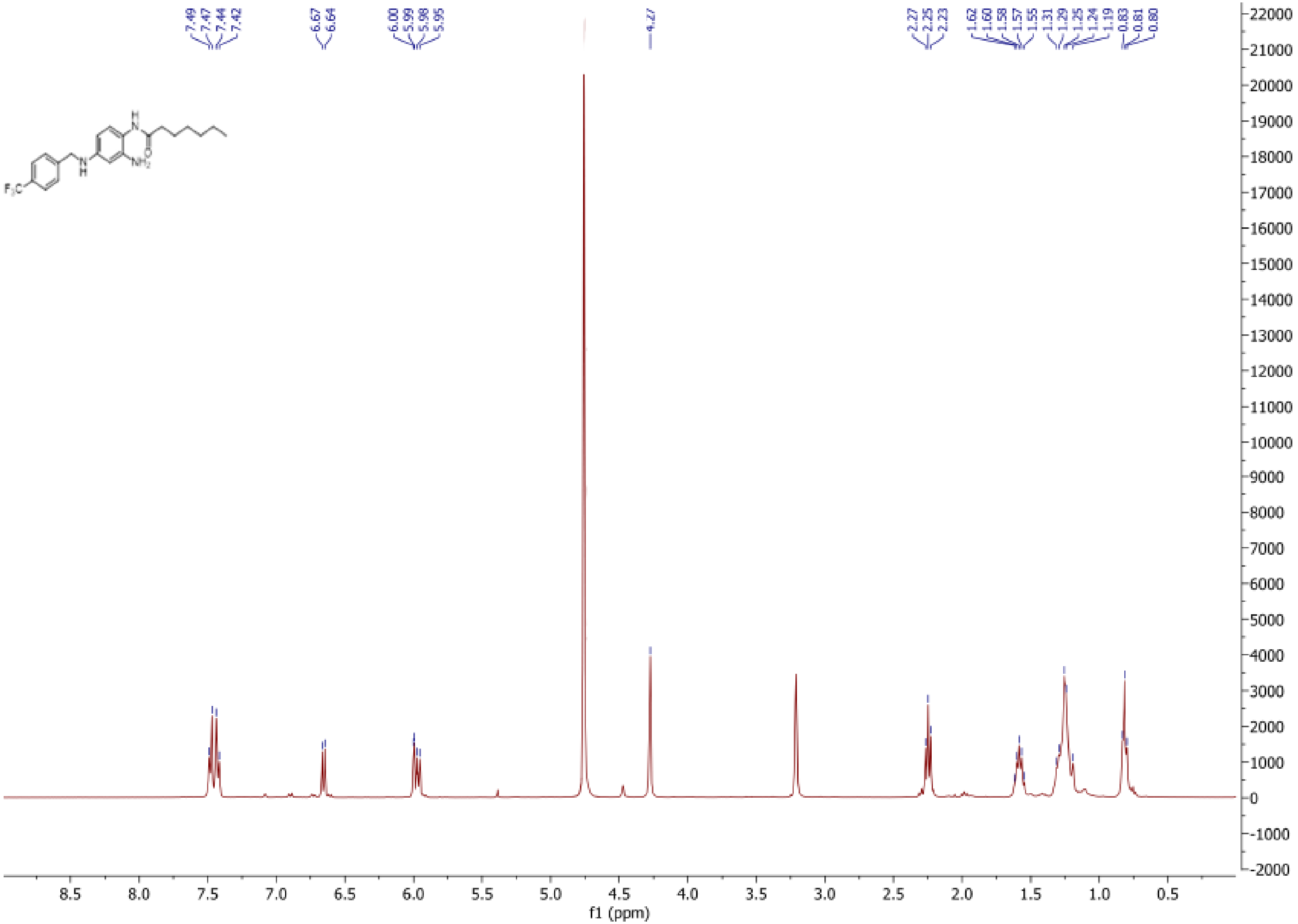

Figure S4: ^{1}H NMR spectra of derivative **13**

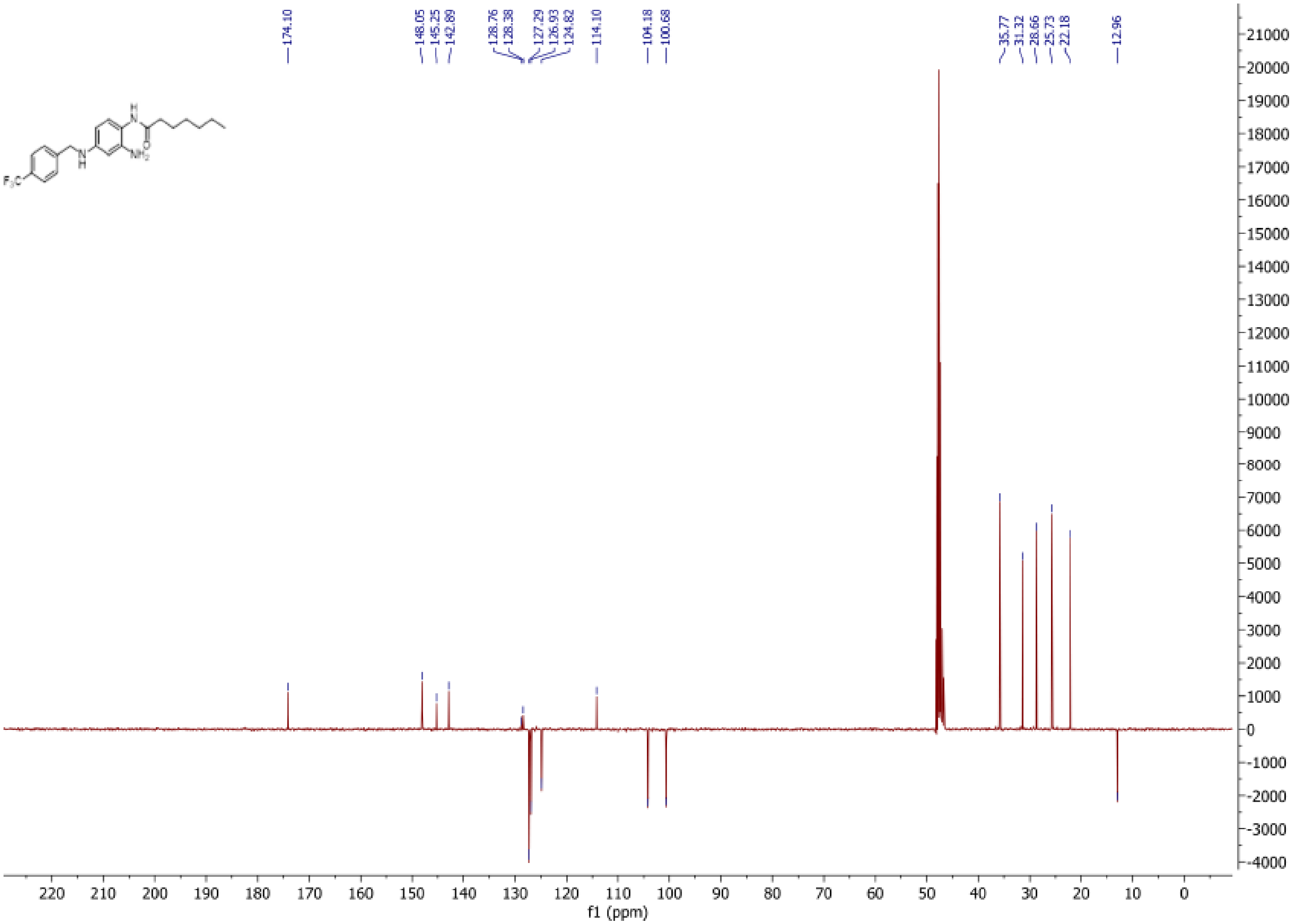

Figure S5: DEPT NMR spectra of derivative **13**

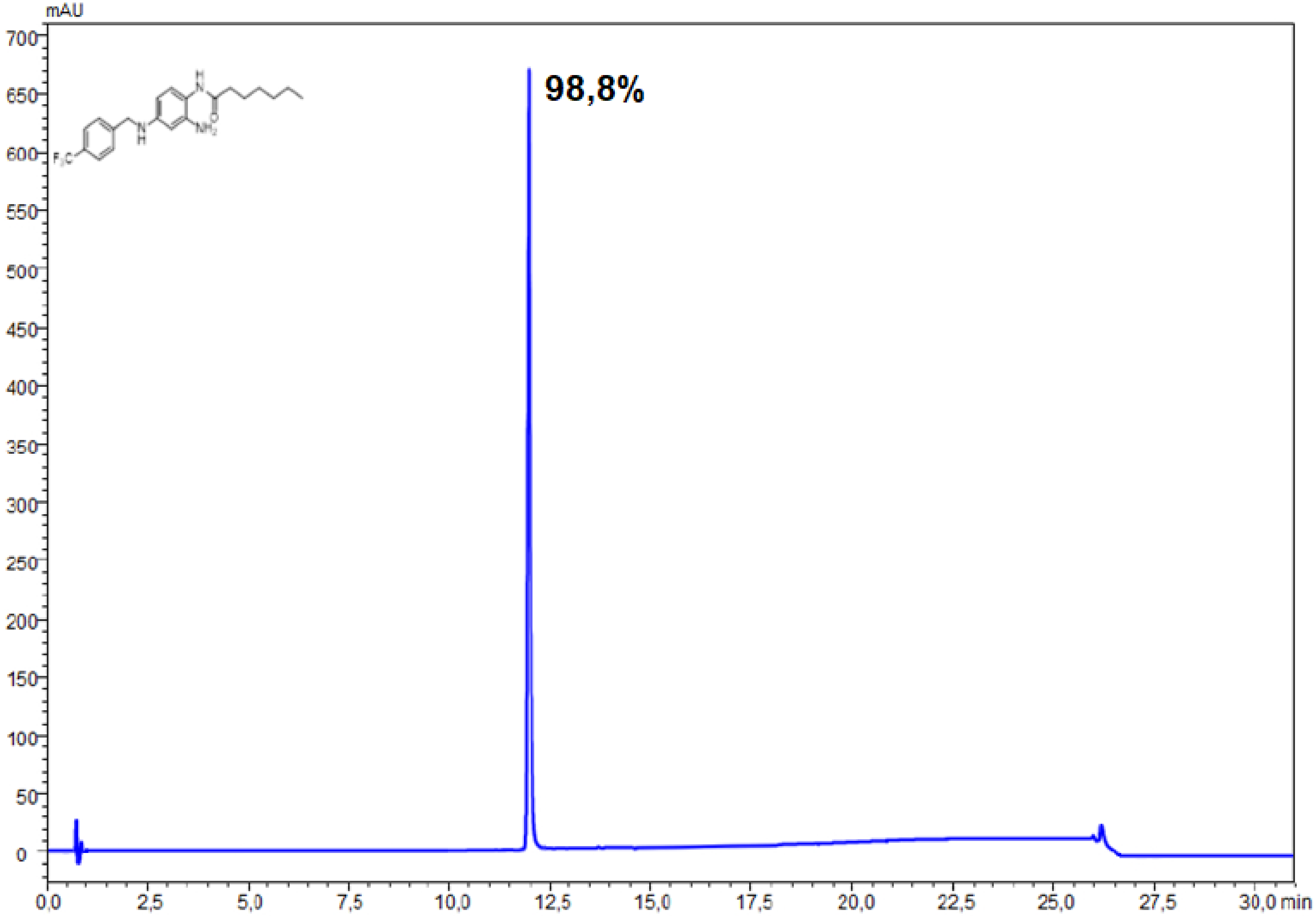

Figure S6: HPLC trace of derivative **13**

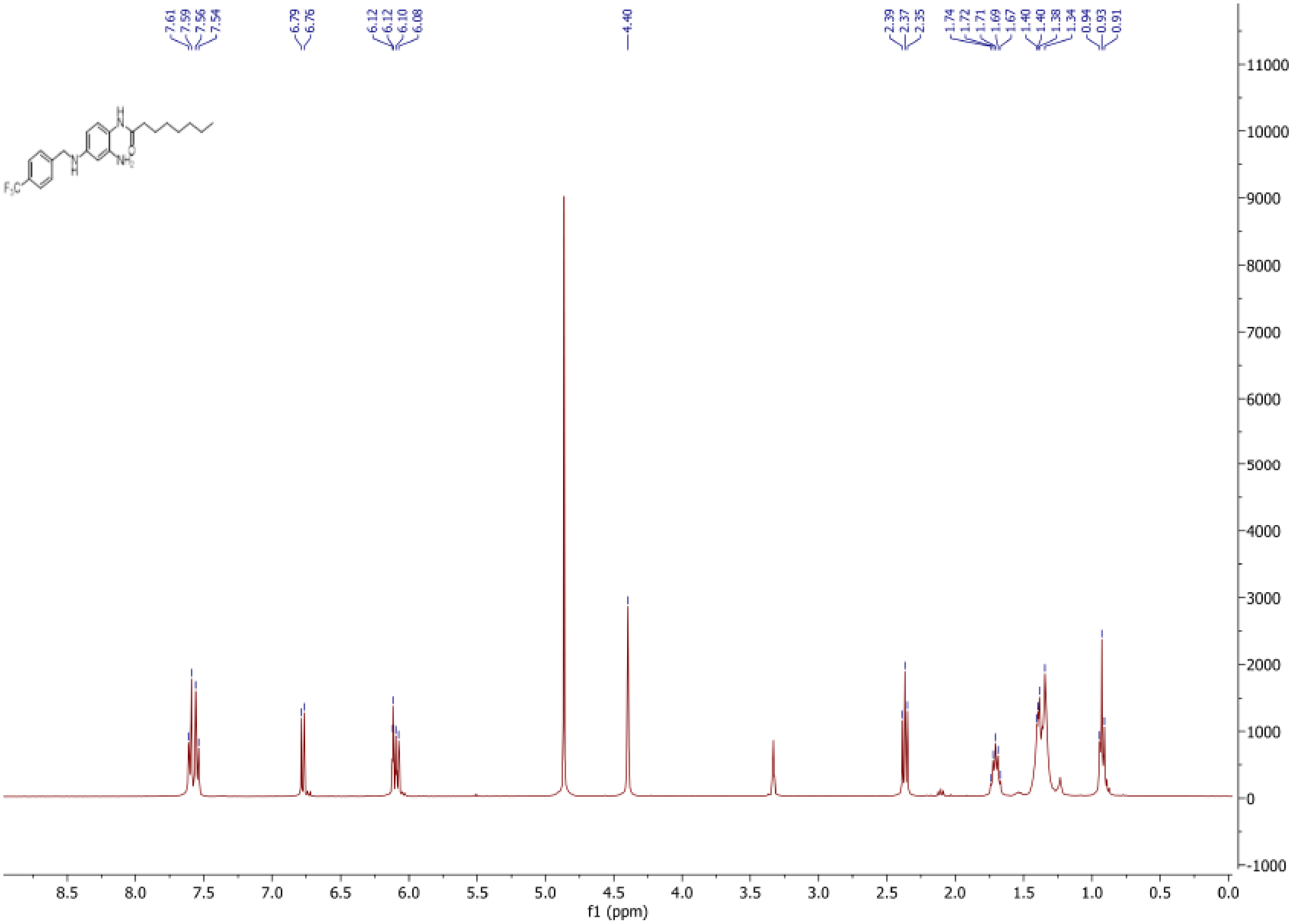

Figure S7: ^{1}H NMR spectra of derivative **14**

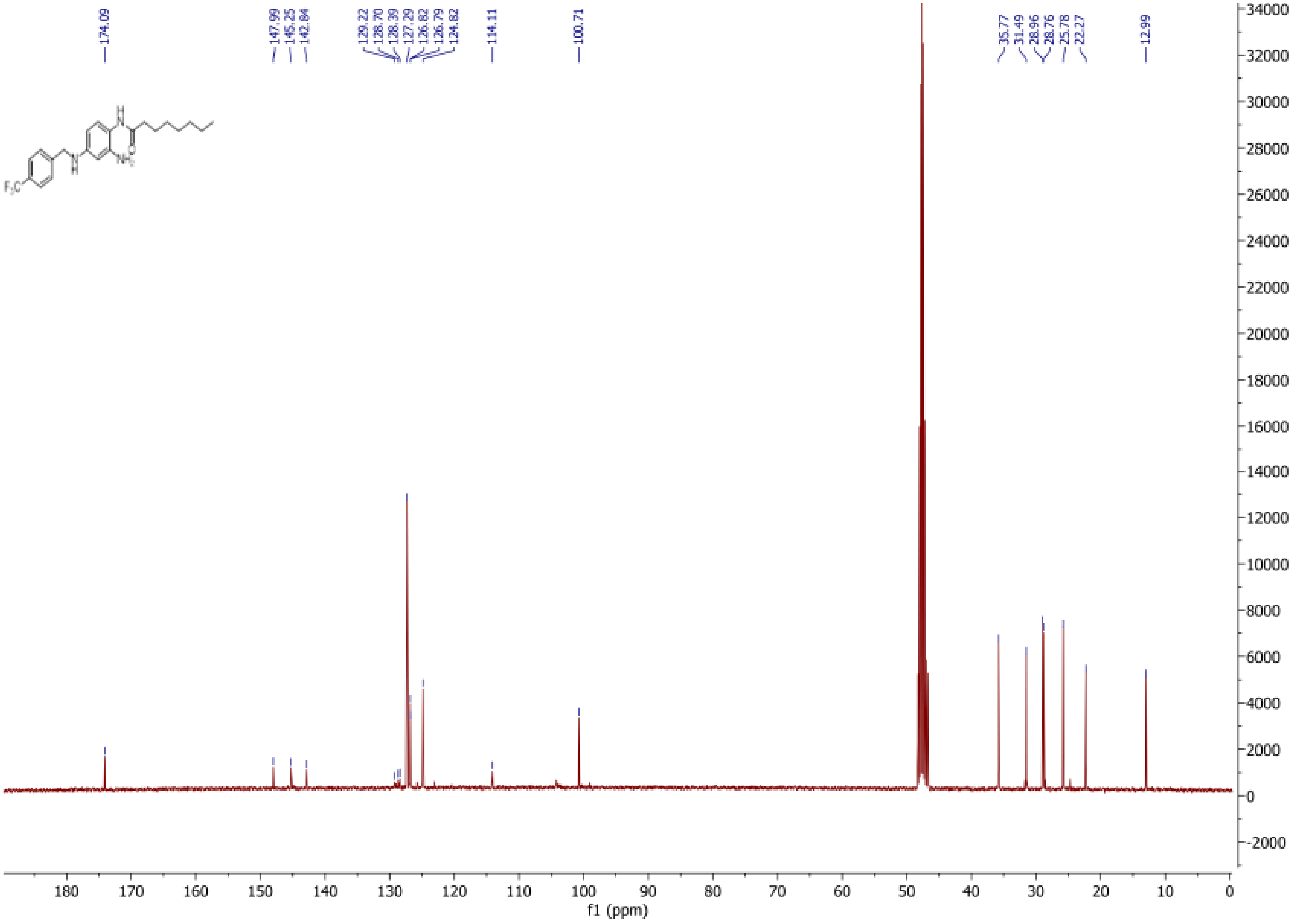

Figure S8: ^{13}C NMR spectra of derivative **14**

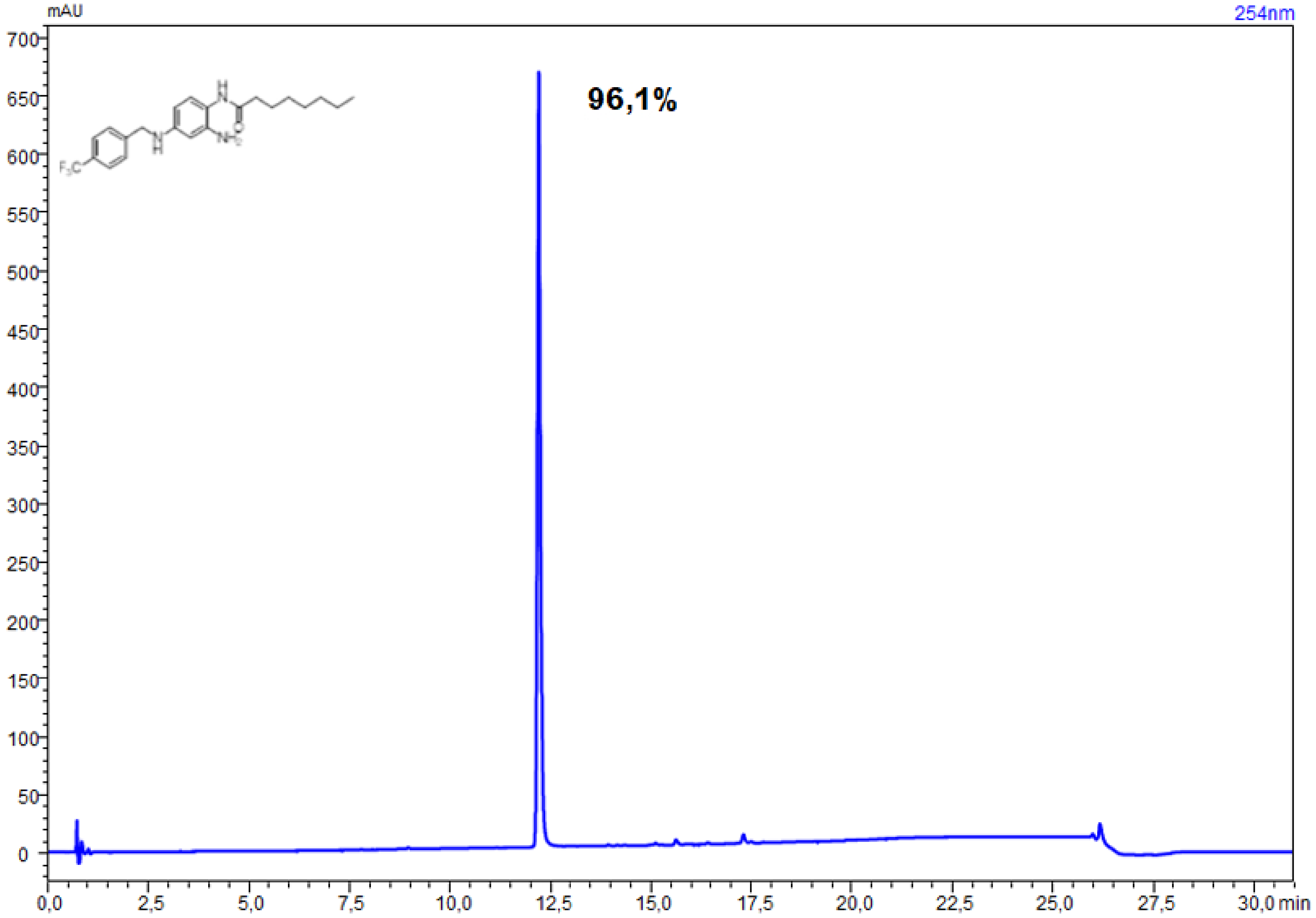

Figure S9: HPLC trace of derivative **14**

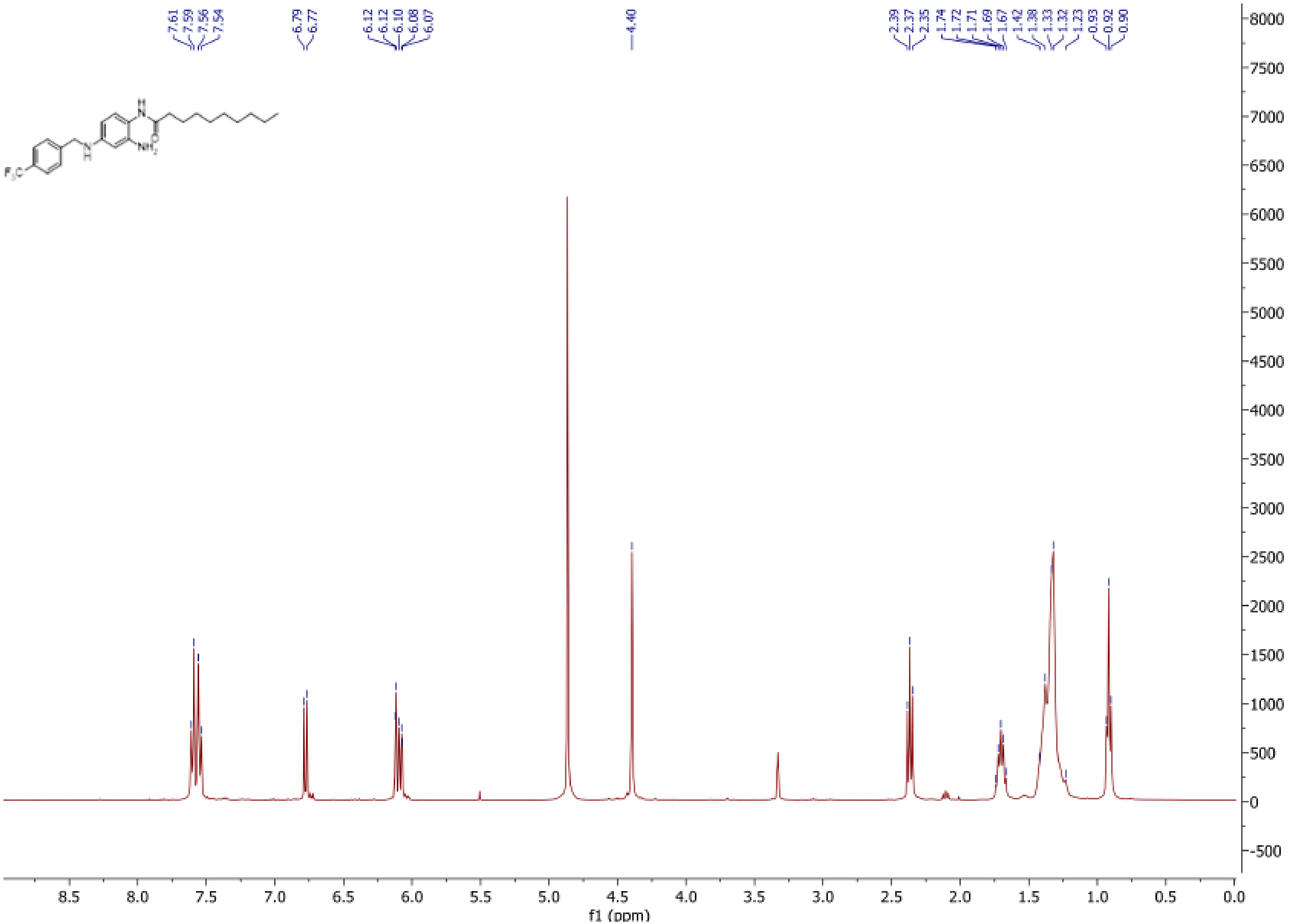

Figure S10: ^{1}H NMR spectra of derivative **15**

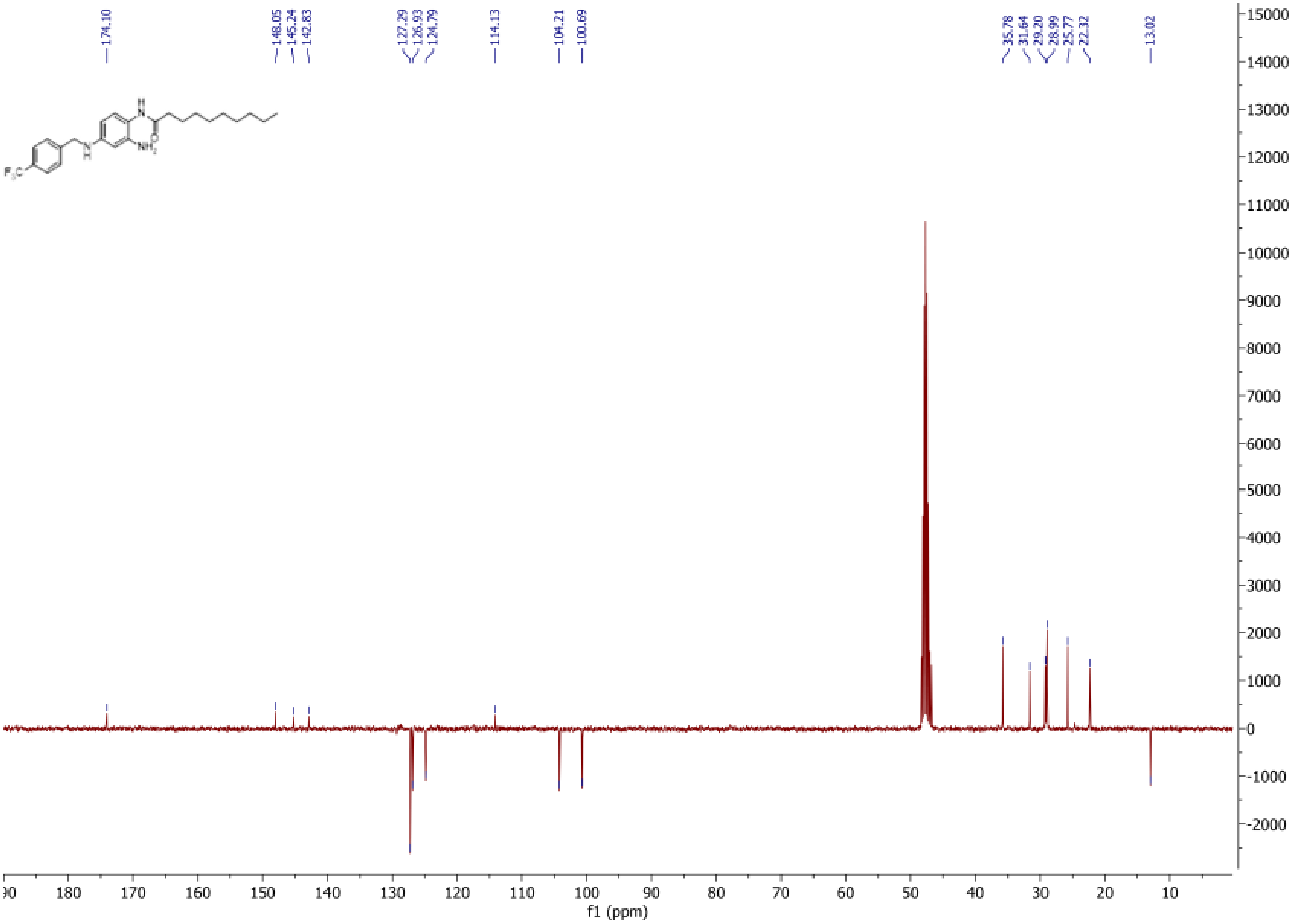

Figure S11: DEPT NMR spectra of derivative **15**

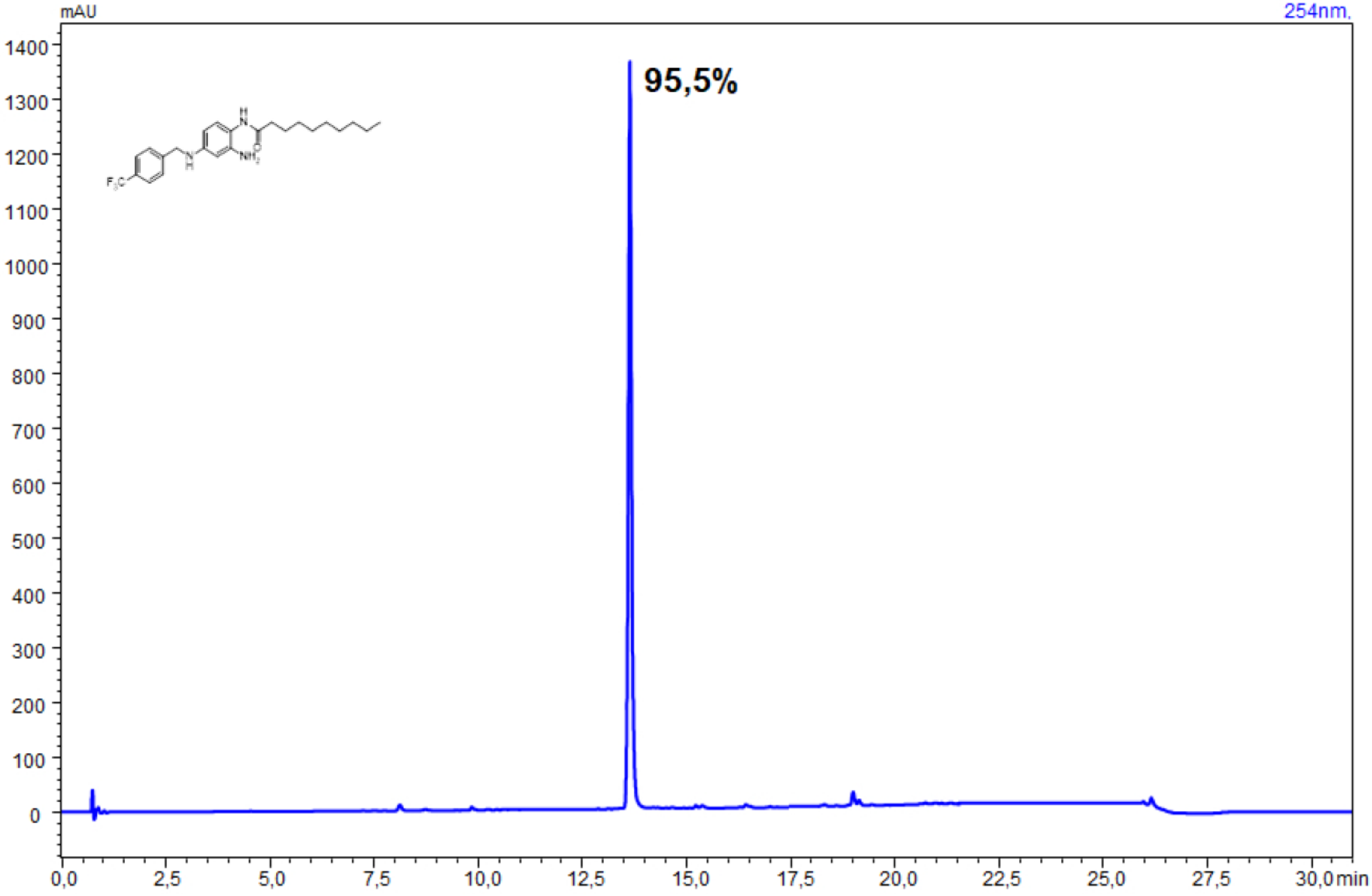

Figure S12: HPLC trace of derivative **15**

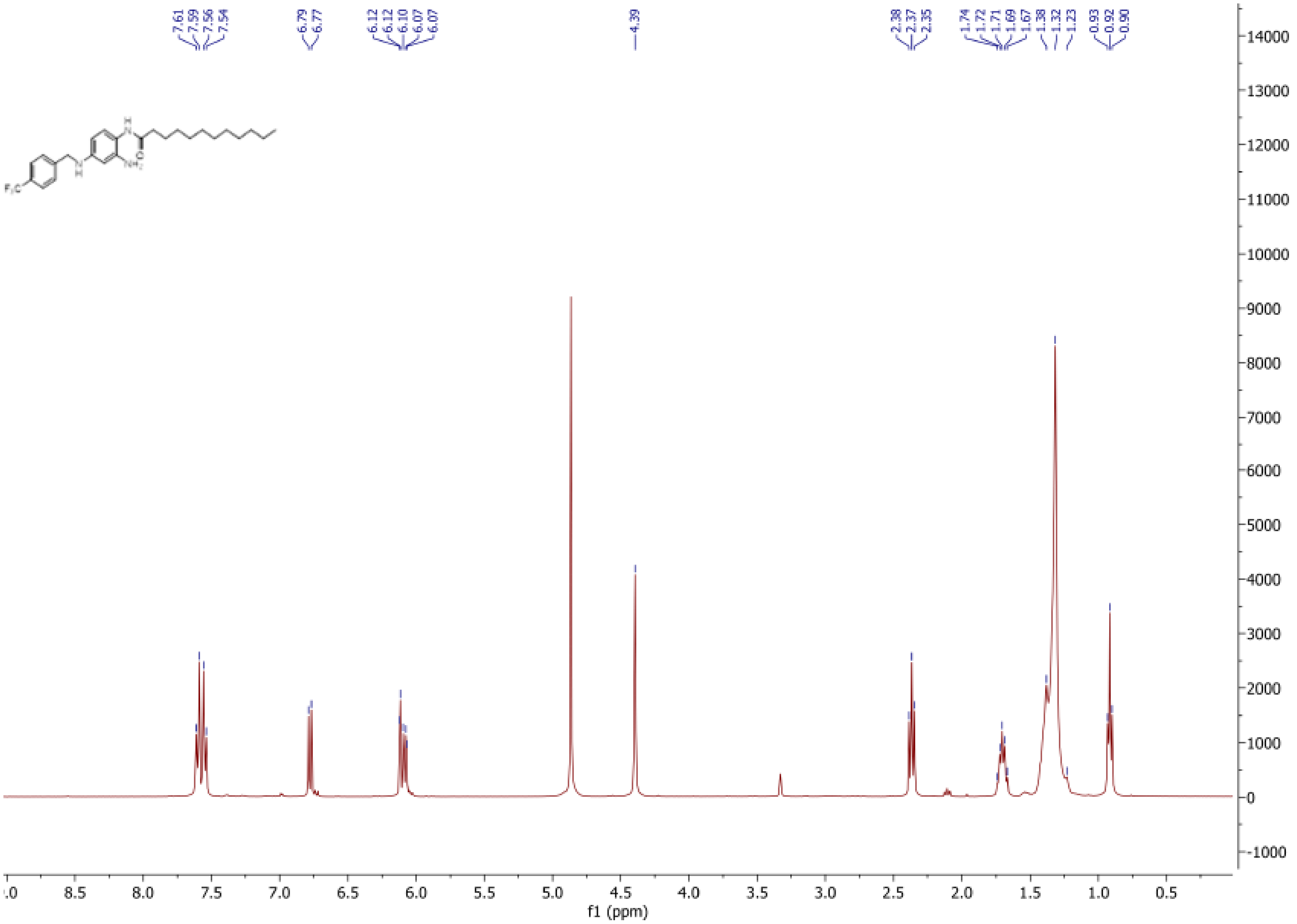

Figure S13: ^{1}H NMR spectra of derivative **16**

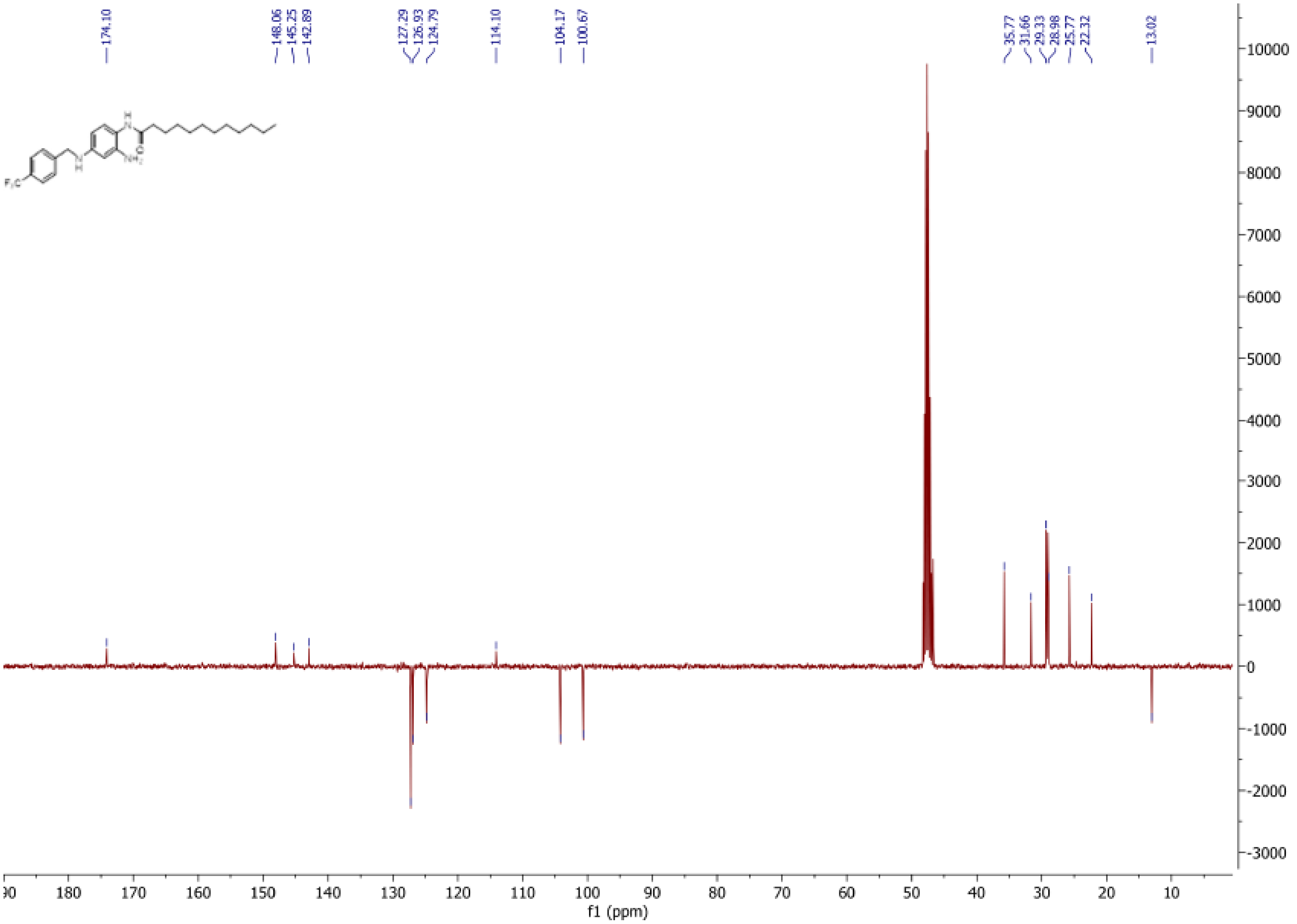

Figure S14: DEPT NMR spectra of derivative **16**

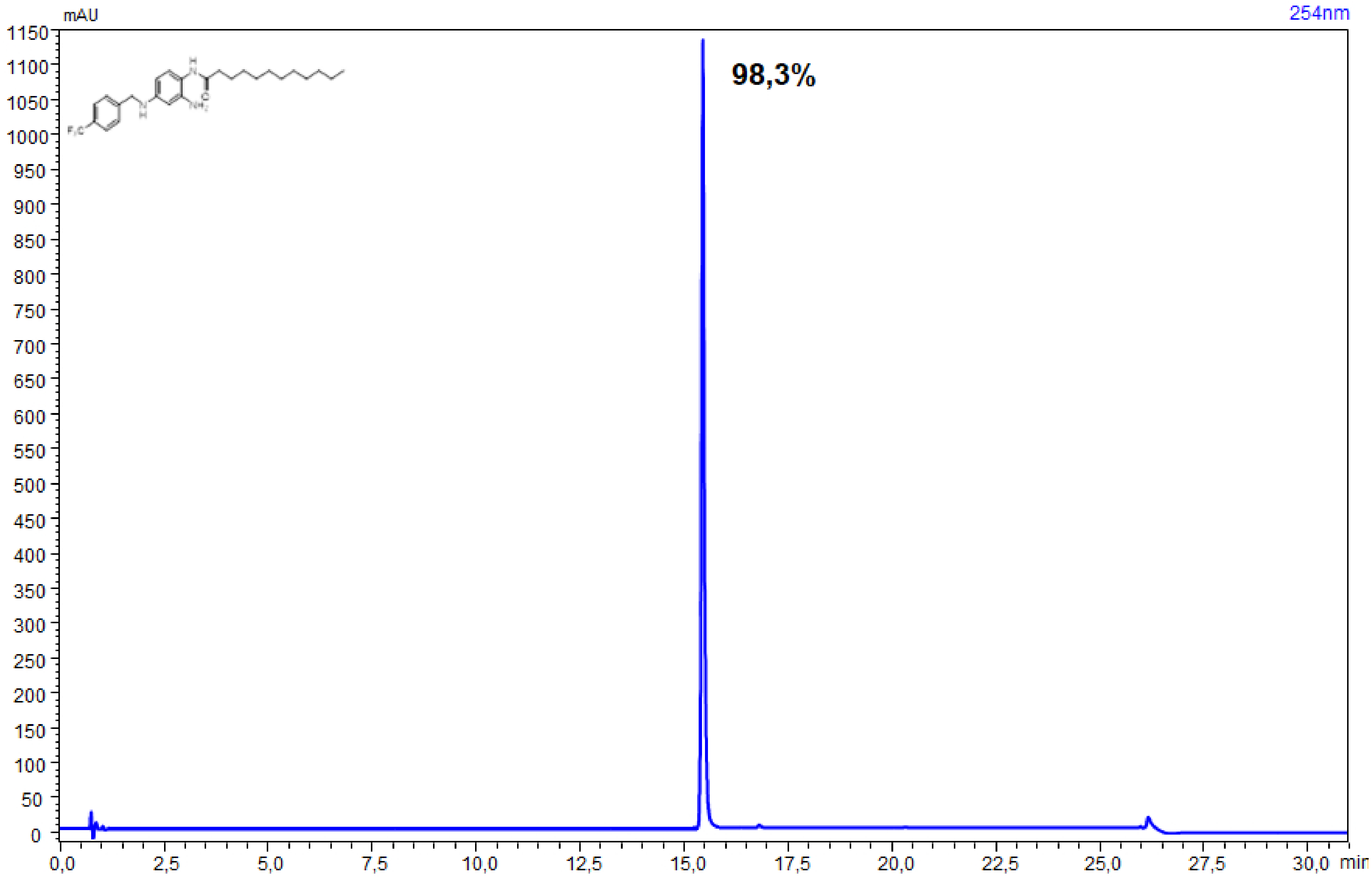

Figure S15: HPLC trace of derivative **16**

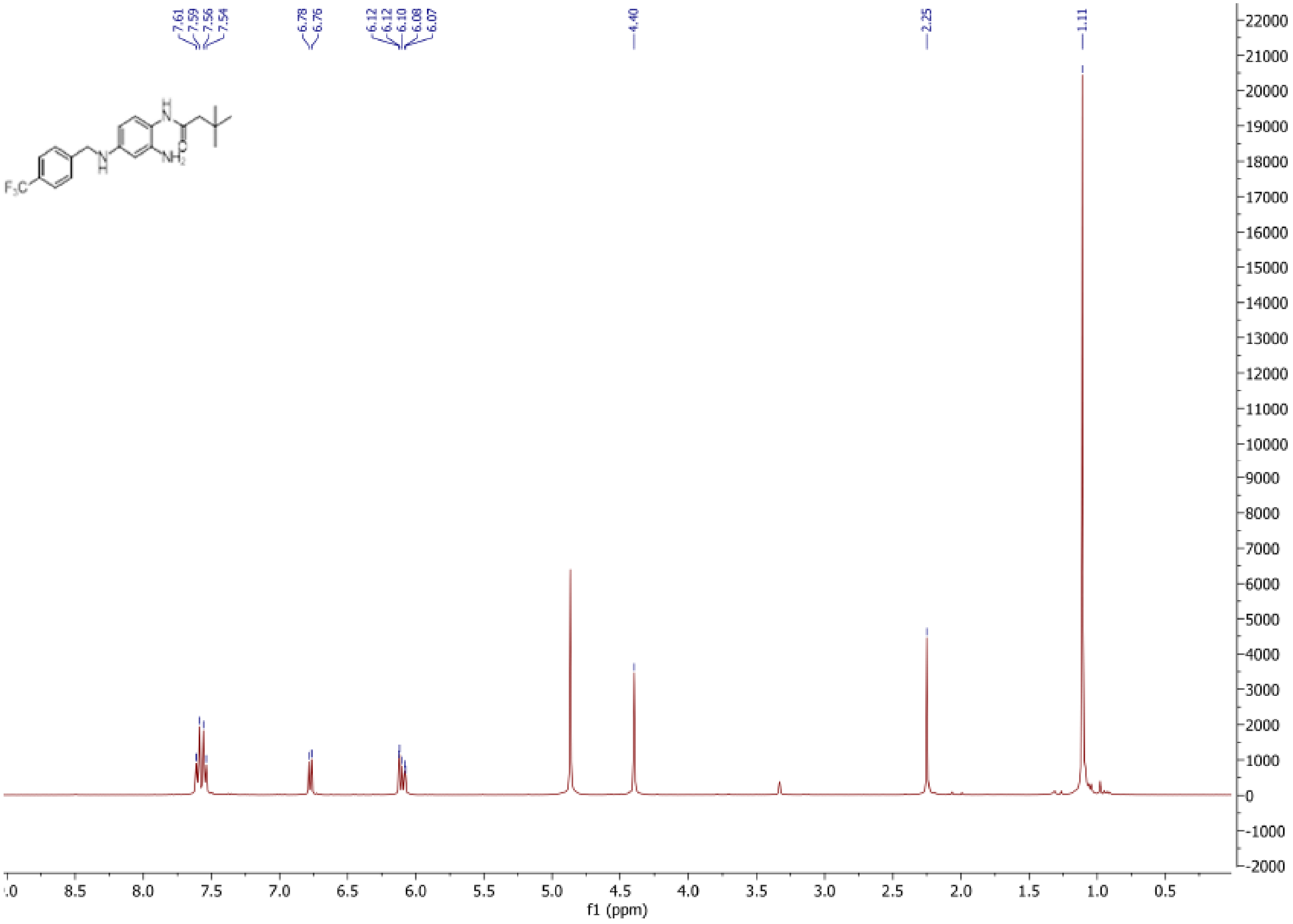

Figure S16: ^{1}H NMR spectra of derivative **17**

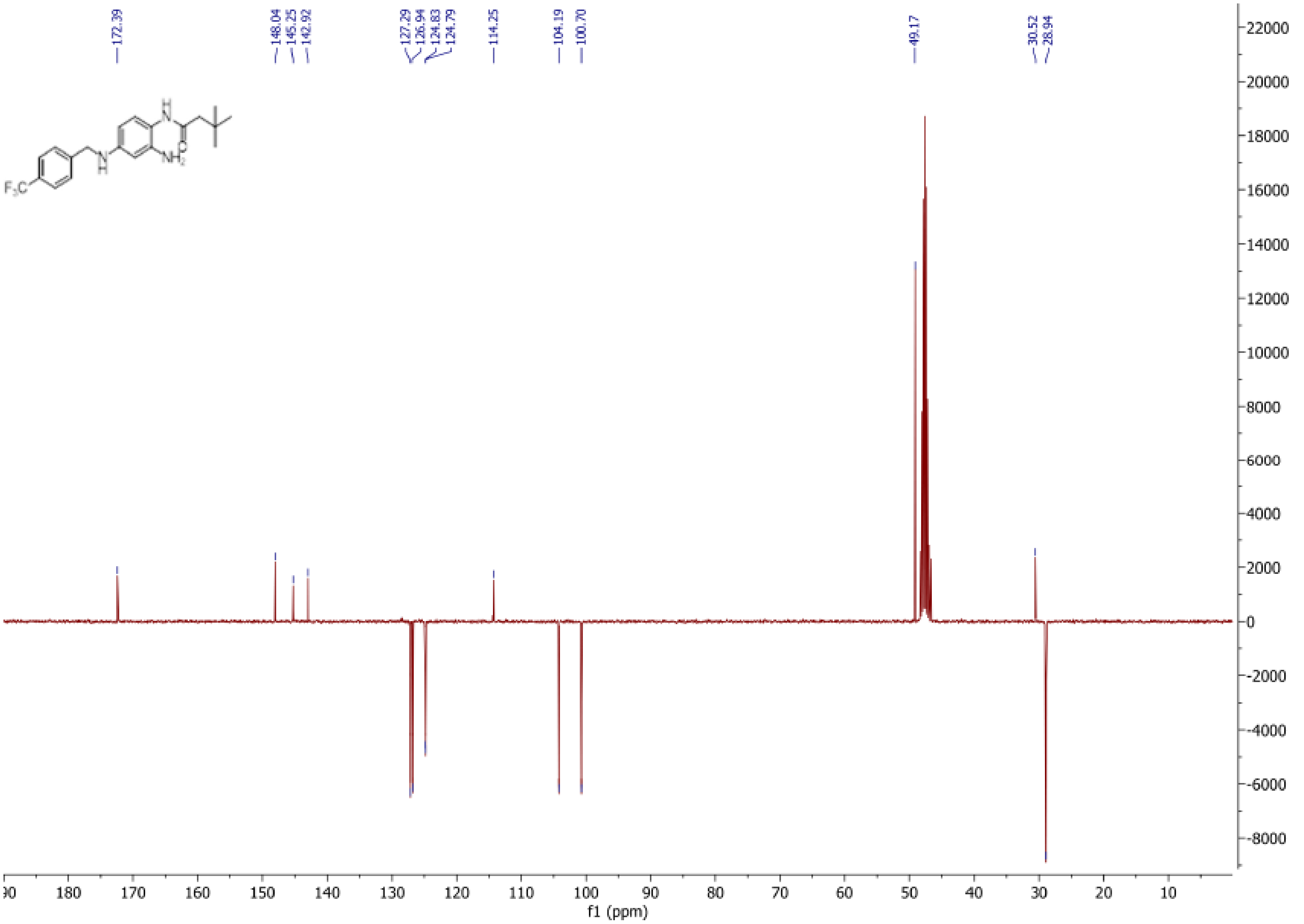

Figure S17: DEPT NMR spectra of derivative **17**

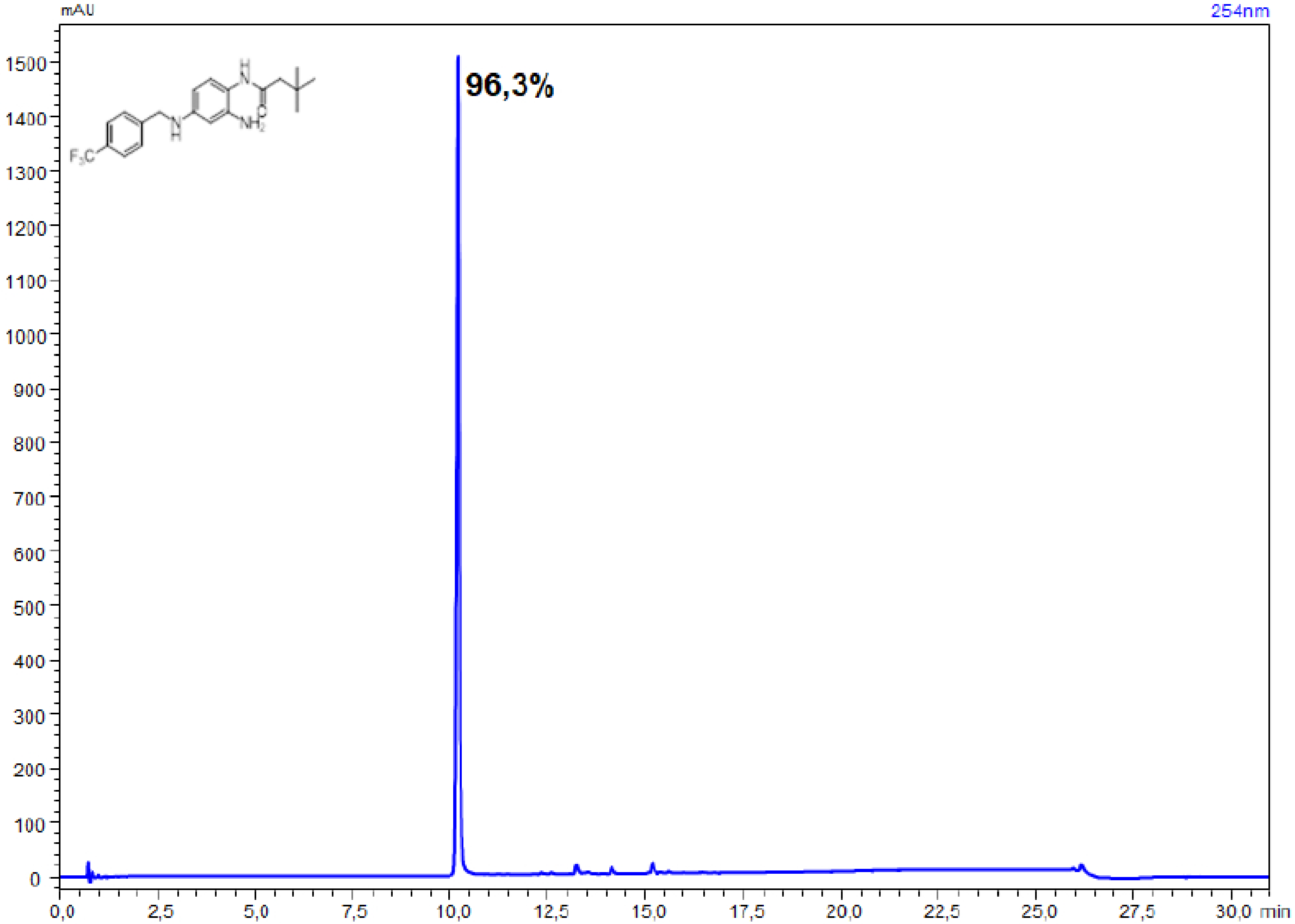

Figure S18: HPLC trace of derivative **17**

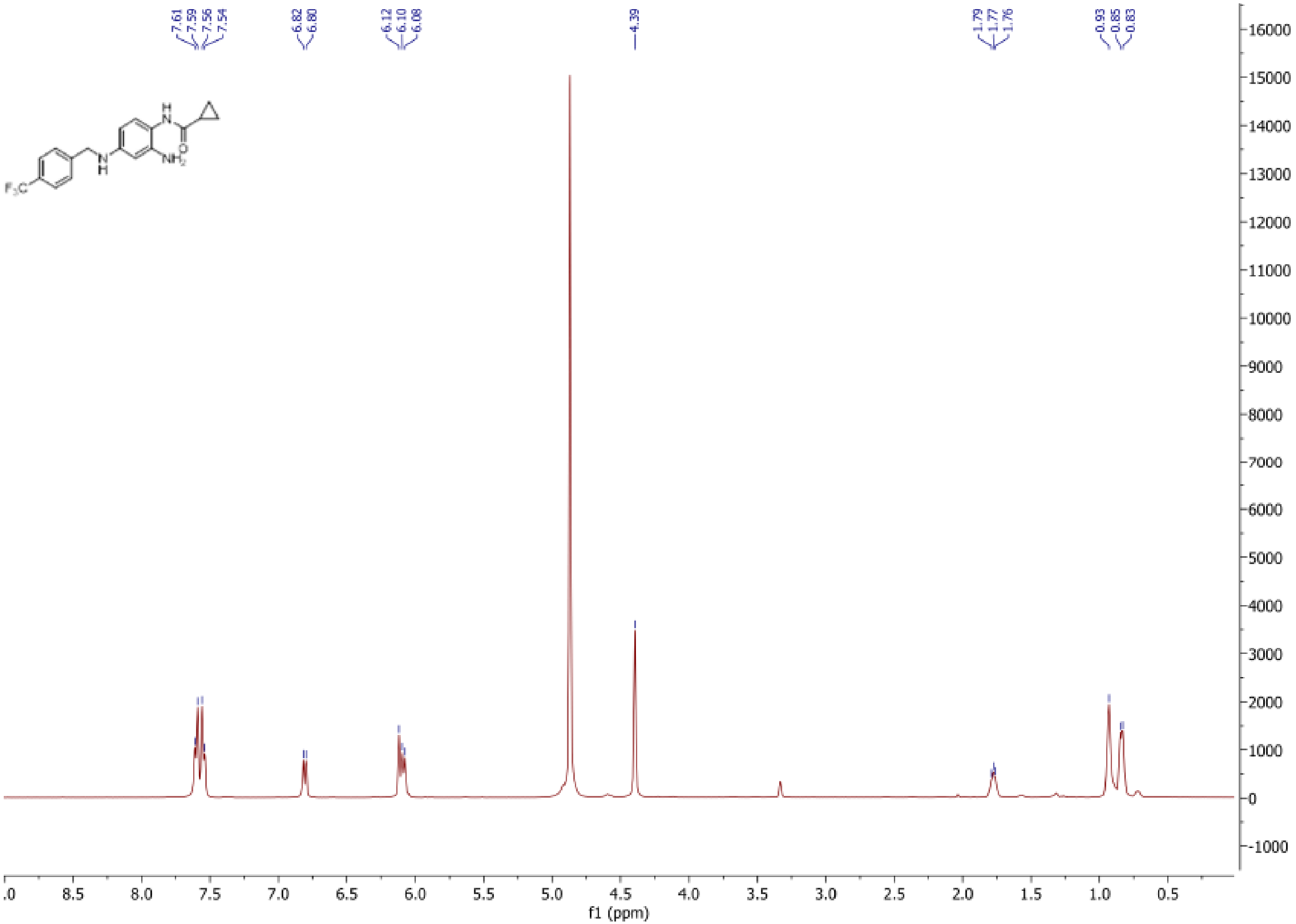

Figure S19: ^{1}H NMR spectra of derivative **18**

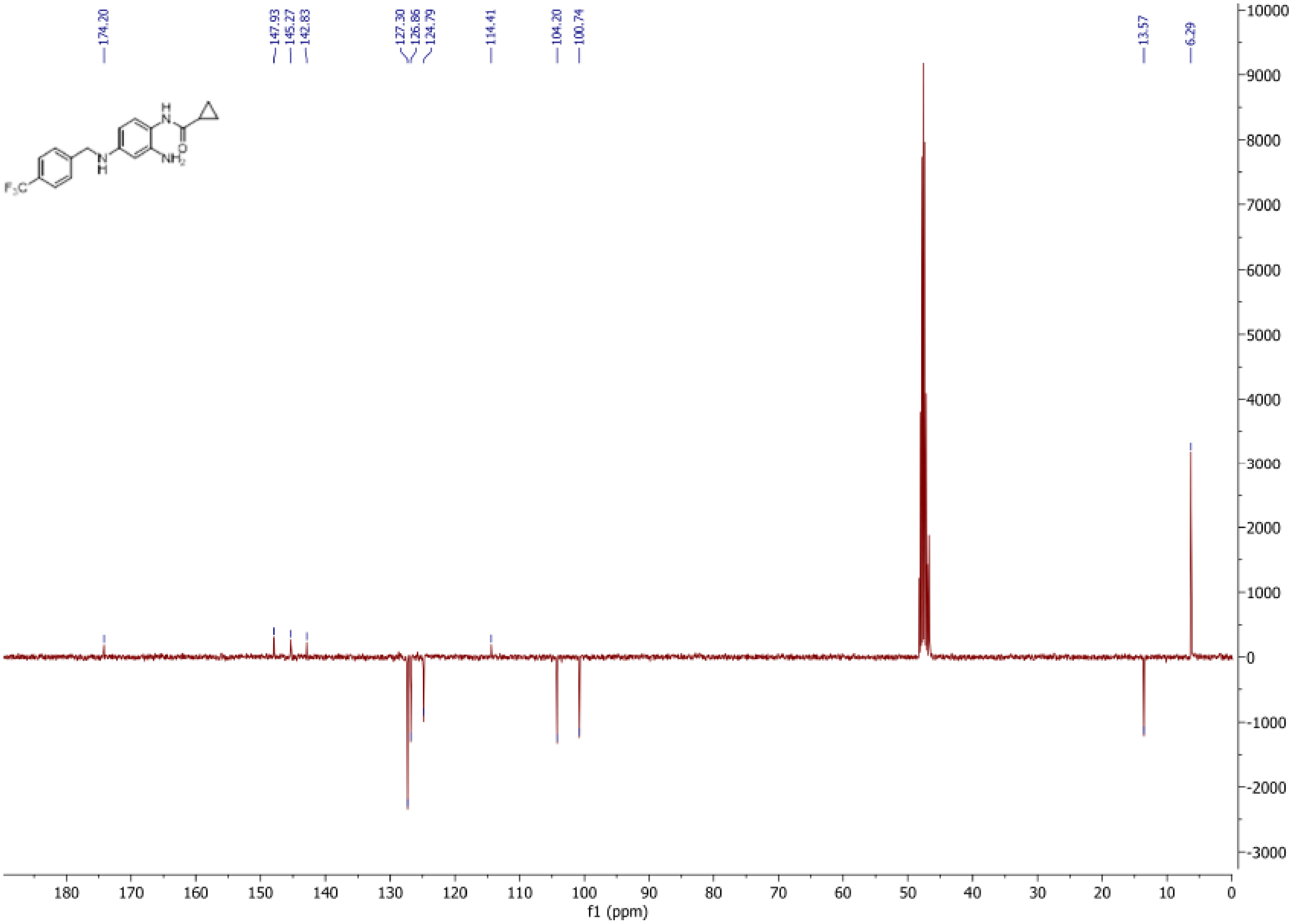

Figure S20: DEPT NMR spectra of derivative **18**

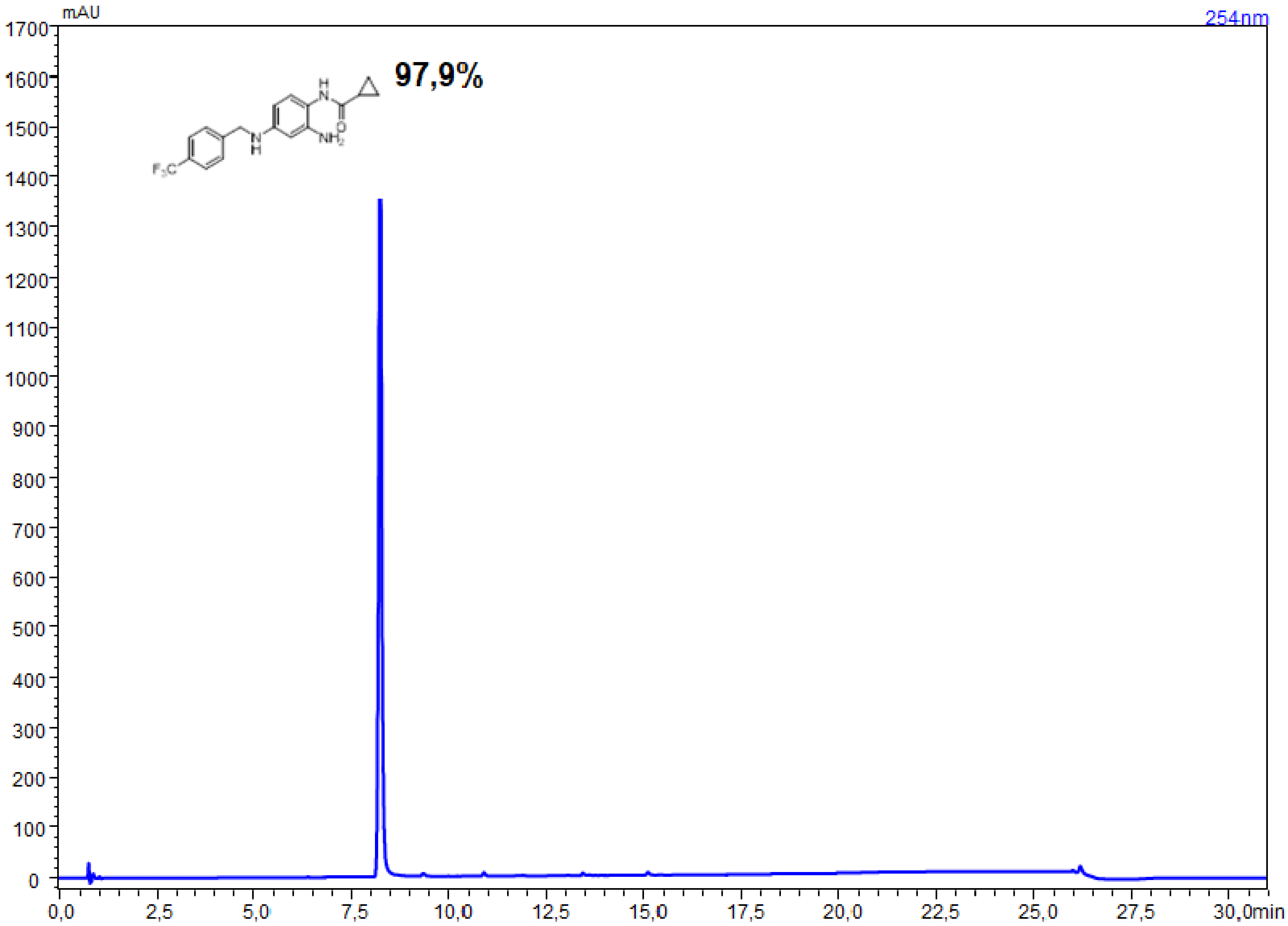

Figure S21: HPLC trace of derivative **18**

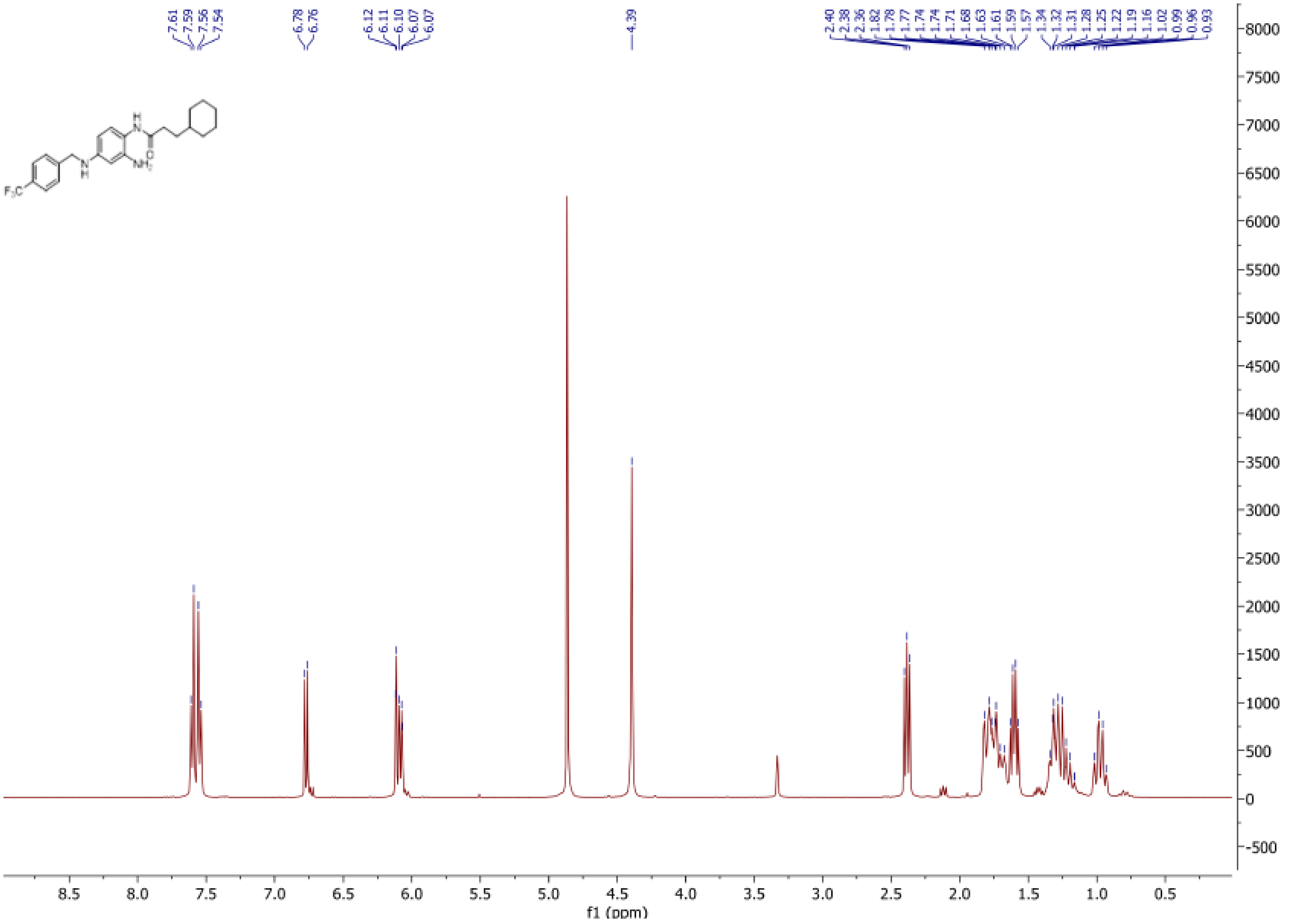

Figure S22: ^{1}H NMR spectra of derivative **19**

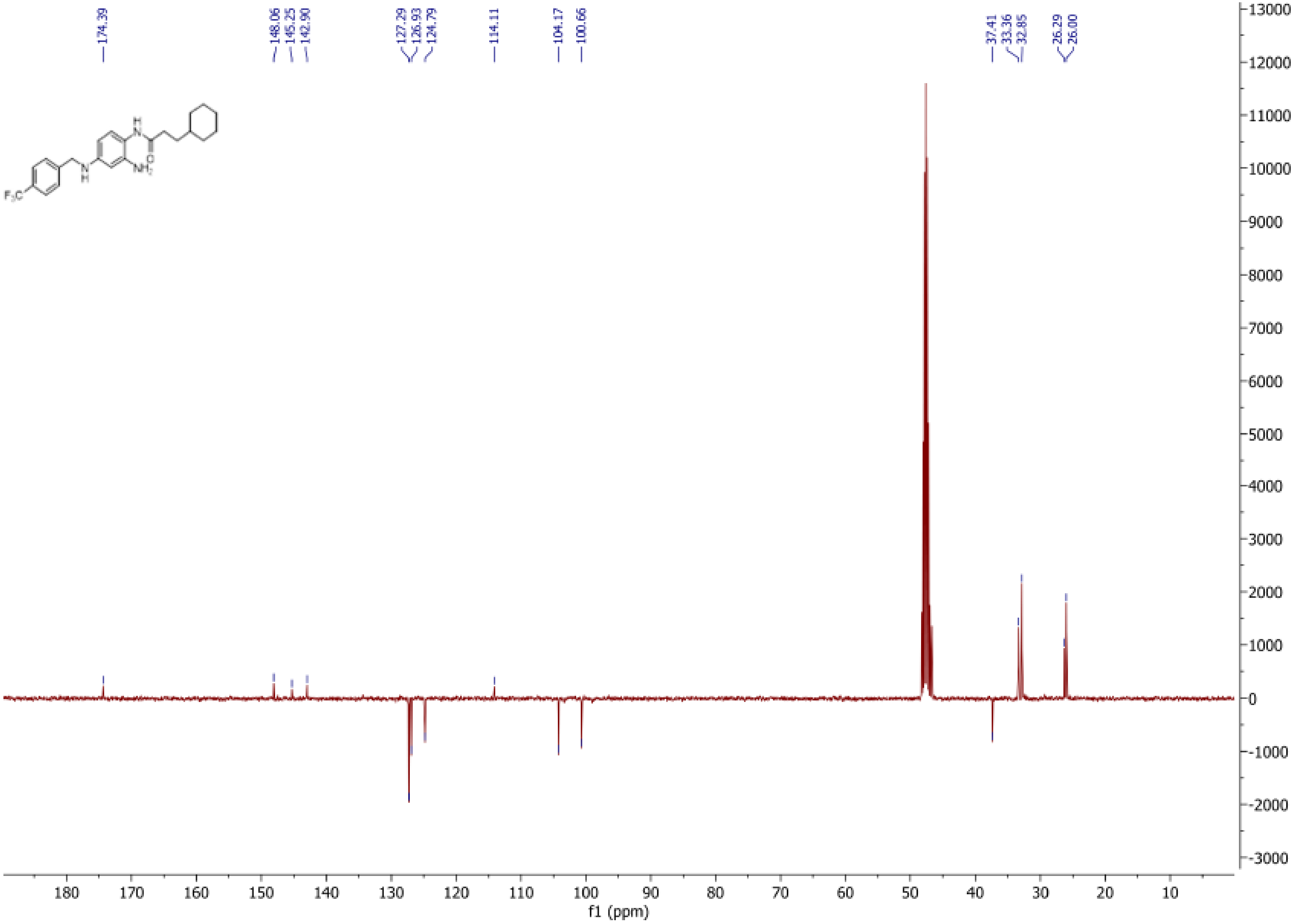

Figure S23: DEPT NMR spectra of derivative **19**

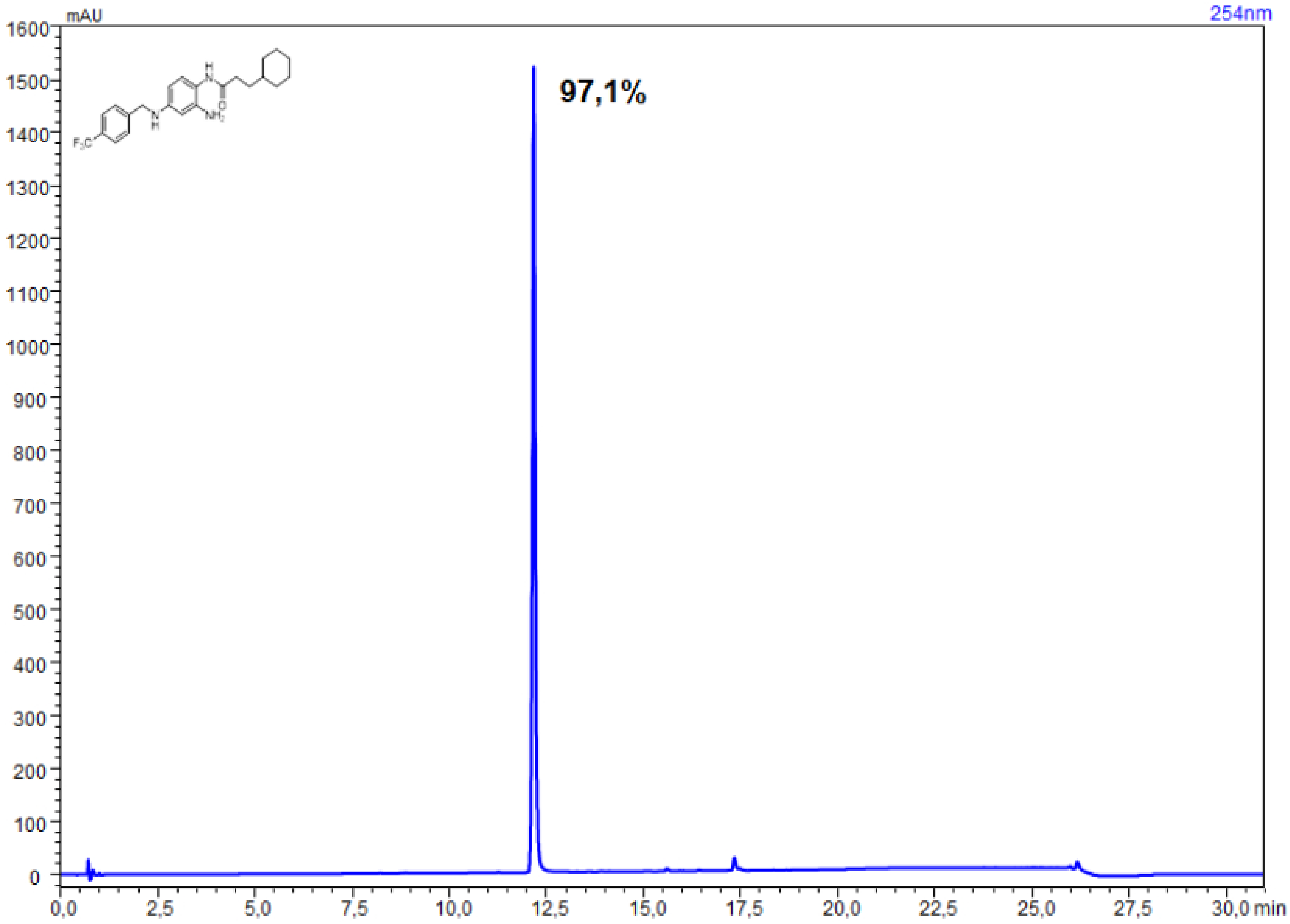

Figure S24: HPLC trace of derivative **19**

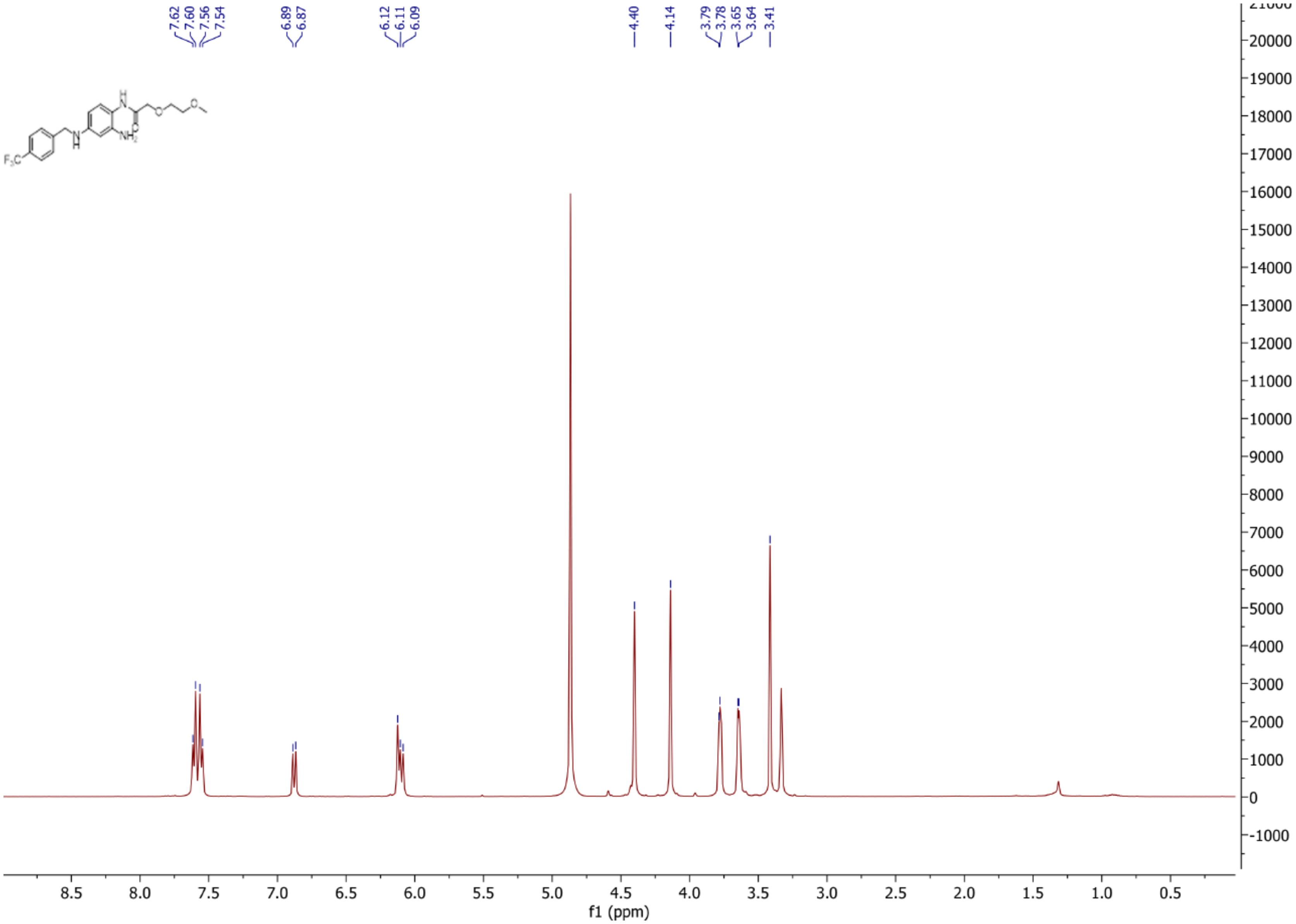

Figure S25: ^{1}H NMR spectra of derivative **20**

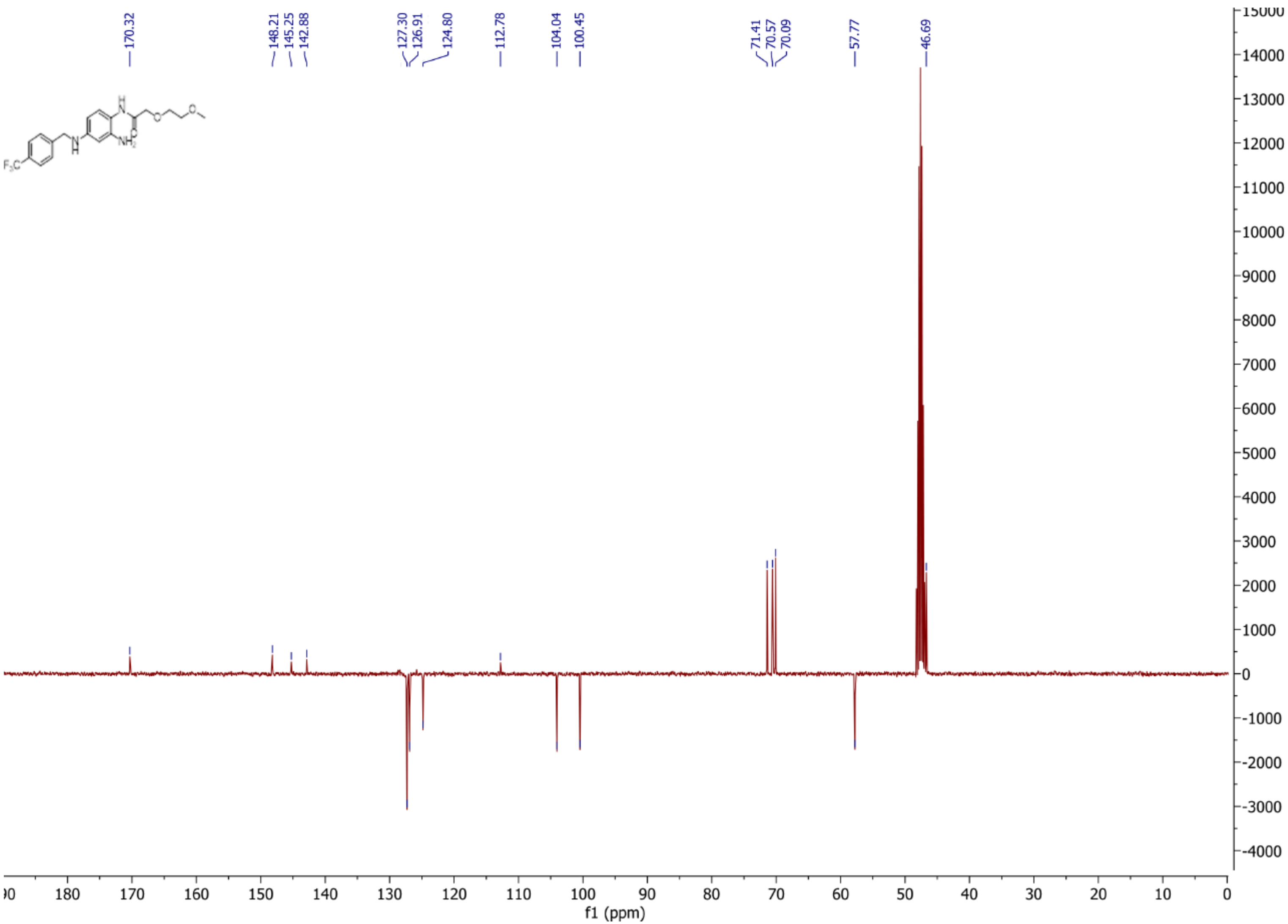

Figure S26: DEPT NMR spectra of derivative **20**

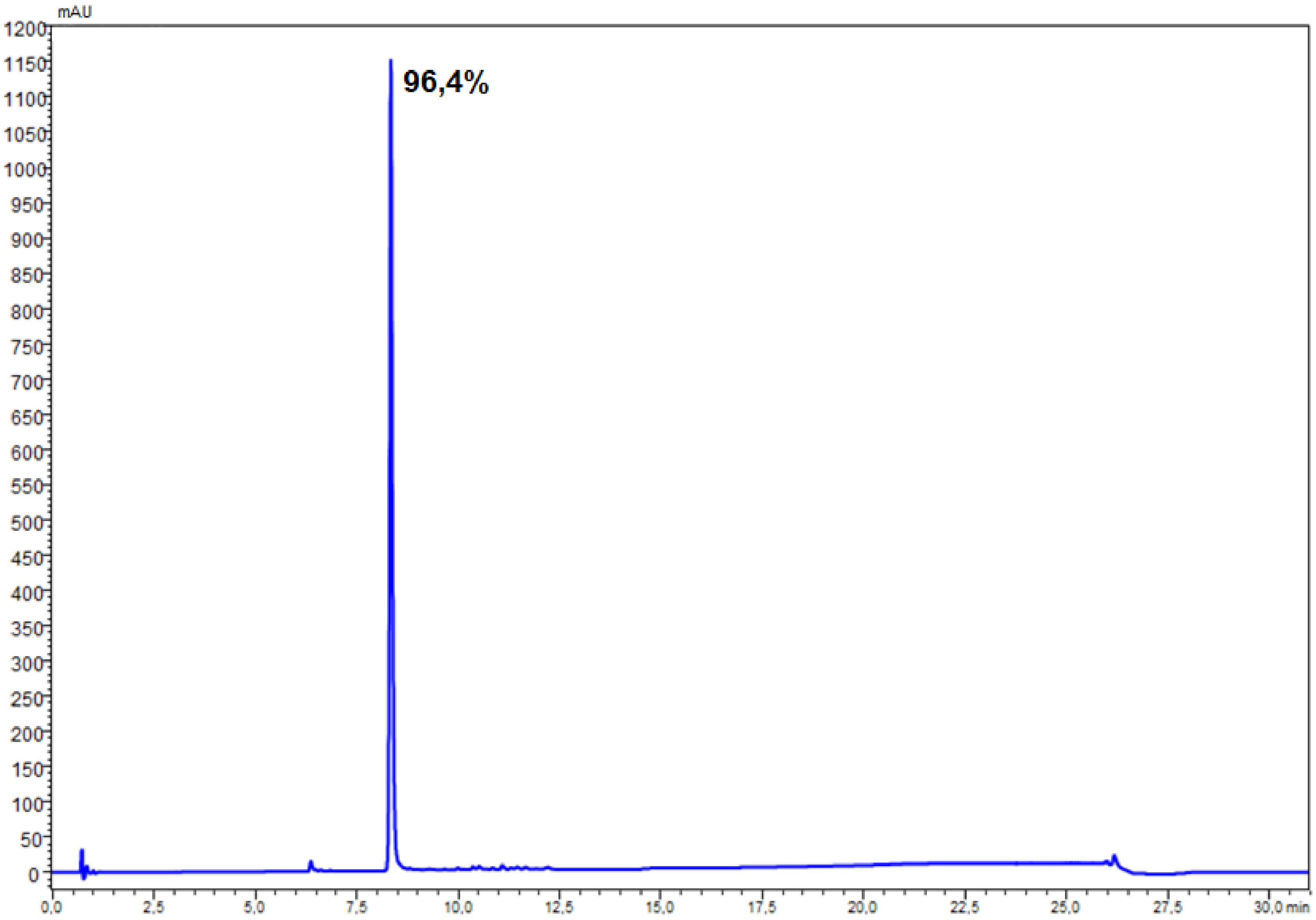

Figure S27: HPLC trace of derivative **20**

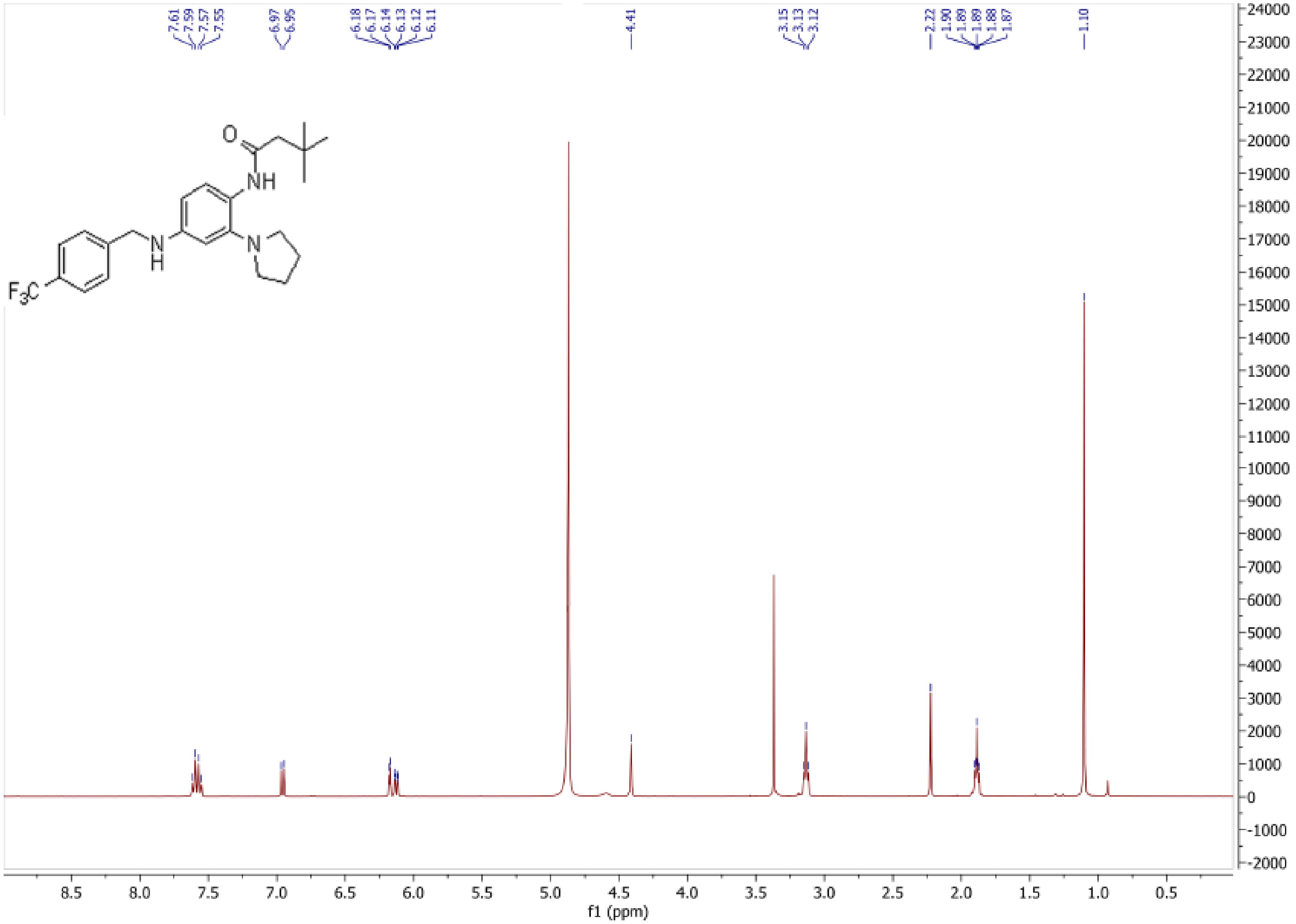

Figure S28: ^{1}H NMR spectra of derivative **23**

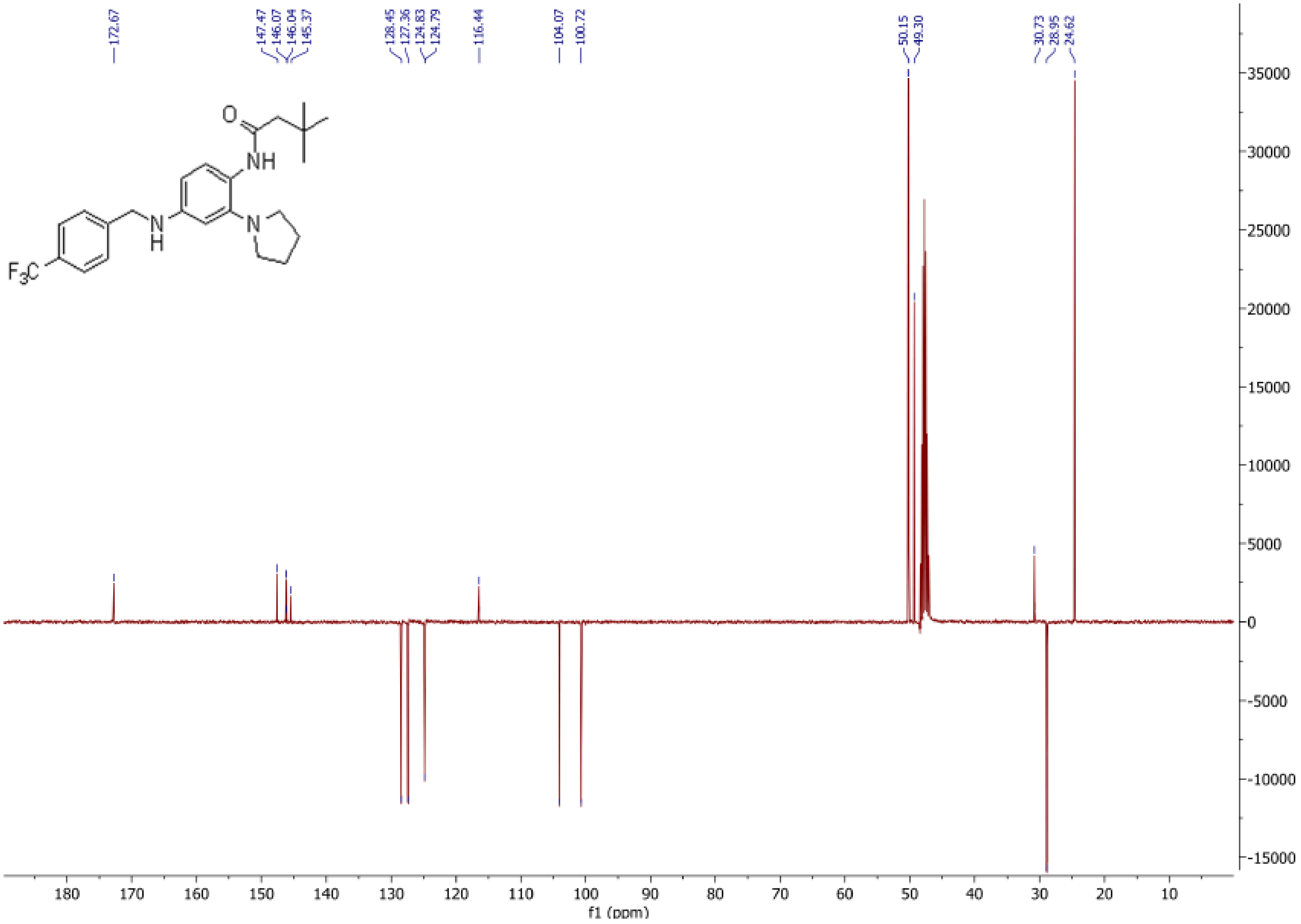

Figure S29: DEPT NMR spectra of derivative **23**

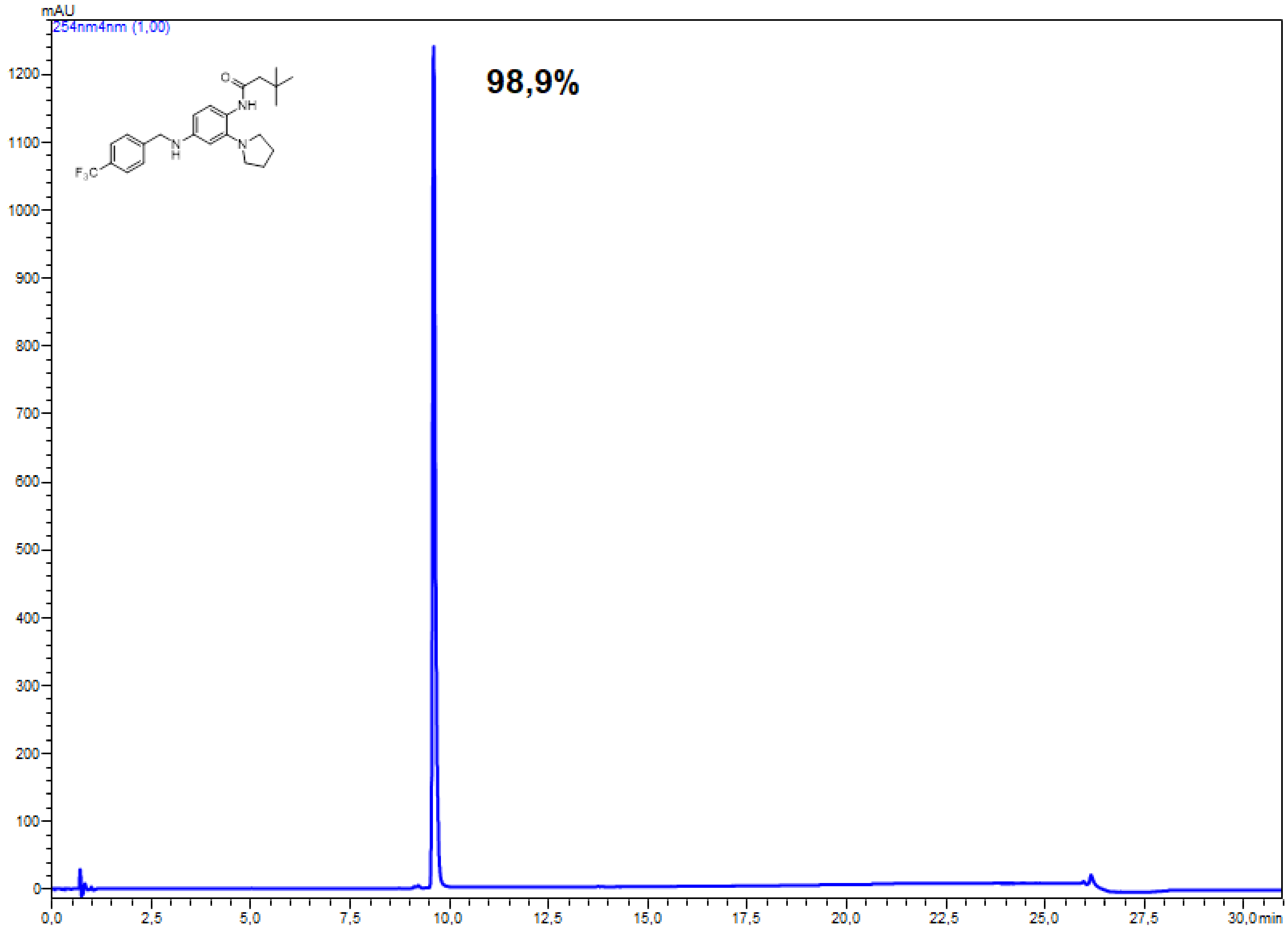

Figure S30: HPLC trace of derivative **23**

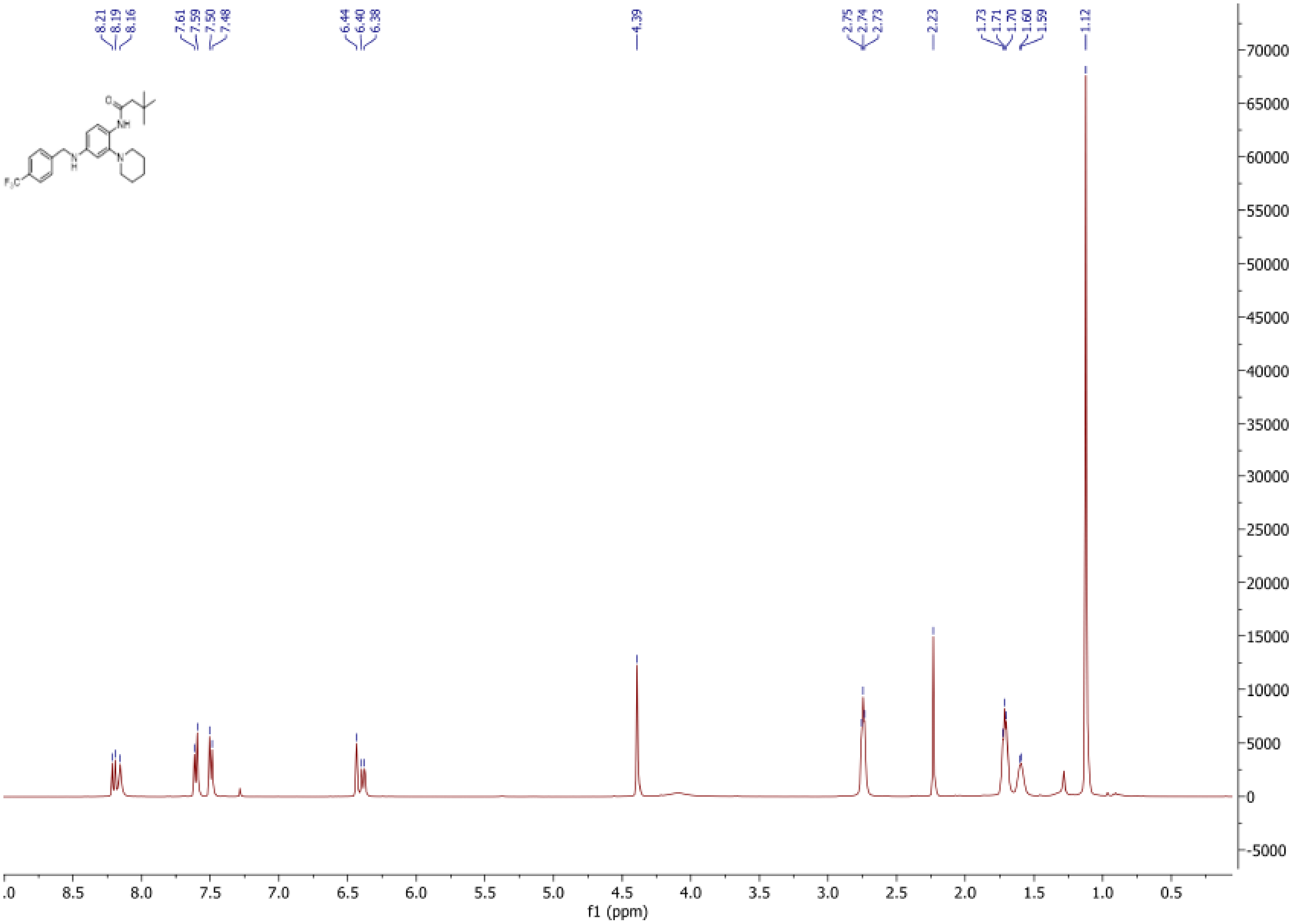

Figure S31: ^{1}H NMR spectra of derivative **24**

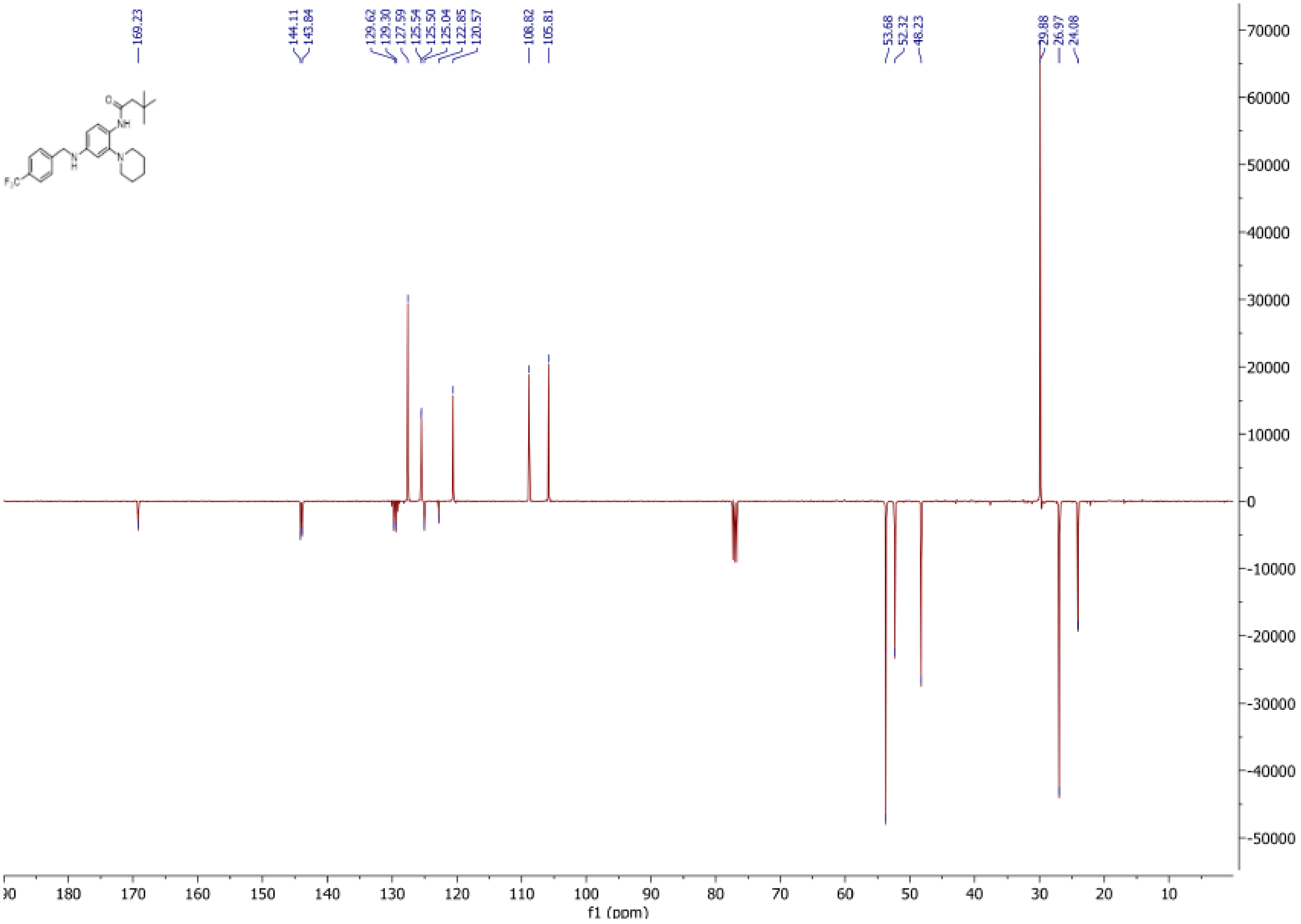

Figure S32: DEPT NMR spectra of derivative **24**

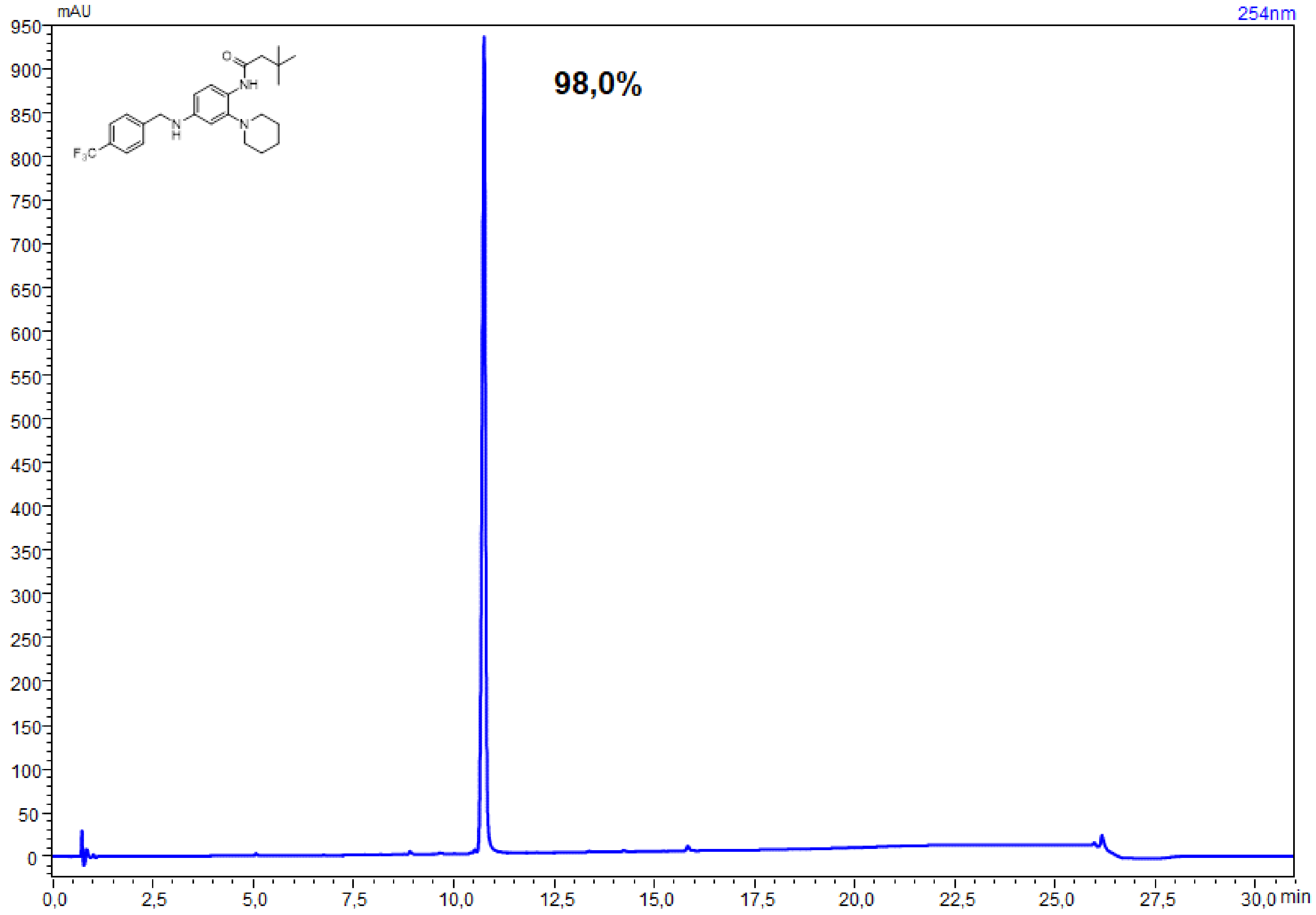

Figure S33: HPLC trace of derivative **24**

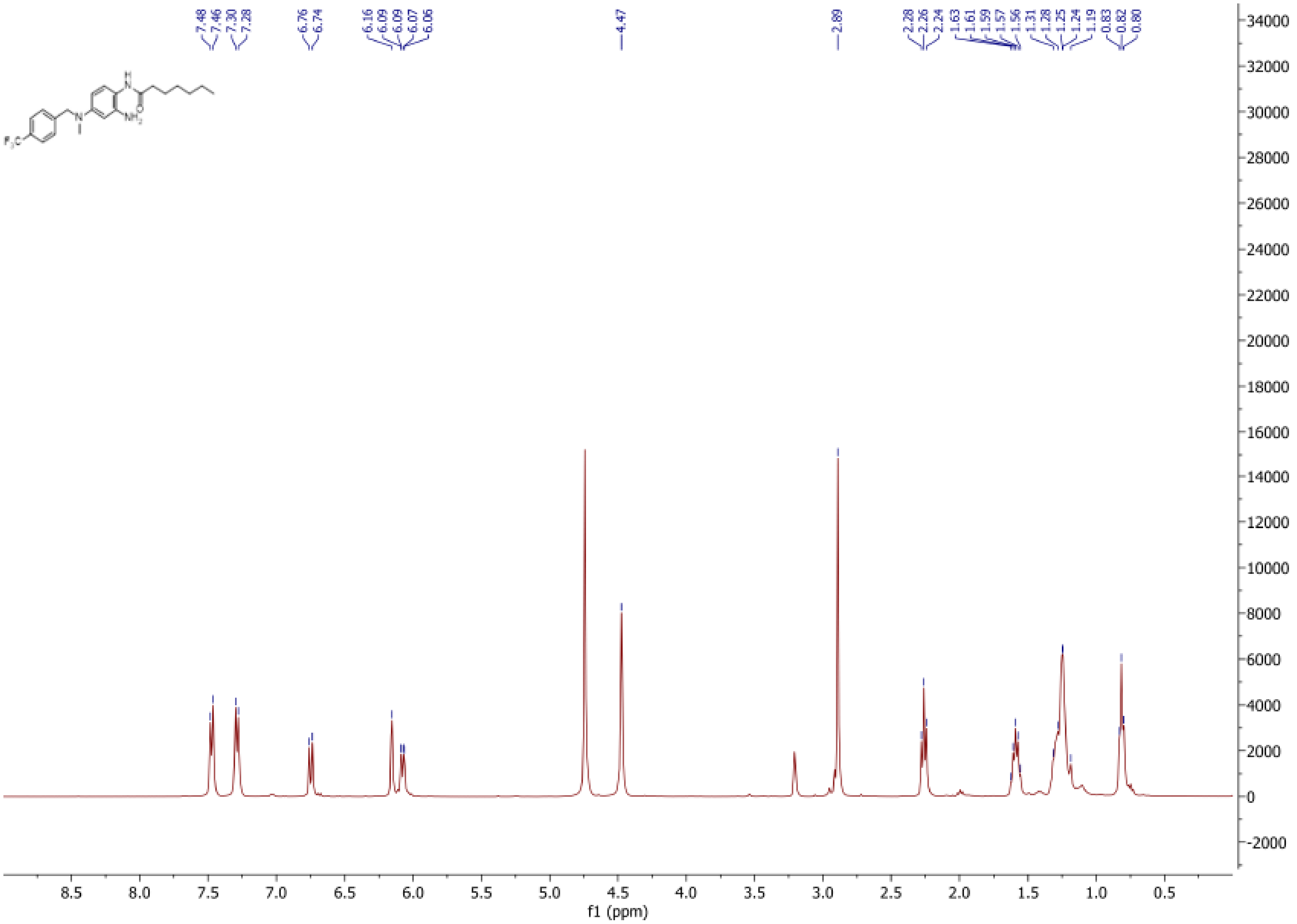

Figure S34: ^{1}H NMR spectra of derivative **25**

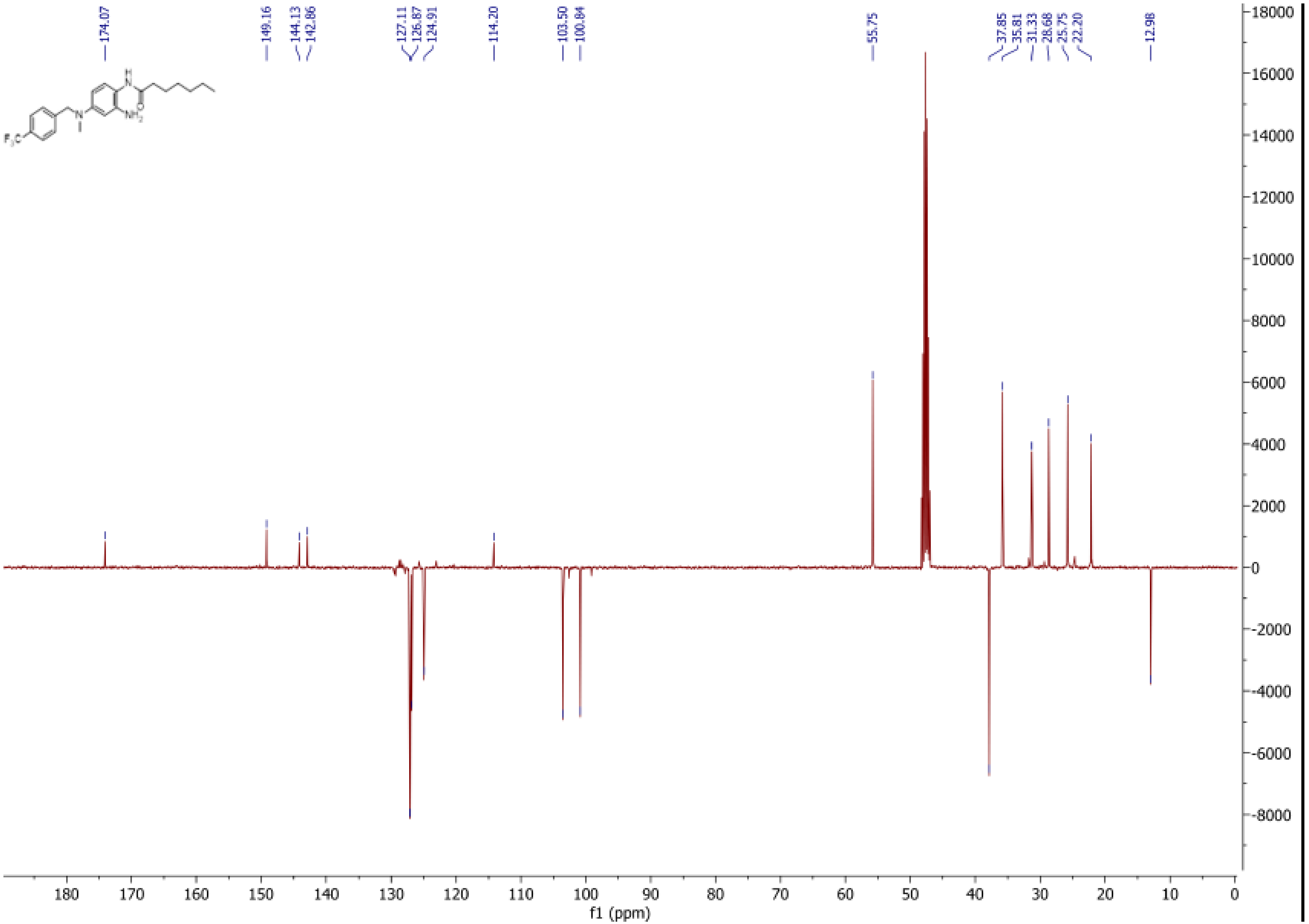

Figure S35: DEPT NMR spectra of derivative **25**

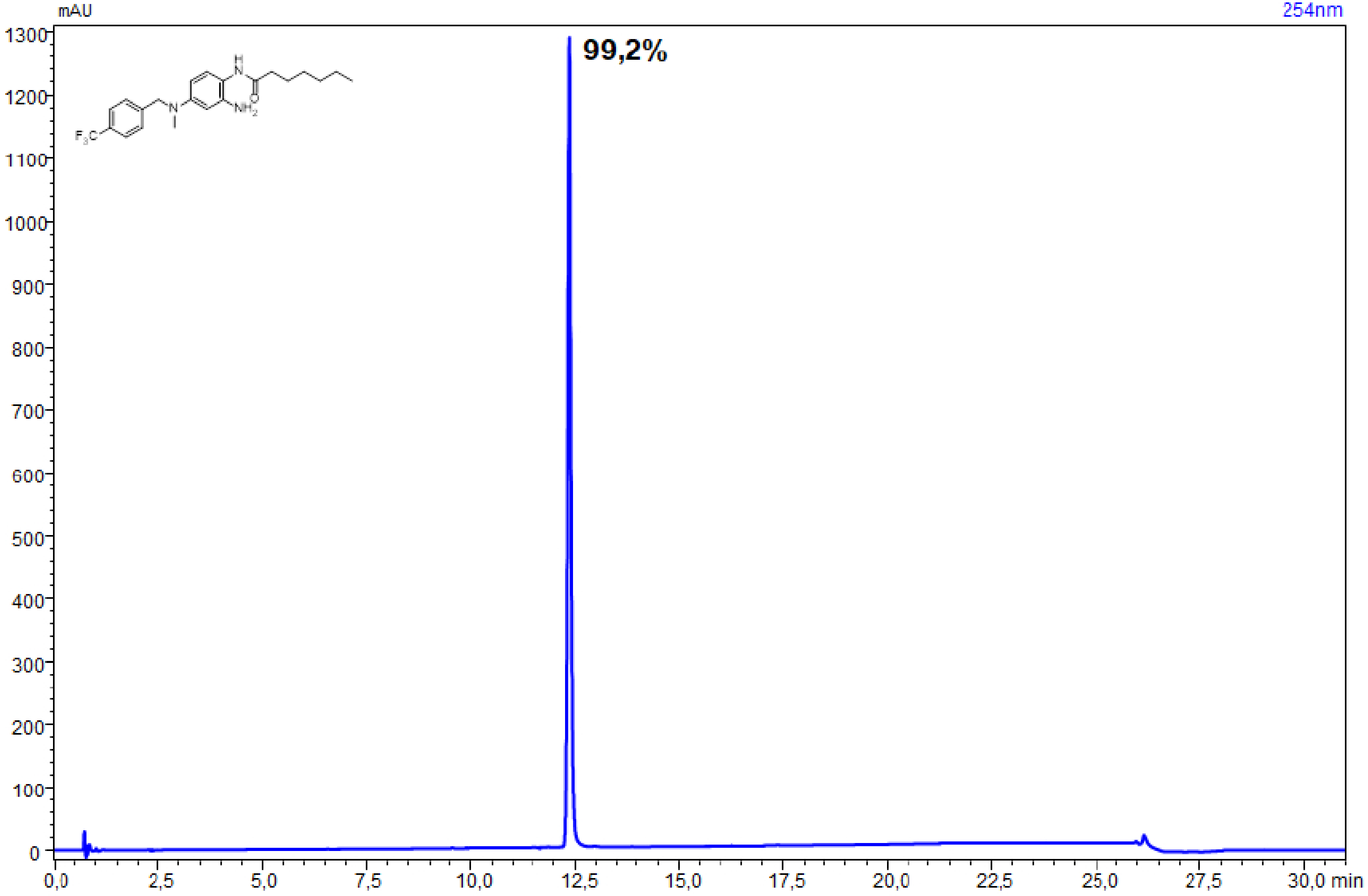

Figure S36: HPLC trace of derivative **25**

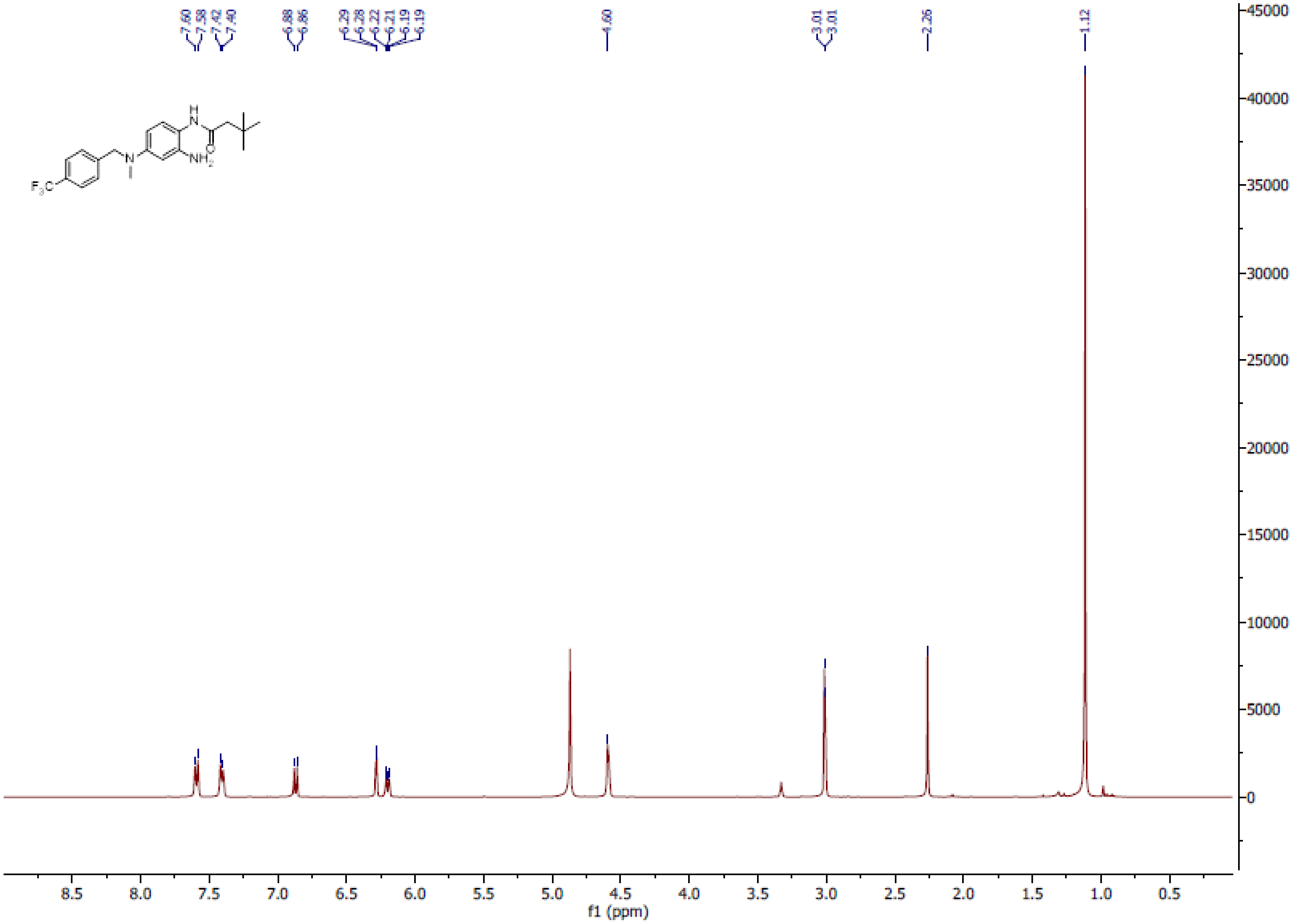

Figure S37: ^{1}H NMR spectra of derivative **26**

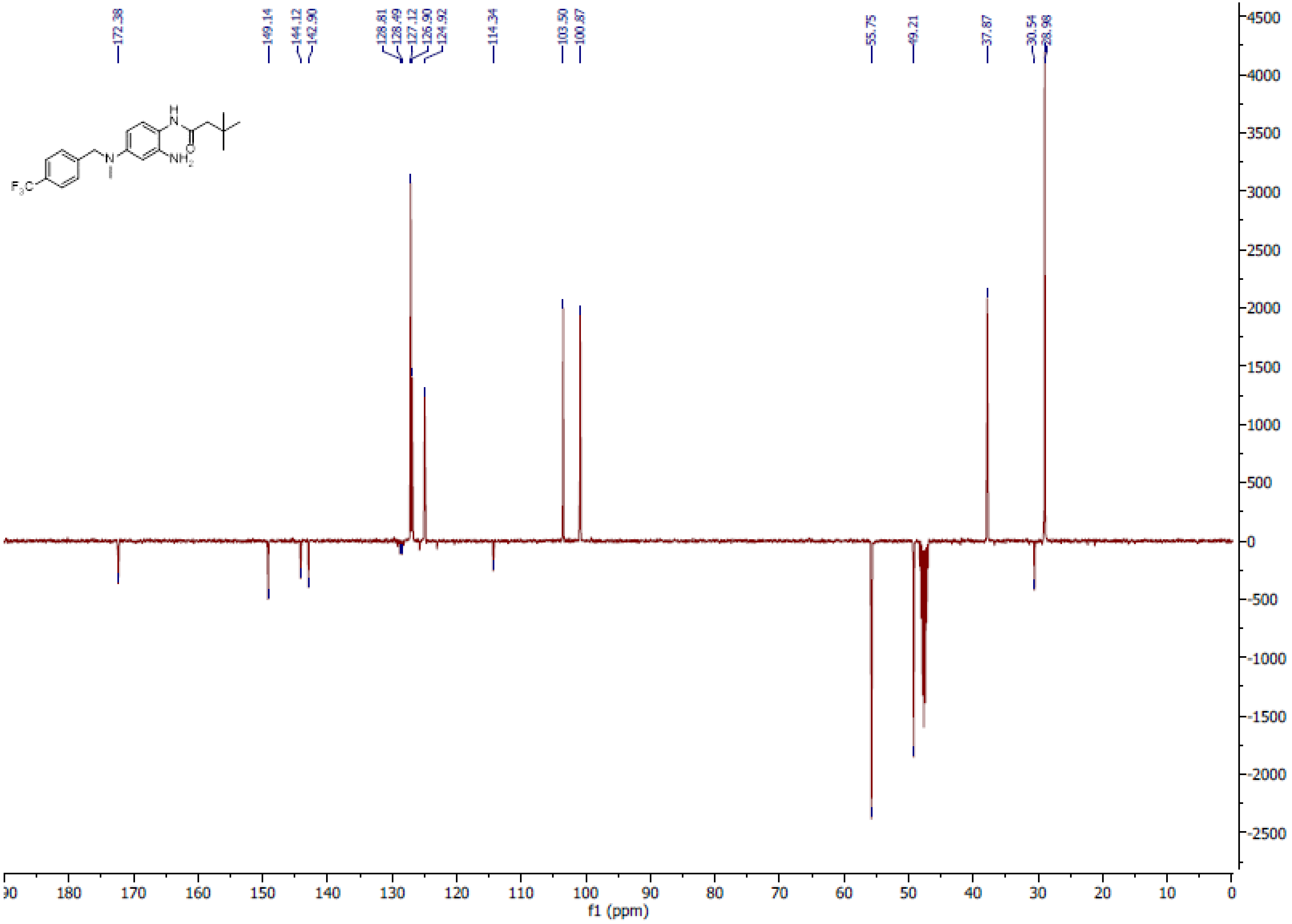

Figure S38: DEPT NMR spectra of derivative **26**

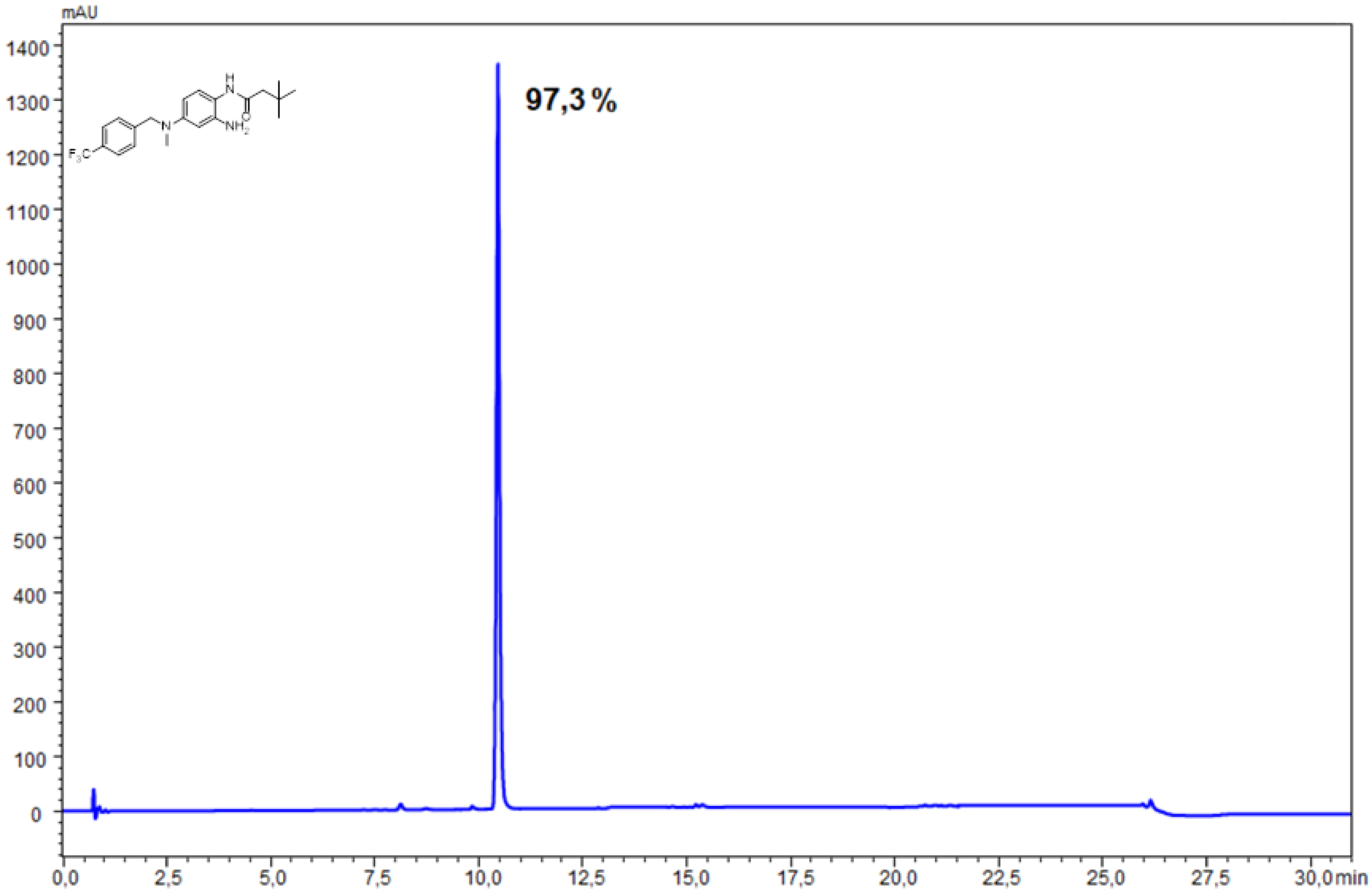

Figure S39: HPLC trace of derivative **26**

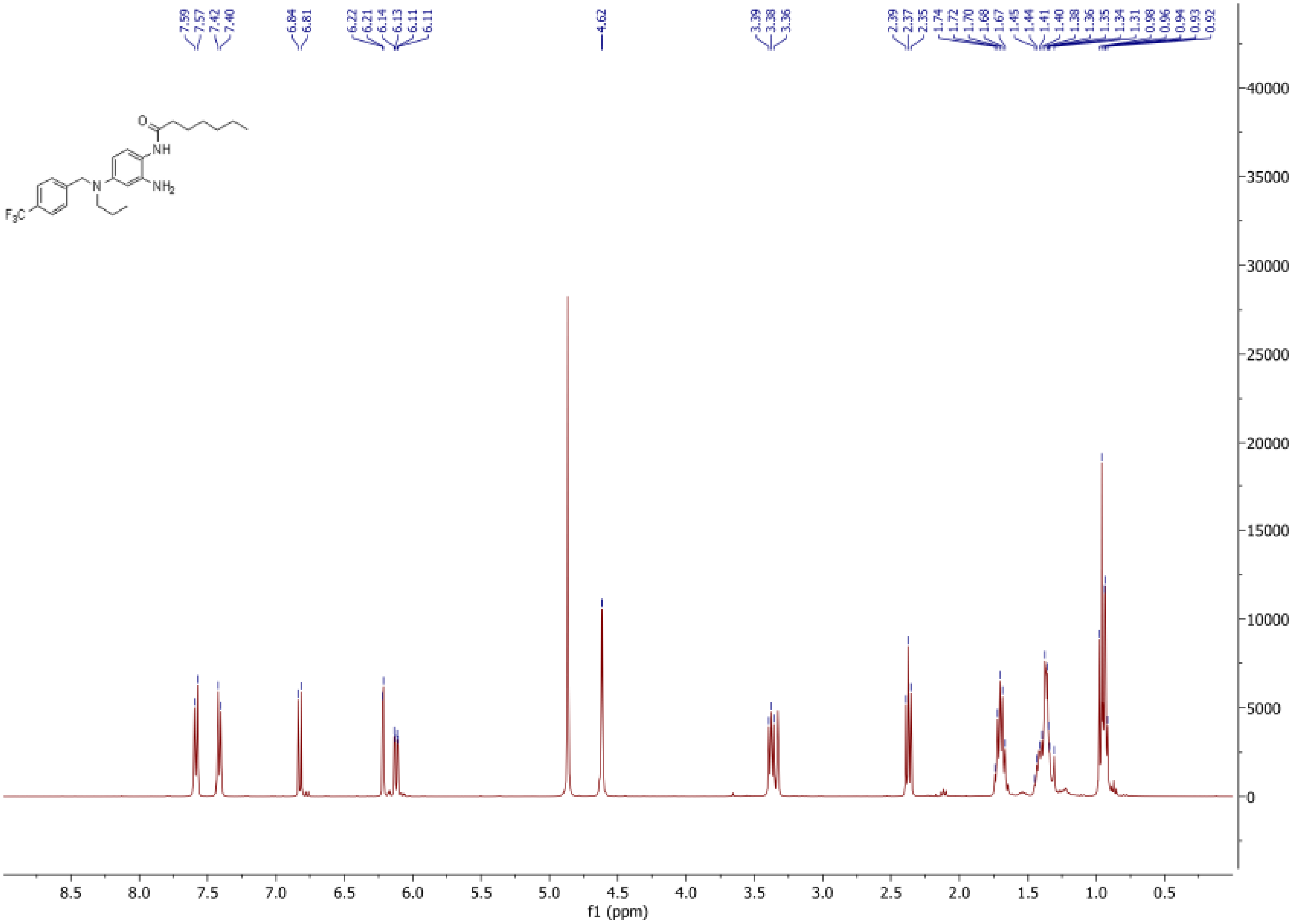

Figure S30: ^{1}H NMR spectra of derivative **27**

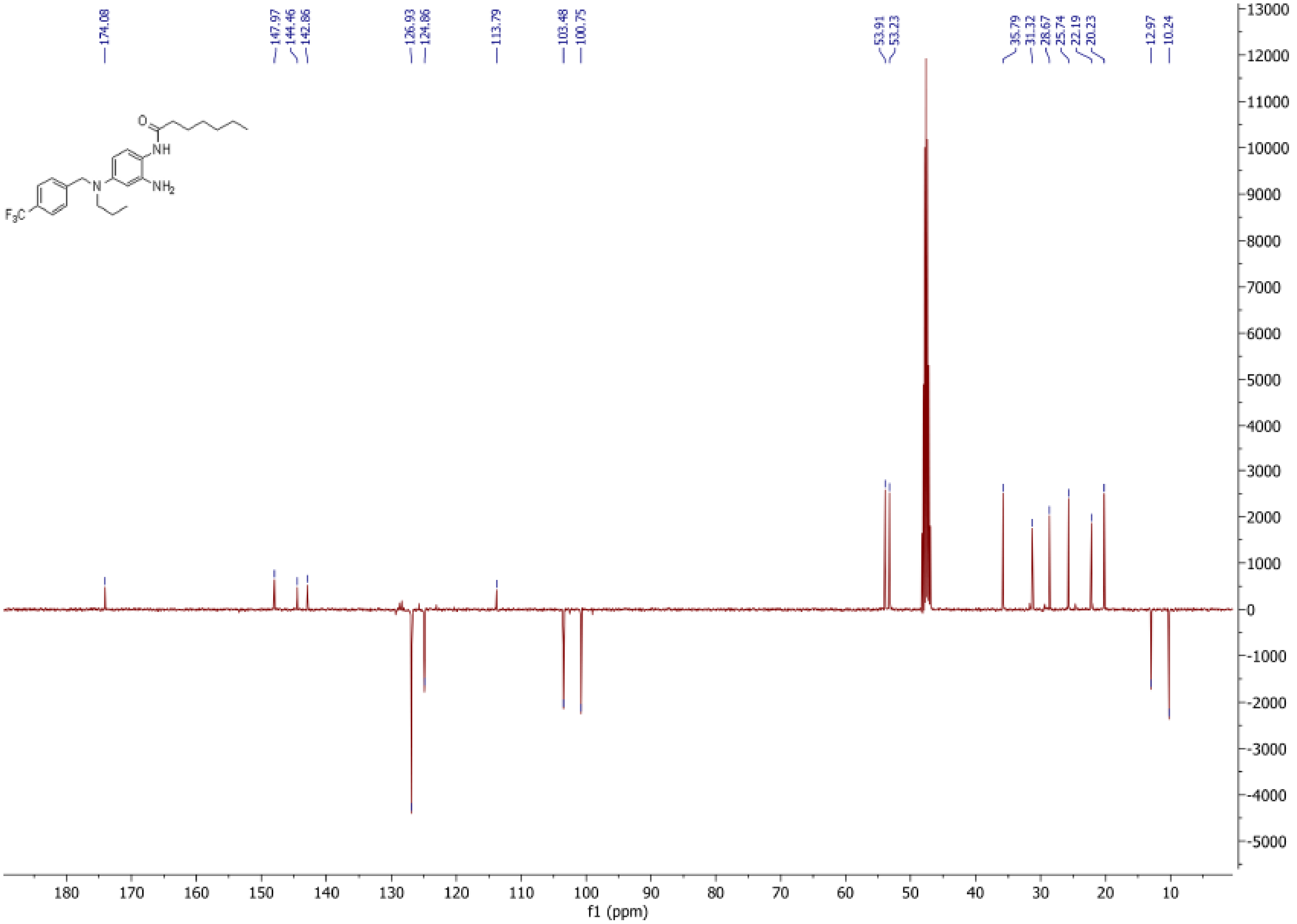

Figure S41: DEPT NMR spectra of derivative **27**

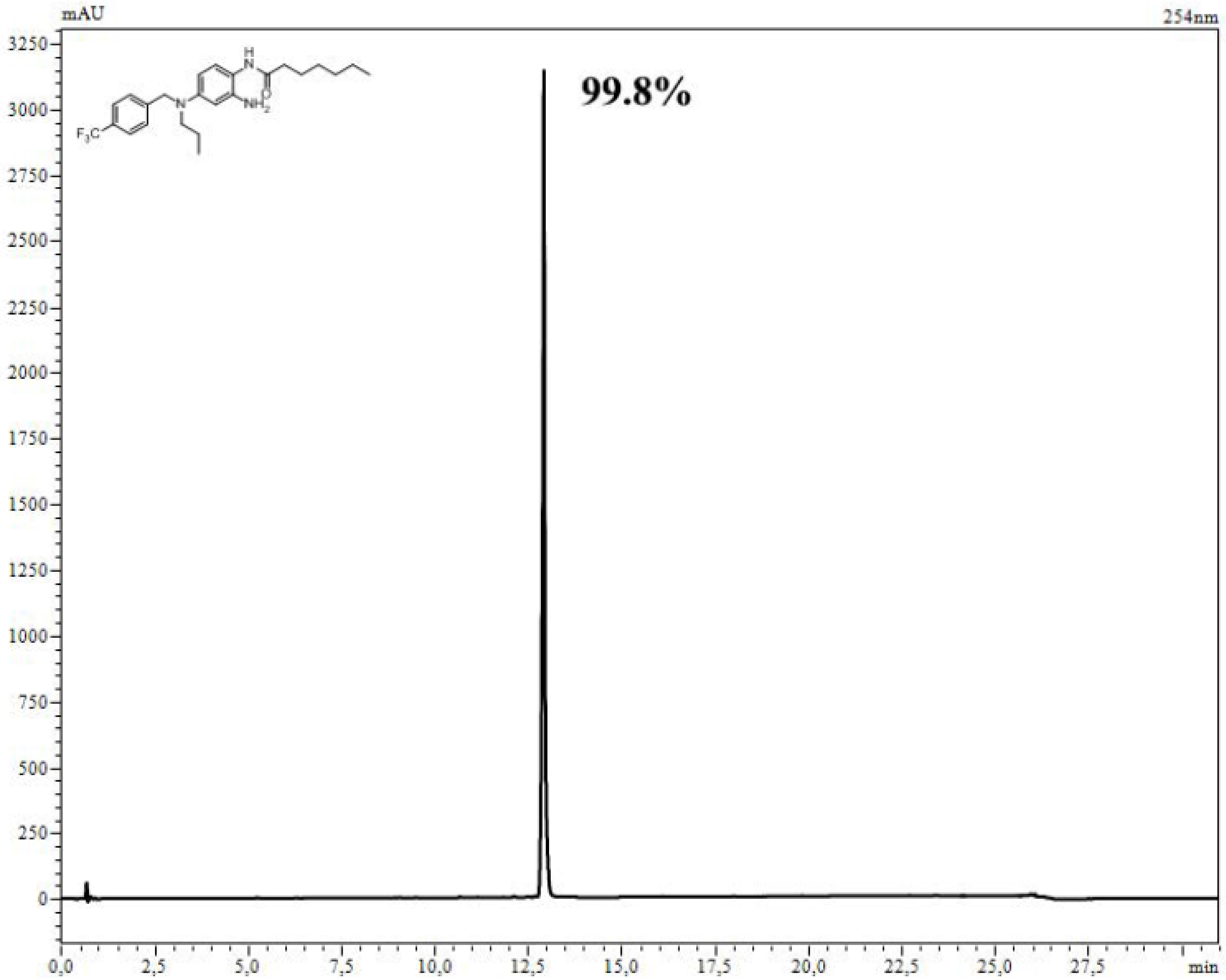

Figure S42: HPLC trace of derivative **27**

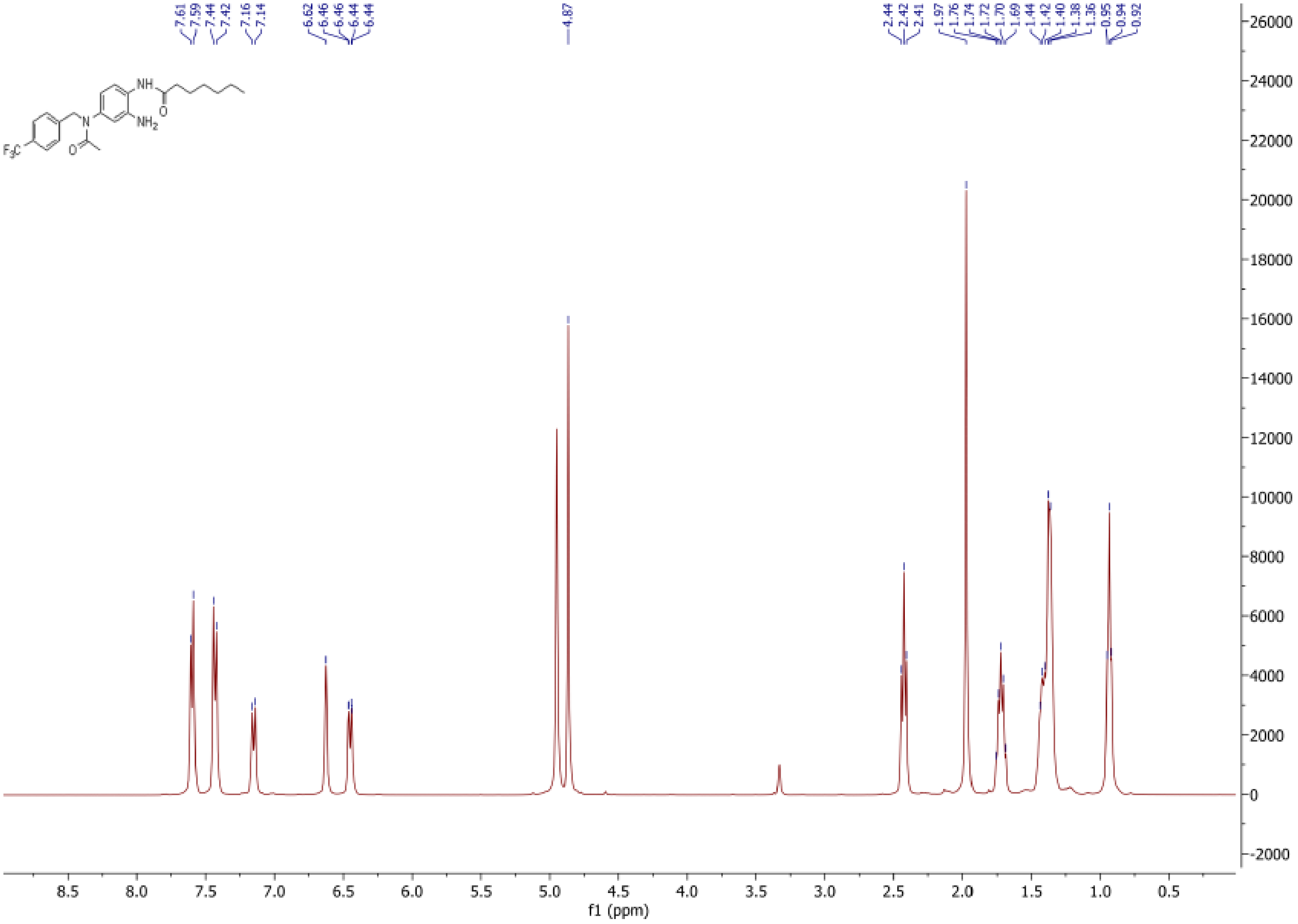

Figure S43: ^{1}H NMR spectra of derivative **28**

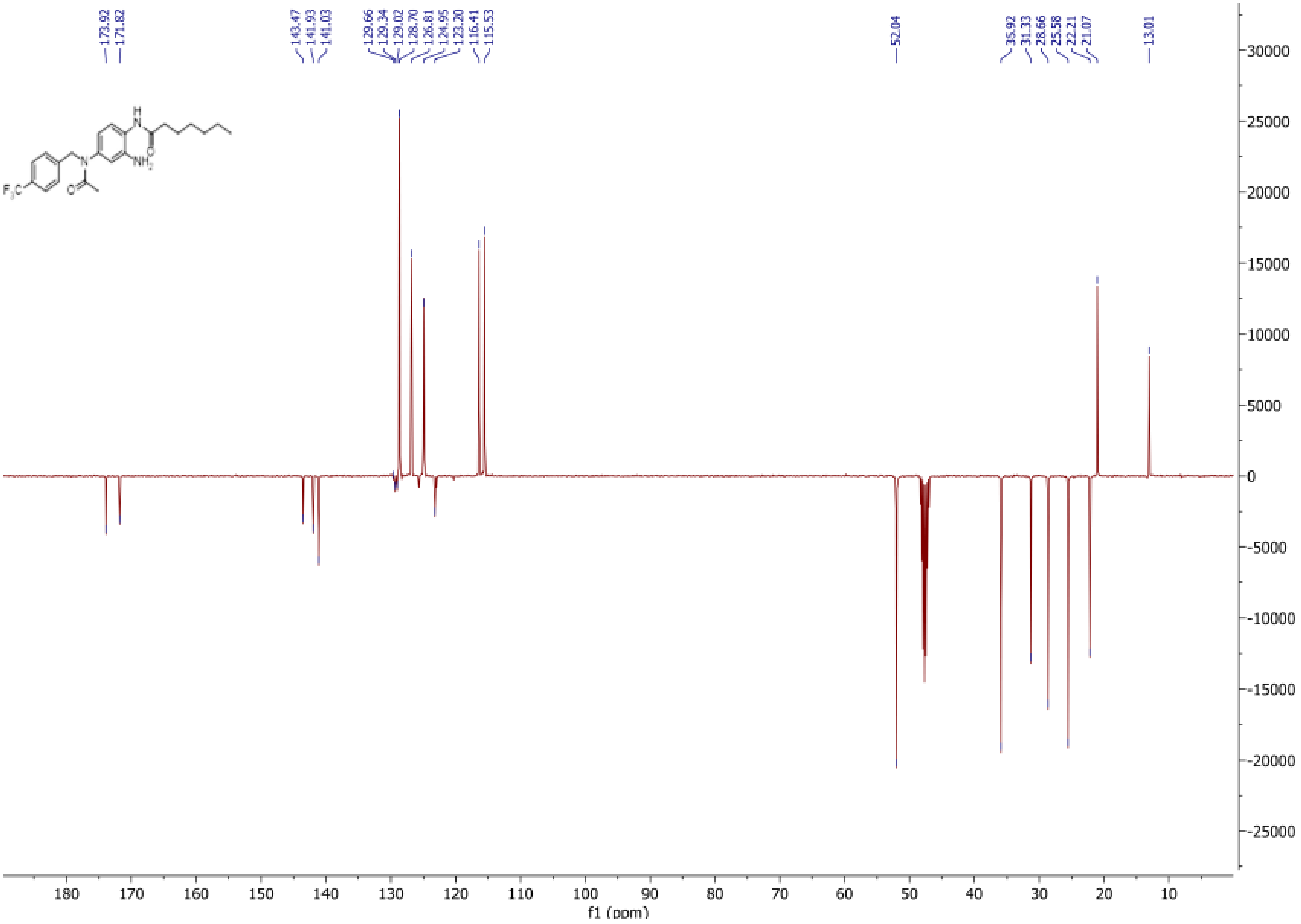

Figure S44: ^{13}C NMR spectra of derivative **28**

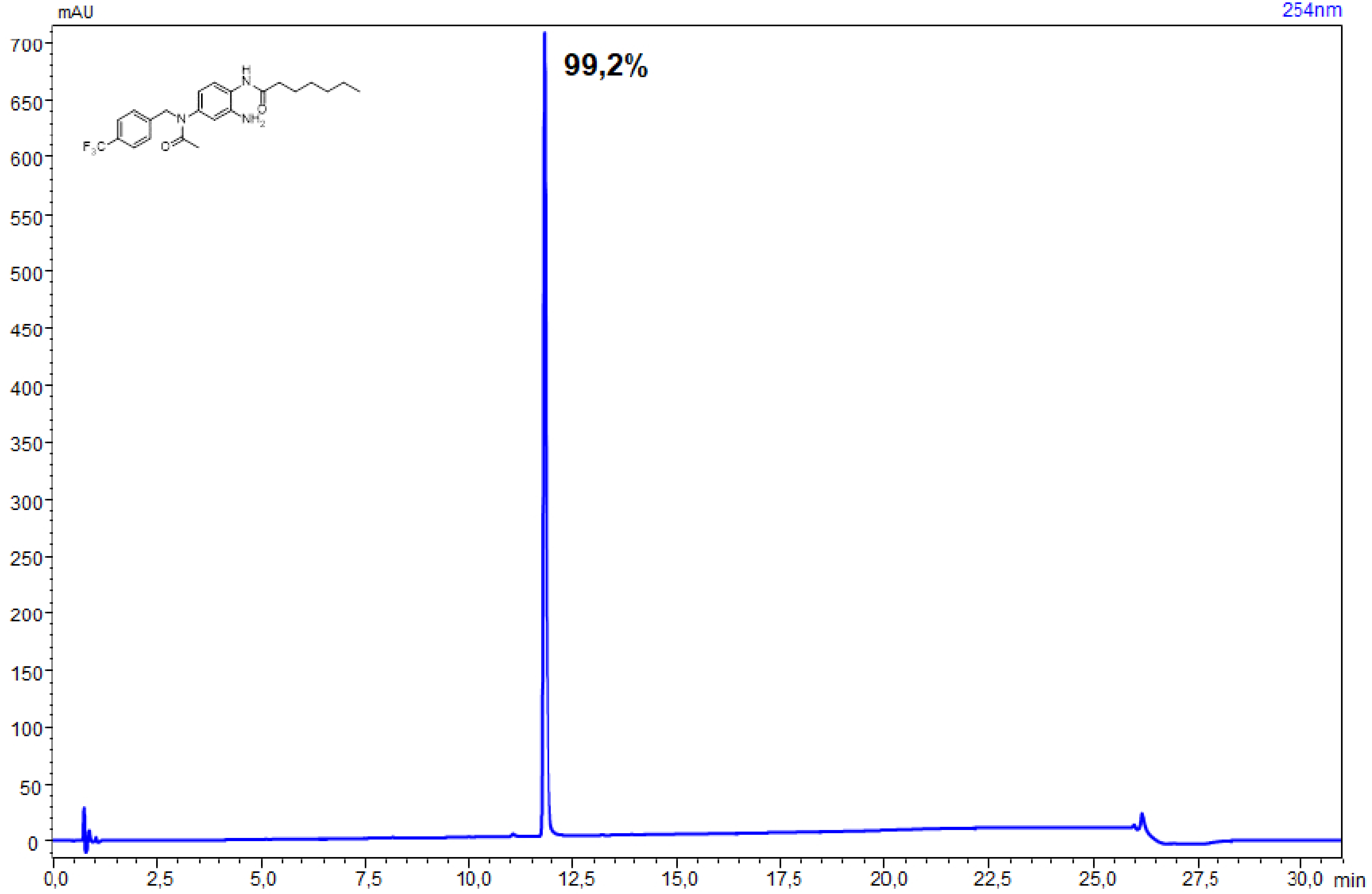

Figure S45: HPLC trace of derivative **28**

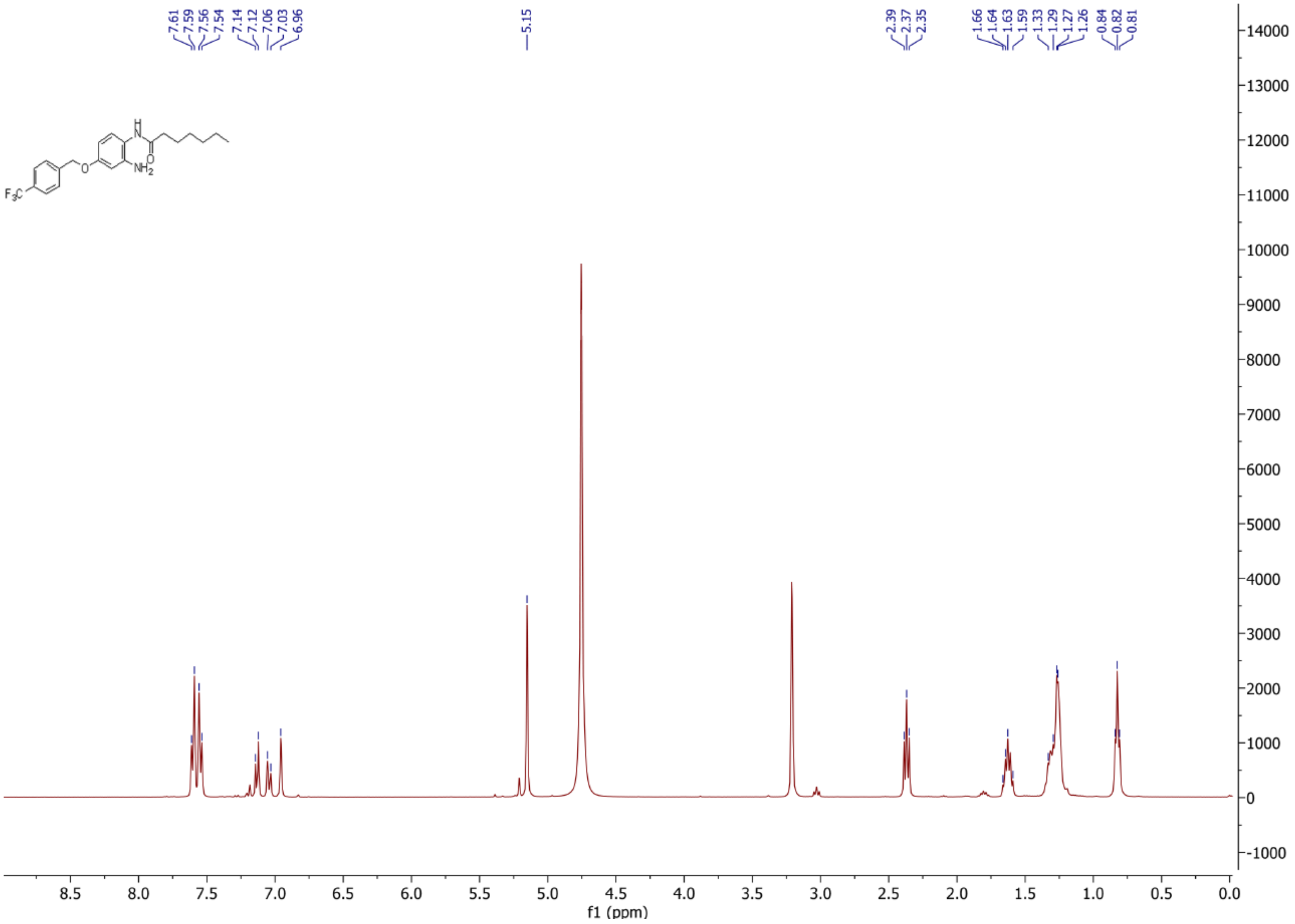

Figure S46: ^{1}H NMR spectra of derivative **31**

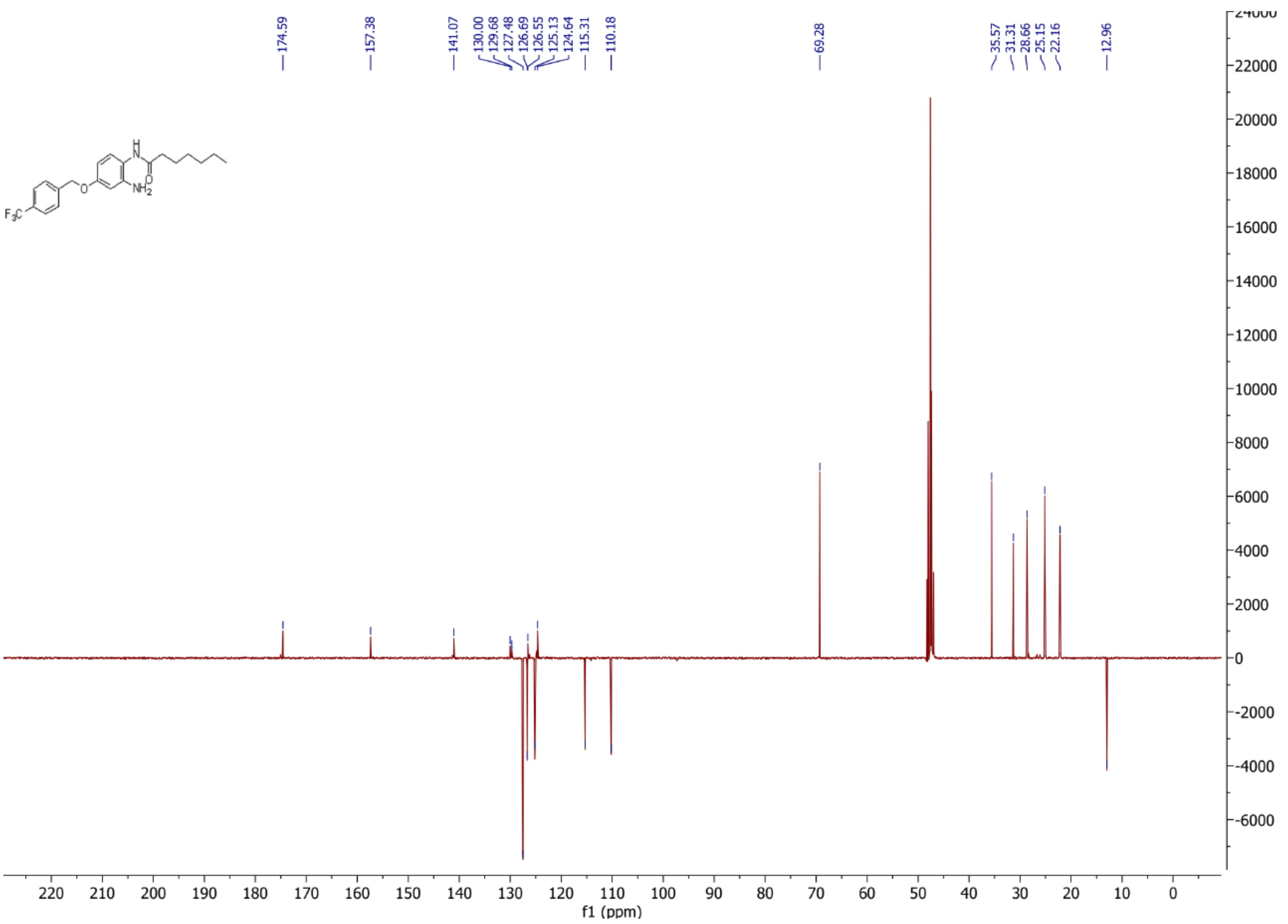

Figure S47: DEPT NMR spectra of derivative **31**

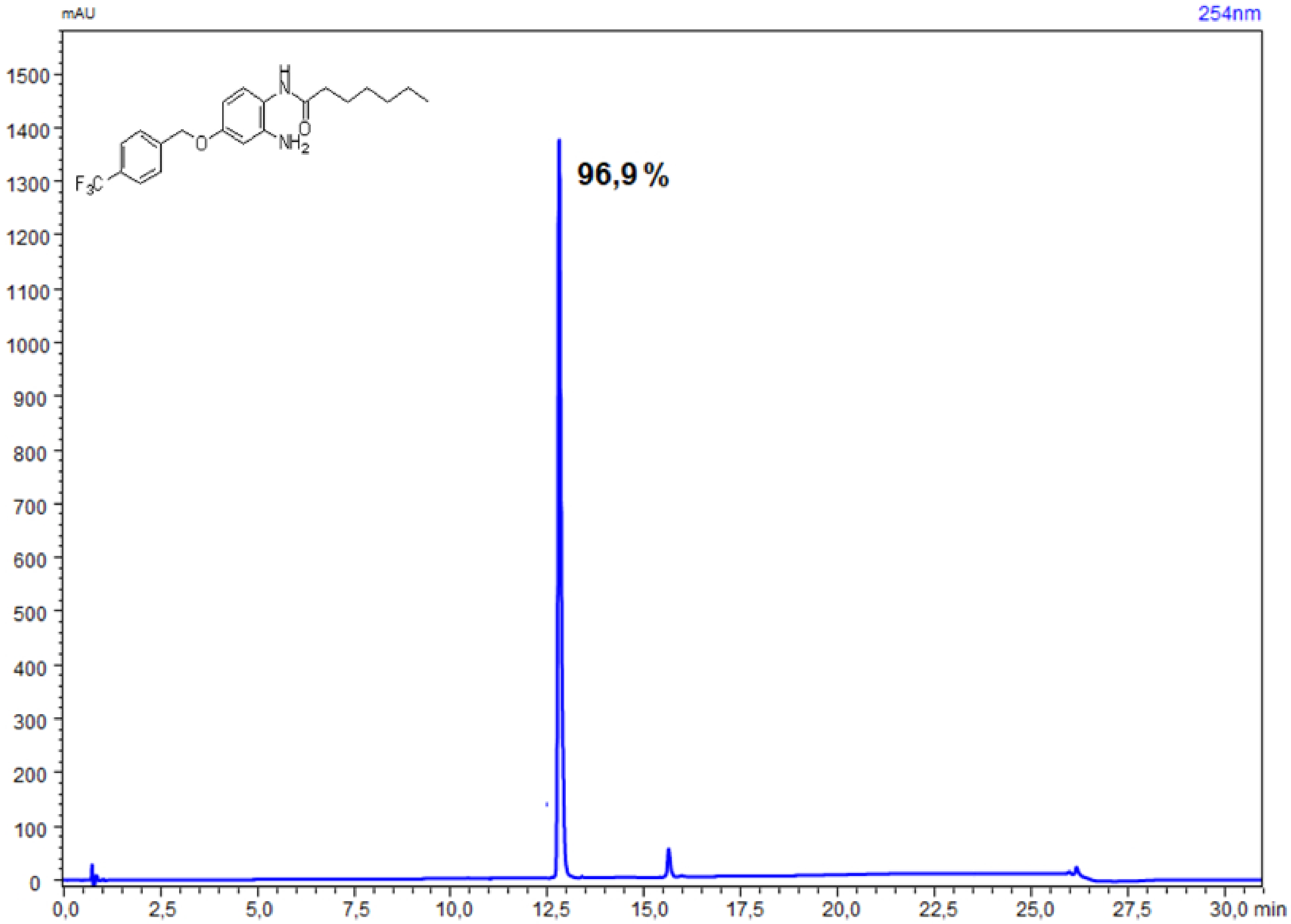

Figure S48: HPLC trace of derivative **31**

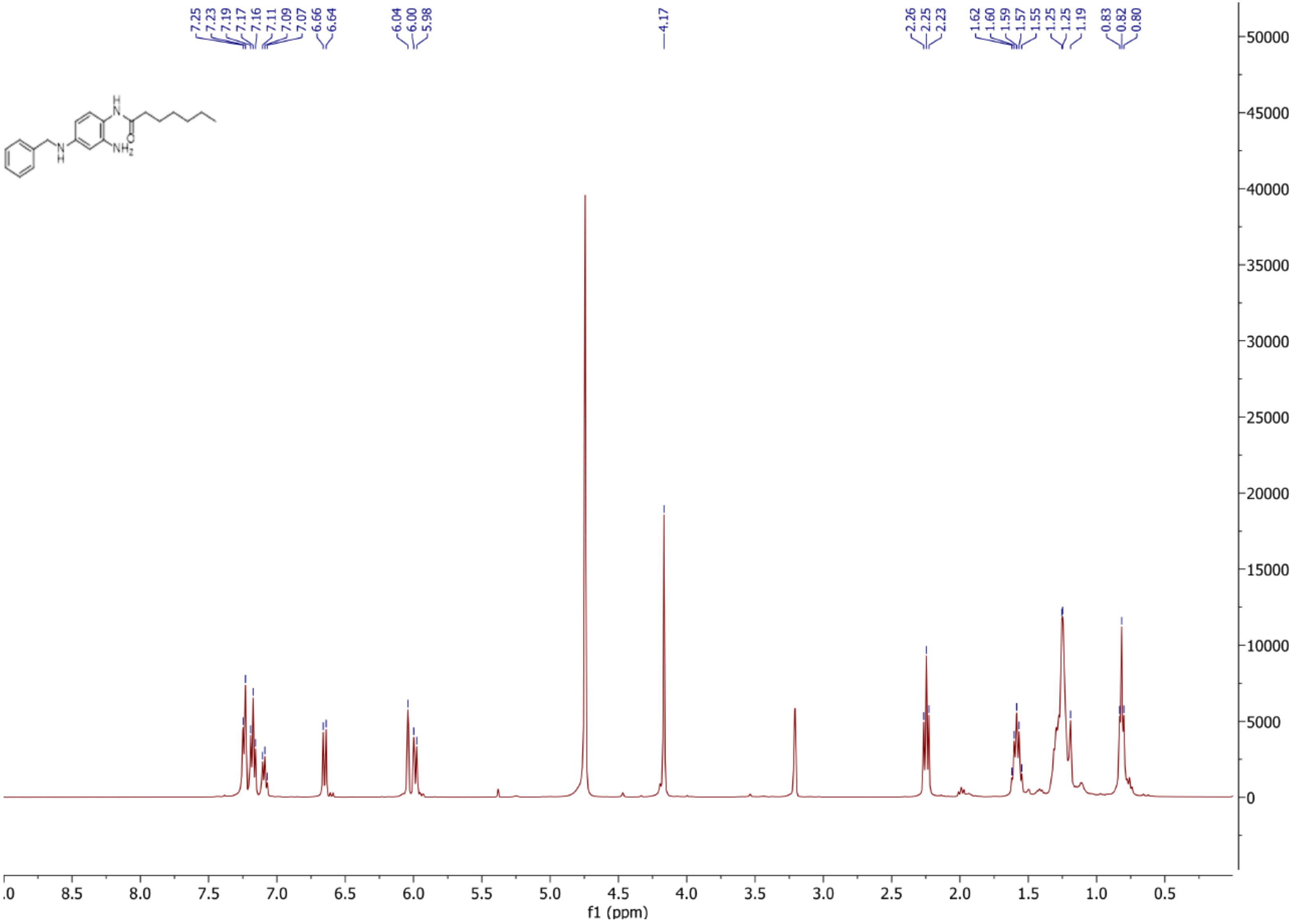

Figure S49: ^{1}H NMR spectra of derivative **41**

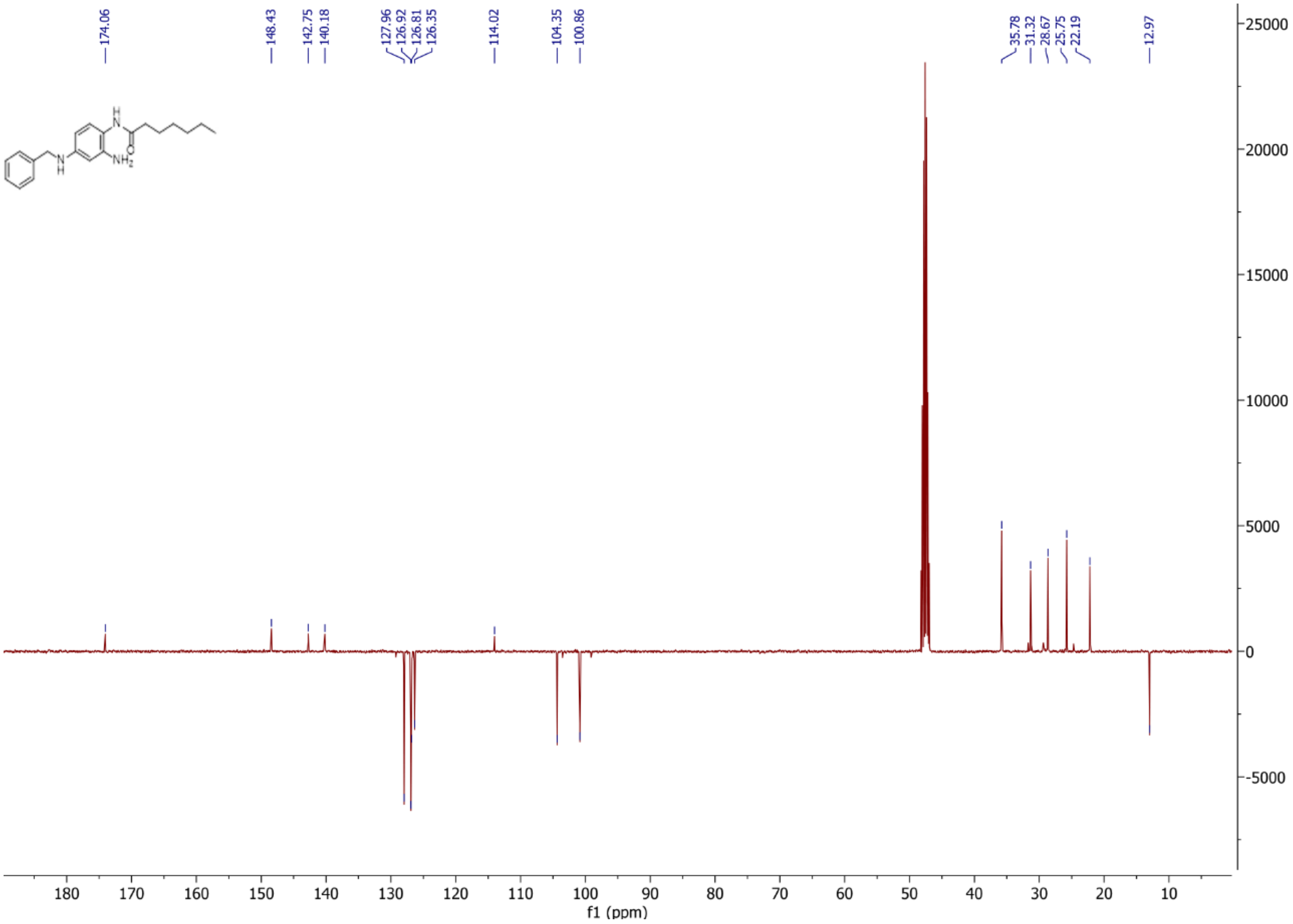

Figure S50: DEPT NMR spectra of derivative **41**

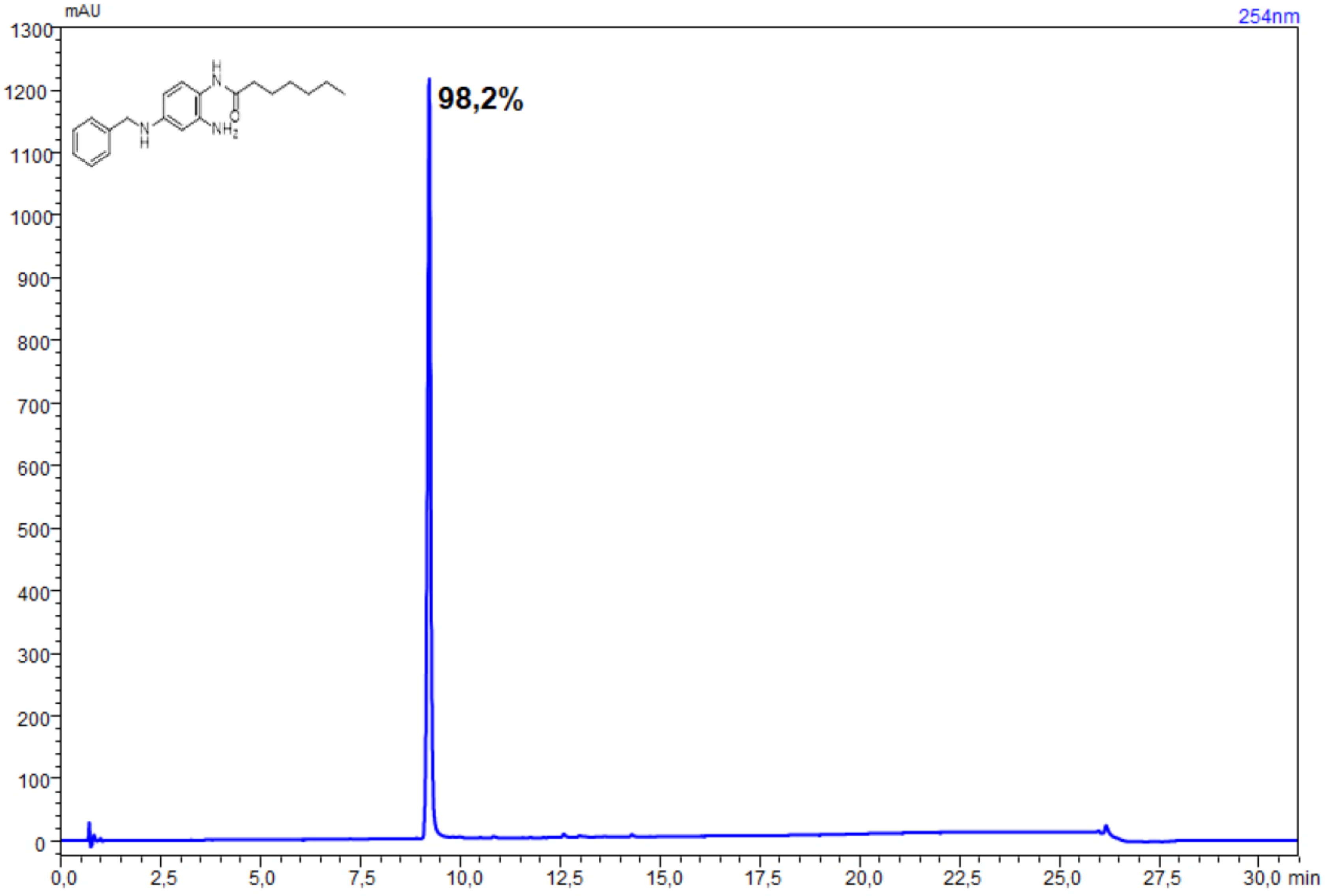

Figure S51: HPLC trace of derivative **41**

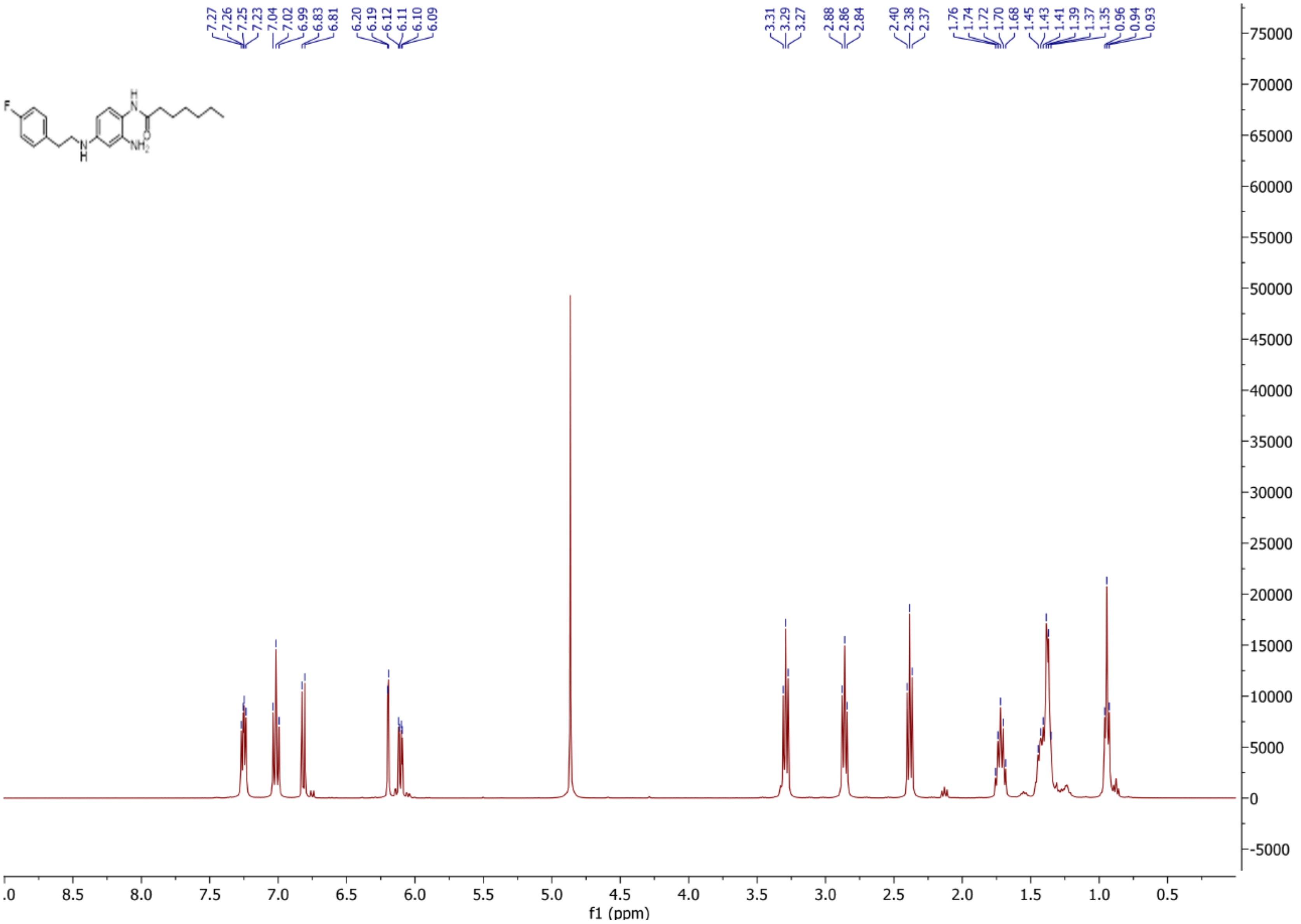

Figure S52: ^{1}H NMR spectra of derivative **42**

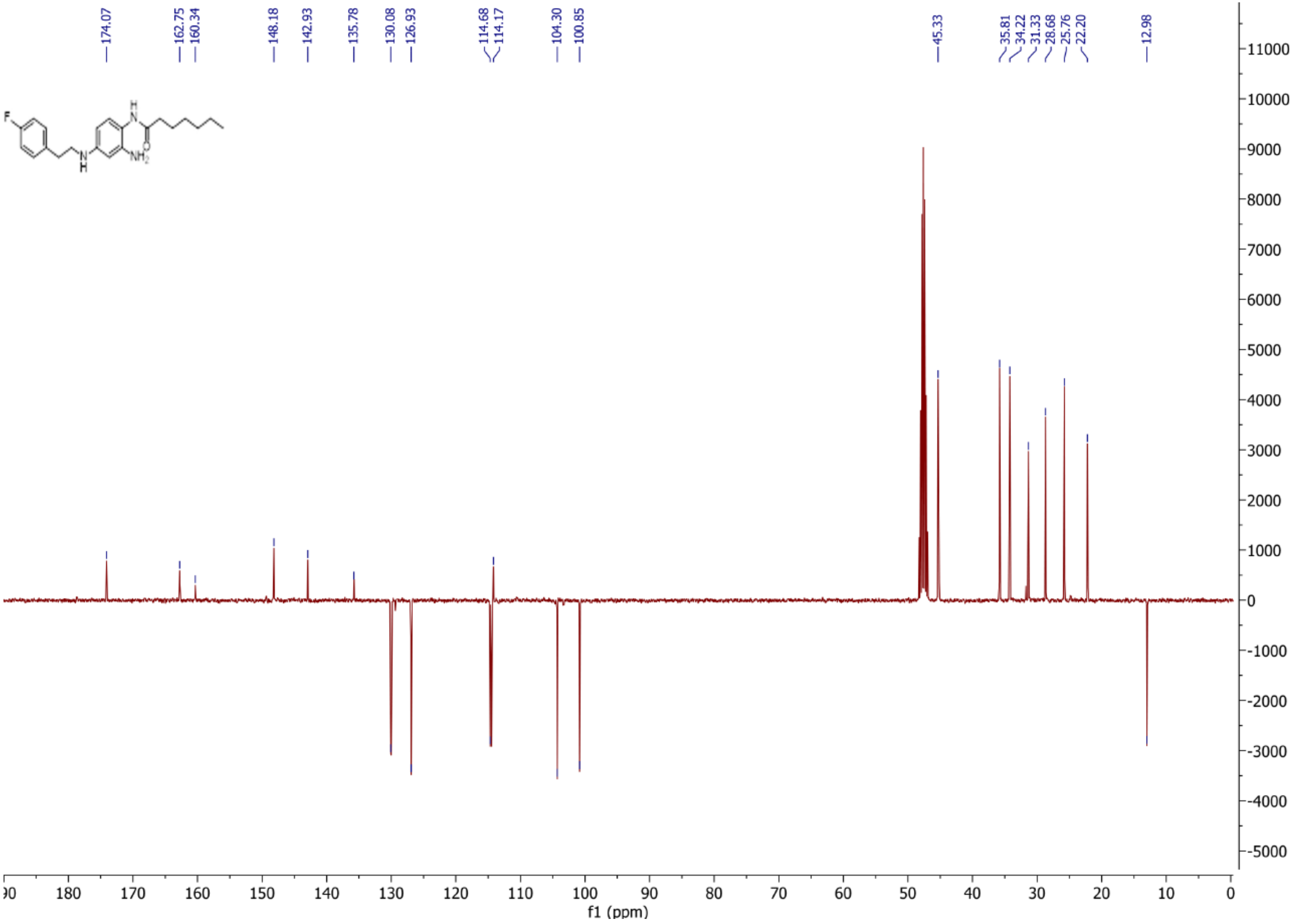

Figure S53: DEPT NMR spectra of derivative **42**

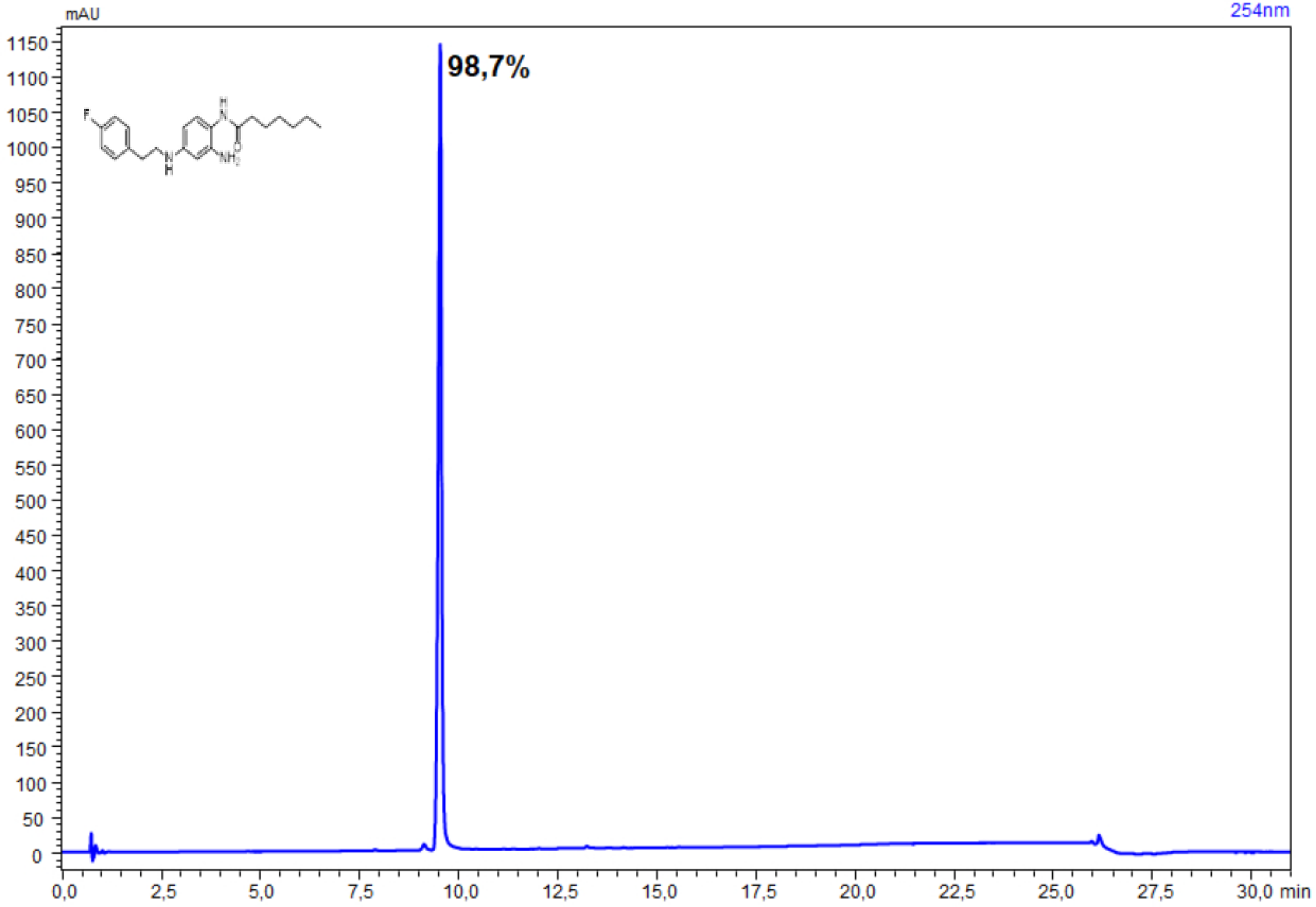

Figure S54: HPLC trace of derivative **42**

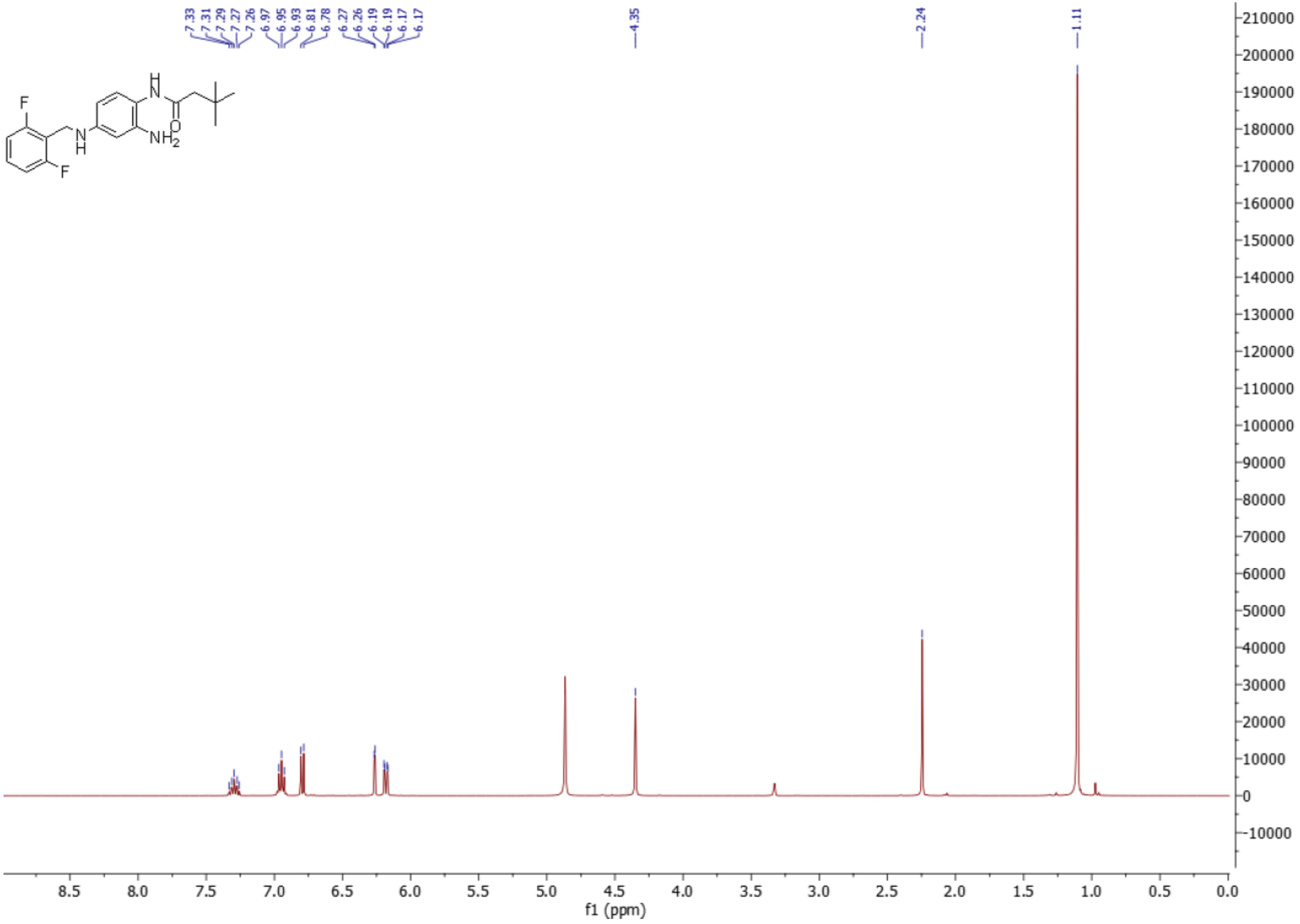

Figure S55: ^{1}H NMR trace of derivative **43**

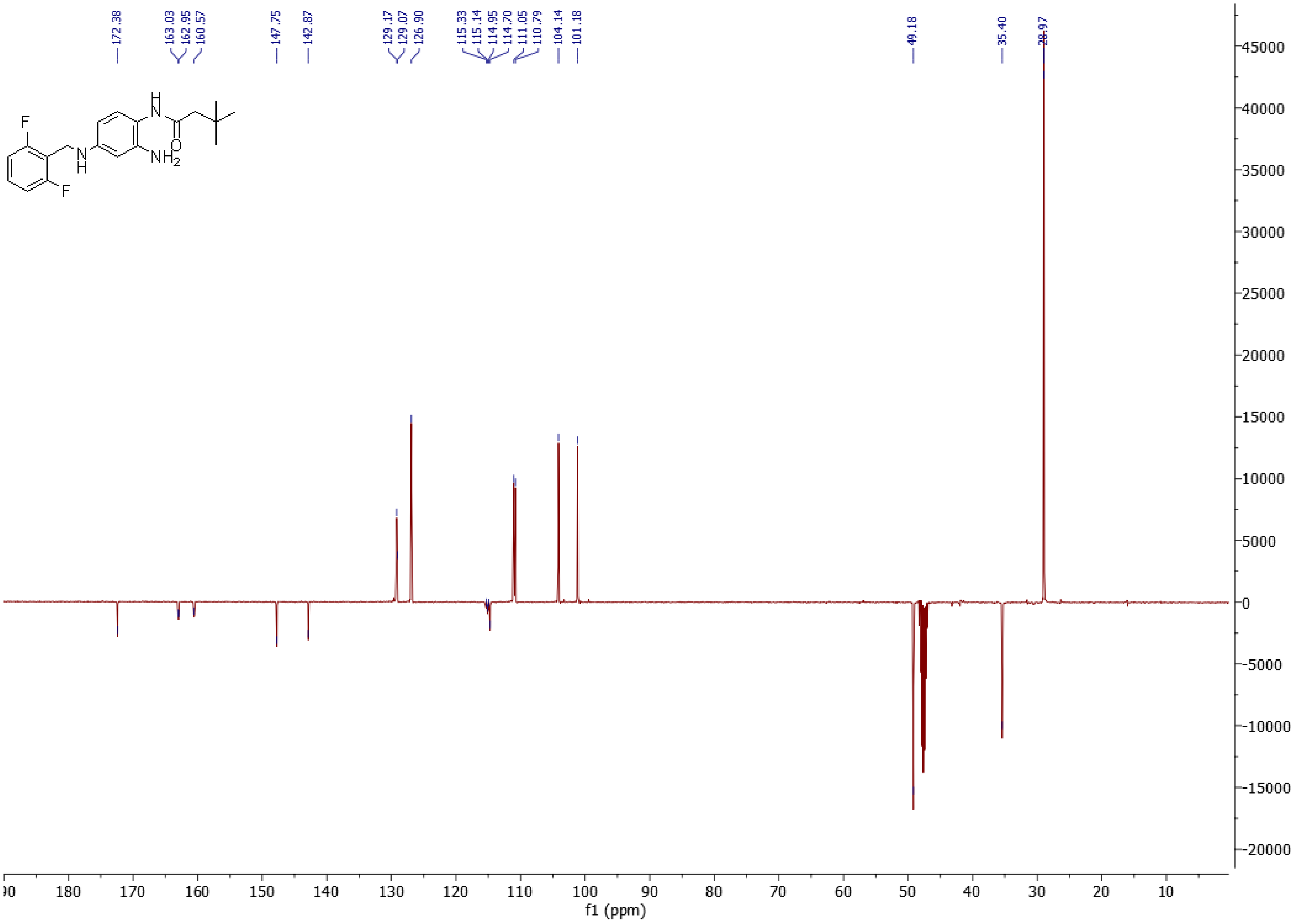

Figure S56: DEPT NMR spectra of derivative **43**

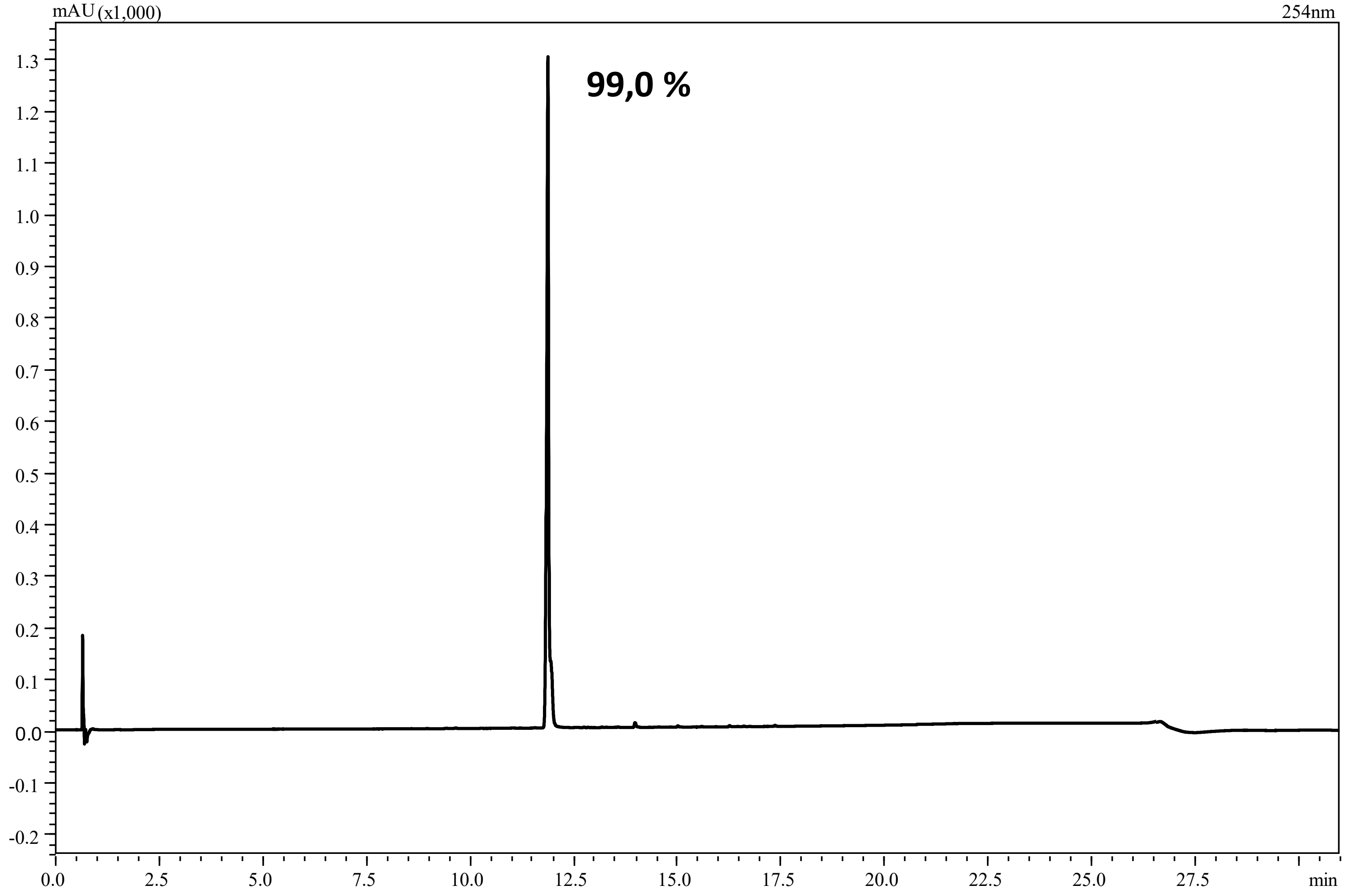

Figure S57: HPLC trace of derivative **43**

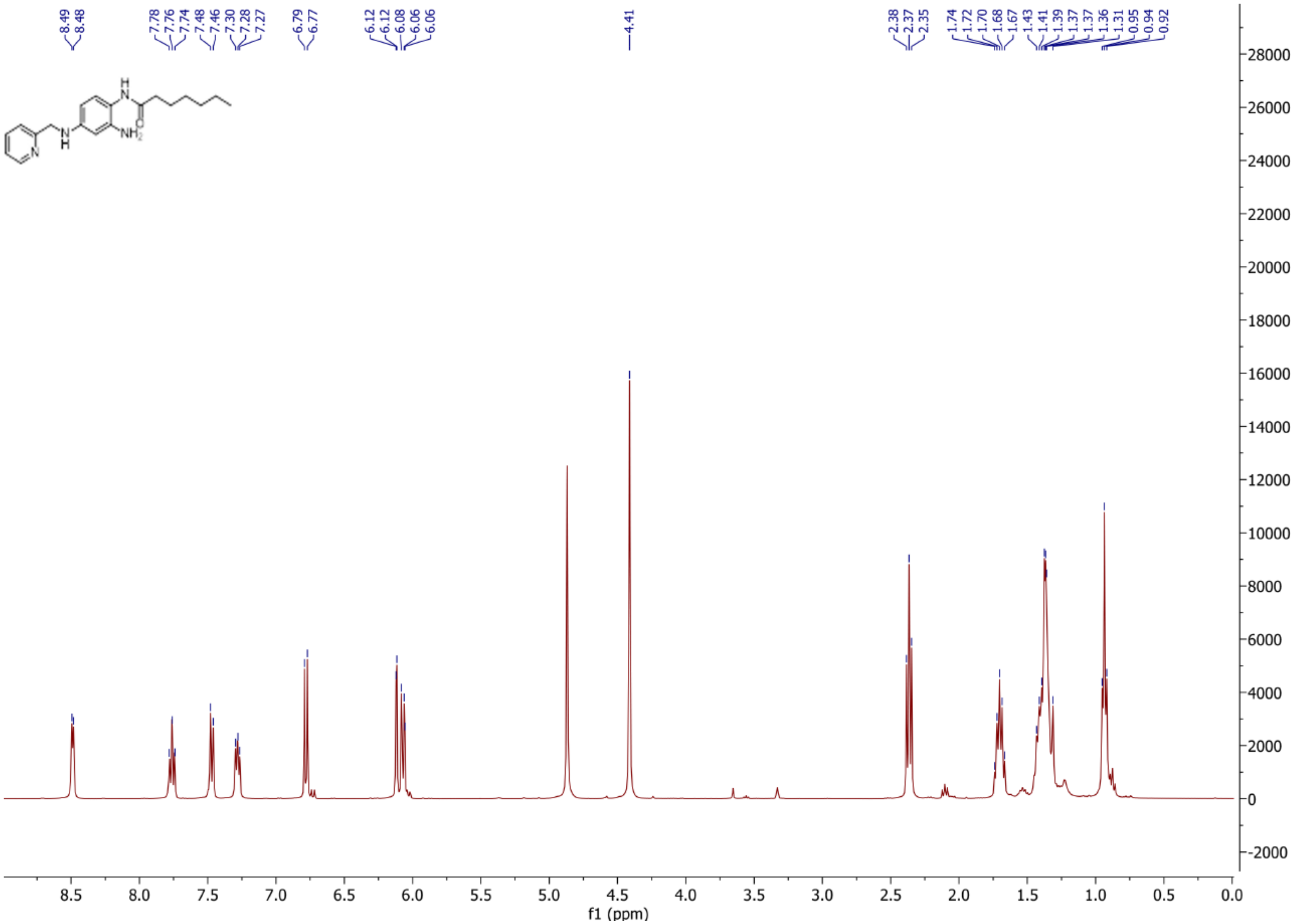

Figure S58: ^{1}H NMR spectra of derivative **47**

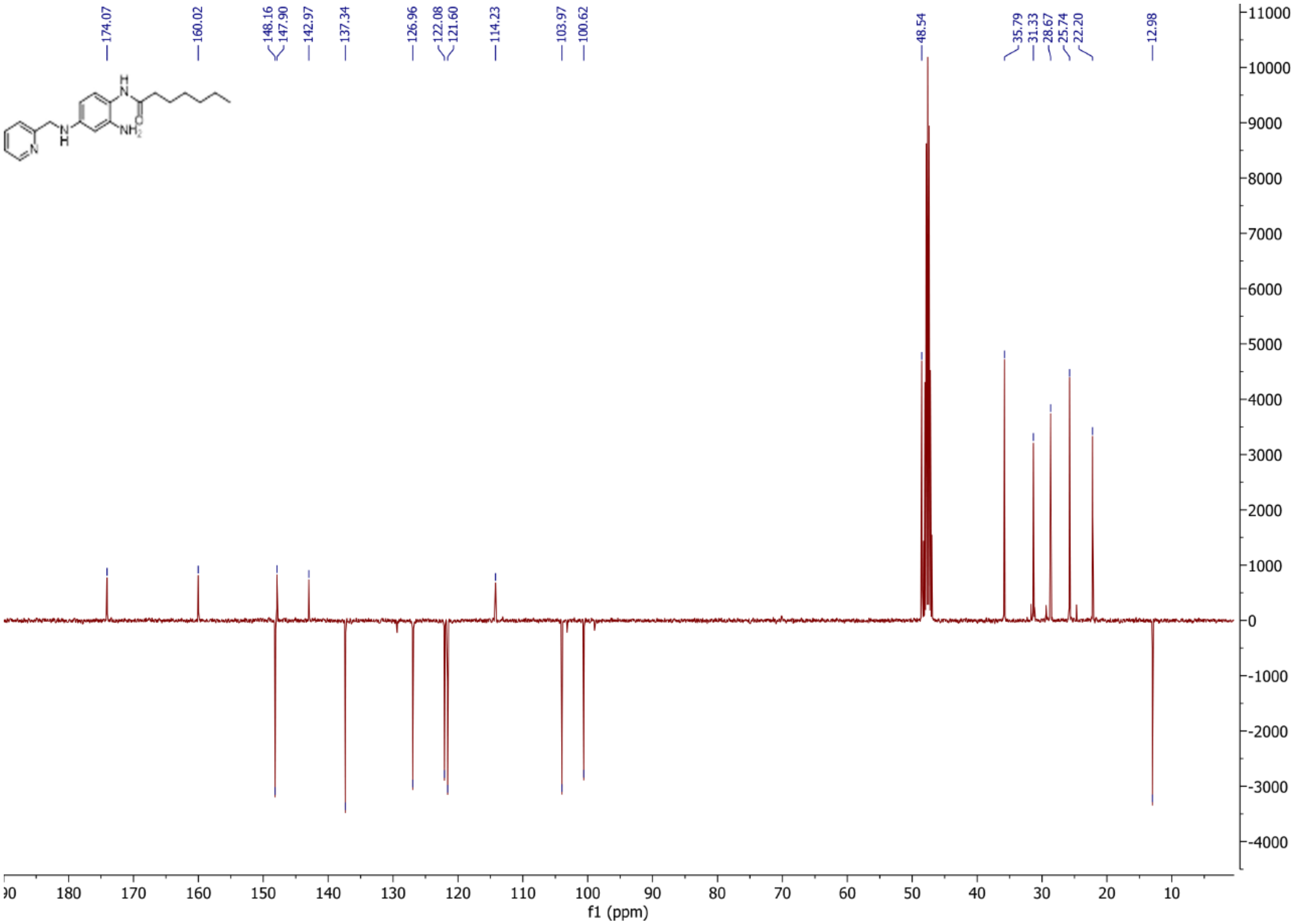

Figure S59: DEPT NMR spectra of derivative **47**

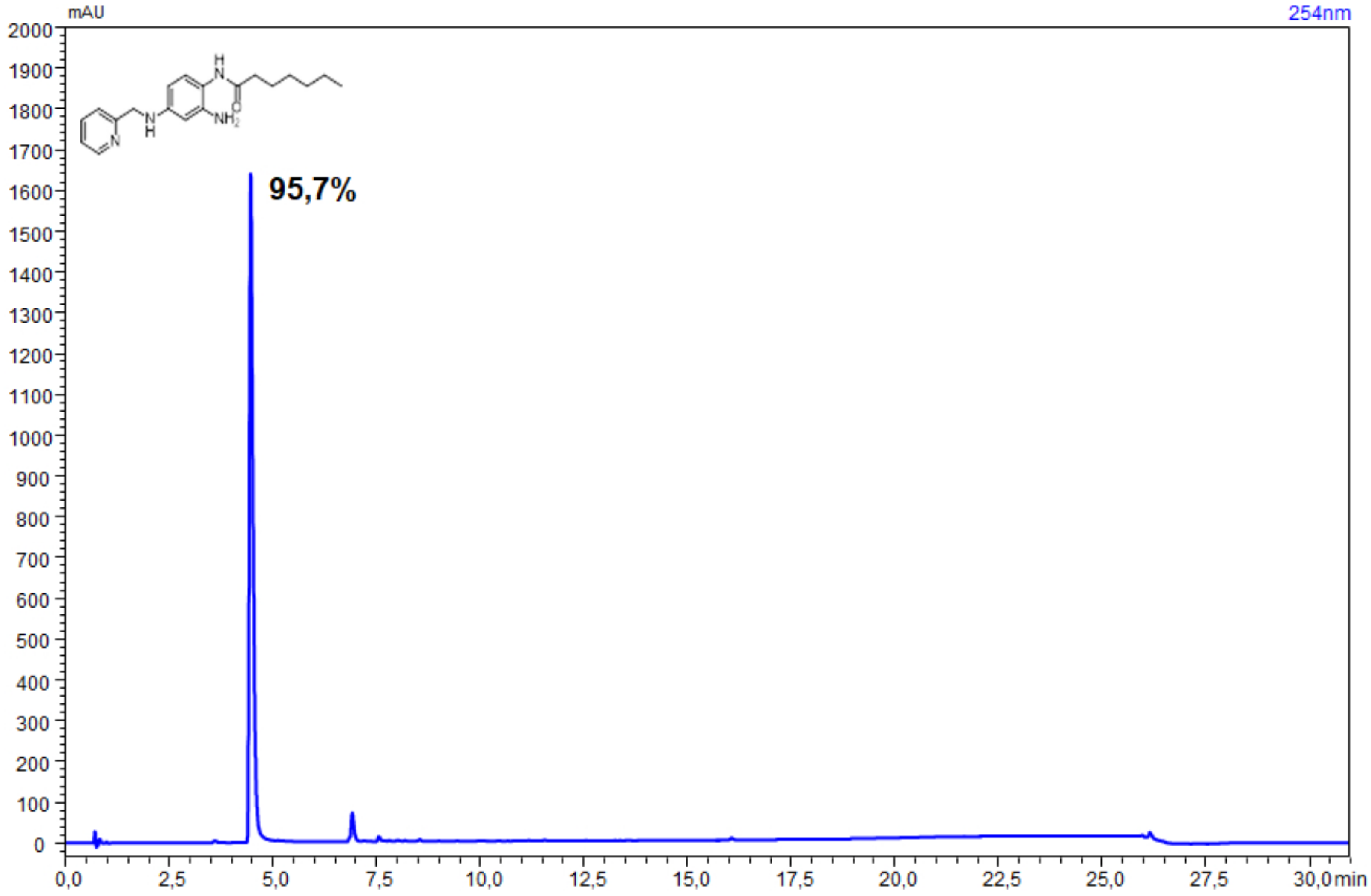

Figure S60: HPLC trace of derivative **47**

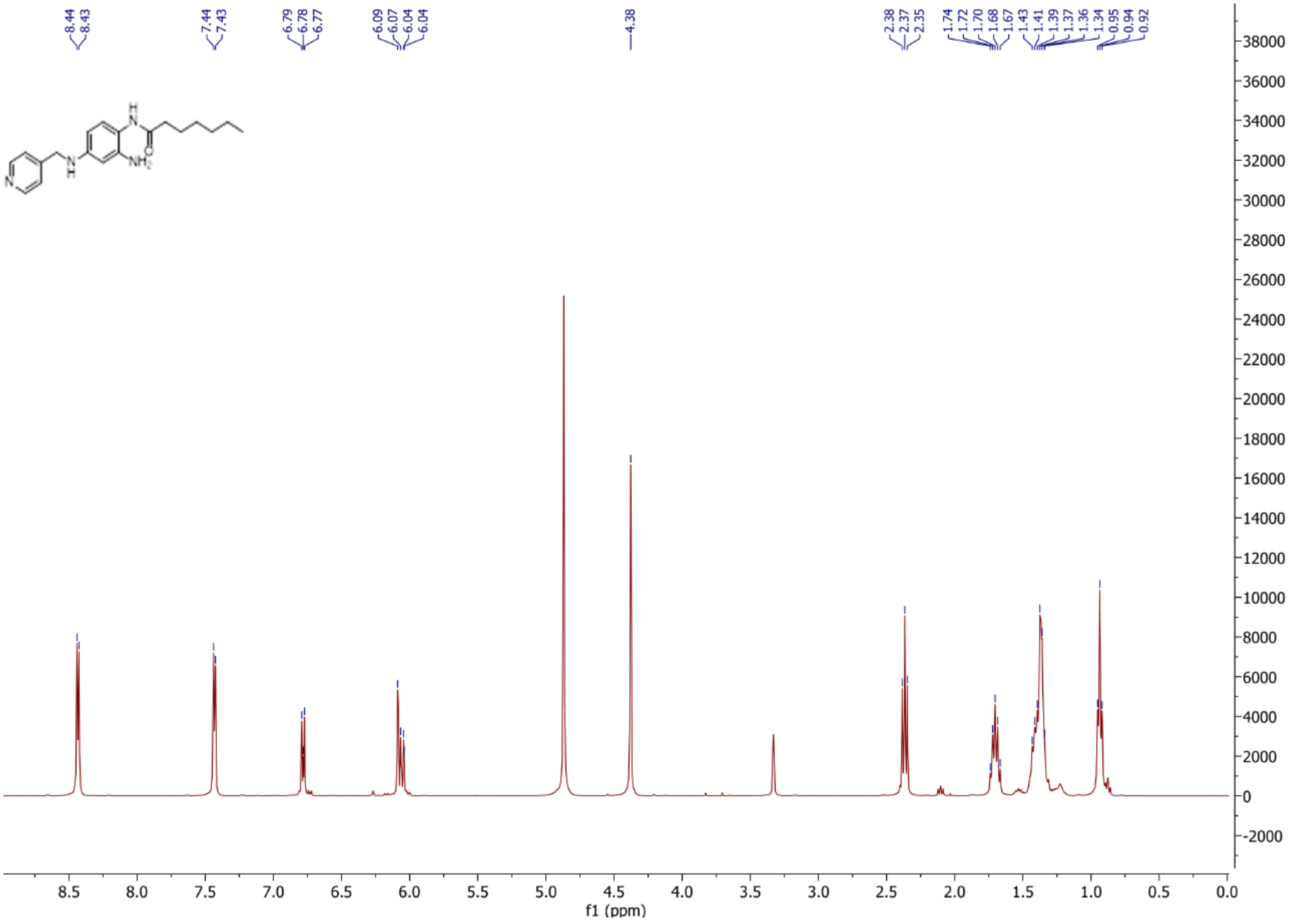

Figure S61: ^{1}H NMR spectra of derivative **51**

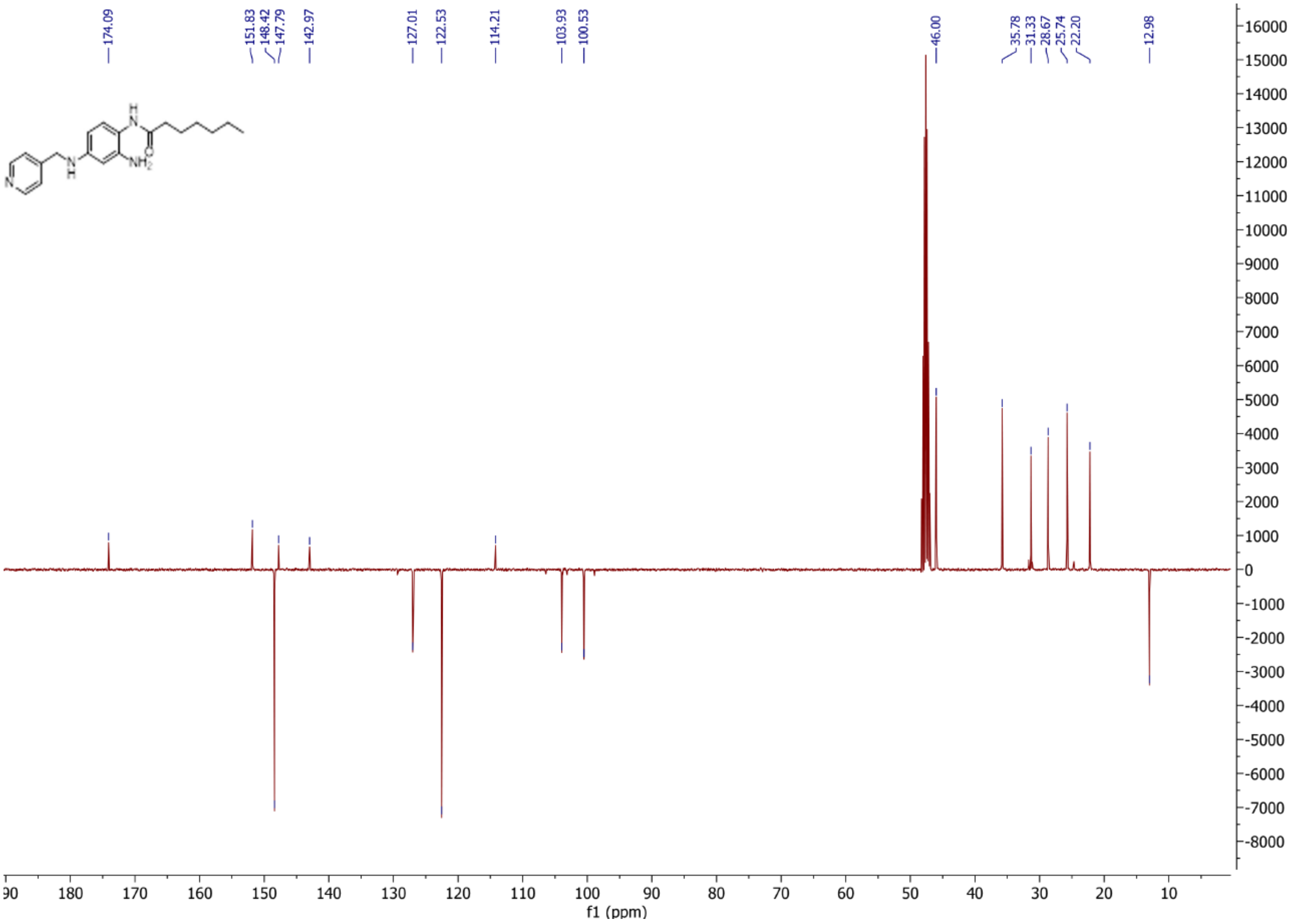

Figure S62: DEPT NMR spectra of derivative **51**

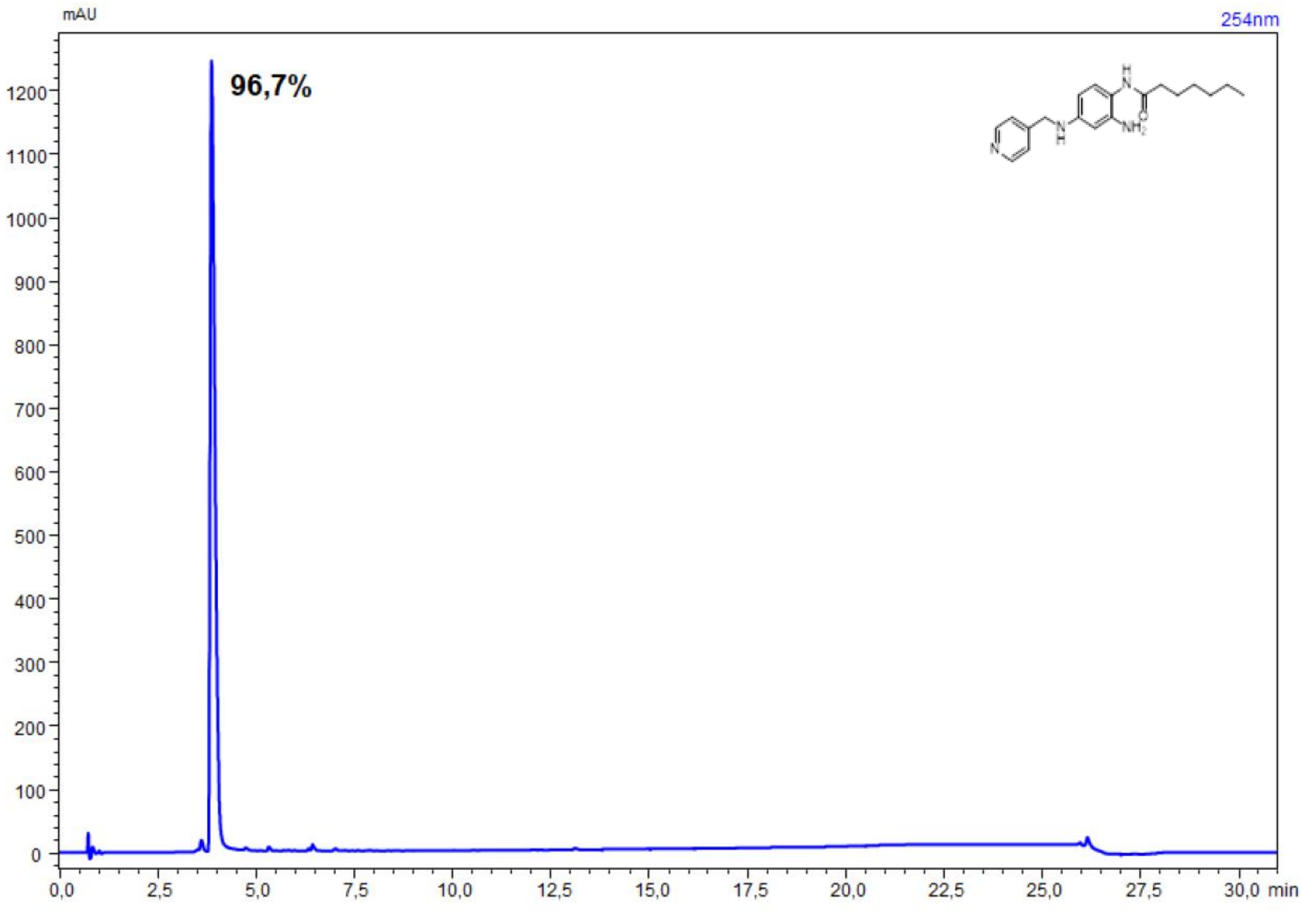

Figure S63: HPLC trace of derivative **51**

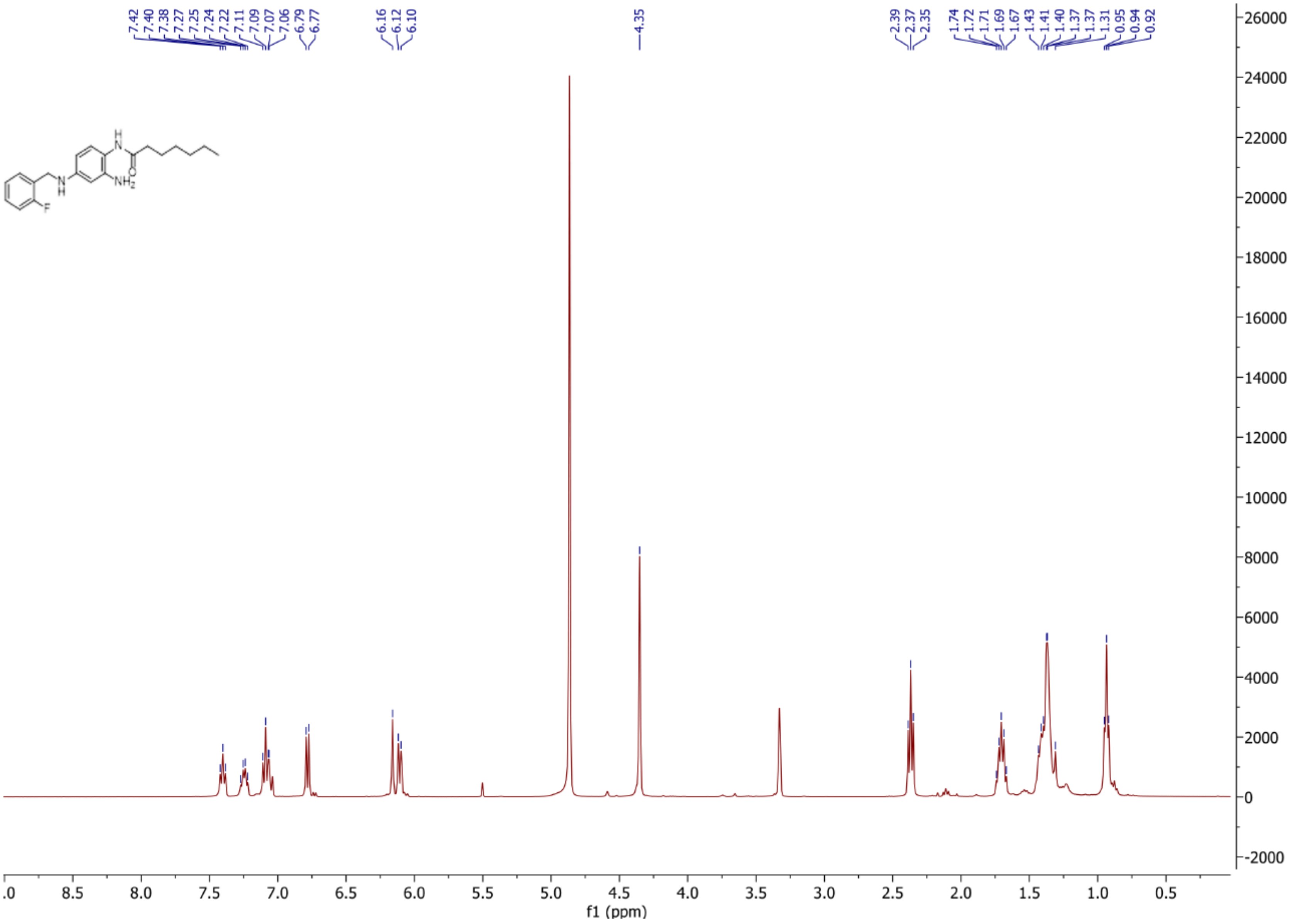

Figure S64: ^{1}H NMR spectra of derivative **52**

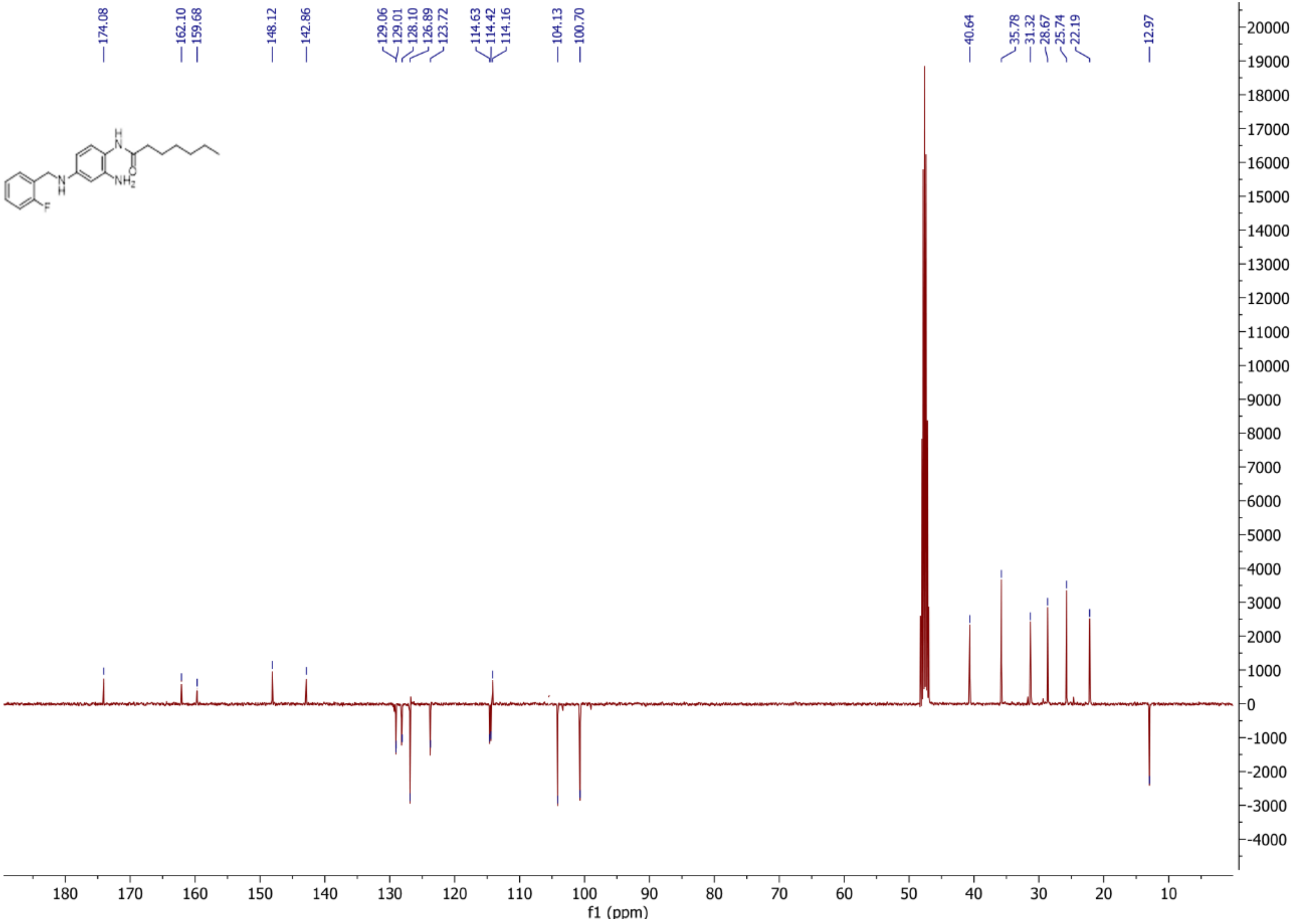

Figure S65: DEPT NMR spectra of derivative **52**

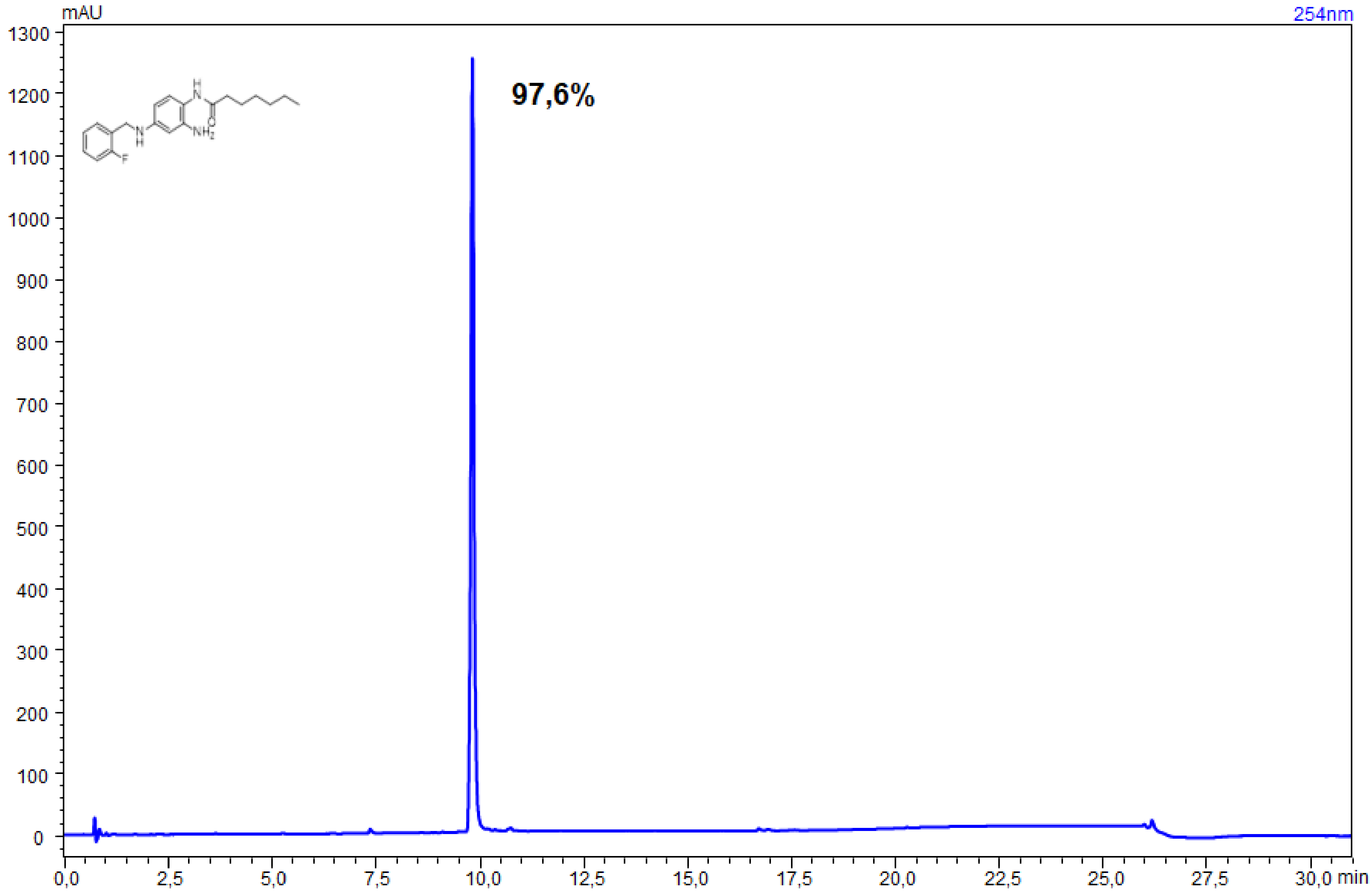

Figure S66: HPLC trace of derivative **52**

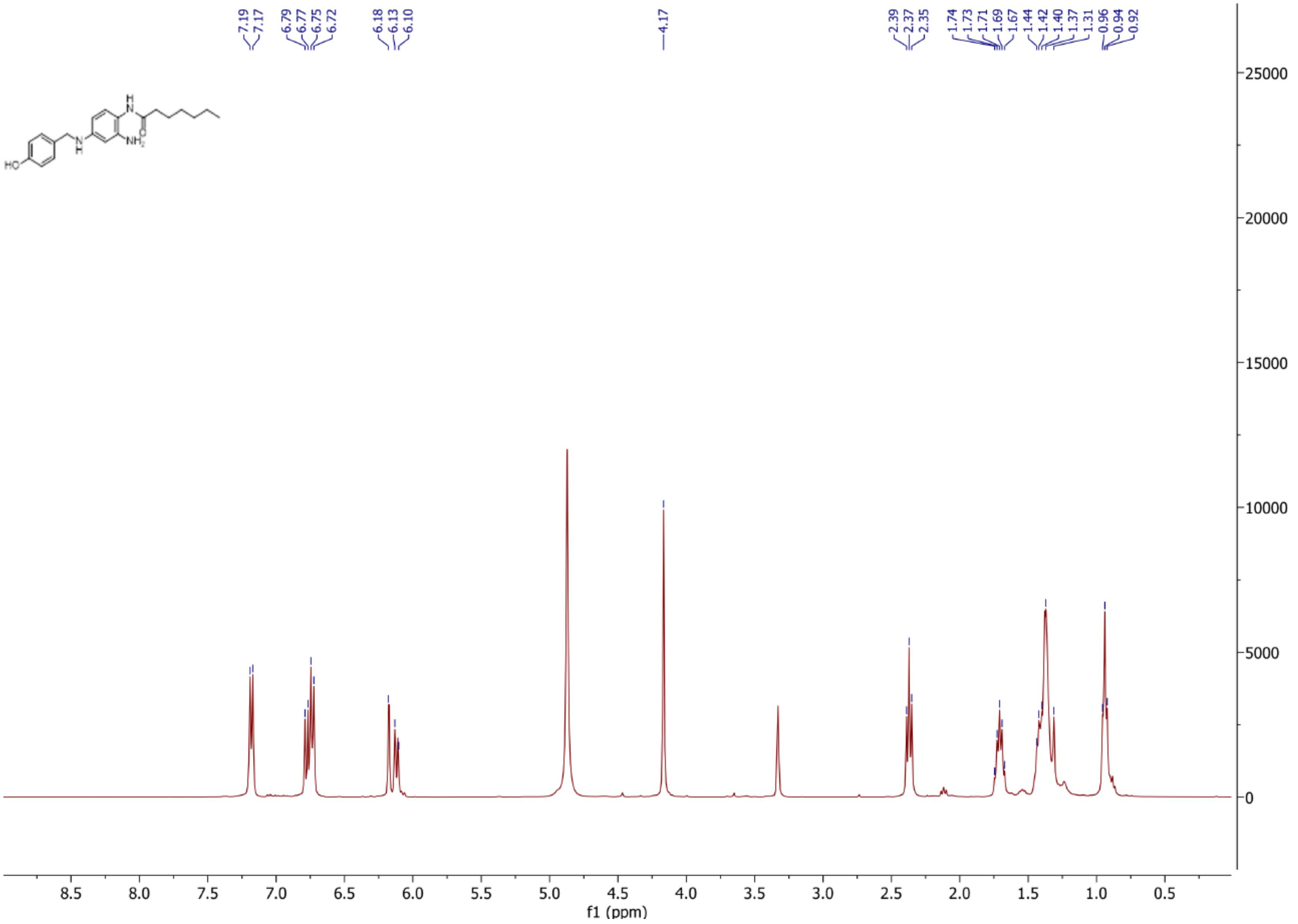

Figure S67:^{1}H NMR spectra of derivative **57**

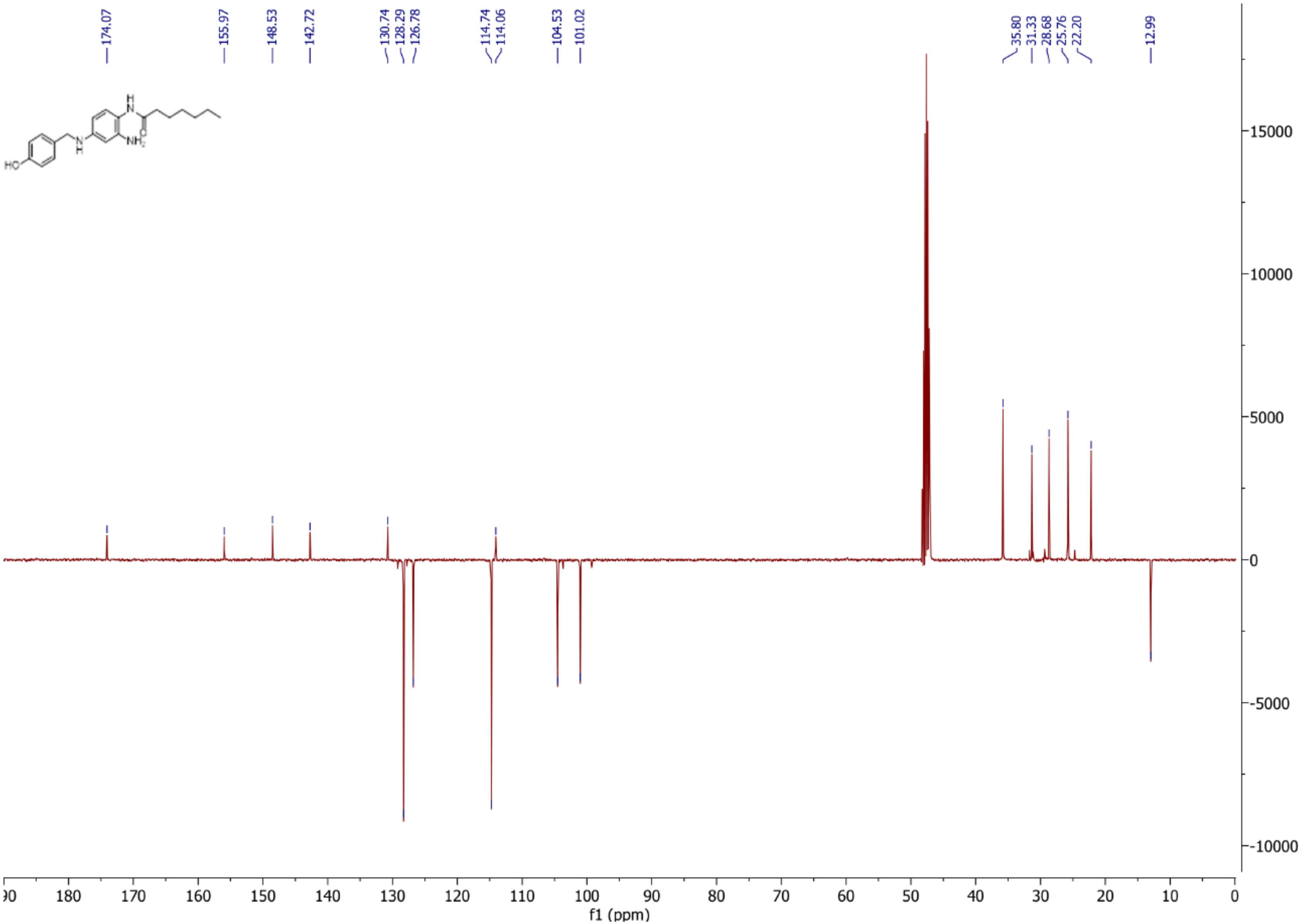

Figure S68: DEPT NMR spectra of derivative **57**

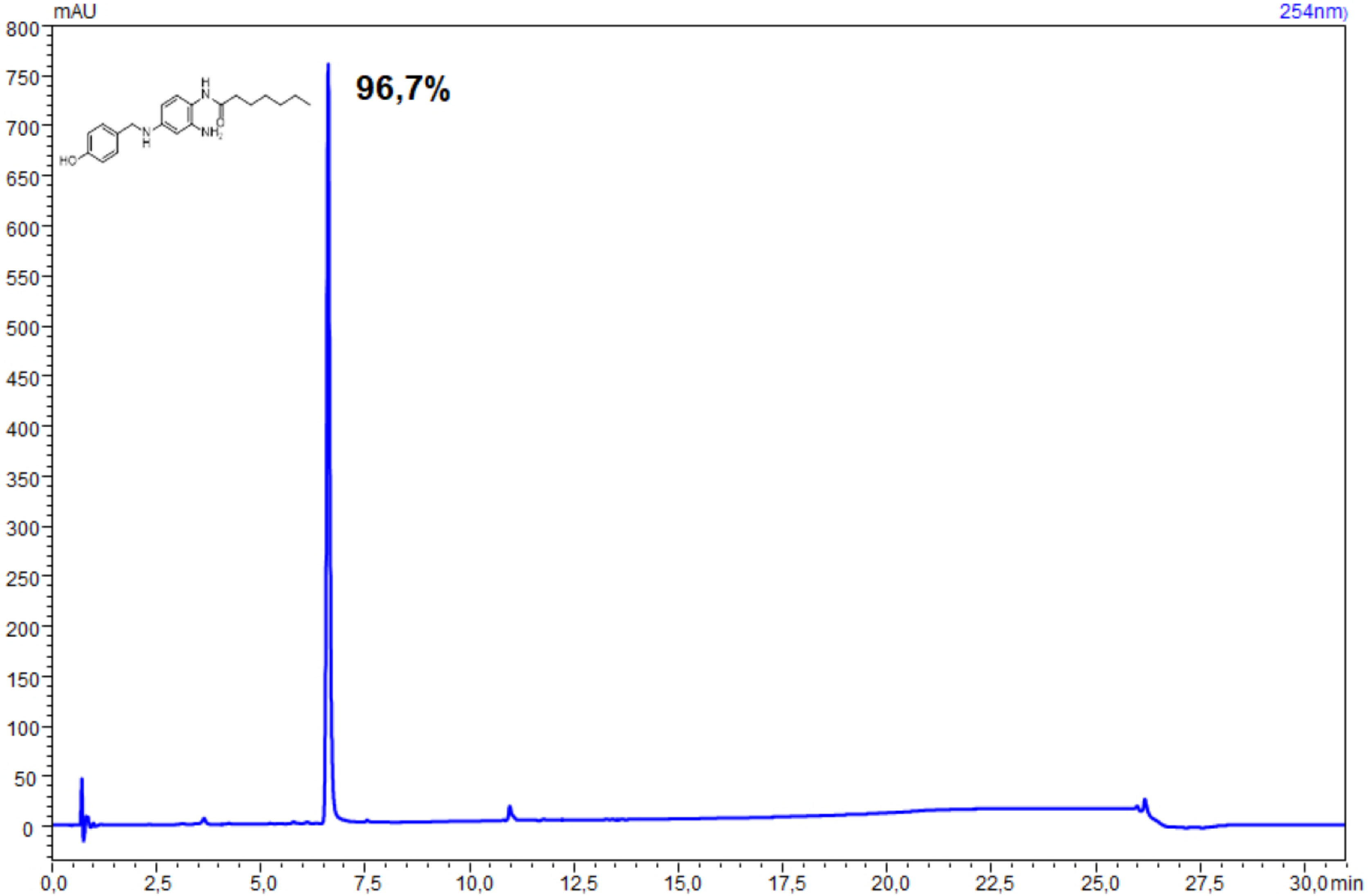

Figure S69: HPLC trace of derivative **57**

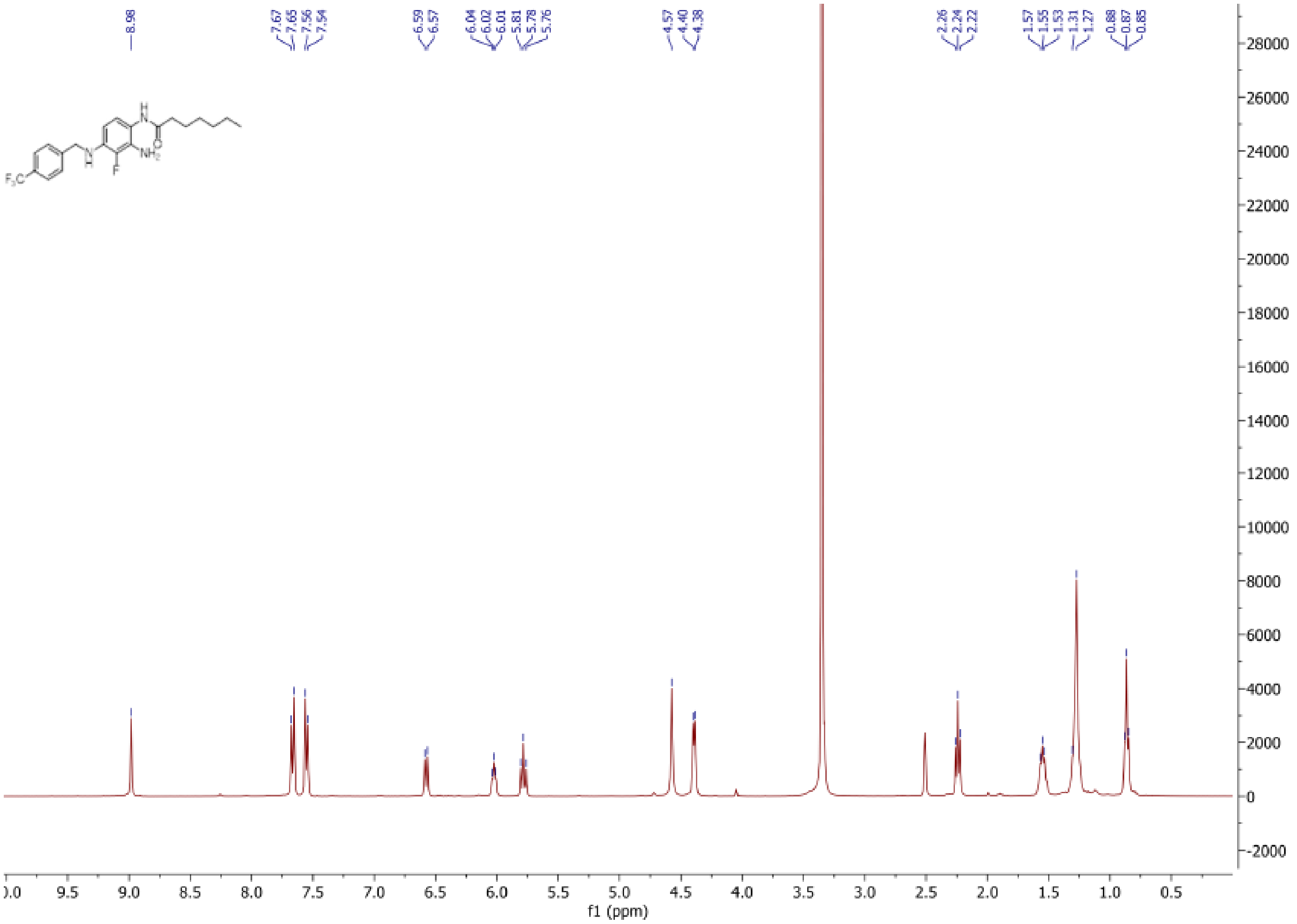

Figure S70: ^{1}H NMR spectra of derivative **59**

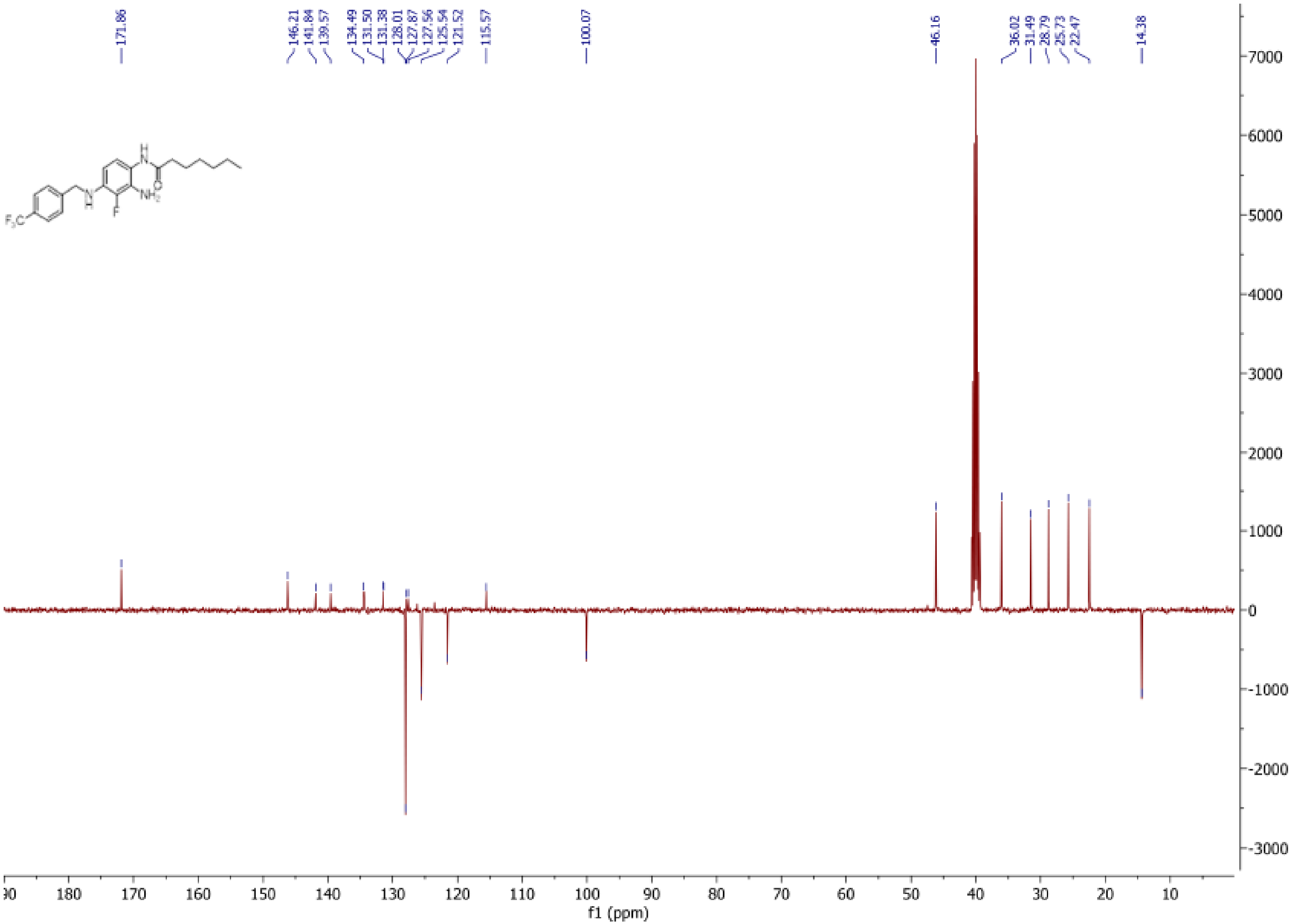

Figure S71: DEPT NMR spectra of derivative **59**

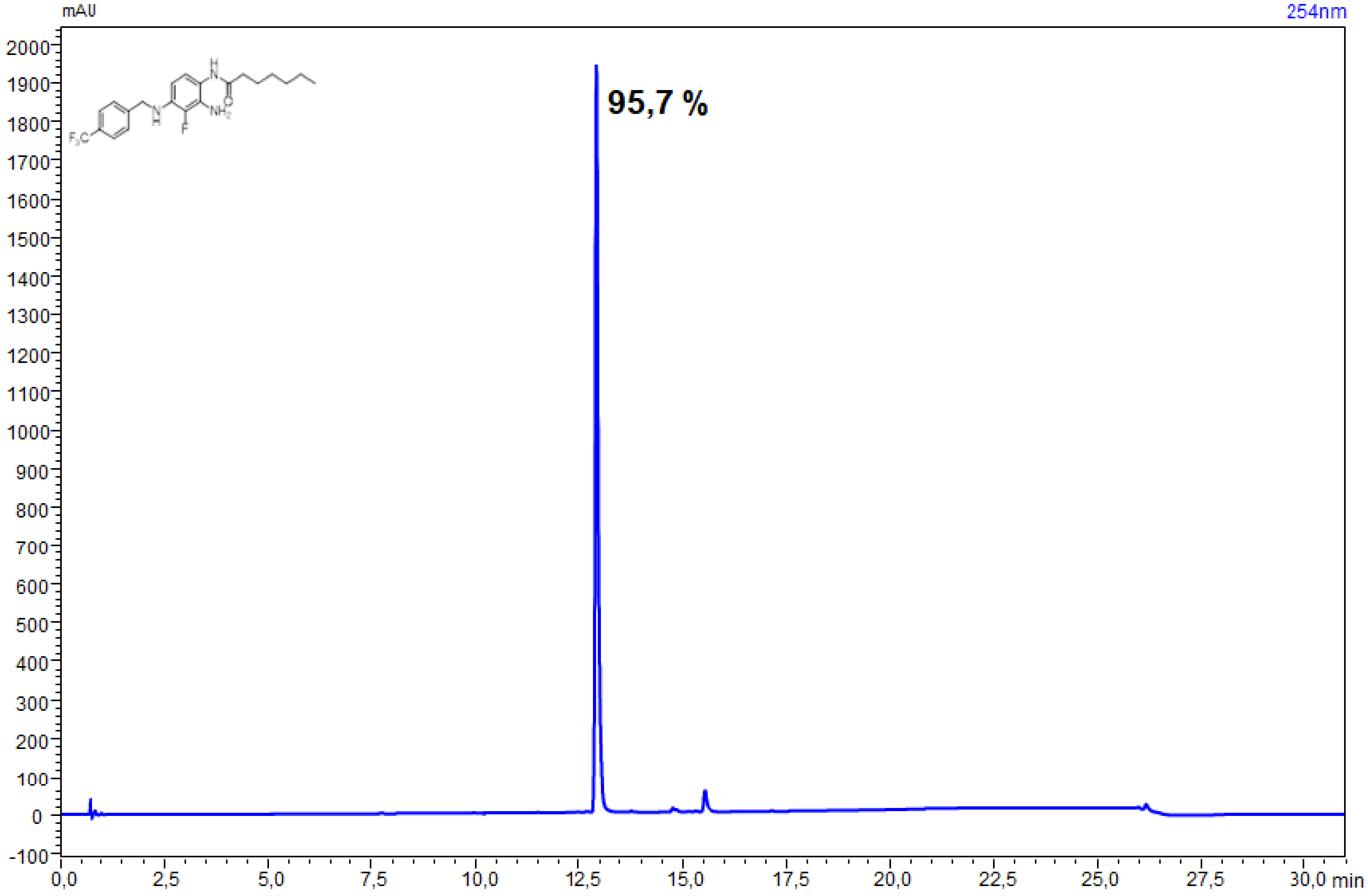

Figure S72: HPLC trace of derivative **59**

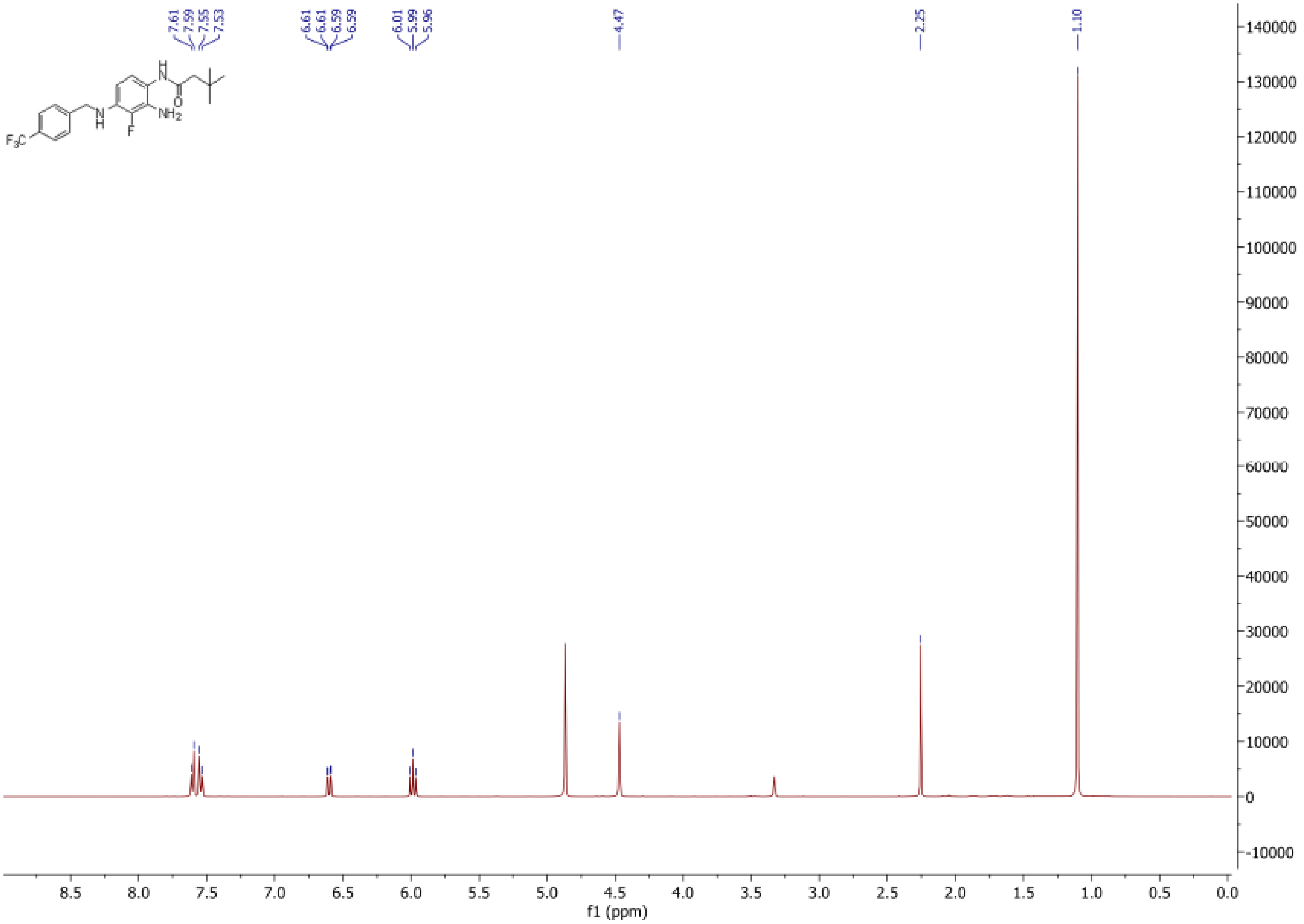

Figure S73: ^{1}H NMR spectra of derivative **60**

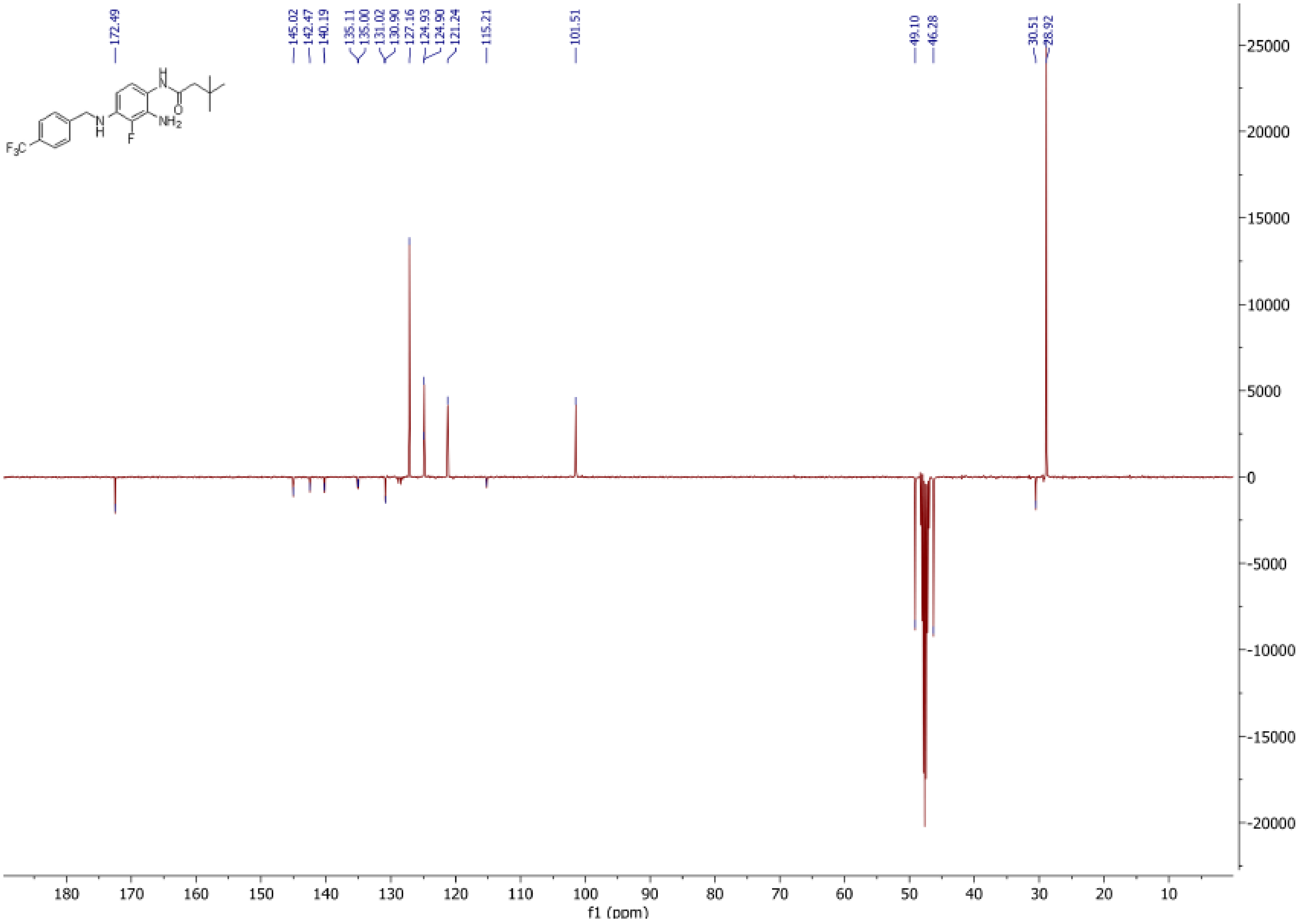

Figure S74: DEPT NMR spectra of derivative **60**

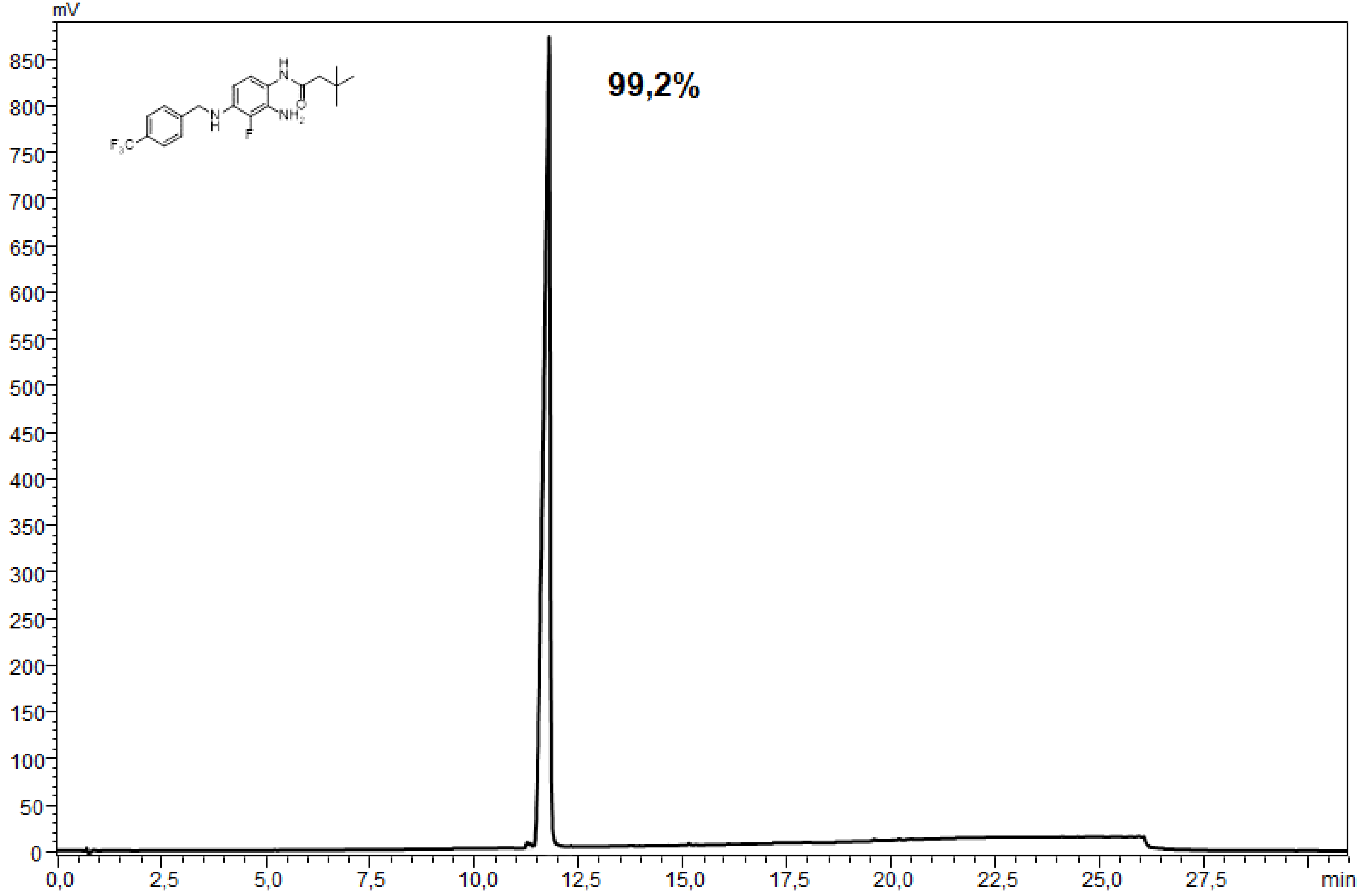

Figure S75: HPLC trace of derivative **60**

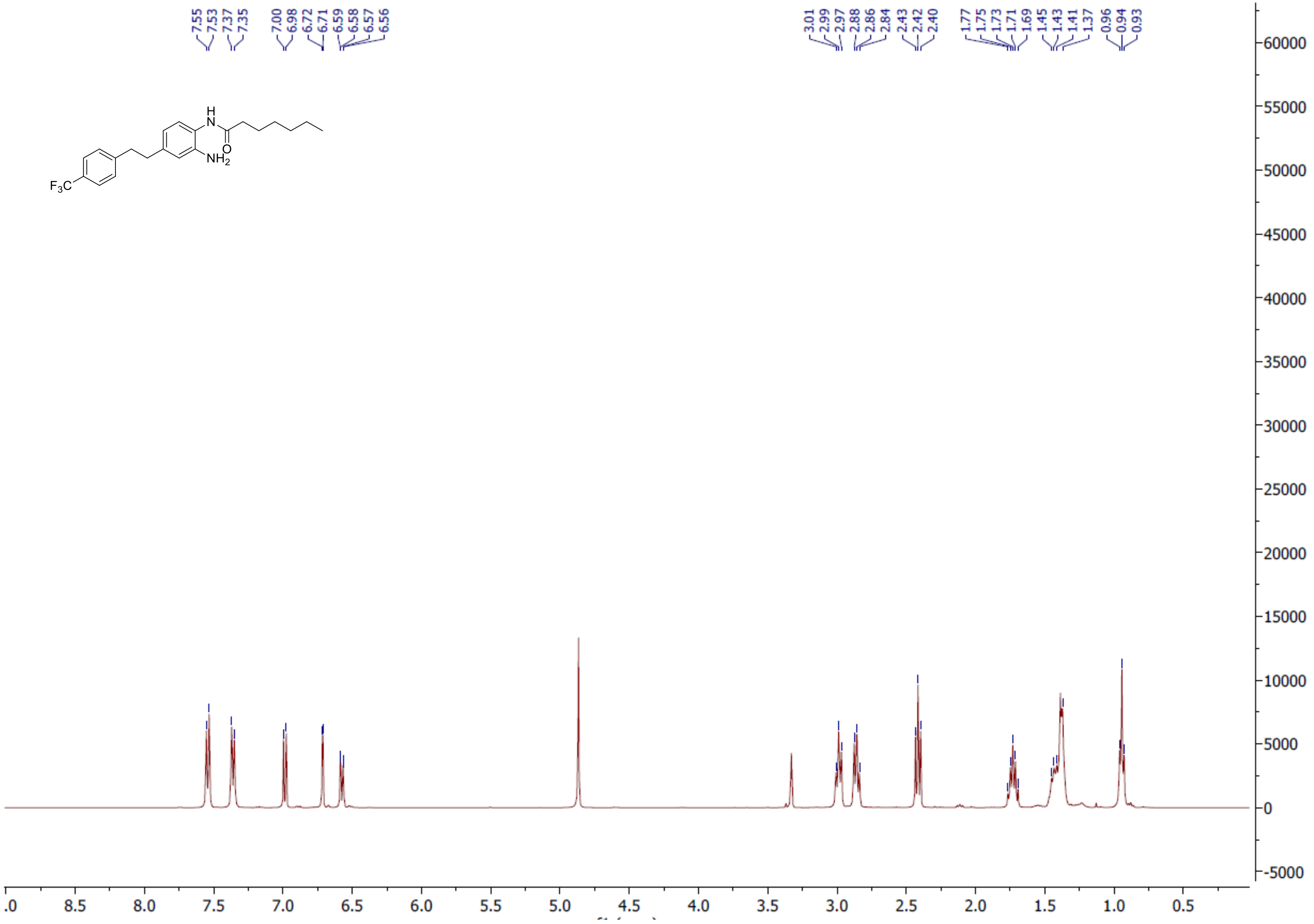

Figure S76: ^{1}H NMR spectra of derivative **67**

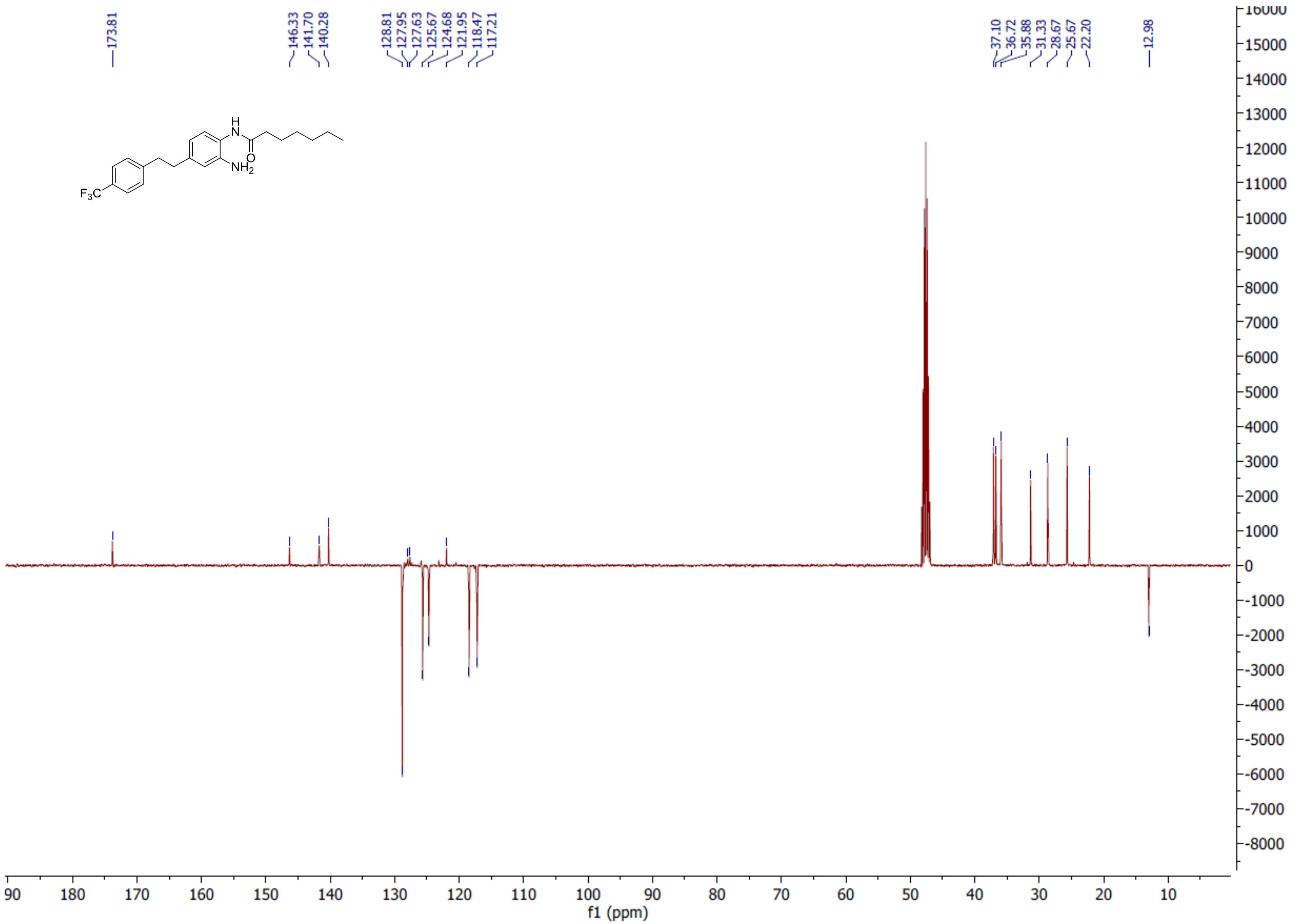

Figure S77: DEPT NMR spectra of derivative **67**

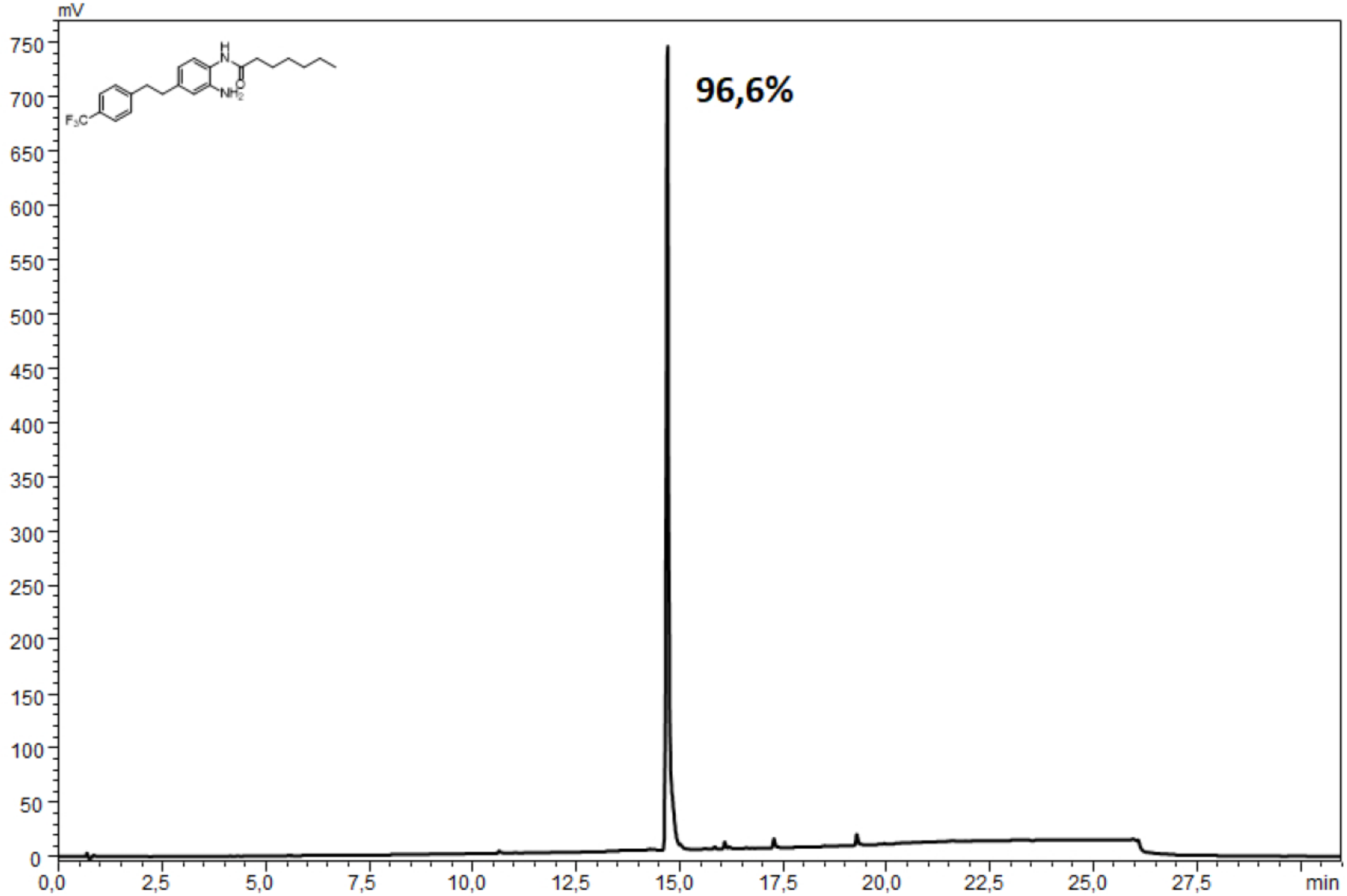

Figure S78: HPLC trace of derivative **67**

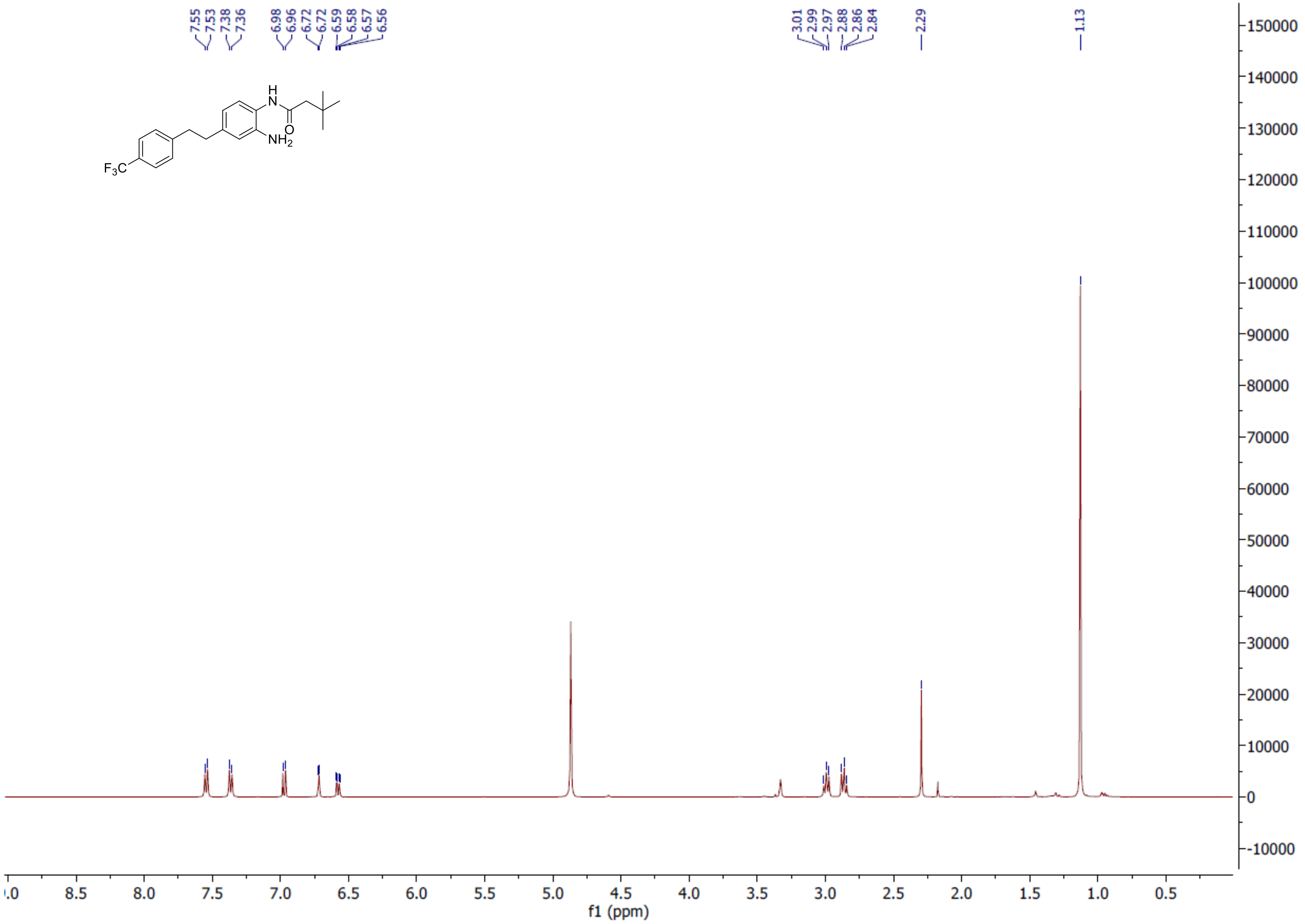

Figure S79: ^{1}H NMR spectra of derivative **68**

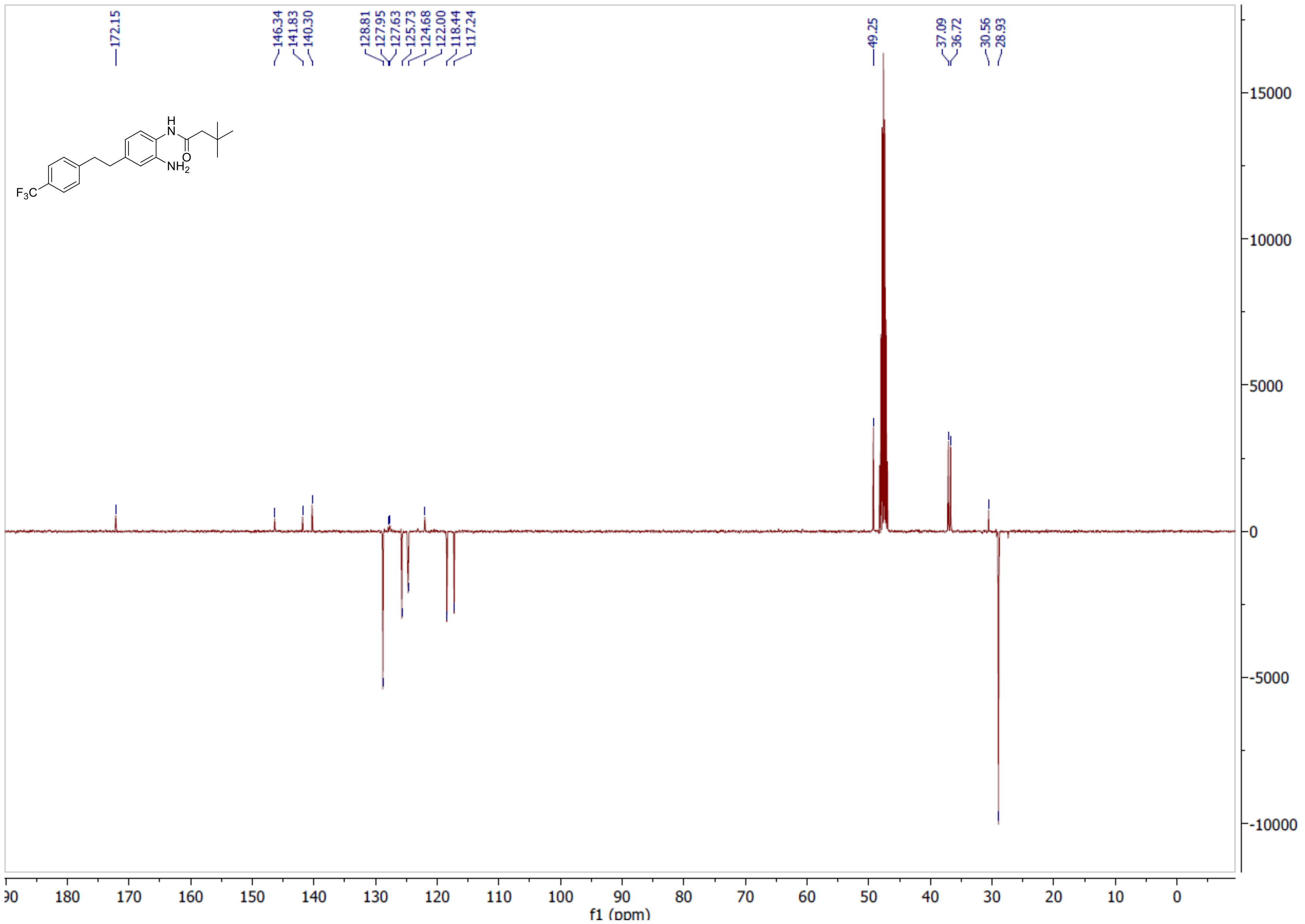

Figure S80: DEPT NMR spectra of derivative **68**

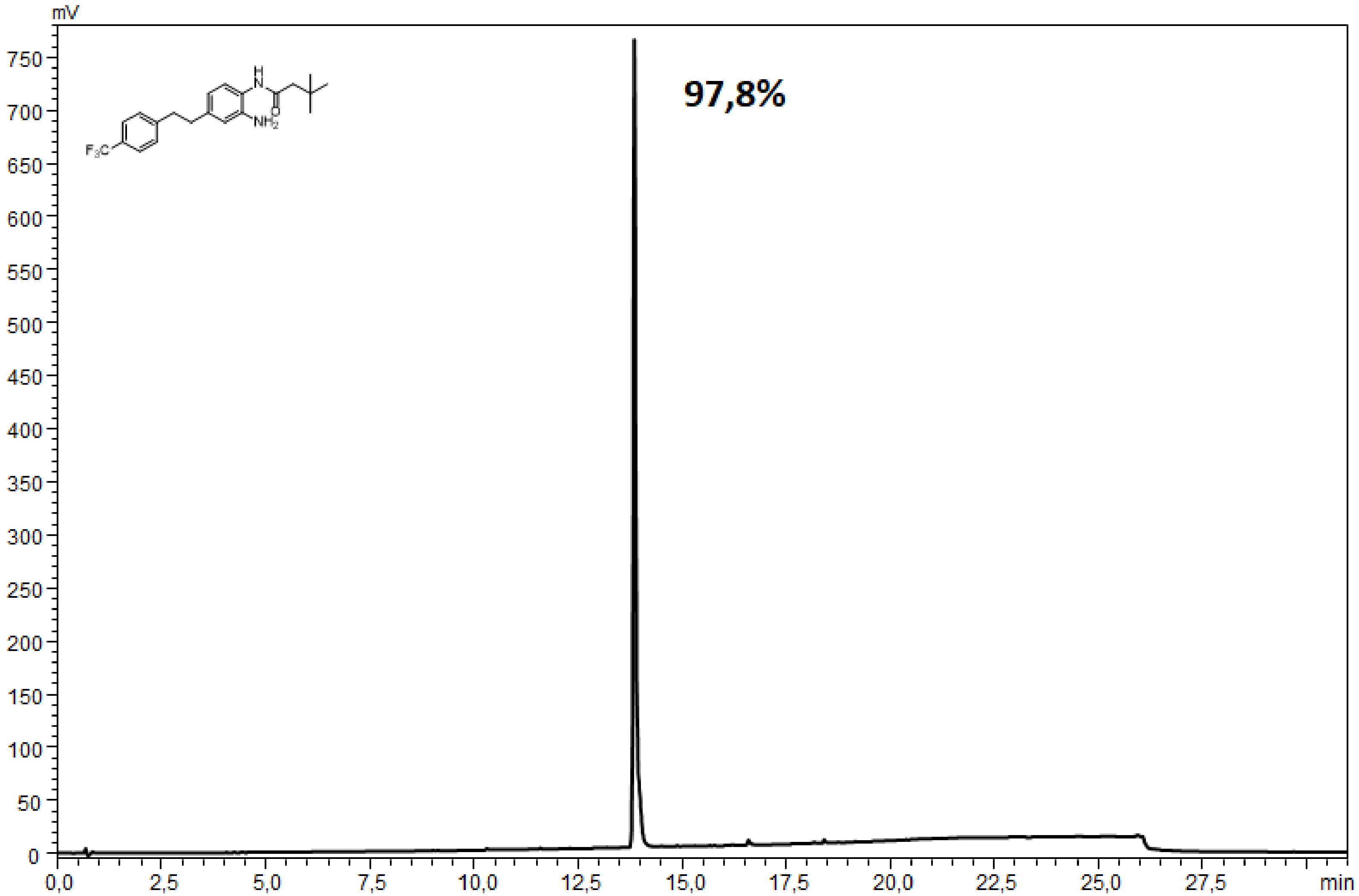

Figure S81: HPLC trace of derivative **68**

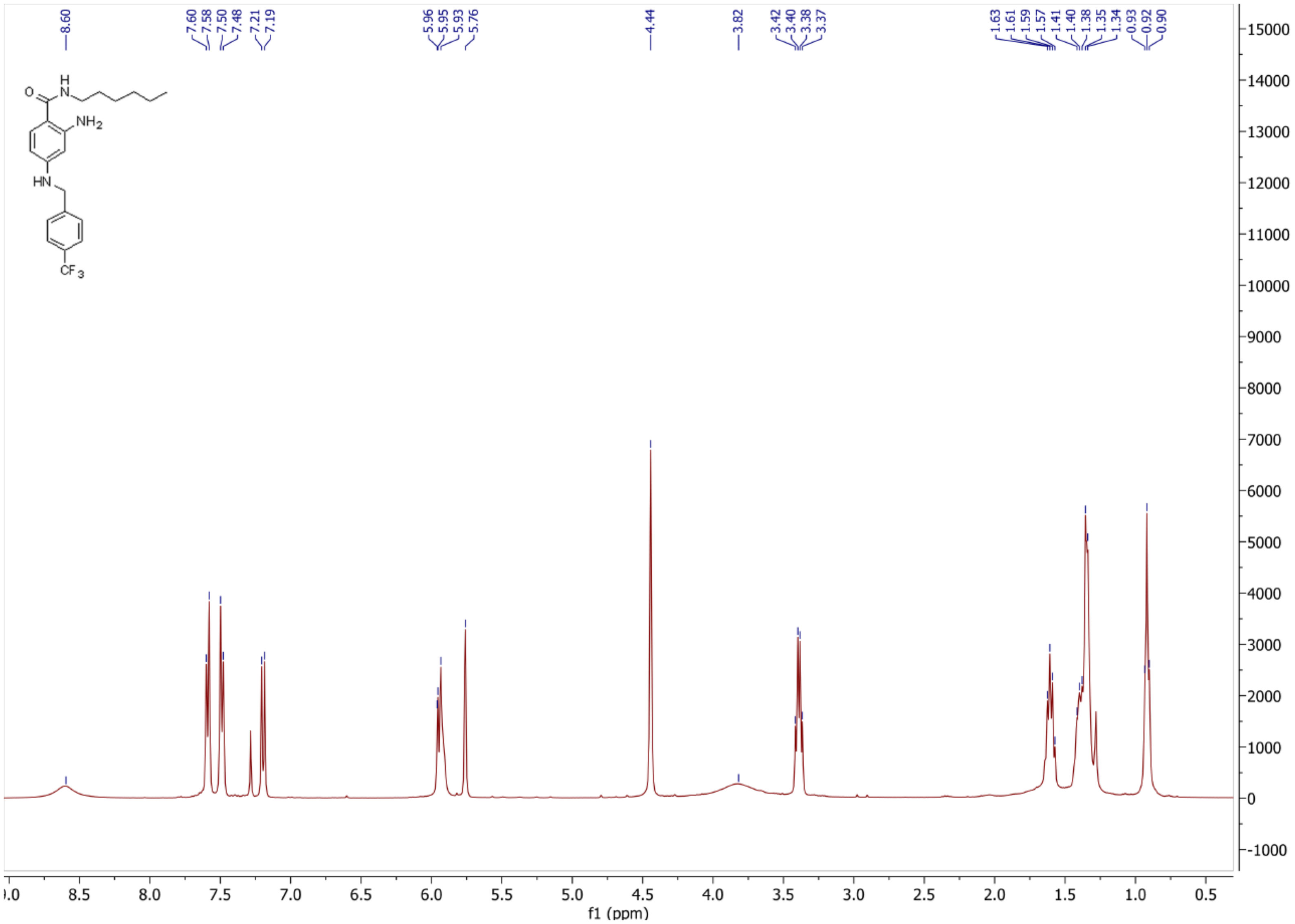

Figure S82:^{1}H NMR spectra of derivative **71**

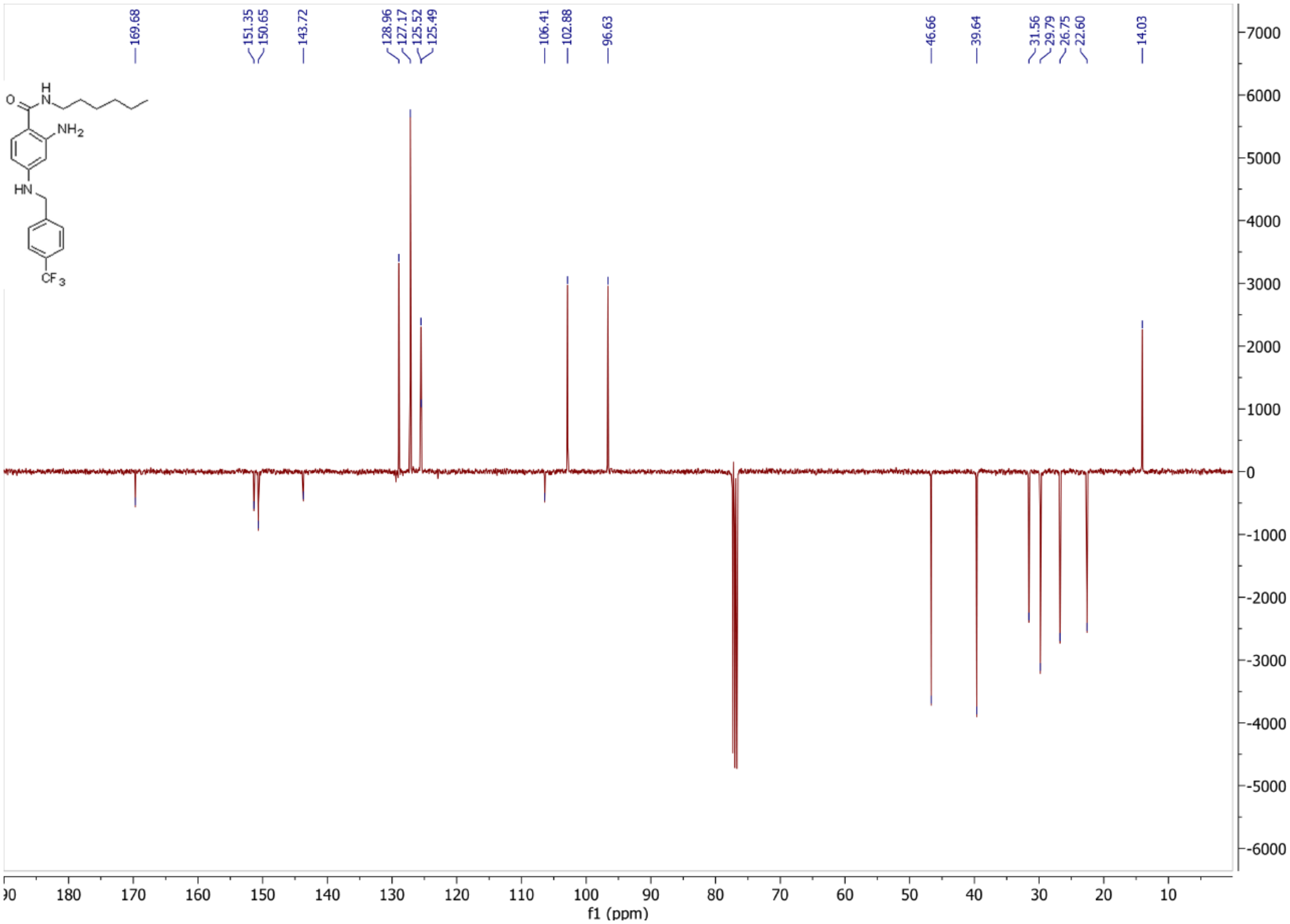

Figure S83: DEPT NMR spectra of derivative **71**

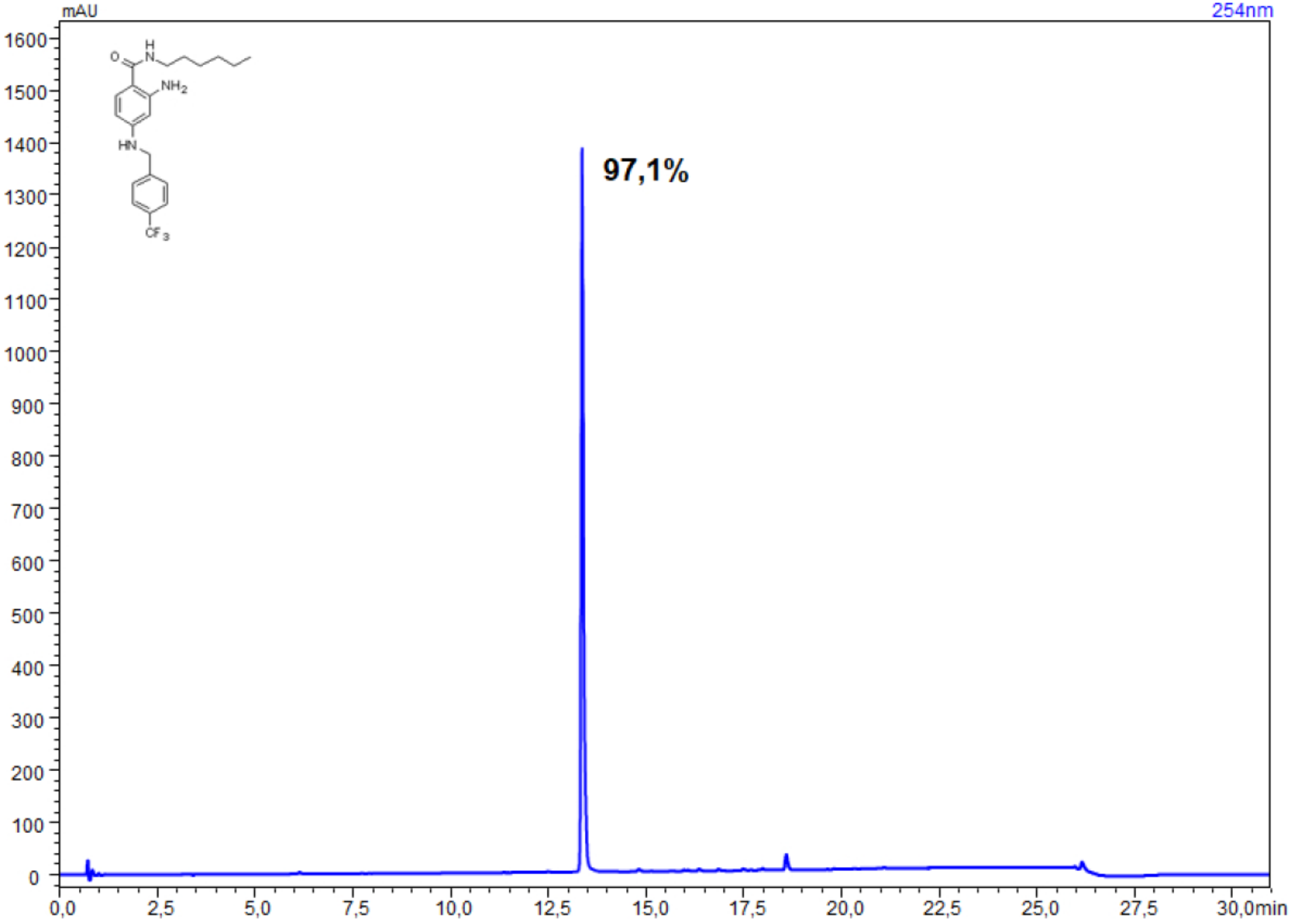

Figure S84: HPLC trace of derivative **71**

Table S1. Photoinduced degradation of retigabine (RET) and its analogues under UV Lighting. Results are expressed as percentage of degradation ± SD.

Compound	% Degradation (3h, UV)	Dimers Formation
**RET**	61.3±0.1	Yes
**13**	30.6 ± 2.2	Yes
**14**	73.2±0.4	Yes
**17**	79.8 ± 4.2	Yes
**19**	64.8 ± 0.9	Yes
**23**	97.7± 1.0	No
**24**	79.8 ± 4.2	No
**41**	19.5±4.7	Yes
**43**	98.4±0.2	Yes
**52**	74.9± 0.1	Yes
**25**	63.3±2.8	No
**26**	99.5±0.1	No
**60**	34.8± 1.8	No

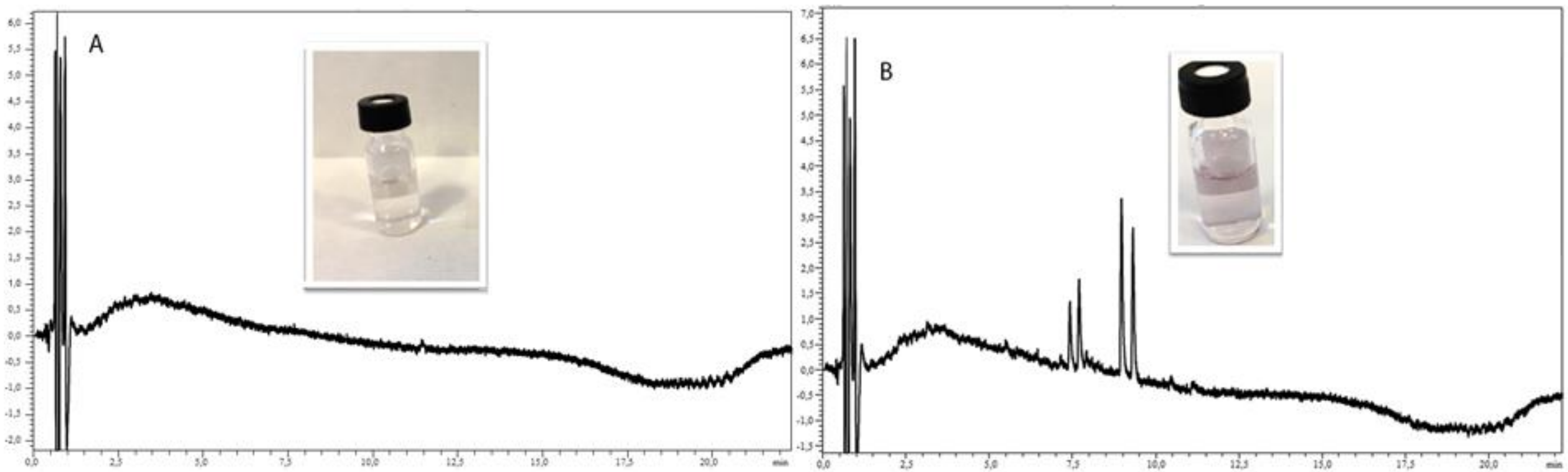

Figure S85: A) HPLC traces of retigabine at 550 nm together with picture of the clear sample vial at T_0; B) HPLC traces of retigabine at 550 nm 3h after exposition to light together with picture of the oxidized sample vial

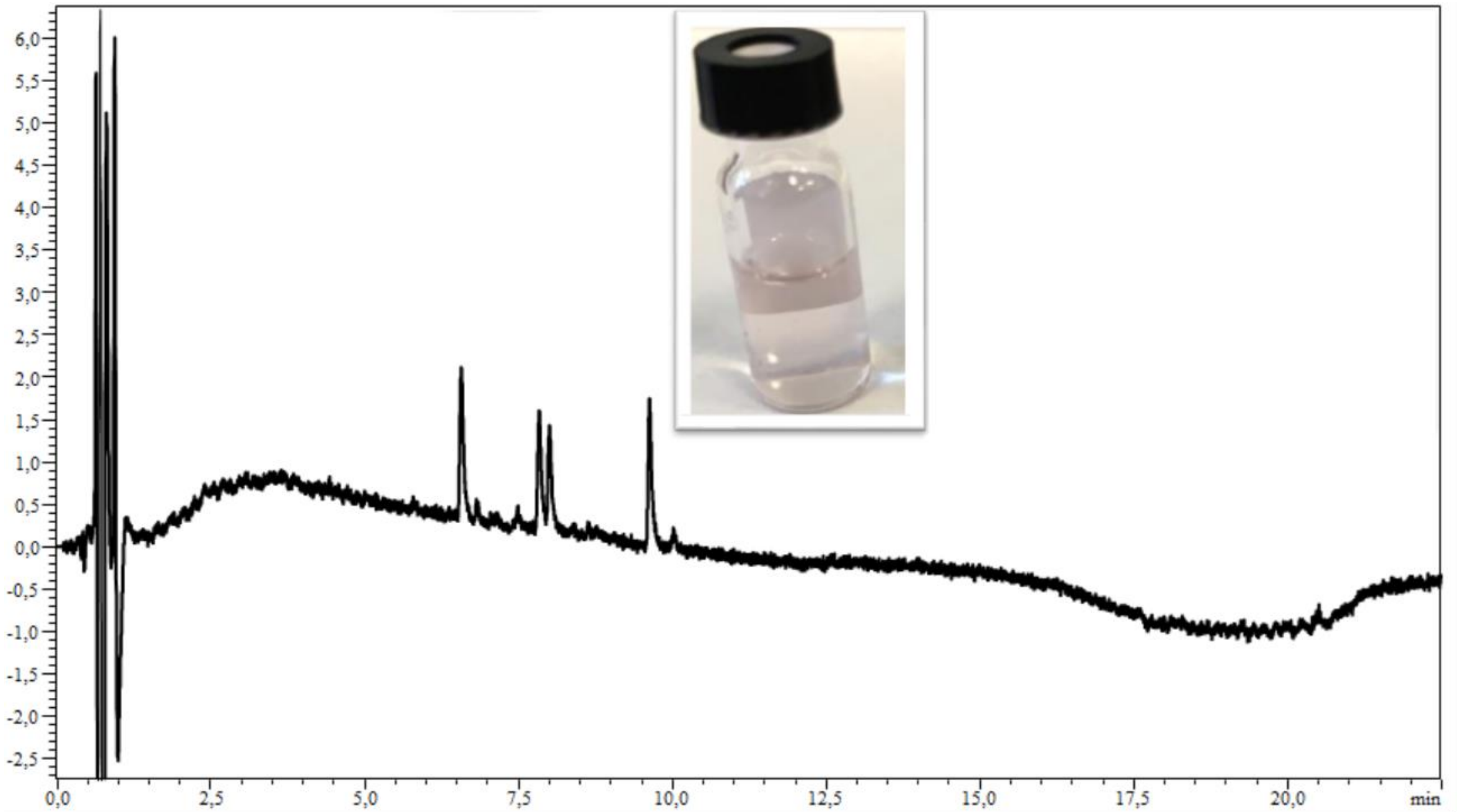

Figure S86: HPLC traces of compound **18** at 550 nm 3h after exposition to light together with picture of the oxidized sample vial

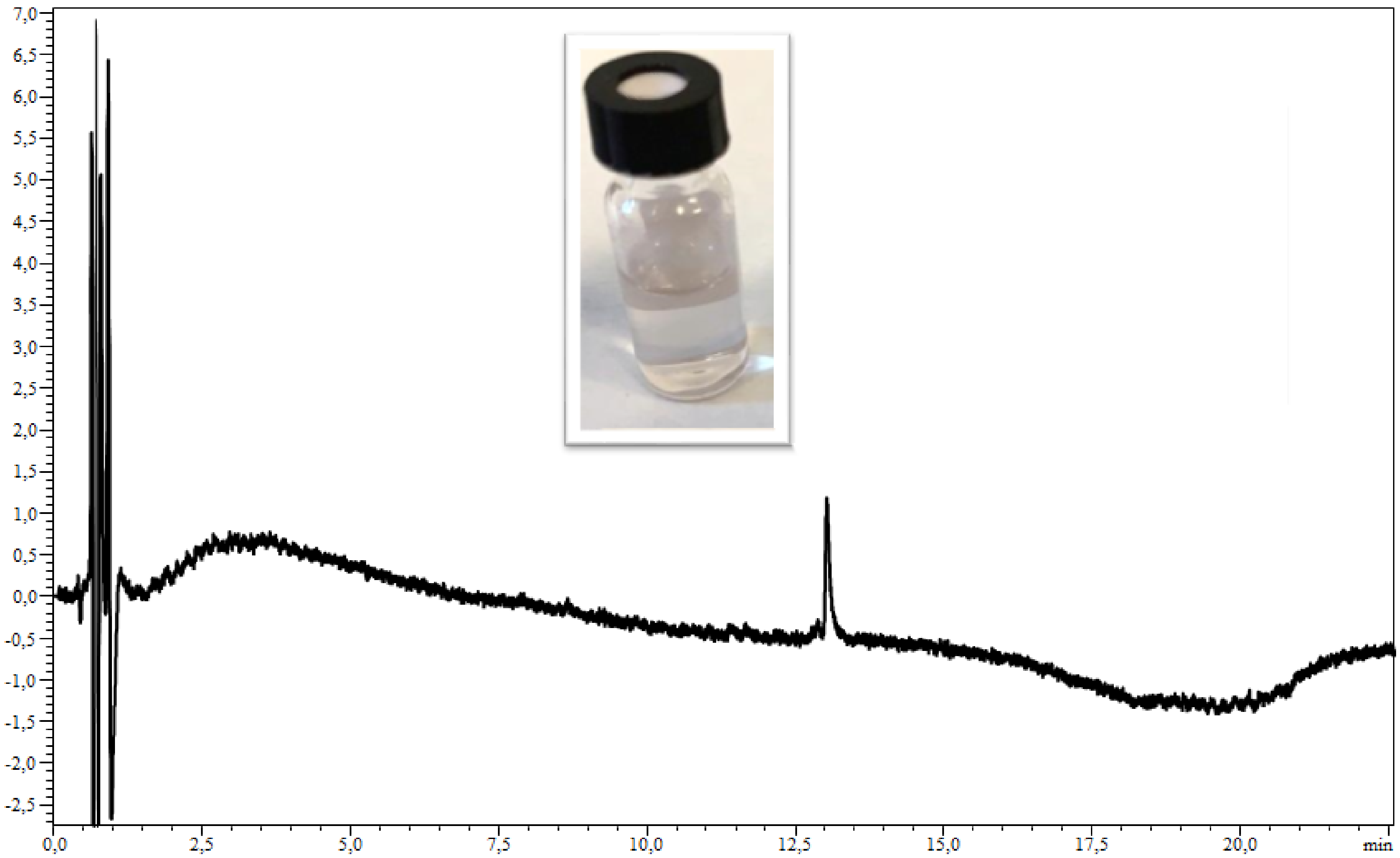

Figure S87: HPLC traces of compound **19** at 550 nm 3h after exposition to light together with picture of the oxidized sample vial

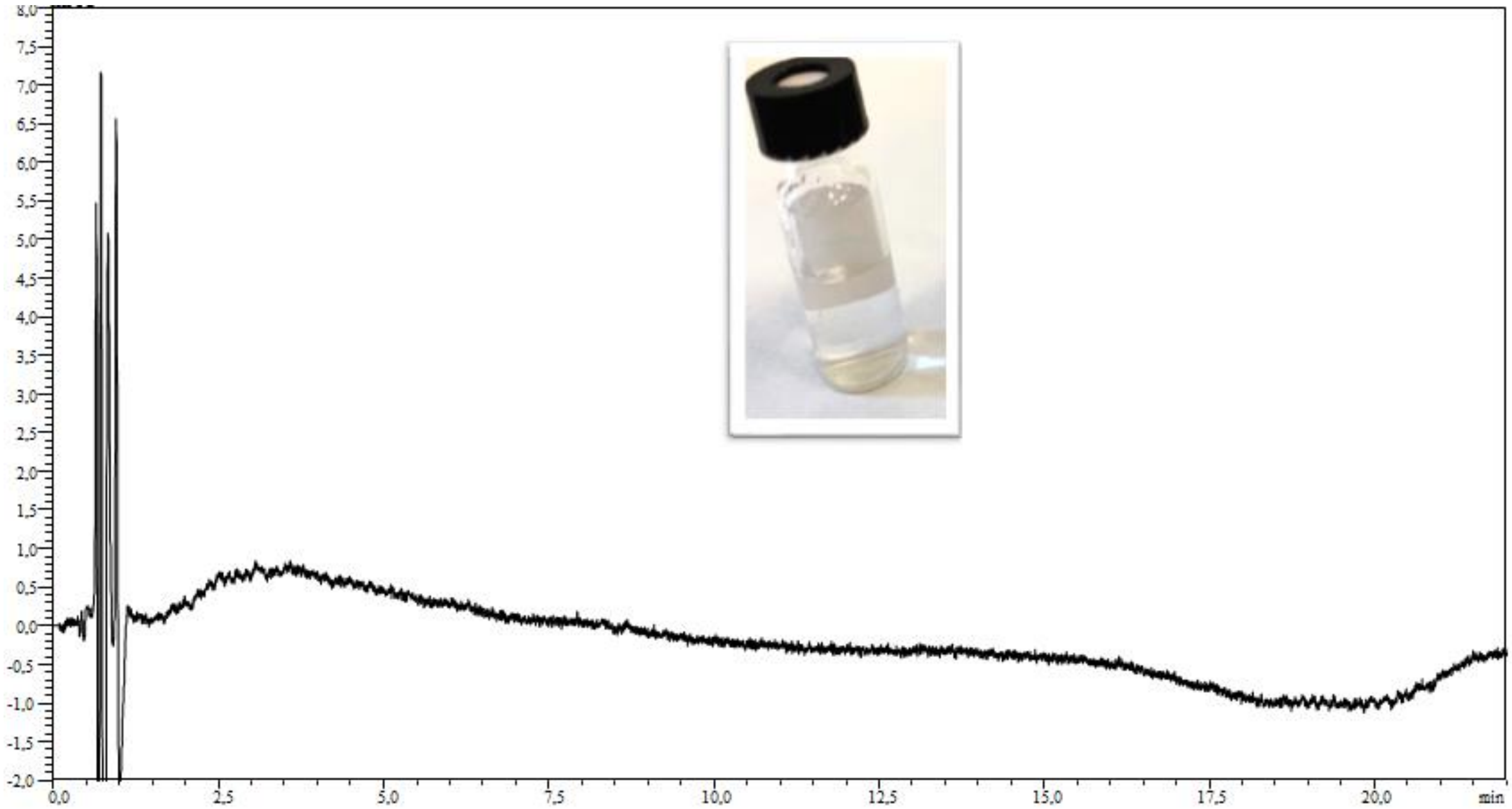

Figure S88: HPLC traces of compound **25** at 550 nm 3h after exposition to light together with picture of the clear sample vial

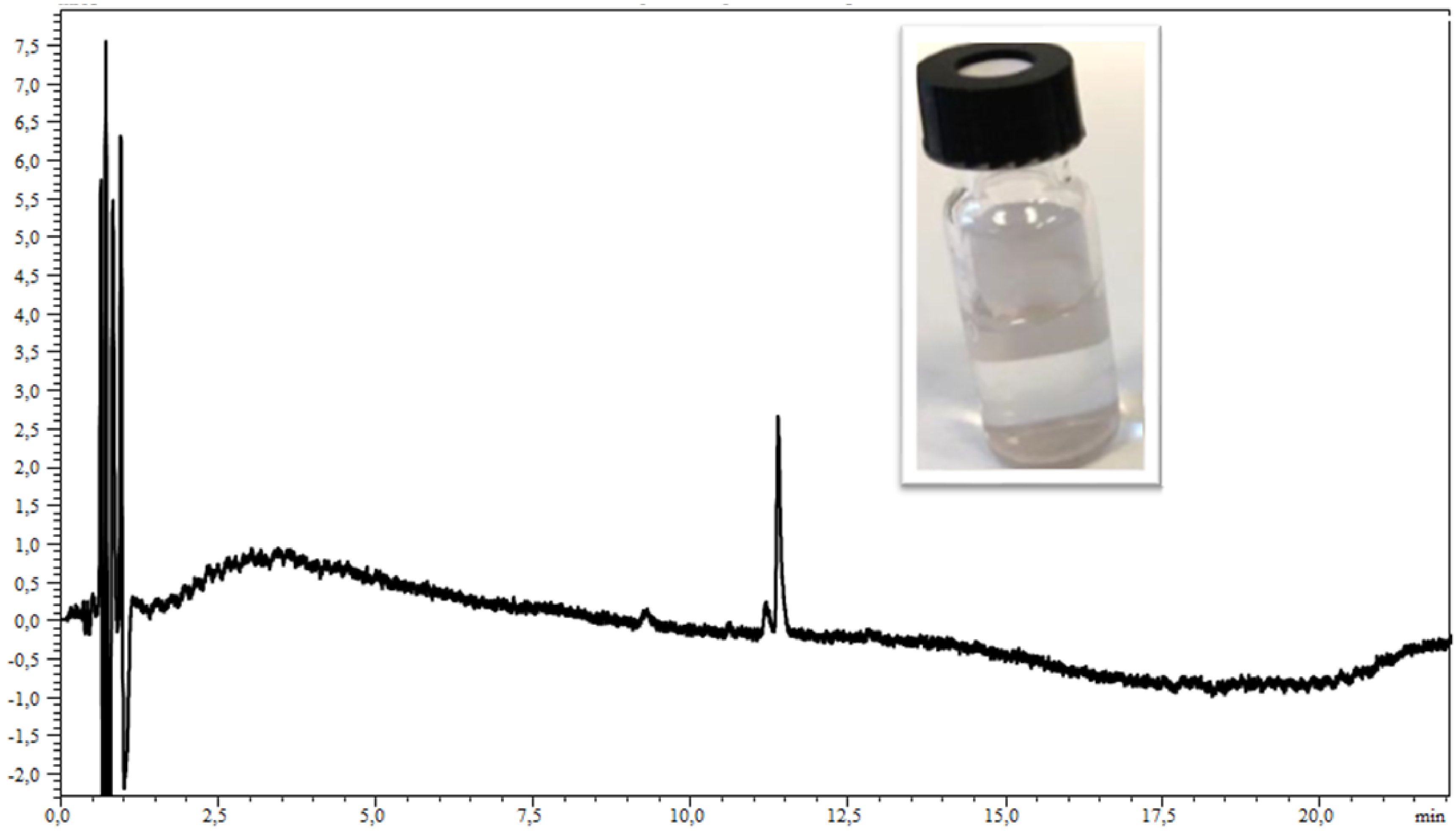

Figure S89: HPLC traces of compound **52** at 550 nm 3h after exposition to light together with picture of the oxidized sample vial

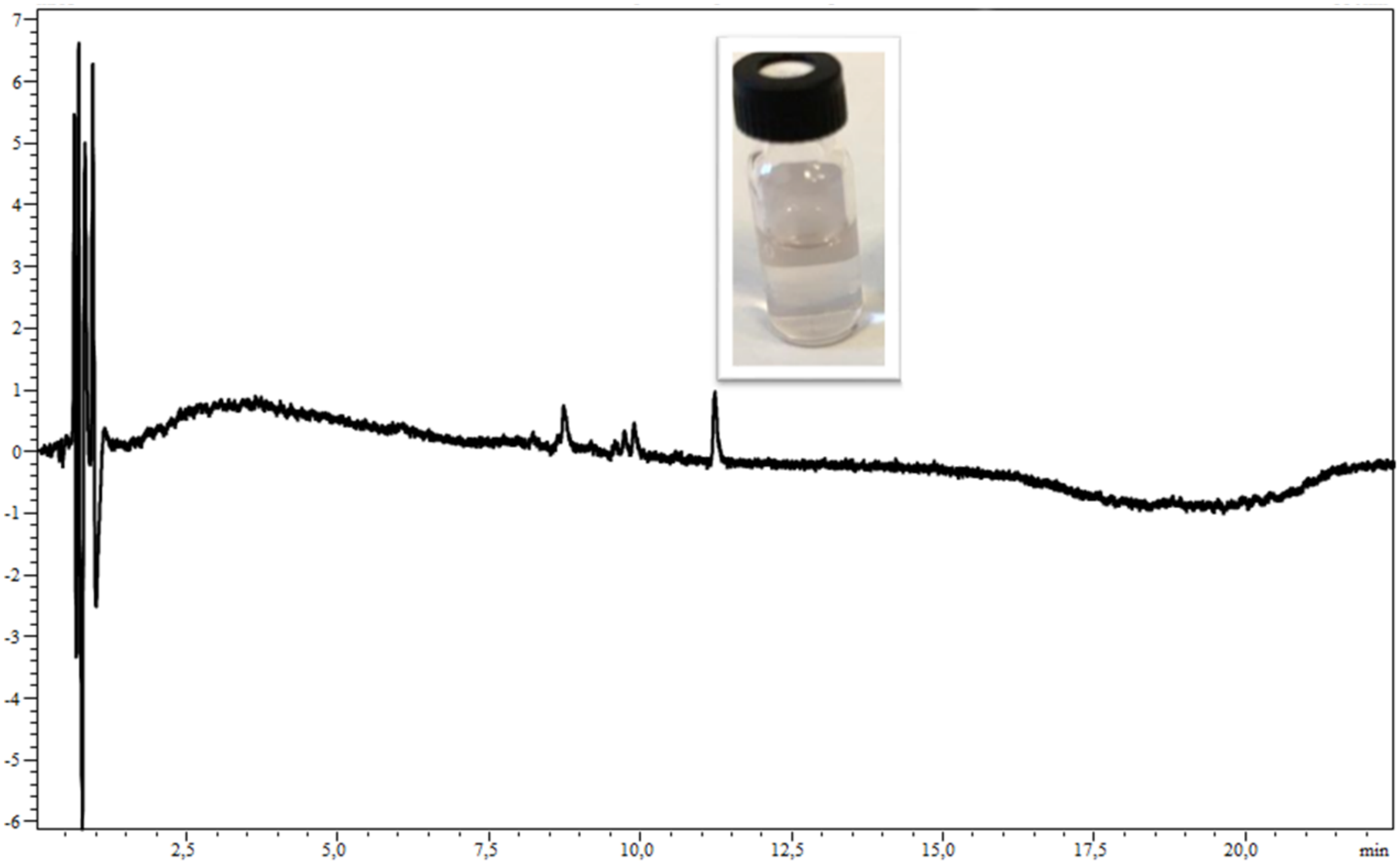

Figure S90: HPLC traces of compound **17** at 550 nm 3h after exposition to light together with picture of the oxidized sample vial

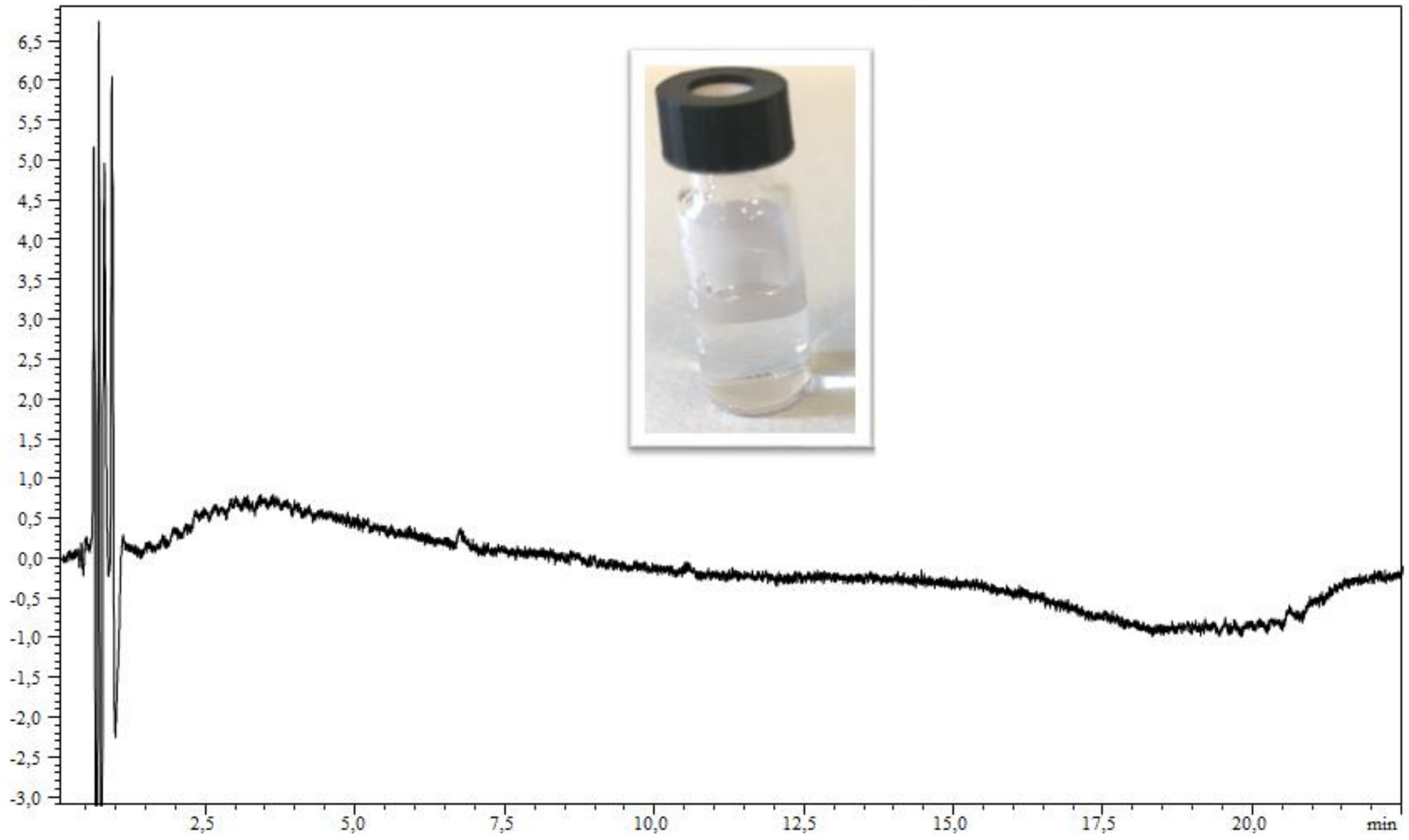

Figure S91: HPLC traces of compound **26** at 550 nm 3h after exposition to light together with picture of the clear sample vial

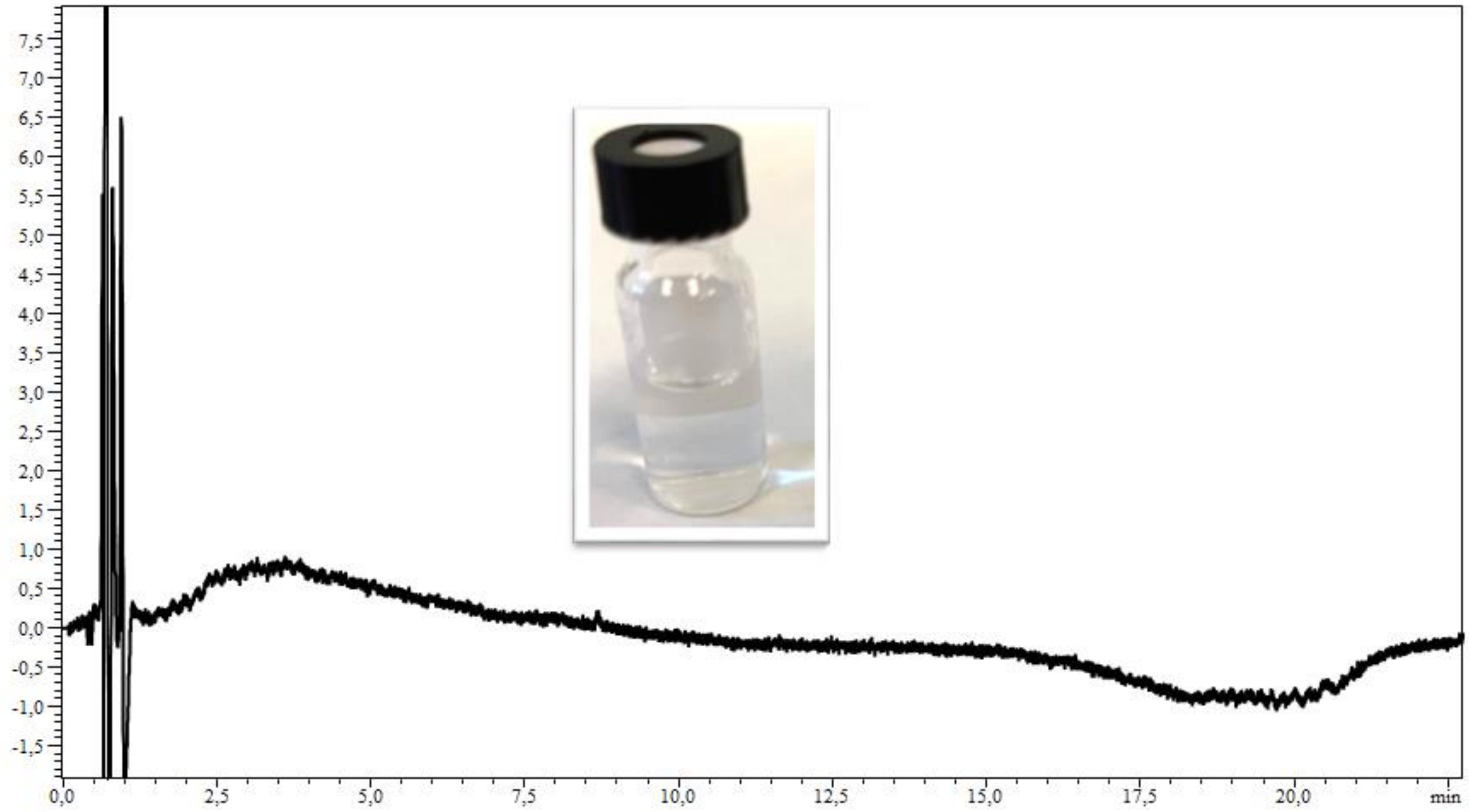

Figure S92: HPLC traces of compound **60** at 550 nm 3h after exposition to light together with picture of the clear sample vial

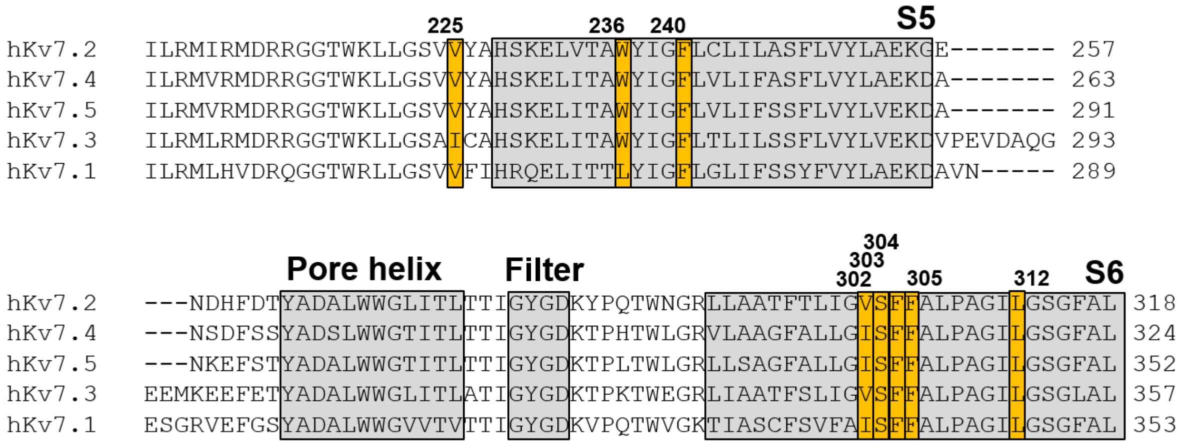

Figure S93. Sequence alignment of the S5, S6 and the S5-S6 intervening linker forming the pore domain of the indicated Kv subunits (www.ebi.ac.uk/Tools/psa/). Numbers above the sequences indicate the residues interacting with retigabine, **23a** and **24a**.

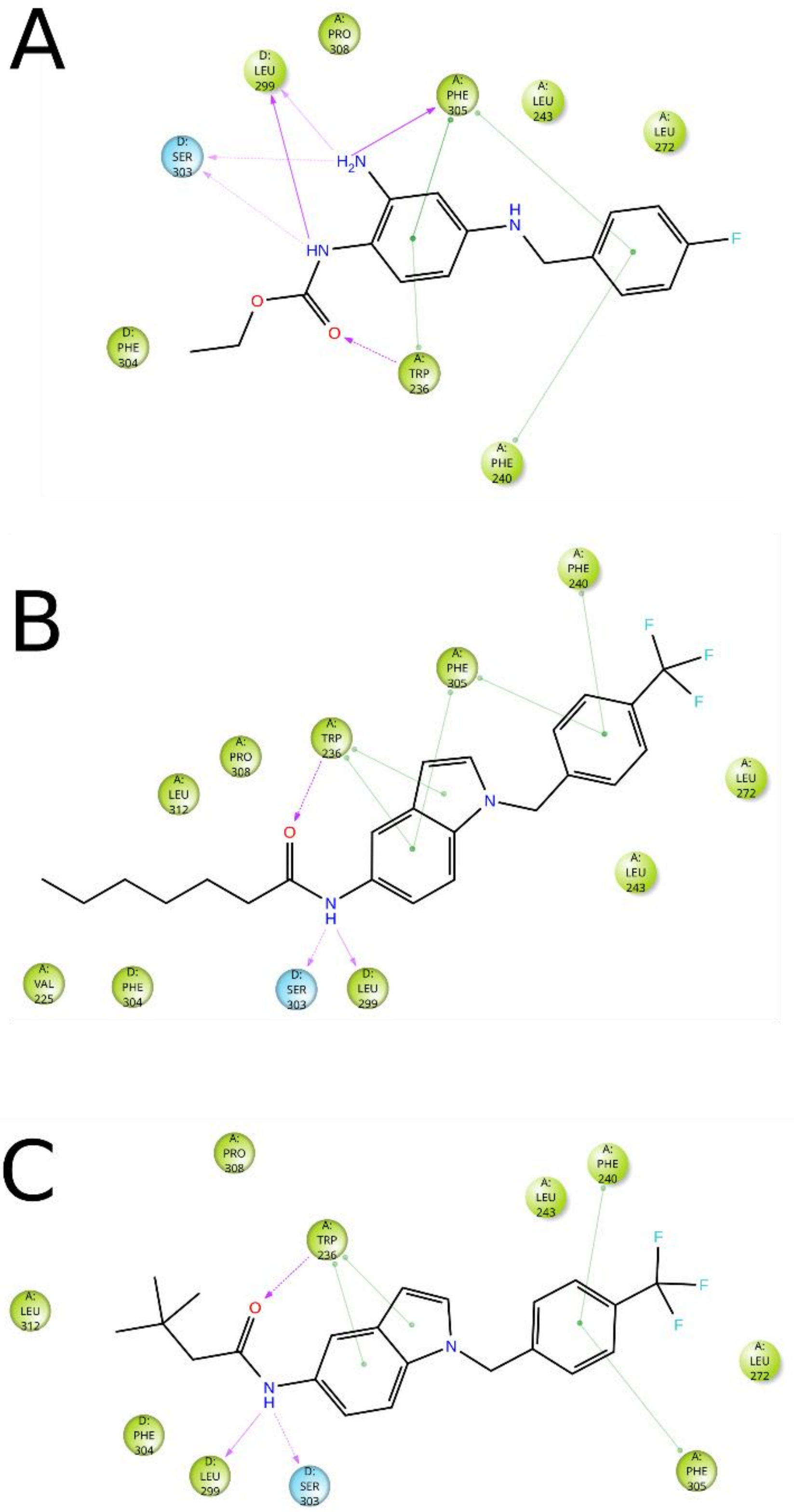

Figure S94. Ligand interaction diagram of 120ns-long MD simulations of Kv7.2 in complex with retigabine (A), **23a** (B) and **24a** (C). Only residues interacting with the ligand for at least 12ns out of 120ns of MD simulation time are shown. Hydrophobic residues are depicted in green, polar ones in cyan. H-bonds are represented by magenta arrows (dashed when side chain atoms are involved); green solid lines represent π-π interactions.

Compound	*V225*	*F304*	*L312*
Retigabine	6.59±0.59 Å	3.83±0.42 Å	4.52±0.38 Å
**23a**	4.21±0.49 Å	4.17±0.46 Å	3.85±0.32 Å
**24a**	4.78±0.35 Å	3.80±0.34 Å	3.88±0.24 Å

Table S2. Average distances between ligands retigabine, **23a** and **24a** and Kv7.2 residues V225, F304 and L312. Minimum distances between ligands and protein atoms are averaged over 120ns-long MD simulations and are reported together with their standard deviations. For the protein, side chain heavy atoms only have been considered, while for the ligands were considered only the heavy atoms at N1 (excluding amide moiety atoms).

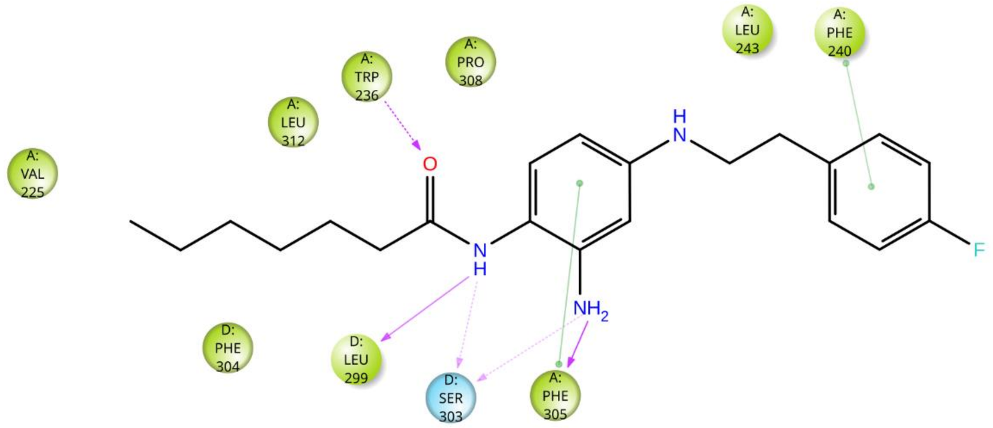

Figure S95. Ligand interaction diagram of 120ns-long MD simulations of Kv7.2 in complex with **42**. Only residues interacting with the ligand for at least 12ns out of 120ns of MD simulation time are shown. Hydrophobic residues are depicted in green, polar ones in cyan. H-bonds are represented by magenta arrows (dashed when side chain atoms are involved); green solid lines represent π-π interactions.

Compound	*F240*	*L272*	*F305*
Retigabine	5.34±0.48 Å	6.57±0.63 Å	6.12±0.34 Å
**42**	5.13±0.45 Å	7.60±0.79 Å	6.96±0.58 Å

Table S3. Average distances between ligands retigabine and **42** and Kv7.2 residues F240, L272 and F305. Distances between ligands and protein atoms are averaged over 120ns-long MD simulations and are reported together with their standard deviations. Distances have been calculated between residues side chain atoms centroids and the centroid of each ligand terminal phenyl group.

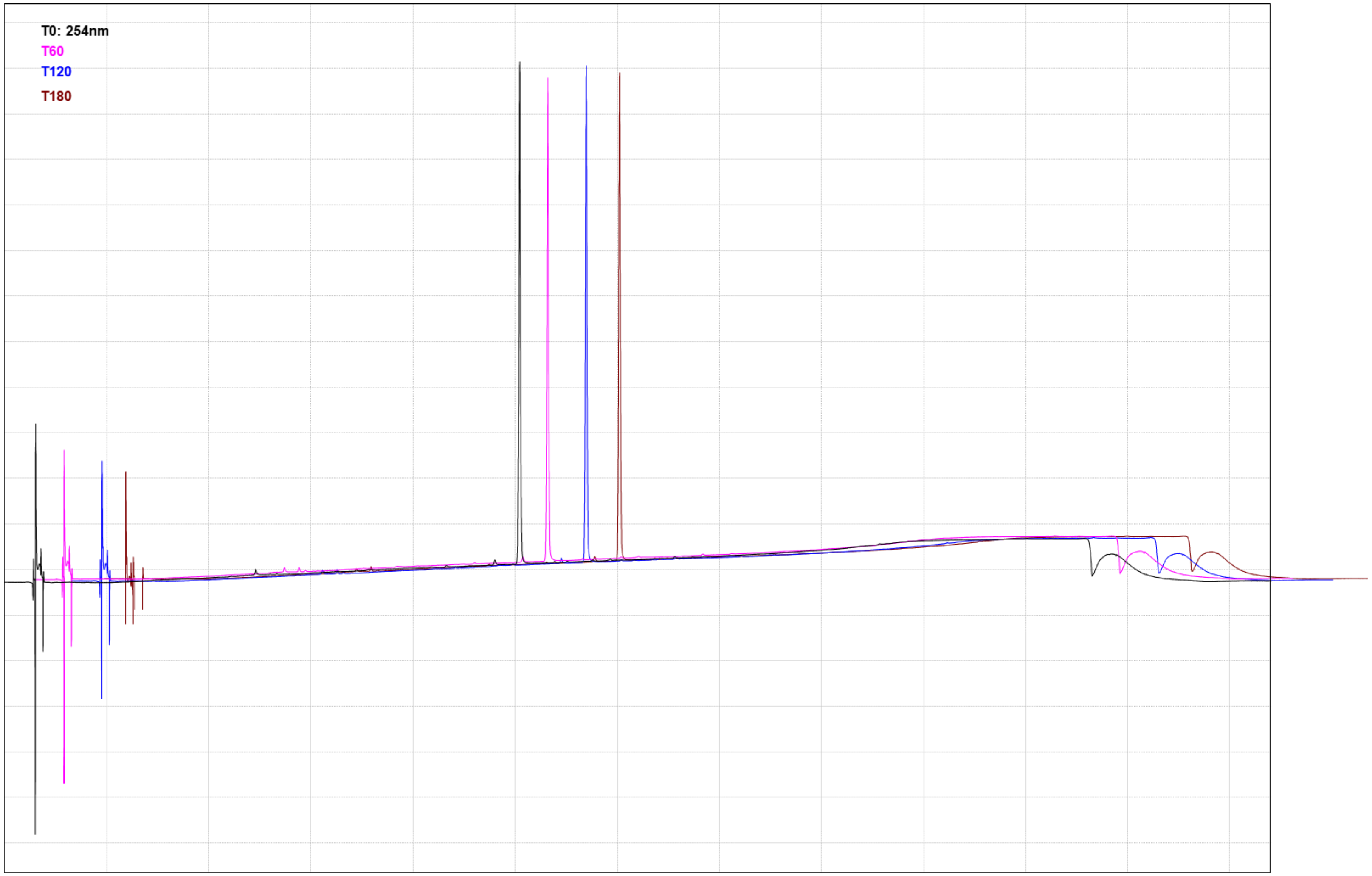

Figure S96. HPLC chromatograms at different time points showing stability of compound **60** solubilized in saline buffer

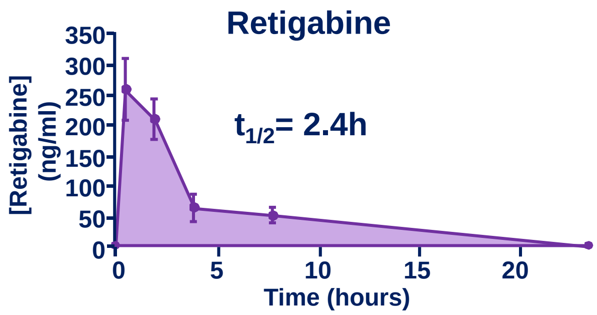

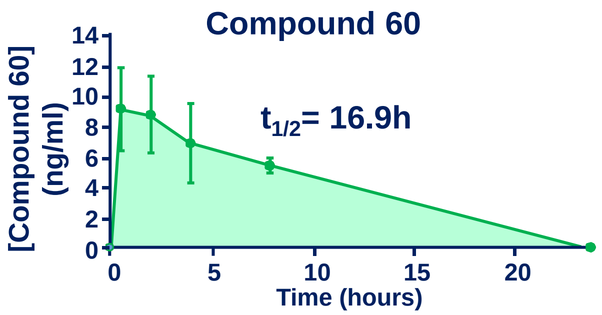

Retigabine Dose= 3mg/Kg	
Time (hours)	ng_{drug}/ml_{plasma}
0.5	256.0 ± 51.5
2	207.2 ± 33.3
4	620 ± 23.0
8	50.0 ± 13.0
24	0

c60 Dose= 0.3mg/Kg	
Time (hours)	ng_{drug}/ml_{plasma}
0.5	9.0 ± 2.7
2	8.7 ± 2.5
4	6.8 ± 2.6
8	5.4 ± 0.5
24	0

Figure S97. Plasma concentration-time curves after a single-dose of Retigabine (3mg/Kg) or compound 60 (0,3 mg/Kg) injected i.p in rats. Values are represented as mean with ±SD (n=3)

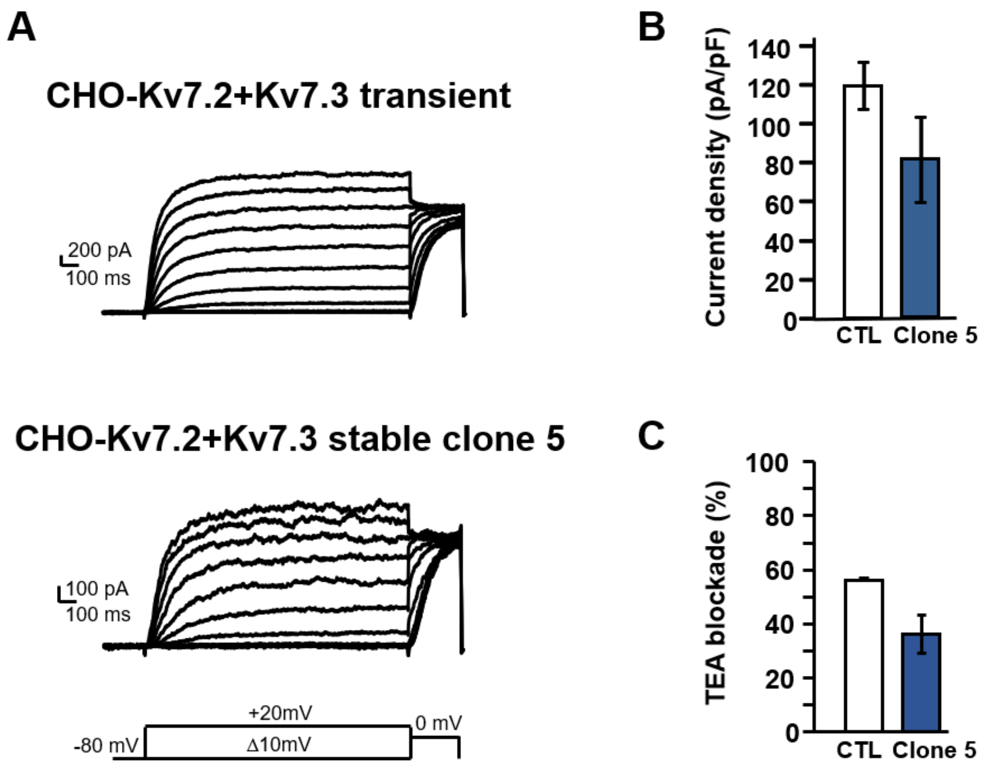

Figure S98. Pharmacological characterization of CHO cells expressing Kv7.2/Kv7.3 channels. (A) Representative current traces from CHO cells transiently- or stably-expressing Kv7.2/Kv7.3 channels. (B) Current density calculated at 0 mV from CHO cells transiently- (CTL) or stably-expressing Kv7.2/Kv7.3 (Clone 5) channels. (C) Current inhibition induced by application of 3 mM tetraethylammonium (TEA).
